# Design principles for cyclin K molecular glue degraders

**DOI:** 10.1038/s41589-023-01409-z

**Published:** 2023-09-07

**Authors:** Zuzanna Kozicka, Dakota J. Suchyta, Vivian Focht, Georg Kempf, Georg Petzold, Marius Jentzsch, Charles Zou, Cristina Di Genua, Katherine A. Donovan, Seemon Coomar, Marko Cigler, Cristina Mayor-Ruiz, Jonathan L. Schmid-Burgk, Daniel Häussinger, Georg E. Winter, Eric S. Fischer, Mikołaj Słabicki, Dennis Gillingham, Benjamin L. Ebert, Nicolas H. Thomä

**Affiliations:** 1 Friedrich Miescher Institute for Biomedical Research, Basel, Switzerland; 2 Department of Biology, University of Basel, Basel, Switzerland; 3 Department of Chemistry, University of Basel, Basel, Switzerland; 4 Institute of Clinical Chemistry and Clinical Pharmacology, University and University Hospital Bonn, Bonn, Germany; 5 Broad Institute of MIT and Harvard, Cambridge, MA USA; 6 Department of Medical Oncology, Dana-Farber Cancer Institute, Boston, MA USA; 7 Department of Biological Chemistry and Molecular Pharmacology, Harvard Medical School, Boston, MA USA; 8 Department of Cancer Biology, Dana-Farber Cancer Institute, Boston, MA USA; 9 CeMM Research Center for Molecular Medicine of the Austrian Academy of Sciences, Vienna, Austria; 10 IRB Barcelona—Institute for Research in Biomedicine, The Barcelona Institute of Science and Technology, Barcelona, Spain; 11 Howard Hughes Medical Institute, Boston, MA USA; 12 Present Address: Monte Rosa Therapeutics, Basel, Switzerland; 13 Present Address: Yale University, New Haven, CT USA; 14 Present Address: VantAI, New York, NY USA

**Keywords:** Small molecules, Structure-based drug design, X-ray crystallography, Cancer therapy

## Abstract

Molecular glue degraders are an effective therapeutic modality, but their design principles are not well understood. Recently, several unexpectedly diverse compounds were reported to deplete cyclin K by linking CDK12–cyclin K to the DDB1–CUL4–RBX1 E3 ligase. Here, to investigate how chemically dissimilar small molecules trigger cyclin K degradation, we evaluated 91 candidate degraders in structural, biophysical and cellular studies and reveal all compounds acquire glue activity via simultaneous CDK12 binding and engagement of DDB1 interfacial residues, in particular Arg928. While we identify multiple published kinase inhibitors as cryptic degraders, we also show that these glues do not require pronounced inhibitory properties for activity and that the relative degree of CDK12 inhibition versus cyclin K degradation is tuneable. We further demonstrate cyclin K degraders have transcriptional signatures distinct from CDK12 inhibitors, thereby offering unique therapeutic opportunities. The systematic structure–activity relationship analysis presented herein provides a conceptual framework for rational molecular glue design.

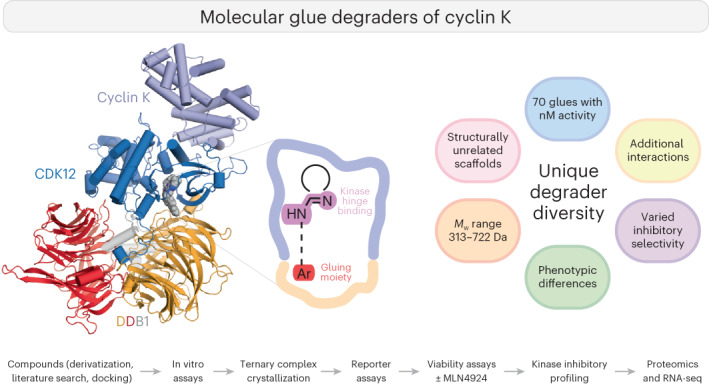

## Abstract (web summary)

Detailed analysis of the structure–activity relationship for cyclin K degraders reveals diverse compounds that acquire glue activity through simultaneous binding to the CDK12 kinase pocket and engagement of several key DDB1 interfacial residues.

## Main

The modulation of protein–protein interactions has become an important avenue of therapeutic intervention^1,2^. Recent advances in targeted protein degradation illustrate that compound-induced proximity between a ubiquitin ligase and a target protein can lead to target ubiquitination and degradation^3–5^. Of particular interest are molecular glue degraders, which are drug-like compounds that leverage complementary protein–protein interfaces to induce cooperative ligase–target interactions leading to target depletion^6^. Despite the clinical success of thalidomide derivatives and several recent, largely serendipitous discoveries of other molecular glue degraders^7–13^, the rules that govern their discovery, design and rational optimization remain poorly defined^14^.

We recently reported that CR8 (**1**), a preclinical cyclin-dependent kinase (CDK) inhibitor, is a molecular glue degrader that binds CDK12–cyclin K and recruits the DDB1–CUL4–RBX1 E3 ligase core to ubiquitinate cyclin K (ref. ^15^). Structural studies revealed that CR8 binds the ATP pocket of CDK12, leaving a phenylpyridine moiety exposed on the kinase surface to induce complex formation with the ligase adaptor DDB1. This places the CDK12-associated cyclin K in a position normally adopted by CRL4 substrates. CR8 therefore hijacks DDB1 in a manner that bypasses the requirement of a canonical substrate receptor (DCAF) typical for CUL4-based E3 ligases. The extensive (~2,100 Å^2^) DDB1–CDK12 interface is highly complementary and reveals a helical motif in the CDK12 C-terminal extension that engages DDB1 in a DCAF-like manner. While a basal affinity of ~50 µM was measured between DDB1 and CDK12–cyclin K in the absence of a compound, CR8 enhanced this affinity into the low nanomolar range, triggering polyubiquitination and degradation of cyclin K (ref. ^15^). CR8 therefore shows dual activity, promiscuous CDK inhibition and selective cyclin K degradation, which leads to robust inactivation of CDK12, an emerging therapeutic target in oncology and beyond^16–19^. Recently, chemically distinct compounds have been found to degrade cyclin K (refs. ^8,10,11,20,21^), but as several lack any obvious chemical similarity to CR8 (refs. ^8,10,11^), their precise mode of action remained unknown.

In this Article, we perform a systematic dissection of the cyclin K degrader structure–activity relationship (SAR), investigate the in vitro and cellular activity of the compounds and present crystal structures of 28 glue-induced ternary complexes. Our findings inform on how target-binding compounds can acquire gain-of-function properties, yielding generalizable learnings for molecular glue degrader design.

## Results

### CR8 tolerates modifications retaining molecular glue activity

**Fig. 1:**
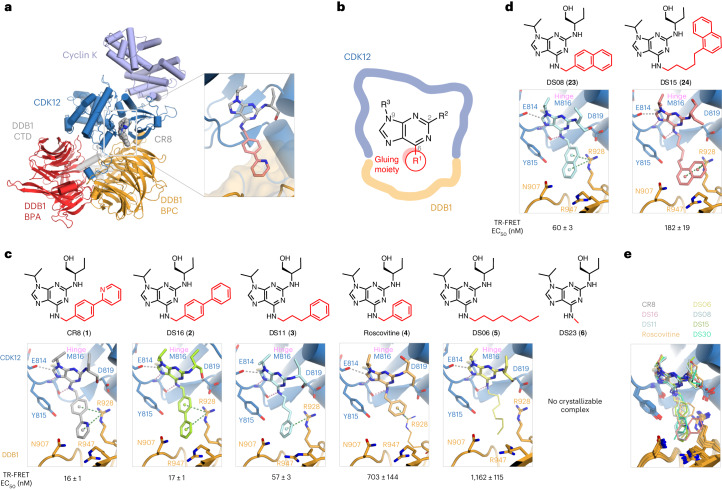
Modifications of the CR8 scaffold preserve its molecular glue activity. **a**, Crystal structure of the DDB1–CR8–CDK12–cyclin K complex (PDB: 6TD3) (ref. ^15^). The zoomed panel depicts the binding mode of CR8, with the phenylpyridine moiety (gluing moiety; pale red) engaging DDB1 (surface representation). **b**, Chemical structure of the 2,6,9-trisubstituted purine core. The R_1_ group is referred to as the gluing moiety and shown in red throughout. **c**, Chemical structures of CR8, DS16, DS11, roscovitine, DS06, DS23 and ternary complex crystal structures of the DDB1–CDK12 interfaces induced by those compounds, listed from best to worst binder, and their associated TR-FRET EC_50_ values. Corresponding TR-FRET curves can be found in Extended Data Fig. 1e. **d**, Chemical structures of DS08 and DS15 and ternary complex crystal structures of interfaces induced by those compounds. Corresponding TR-FRET curves can be found in Extended Data Fig. 1i. **e**, Overlay of ternary complex structures from **c** and **d**. In **c** and **d**, interactions are represented by dashed lines. Hydrogen bonds to the hinge region are shown in pink, other hydrogen bonds in yellow, aromatic H-bonds in gray, and π–cation interactions in green. Regions with no unambiguous *F*_o_–*F*_c_ density at 1*σ* are displayed with a smaller stick radius. Density maps, omit maps and interaction distances can be found in Supplementary Figs. 1–3.

**Extended Data Fig. 1:**
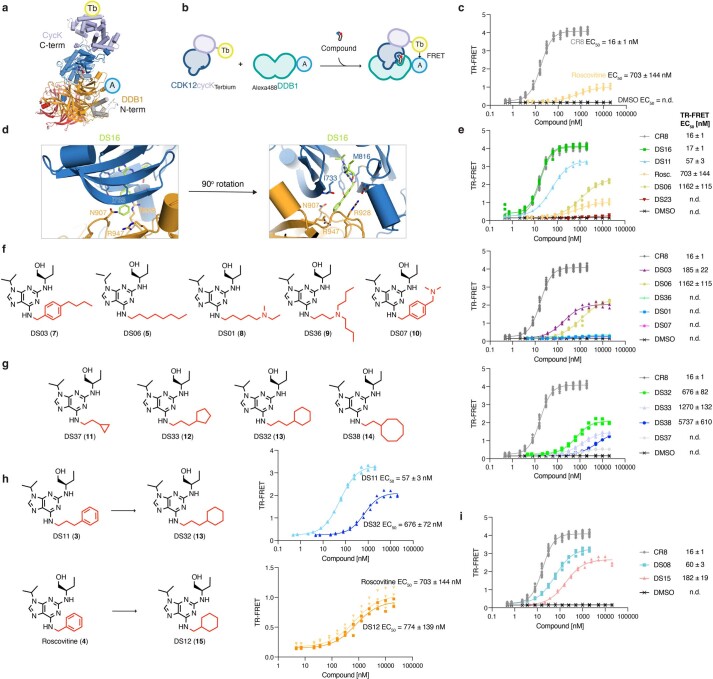
Mapping of interfacial pocket dimensions and key interactions with CR8 derivative series. **a**, Fluorescent label positions chosen for the optimized TR-FRET assay. **b**, Schematic of the compound titration used for ternary complex formation assessment. **c**, Optimized in vitro TR-FRET complex formation assay for CR8 and roscovitine reveals a large difference in activity (much larger than previously reported^15^) between the two compounds in accordance with the lack of cyclin K degradation activity of roscovitine in cells^15^. The assay is therefore an appropriately sensitive readout for the evaluation of closely related compounds. **d**, CDK12 loop (a.a. 731-743) that encloses the active site, with the Ile733 sidechain oriented towards the compound. This loop is omitted from most figure panels for clarity. **e**, In vitro TR-FRET complex formation assay for compound shown in Fig. 1c. **f**, Chemical structures of a series of derivatives bearing aliphatic chains at the R_1_ position listed from best to worst binder (left). In vitro TR-FRET complex formation assay for these compounds (right). **g**, Chemical structures of a series of derivatives bearing saturated rings at the R_1_ position listed from best to worst binder (left). In vitro TR-FRET complex formation assay for these compounds (right). **h**, The impact of aromaticity on ternary complex formation. Chemical structures of two aromatic-aliphatic pairs and the associated in vitro TR-FRET compound titration results. **i**, In vitro TR-FRET complex formation assay for compounds shown in Fig. 1d. Crystal structures with compounds DS08 and DS15 are displayed in Fig. 1e. (**c**, **e**-**i**) Individual replicates are shown (n = 17 for CR8; n = 4 for DS06, DS08, roscovitine; n = 2 for others). Source data

To understand how chemically dissimilar compounds can commit cyclin K for degradation, we first focused on the CR8 scaffold. The DDB1–CR8–CDK12–cyclin K complex crystal structure found the compound bound at the CDK12–DDB1 interface, with the phenylpyridine moiety of CR8 protruding towards DDB1 and bridging this interface (Fig. 1a,b)^15^. We set out to dissect the CR8 SAR and explore whether moieties other than phenylpyridine (substituents at position R_1_, hereafter referred to as ‘gluing moieties’ (C6 position in the 2,6,9-trisubstituted nomenclature; Fig. 1b)) promote CDK12–DDB1 interactions. For this, we developed an optimized time-resolved fluorescence energy transfer (TR-FRET) assay that accurately measured in vitro complex formation between CDK12–cyclin K and DDB1 in the presence of small molecules and confirmed that CR8 induces tight complex formation (EC_50_ = 16 ± 1 nM) (Extended Data Fig. 1a–c). Biophysical characterization was complemented by crystallization of compound-induced ternary complexes when possible (Supplementary Tables 1 and 2 and Supplementary Figs. 1–3).

First, we explored the CR8 SAR through the gradual simplification of its gluing moiety. A derivative bearing a biphenyl substituent (DS16 (**2**)) instead of the phenylpyridine displayed the same binding mode as CR8, with the purine core held in the CDK12 active site by two hydrogen bonds to the kinase hinge (Met816), a region connecting the N- and C-terminal lobes of the catalytic domain (Fig. 1c). The compound is enclosed by a CDK12 loop (amino acids (a.a.) 731–743, with Ile733 approaching the ligand (Extended Data Fig. 1d); omitted in most figures for clarity), and the gluing moiety engages in π–cation interactions with Arg928 of DDB1 (Fig. 1c). Interestingly, DS16 showed activity equivalent to CR8, demonstrating that the hydrogen bond acceptor (HBA) in the ring is not required for robust complex formation in vitro (Extended Data Fig. 1e). While a phenyl ring on a propyl chain (DS11 (**3**)) supported robust complex formation (EC_50_ = 57 ± 3 nM), shortening the chain by two alkyl carbons yielded roscovitine (**4**), which gave much poorer recruitment (EC_50_ = 703 ± 144 nM) (Fig. 1c and Extended Data Fig. 1e). Strikingly, even an octyl chain (DS06 (**5**)) protruding into DDB1 supported the formation of an analogous complex, albeit with a lower affinity (EC_50_ = 1,162 ± 115 nM) (Fig. 1c and Extended Data Fig. 1e). As the ternary complex appeared surprisingly permissive to changes in the CR8 scaffold, we asked whether simply filling the kinase pocket would be sufficient to facilitate the interaction. However, a di-substituted purine core, with only a methyl in R_1_ (DS23 (**6**)), did not appreciably promote the binding (Fig. 1c and Extended Data Fig. 1e), demonstrating that more extensive engagement of DDB1 residues by the compound is required for molecular glue activity.

### R_1_ interactions govern DDB1 recruitment

**Extended Data Fig. 2:**
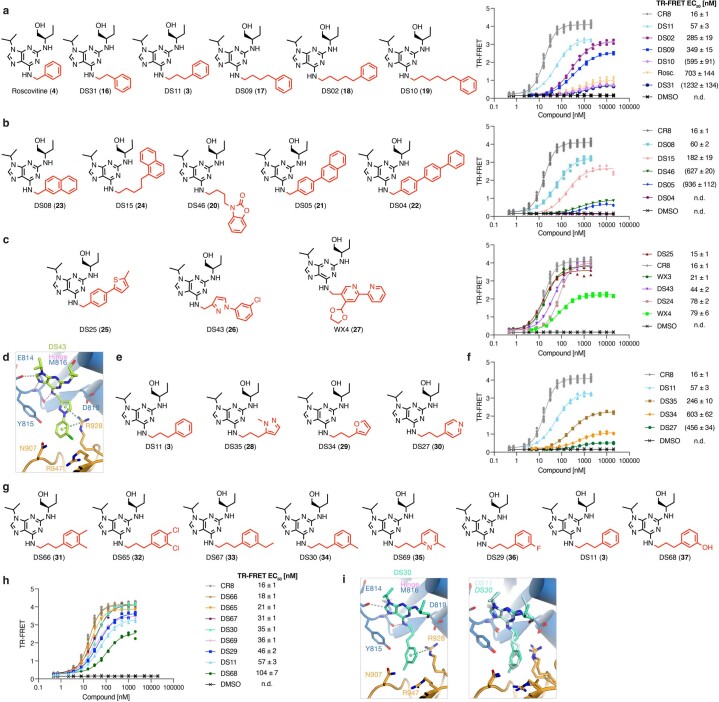
Miscellaneous modifications of CR8-like scaffolds. **a**, Chemical structures of a series of derivatives bearing an alkyl phenyl R_1_ group with the alkyl linker varying in length listed from best to worst binder (left). In vitro TR-FRET complex formation assay for the compounds shown (right). **b**, Chemical structures of several derivatives containing fused or multiple rings listed from best to worst binder (left). In vitro TR-FRET complex formation assay for the compounds shown (right). **c**, Chemical structures of various CR8 derivatives listed from best to worst binder (left). In vitro TR-FRET complex formation assay for these compounds (right). **d**, Ternary complex crystal structure with DS43. **e**, Chemical structures of derivatives bearing heterocyclic gluing moieties listed from best to worst binder. **f**, In vitro TR-FRET complex formation assay for compounds shown in (**e**). **g**, Chemical structures of several compounds derived from simple modifications of the simplified CR8-like scaffold DS11 listed from best to worst binder. **h**, In vitro TR-FRET complex formation assay for compounds shown in (**g**). **i**, Crystal structure of the ternary complex formed with DS30 (chemical structure in (**g**)) (left) and overlay of the binding mode of DS11 and DS30 (right). (**a-c**, **f, h**) Individual replicates are shown (n = 17 for CR8; n = 4 for DS08, DS30, DS66, roscovitine; n = 2 for others). (**d, i**) Interactions are represented by dashed lines. Hinge hydrogen bonds are shown in pink, aromatic H-bonds in grey, other hydrogen bonds in yellow, and π-cation interactions in green. Source data

We then probed the interactions between the DDB1 Arg928 and the solvent-exposed arene moiety of the ligand and noted that compounds featuring aliphatic chains or rings as their gluing moieties did not support high-affinity complex formation (Extended Data Fig. 1f–h, **7**–**15**). Further modifications of an otherwise robust recruiter DS11, including extension, shortening or rigidification of the alkyl chain, or introduction of larger π-systems all negatively impacted activity, underscoring the importance of correct arene positioning and identifying steric constraints for bulky compounds that lack conformational plasticity (Fig. 1d, Extended Data Figs. 1i and 2a,b, **16**–**24**; see also Supplementary Note for a more detailed discussion of the underlying SAR). Moreover, while exchanging the phenyl rings in the R_1_ of CR8 for heterocycles or substituted arenes (for example, DS25 (**25**) and DS43 (**26**)) only led to small changes in affinity, the phenyl group of DS11 was strongly preferred over both electron-rich and electron-poor heterocycles (Extended Data Fig. 2c–f; **27**–**30**). Simple derivatization of DS11, such as dimethylation (DS66 (**31**); EC_50_ = 18 ± 1 nM) increased in vitro activity to CR8 levels (Extended Data Fig. 2g–i, **32**–**37**). Taken together, these results identify interactions between Arg928 and diverse aromatic groups in the ligand’s gluing moiety as key mediators of DDB1–CDK12 molecular glue activity.

**Extended Data Fig. 3:**
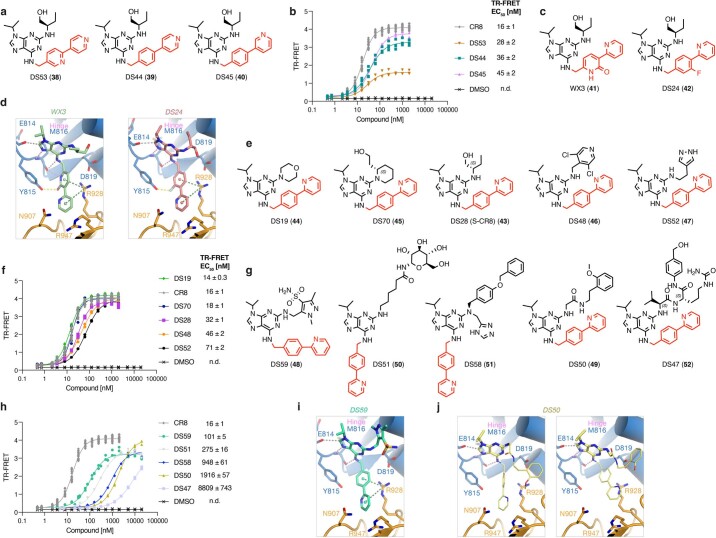
Other modifications of R_1_ and R_2_ groups. **a**, Chemical structures of CR8 derivatives where the location of the phenylpyridine nitrogen is varied, listed from best to worst binder. **b**, In vitro TR-FRET complex formation assay for compounds shown in (**a**), DMSO, and CR8. **c**, Chemical structures of CR8 derivatives. TR-FRET curves can be found in Extended Data Fig. 2c. **d**, Structures of interfaces induced by WX3 and DS24. **e**, Chemical structures of derivatives with various R_2_ substituents, for example inspired by SR-4835 (DS19), dinaciclib (DS70) or 21195 (DS48). **f**, In vitro TR-FRET complex formation assay for compounds shown in (**e**). **g**, Chemical structures of compounds obtained through R-group docking at the R_2_ position, displayed from best to worst binder. **h**, In vitro TR-FRET complex formation assay for compounds shown in (**g**). **i**, Crystal structure of the ternary complex formed with DS59. Interactions are represented by dashed lines. Hinge hydrogen bonds are shown in pink, aromatic H-bonds in grey, and π-cation interactions in green. Possible π-cation interactions between the diazole and proximal CDK12 lysine residues (K756, K861) were omitted for clarity**. j**, Crystal structure of the ternary complex formed with DS50. The R_1_ and R_2_ substituents could not be unambiguously fit into the density and were set to zero occupancy and are displayed with a smaller stick radius. Two probable conformations are shown. (**b, f, h**) Individual replicates are shown (n = 17 for CR8; n = 4 for DS28, DS44, DS53, DS59; n = 2 for others). Source data

Next, we focused on putative hydrogen bonding interactions of the gluing moiety. The pyridine nitrogen in the gluing moiety is a potential HBA (Extended Data Fig. 3a,b and Supplementary Note, **38**–**40**). We found that changing from a phenylpyridine to biphenyl (CR8 to DS16) (Fig. 1c and Extended Data Fig. 1e) or methylpyridine to tolyl (DS69 (**35**) to DS30 (**34**)) (Extended Data Fig. 2g,h) had no effect on in vitro activity, demonstrating that the HBA is dispensable. Our structural evaluation further highlighted the CDK12 residue Tyr815 as a potential additional hydrogen-bonding contact in the vicinity of the compound. We hence designed WX3 (**41**) bearing a 2-pyridinone ring instead of the first phenyl ring of CR8 (Extended Data Fig. 3c). Despite crystallographic analysis revealing the expected binding mode, the binding affinity did not improve (EC_50_ = 21 ± 1 nM) (Extended Data Figs. 2c and 3d). Other modifications, such as a fluorine at this position (DS24, (**42**)), led to decreased affinity (Extended Data Figs. 2c and 3c,d and Supplementary Note).

In summary, exploration of CR8 SAR around the R_1_ position demonstrated that surprisingly diverse gluing moieties (arenes and heteroarenes of varying size, but also aliphatic groups—albeit weakly) can engage the Arg928 side chain and therefore facilitate DDB1–CDK12 interactions. We show that, while an HBA is dispensable, appropriate steric and electronic properties for effective π–cation interactions with DDB1 are required for high-affinity complex formation. The position of DDB1 Arg928 remains relatively static in all structures, probably due to its anchoring interaction with CDK12 Asp819 (Fig. 1e). We conclude that diverse substituents can be accommodated in this *~*370 Å^3^ interfacial cavity in disparate ways, with each proficient molecular glue compound engaging DDB1 Arg928.

### Diverse R_2_ modifications are tolerated for complex formation

Next, we explored modifications of other positions on the CR8 scaffold. The R_3_ group faces into the kinase pocket and would primarily be expected to govern kinase binding. Instead, we focused on the aminobutanol moiety at the R_2_ position, which in the CR8-induced complex is largely solvent-exposed. The inversion at this stereocenter (S-CR8; DS28 (**43**); EC_50_ = 32 ± 1 nM) or introducing a morpholino at the R_2_ position (DS19 (**44**); EC_50_ = 14 ± 0.3 nM) gave rise to compounds with comparable activity to CR8 (R-CR8 is referred to as CR8 throughout) (Extended Data Fig. 3e,f), suggesting that hydrogen-bonding interactions between R_2_ and the CDK12 backbone do not strongly contribute to binding. Installation of a hydroxyethyl piperidine functionality, present in the potent CDK inhibitor dinaciclib^22^, (DS70, (**45**)) also yielded a potent molecular glue (EC_50_ = 18 ± 1 nM) (Extended Data Fig. 3e,f). On the other hand, substituting with a dichloropyridine (DS48, (**46**)) or pyrazole (DS52, (**47**)) led to a few-fold reduction in affinity (Extended Data Fig. 3e,f). In pursuit of more structurally diverse compounds, we performed R-group docking at this position using Glide (Schrödinger) and evaluated several compounds (DS59 (**48**) and DS50 (**49**)) stipulated to engage in additional interactions, yet their activity was modest (Extended Data Fig. 3g–j, **50**–**52**).

Hence, while changes at the R_1_ position are the most consequential for gain-of-function glue activity, derivatization at R_2_ further affects complex formation, and both R_1_ and R_2_ can tune important properties of the compound. Substantial changes in the compound structure, size and geometry of the key interaction with DDB1 Arg928 can be accommodated with surprisingly small penalties in glue-induced binding affinity.

### CDK12-selective inhibitors can be cyclin K degraders

**Fig. 2:**
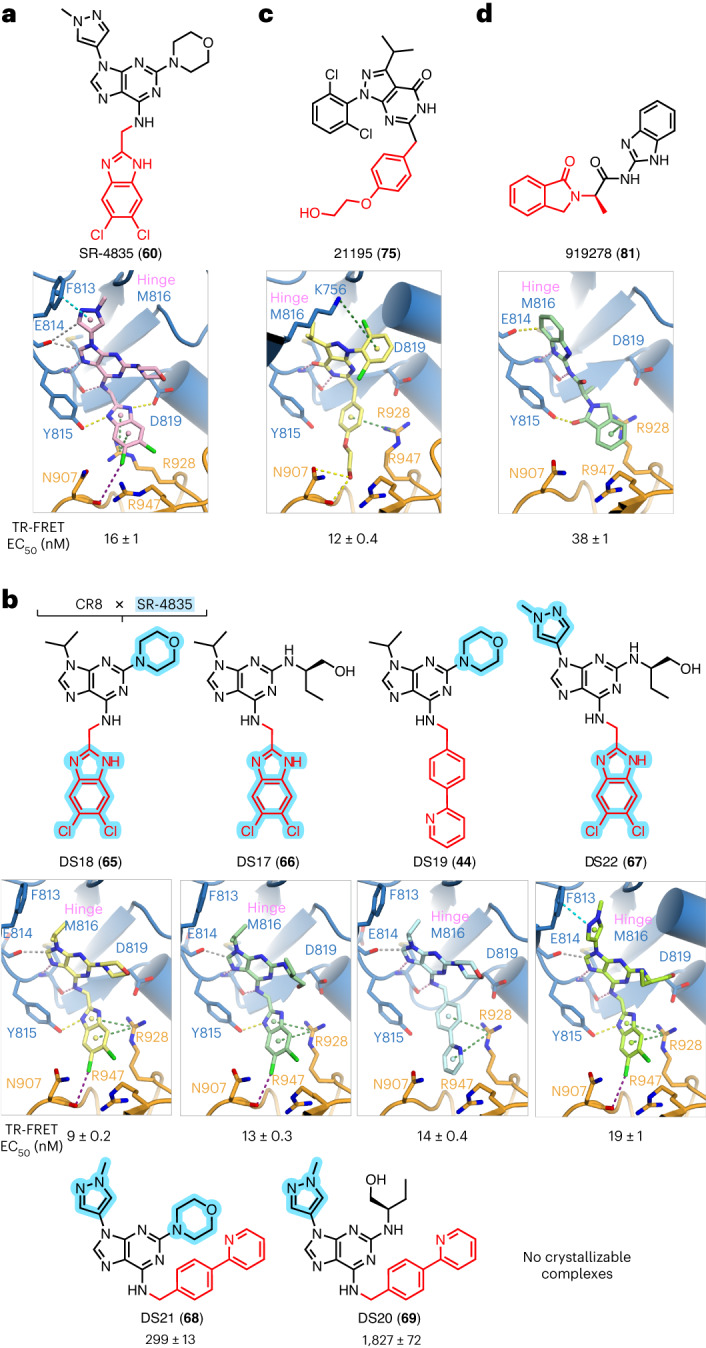
Published CDK inhibitors have cryptic molecular glue activity. **a**, Chemical structure of SR-4835 and ternary complex crystal structure of the DDB1–CDK12 interface induced by the compound. **b**, Chemical structures of CR8/SR-4835 hybrid compounds and ternary complex crystal structures of the DDB1–CDK12 interfaces induced by DS17, DS18, DS19 and DS22. **c**, Chemical structure of 21195 and ternary complex crystal structure of the DDB1–CDK12 interface induced by the compound. **d**, Chemical structure of 919278 and ternary complex crystal structure of the DDB1–CDK12 interface induced by the compound. In **a**–**d**, interactions are represented by dashed lines. Hinge hydrogen bonds are shown in pink, other hydrogen bonds in yellow, aromatic H-bonds in gray, π–cation interactions in green, π–π interactions in cyan and halogen bonds in purple. Density maps, omit maps and interaction distances can be found in Supplementary Figs. 1–3.

**Extended Data Fig. 4:**
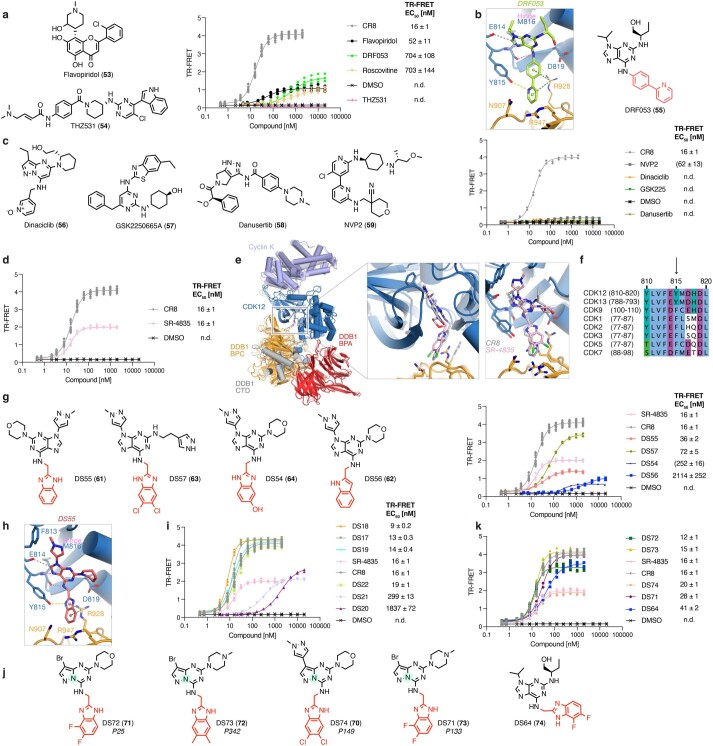
Evaluation of known compounds and their derivatives for cryptic molecular glue activity. **a**, In vitro TR-FRET complex formation assay for various CDK12 inhibitors. **b**, Crystal structure of the ternary complex formed with DRF053 and the chemical structure of DRF053. **c**, In vitro TR-FRET complex formation assay with other CDK inhibitors. **d**, In vitro TR-FRET complex formation assay for SR-4835 (structure in Fig. 2a). **e**, SR-4835 induces conformational changes in the N-lobe of CDK12 as compared to CR8. The CR8-bound structure is shown with 50% transparency. **f**, Multiple sequence alignment of human CDKs highlighting a unique tyrosine residue in CDK12/13 (CDK12 Tyr815). **g**, Chemical structures of SR-4835 derivatives, displayed from best to worst binder (left). In vitro TR-FRET complex formation assay for these compounds (right). **h**, Crystal structure of the ternary complex formed with DS55. **i**, In vitro TR-FRET complex formation assay for DS17-22 (structures in Fig. 2d). **j**, Chemical structures of compounds disclosed in patent WO2021116178^27^ and derivative DS64 bearing a related gluing moiety, listed from best to worst binder. Purine scaffold hopping is emphasized with a green highlight. **k**, In vitro TR-FRET complex formation assay for compounds shown in (**j**). (**a**, **c**, **d**, **g**, **i**, **k**) Individual replicates are shown (n = 17 for CR8; n = 4 for SR-4835, DS17, DS18, DS72, DS74; n = 2 for others). (**b**, **h**) Regions with no unambiguous F_**o**_-F_**c**_ density at 1σ are displayed with a smaller stick radius. Source data

Having established that gluing activity is common among CR8 derivatives, we next aimed to explore whether purine-based compounds with more diverse substituents also show gain-of-function molecular glue activity. We turned to published kinase inhibitors (Fig. 2 and Extended Data Fig. 4a–c, **53**–**59**), and first focused on the CDK12-selective inhibitor SR-4835 (refs. ^18,23^) (**60**), a compound with a CR8-like 2,6,9-trisubstituted purine structure but different R_1–3_ substituents. SR-4835 potently recruited DDB1 to CDK12–cyclin K (EC_50_ = 16 ± 1 nM) (Fig. 2a and Extended Data Fig. 4d), consistent with a recent report that it degrades cyclin K (ref. ^10^). Crystallization of the ternary complex revealed that SR-4835 binds in a manner similar to CR8, yet it induces conformational changes in the N-lobe of the kinase, most notably in the a.a. 731–743 loop (Extended Data Fig. 4e) (root mean square deviation (RMSD) of 1.6 Å). The imidazole ring of the gluing moiety interacts with DDB1 Arg928 and is additionally positioned by hydrogen-bonding interactions with Tyr815 and Asp819 of CDK12 (Fig. 2a). Tyr815 is specific to CDK12/13, with a phenylalanine present at this position in other CDKs (Extended Data Fig. 4f), which explains the high inhibitory selectivity of SR-4835. We also observed additional CDK12–ligand contacts, with π–π interactions between methylpyrazole and the kinase gatekeeper residue Phe813, a putative halogen bond to DDB1 Asn907, and two C_Ar_-H···O interactions with the backbone carbonyl of Glu814 (Fig. 2a).

To further explore the contributions of SR-4835 structural features to its activity, we evaluated various derivatives (Extended Data Fig. 4g–k). At R_1_, an unsubstituted benzimidazole gluing moiety (DS55, (**61**)) showed a similar binding mode and only slightly impaired recruitment (EC_50_ = 36 ± 2 nM), while exchanging the benzimidazole for an indole (DS56, (**62**)) largely abolished activity (EC_50_ = 2114 ± 252 nM), highlighting the importance of the two hydrogen-bonding interactions provided by the imidazole moiety of SR-4835 (Extended Data Fig. 4g,h, **63** and **64**). We also synthesized six compounds that are hybrids of SR-4835 and CR8 (DS18 (**65**), DS17 (**66**), DS19, DS22 (**67**), DS21 (**68**) and DS20 (**69**)), yielding a series bearing all possible combination of the R_1-3_ substituents (Fig. 2b). While DS17–19, DS22, CR8 and SR-4835 promoted very robust in vitro complex formation and yielded crystallizable complexes, compounds DS21 and DS20, carrying the phenylpyridine gluing moiety of CR8 and the SR-4835-derived methylpyrazole in R_3_, were poorer glues (Fig. 2b and Extended Data Fig. 4i). We conclude that these two bulky substituents cannot be accommodated together, while all other combinations permit effective complex formation, with DS17–19 being among the most potent in vitro recruiters in our series and superior to CR8 (Fig. 2b and Extended Data Fig. 4i).

Changing the position of one nitrogen atom in the purine ring system was previously reported to increase potency of CR8 (refs. ^20,24–26^). We therefore evaluated the effect of such scaffold hopping in the case of SR-4835, while keeping the R_1-3_ substituents unchanged (DS74, (**70**)). The resulting compound, however, performed comparably to SR-4835 in TR-FRET (EC_50_ = 20 ± 1 nM) (Extended Data Fig. 4j,k). Multiple structurally related compounds were disclosed in a recent patent^27^, and we selected three of these (DS72 (P25, **71**), DS73 (P342, **72**) and DS71 (P133, **73**)) on the basis of their reported potency and similarity to other small molecules in our series (Extended Data Fig. 4j). TR-FRET measurements revealed these molecules facilitate complex formation in vitro with affinities comparable to CR8 and SR-4835 (12 nM < EC_50_ < 28 nM), with DS72 performing the best (Extended Data Fig. 4k). As compounds DS71 and DS72 feature a difluorobenzimidazole gluing moiety previously reported to enhance CDK12 binding^28^, we evaluated substituting the CR8 phenylpyridine for this moiety (DS64, **74**), but found it decreased activity (EC_50_ = 41 ± 2 nM) (Extended Data Fig. 4j,k). Thus, purine scaffold hopping does not independently enhance gluing activity but can yield highly potent glues (DS71–73) in combination with appropriate R_1_–R_3_ substituents.

### Hydrophilic moieties can engage DDB1 to degrade cyclin K

**Extended Data Fig. 5:**
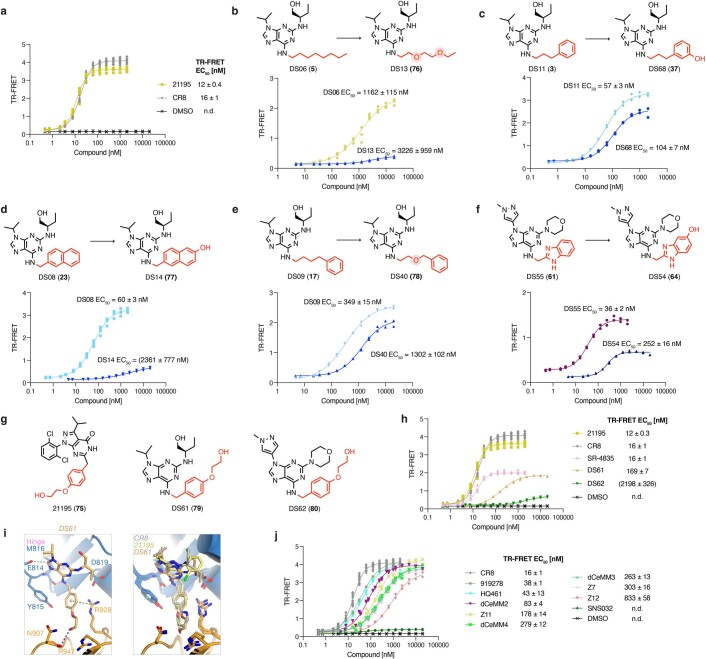
Hydrophilic and small compounds highlight cyclin K degrader diversity. **a**, In vitro TR-FRET complex formation assay for 21195 (structure in Fig. 2c). **(b-f)** Examples of the impact of increasing the hydrophilicity of the gluing moiety on multiple scaffolds including the relevant chemical structures and the associated in vitro TR-FRET compound titration results. **b**, DS06 and DS13. **c**, DS11 and DS68. **d**, DS08 and DS14. **e**, DS09 and DS40. **f**, DS55 and DS54. **g**, Chemical structures of 21195 and compounds resulting from hybridization of CR8 and SR-4835 with this inhibitor, listed from best to worst binder. **h**, In vitro TR-FRET complex formation assay for compounds shown in (**f**). **i**, Crystal structure of the ternary complex formed with DS61 (left) and the overlay of complexes induced by DS61, 21195, and CR8 (right). Interactions are represented by dashed lines. Hinge hydrogen bonds are shown in pink, other hydrogen bonds in yellow, aromatic H-bonds in grey, and π-cation interactions in green. **j**, In vitro TR-FRET complex formation assay for compounds shown in Fig. 2d and Fig. 3a-e. (**a-f**, **h**, **j**) Individual replicates are shown (n = 17 for CR8; n = 4 for DS06, DS08, 21195, SR-4835, dCeMM2, dCeMM4, Z7, Z11, HQ461; n = 2 for other compounds). Source data

To identify more diverse DDB1–CDK12 molecular glues, we performed virtual screening of the ZINC kinase inhibitor library against the DDB1–CDK12 interface^29^. We reasoned that kinase binders constitute a promising pool of putative cyclin K degraders, potentially with diverse exit vectors for DDB1 engagement. One prominent hit was the CRK inhibitor 21195 (RGB-286147, (**75**)) (ref. ^30^) (Fig. 2c). Crystallographic analysis showed that, despite a seemingly different chemical structure, 21195 displays a similar binding mode to CR8 and potently promotes ternary complex formation (EC_50_ = 12 ± 0.4 nM) (Fig. 2c and Extended Data Fig. 5a). The pyrazolopyrimidone core is rotated by 90° with respect to the CR8 purine to allow favorable hinge interactions, while the three substituents occupy equivalent positions to R_1-3_ of CR8 (Fig. 2c). Remarkably, the gluing moiety of 21195, while still bearing a benzene ring capable of the key π–cation interaction with Arg928, has an otherwise hydrophilic character, with a hydroxy group pointing directly towards DDB1 and hydrogen bonding with Asn907 (Fig. 2c).

To test more broadly whether hydrophilic moieties can recruit DDB1 we derivatized several degraders with hydroxy substituents or PEG groups, which led to effects ranging from a few-fold decrease to complete loss of activity across multiple R_1_ scaffolds (Extended Data Fig. 5b–f, **76**–**78**). We also synthesized additional hybrid compounds by transplanting the hydrophilic gluing moiety of 21195 onto CR8 (DS61 (**79**)) or SR-4835 (DS62 (**80**)) scaffolds (Extended Data Fig. 5g–i) and saw that, while DS62 was incompatible with the DDB1–CDK12 interface, DS61 displayed molecular glue activity (EC_50_ = 169 ± 7 nM) and its binding pose preserved the Asn907 interaction, albeit with a less optimal geometry (Extended Data Fig. 5g–i). These results demonstrate that kinase-engaging scaffolds with distinct exit vectors can act as efficient molecular glues, and that hydrophilicity of the gluing moiety does not preclude robust DDB1–CDK12 interactions despite expected desolvation penalties.

### Specific fingerprint defines minimal cyclin K degraders

Having observed that CDK12 binders beyond CR8 derivatives can facilitate an analogous CDK12–DDB1 complex, we investigated more structurally dissimilar CDK12 inhibitors focusing on compound 919278 (**81**) (Fig. 2d). This inhibitor shows weak CDK12 binding affinity (5.6 µM) but pronounced downstream transcriptional effects^31^, which prompted us to hypothesize it may have gain-of-function activity. Strikingly, we found that despite its low molecular weight and lack of chemical similarity to CR8, SR-4835 or 21195, compound 919278 potently recruits DDB1 to CDK12–cyclin K in vitro (EC_50_ = 38 ± 1 nM) (Fig. 2d and Extended Data Fig. 5j). Structural characterization demonstrated that notwithstanding its smaller size, the compound satisfies the key hinge contacts, shows a C_Ar_-H···O interaction with Glu814 akin to that of CR8, and interacts with CDK12 Tyr815 through hydrogen bonding (Fig. 2d). Crucially, its isoindolinone gluing moiety protrudes out into the interface engaging DDB1 Arg928 via π–cation interactions as seen for previous scaffolds (Fig. 2d). Hence, the diversity observed among DDB1–CDK12 glues extends beyond typical purine-based kinase inhibitor scaffolds.

**Fig. 3:**
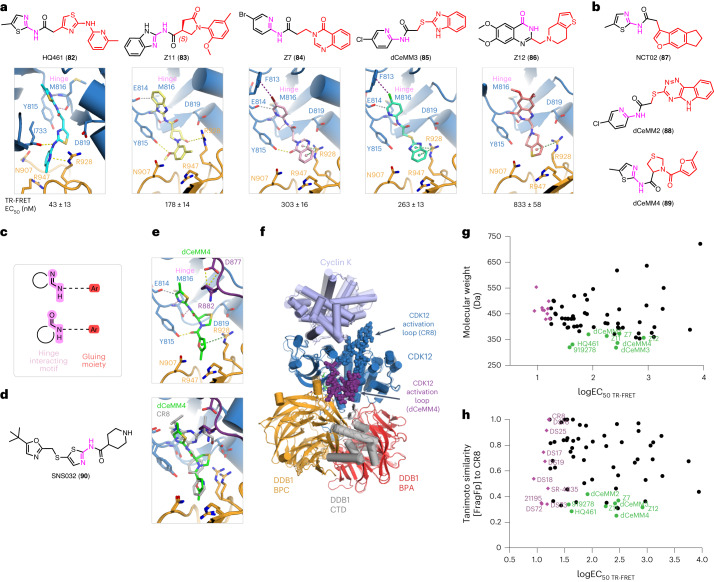
Low-molecular-weight cyclin K glues. **a**, Chemical structures of HQ461, Z11, Z7, dCeMM3 and Z12 and ternary complex crystal structures of the DDB1–CDK12 interfaces induced by each compound. HQ461 could in theory bind at the interface in two directions (see fingerprint in **c**), yet the density, while somewhat ambiguous, suggests the methylpyridine moiety points towards DDB1. **b**, Chemical structures of NCT02, dCeMM2 and dCeMM4, which are cyclin K degraders. **c**, The fingerprint of a cyclin K degrader. **d**, Chemical structure of SNS032, which is not a cyclin K degrader despite binding CDK12. **e**, Ternary complex crystal structure with dCeMM4 (top) and overlay of dCeMM4 and CR8 (bottom). **f**, DDB1–CDK12–cyclin K complex architecture, with a conformational change in a CDK12 activation loop (spheres) induced by dCeMM4. While a closed-loop kinase conformation is often associated with an inactive kinase state whereby the Asp–Phe–Gly (DFG) motif flips, here no DFG flip was observed. **g**, Diversity of cyclin K degraders illustrated through a plot of the compounds’ molecular weight and their ternary complex formation affinity. The ten compounds most active in vitro are shown in purple and the low-molecular-weight cyclin K degraders described above are colored green. **h**, As in **g** but showing the compounds’ Tanimoto similarity to CR8 and their ternary complex formation affinity. **a**,**e**, Interactions are represented by dashed lines. Hinge hydrogen bonds are shown in pink, other hydrogen bonds in yellow, aromatic H-bonds in gray, π–cation interactions in green and halogen bonds in purple. Regions with no unambiguous *F*_o_–*F*_c_ density at 1*σ* are displayed with a smaller stick radius. Density maps, omit maps and interaction distances can be found in Supplementary Figs. 1–3. Source data

Interestingly, multiple other, small cyclin K degraders have recently been published^8,10,11^. Structural evaluation of HQ461 (**82**), Z11 (**83**), Z7 (**84**), dCeMM3 (**85**) and Z12 (**86**) (Fig. 3 and Extended Data Fig. 5j) revealed that all promote the formation of an analogous ternary complex, and sustain the hinge hydrogen bonds and the π–cation interaction with DDB1 Arg928 (suboptimal for Z11 and dCeMM3; for HQ461 and Z11 we observe hydrogen bonding with the side chain instead), while also displaying interactions with additional residues (Fig. 3a). Those include CDK12 Tyr815 (hydrogen bonds for Z7 and Z11), Glu814 (C_Ar_-H···O interaction for Z7, Z11 and dCeMM3), Phe813 (halogen bonds for Z7 and dCeMM3) and Ile733 (hydrogen bond for HQ461) (Fig. 3a).

DDB1–CDK12 complex formation and cyclin K degradation can thus be achieved with small, almost fragment-like compounds. Furthermore, our glue-bound DDB1–CDK12 structures, together with assessment of other reported glues (NCT02 (**87**), dCeMM2 (**88**) and dCeMM4 (**89**)), identify a minimal fingerprint for a cyclin K degrader leveraging a hinge-interacting acceptor–donor motif common to kinase inhibitors, and a gluing moiety bearing an aromatic system extending from the hydrogen bond donor (Fig. 3b,c). The structurally related inhibitor SNS032 (**90**) serves as a negative example, where a non-aromatic piperidine is expected to point towards DDB1 in the described CDK binding mode^32^, consistent with SNS032 displaying no gain-of-function activity (Fig. 3d).

### Rearrangement triggered by dCeMM4 illustrates plasticity

While all other compounds yielded highly similar ternary complexes (maximum RMSD 1.7 Å), the dCeMM4-induced complex was distinctive (RMSD 3.1 Å). The compound is positioned at the DDB1–CDK12 interface, with π–cation interaction of the furan ring with DDB1 Arg928, hydrogen bonding of the carbonyl with CDK12 Tyr815 and C_Ar_-H···O interaction with Glu814 (Fig. 3e). However, further inspection of the compound interface revealed that Arg882 of CDK12 is brought into the vicinity of the pocket and stabilized in position by interactions with Asp877. Such a conformational change is not feasible with other compounds due to steric hindrance, for example, with the aminobutanol moiety of CR8 (Fig. 3e). Remarkably, this local change leads to a rearrangement of the kinase activation loop (a.a. 876–898) yielding a closed-loop CDK12 conformation, apparent in one of the three molecules in the asymmetric unit (chain H, but not B, E) (Fig. 3f). While evidently not strictly required for compound binding, the observed conformation illustrates the inherent plasticity along the extensive DDB1–CDK12 interface.

### Relationships between in vitro and cellular activity

**Fig. 4:**
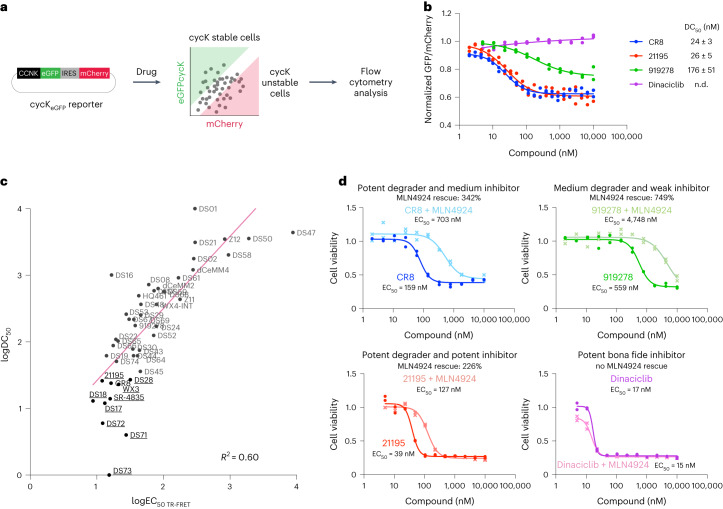
Cellular evaluation of cyclin K molecular glue degraders. **a**, Schematic of the cyclin K dual-color reporter assay. **b**, HEK293T cycK_eGFP_ reporter assay results for four example compounds. Individual replicates are shown (*n* = 2); n.d., not determined. Data for all compounds can be found in Supplementary Fig. 4. **c**, Correlation of in vitro complex formation affinity (logEC_50_ TR-FRET) and cycK_eGFP_ reporter results (logDC_50_) modeled with a linear regression (*R*^2^ = 0.60). The in vitro TR-FRET EC_50_ values for the best compounds (bold) are overestimated, which negatively impacts the correlation. The equivalent plot in linear scale is shown in Extended Data Fig. 6a. **d**, Viability assay in HEK293T cells for four example compounds, with curves corresponding to treatment with the individual drug or additional pretreatment with 100 nM of the neddylation inhibitor MLN4924. Individual replicates are shown (*n* = 2). Data for all compounds can be found in Supplementary Fig. 5. Source data

**Extended Data Fig. 6:**
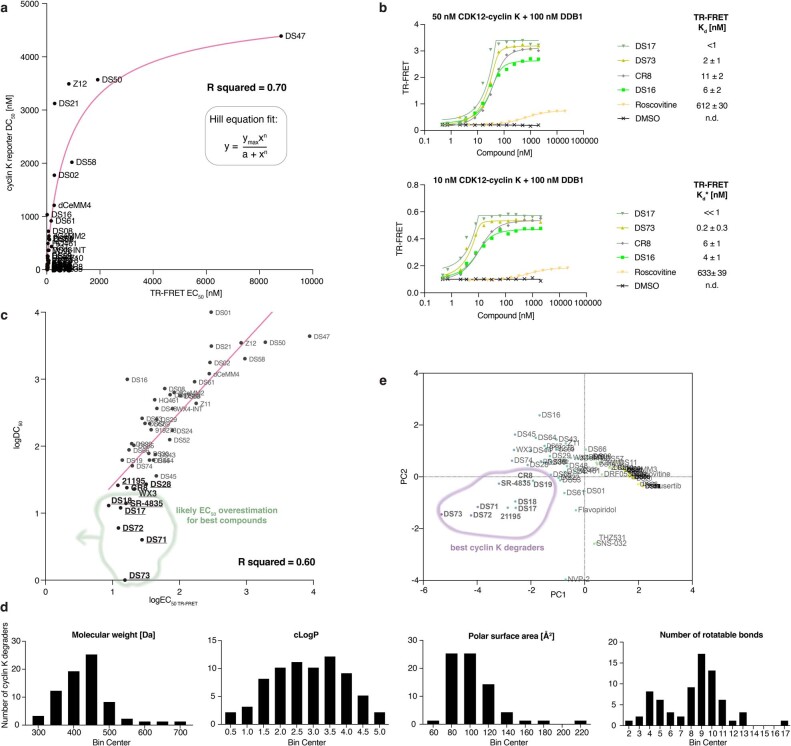
Further characterisation of molecular glue degrader compounds. **a**, Correlation of in vitro assay data (TR-FRET EC_50_ [nM]) with cellular cyclin K_eGFP_ reporter assay results (DC_50_ [nM]), based only on compounds with unambiguous activity in the reporter assay (n = 42). The relationship can be described with a Hill equation (R squared = 0.70), with the equation used solely to describe the mathematical relationship between the two experimental datasets and not for its biological meaning. **b**, In vitro TR-FRET complex formation assay performed as a compound titration with 50 nM CDK12-cyclin K (left) or 10 nM CDK12-cyclin K (right) for several compounds (n = 1). Data were fitted with a quadratic equation appropriate for a case where the expected K_d_ value is lower than the protein concentration used^69^. Lowering the protein concentration resulted in a much smaller assay window but yielded K_d_* values in the sub-nanomolar range for the top compounds (DS17, DS73), while showing no difference for the weak recruiter roscovitine, indicating that the tightest glues lie below the limit of detection of the TR-FRET assay (this is also highlighted by the spurious fit observed for DS17). **c**, Correlation of in vitro complex formation affinity (logEC_50_ TR-FRET) and cycK_eGFP_ reporter results (logDC_50_) modelled with a linear regression (R squared = 0.60). The in vitro TR-FRET values for the best compounds (bold) are overestimated and the green cloud indicates how the data points would be predicted to shift upon accurate affinity quantification, further improving the observed correlation (*c.f*. panel **b**). **d**, Histograms displaying the molecular weight, clogP, polar surface area, and the number or rotatable bonds distributions across the cyclin K degrader compound set. **e**, PCA of the combined multi-assay data for cyclin K degraders. Source data

A fundamental question in the molecular glue degrader field has been the relationship between ternary complex formation and cellular degradation. On the basis of previous examples, a step function-like relationship was postulated, whereby a few-fold difference in in vitro affinity toggles target degradation in cells^15,33^. To investigate this further, we evaluated our set of cyclin K degraders using a dual-color cyclin K–enhanced green fluorescent protein (eGFP) stability reporter in HEK293T cells as previously described^15^ (Fig. 4a, Supplementary Table 2 and Supplementary Fig. 4). We were able to distinguish potent degraders (for example, CR8 and 21195) from weaker (for example, 919278) or non-degrading (for example, dinaciclib) compounds (Fig. 4b). The ten best-performing degraders were DS73, DS71, DS72, DS17, DS18, SR-4835, WX3, CR8, 21195 and DS28, a list that overlaps remarkably well (8/10) with our in vitro data. We then examined the correlation of cyclin K reporter degradation (DC_50_) and in vitro ternary affinity (EC_50(TR-FRET)_) across the entire compound set, including only compounds with unambiguous cellular degradation activity (*n* = 42) (Fig. 4c). A linear correlation between logDC_50_ and logEC_50 (TR-FRET)_ (*R*^2^ = 0.6) (Fig. 4c) was observed, whereas non-log data could be described with a Hill-type equation (*R*^2^ = 0.7) (Extended Data Fig. 6a–c).

The overall EC_50(TR-FRET)_–DC_50_ correlation (Fig. 4c and Extended Data Fig. 6a,b) finds in vitro complex formation largely predictive of in-cell degradation. The sigmoidal relationship further illustrates the physical relationship by which small differences in in vitro affinity translate to larger discrepancies in cellular degradation^33^. While TR-FRET allows us to establish a rank order of in vitro activities, affinities tighter than 50 nM are below the dynamic range of our assay, probably leading to EC_50_ overestimation for the best compounds. Performing the assay at lower protein concentrations for selected compounds revealed subnanomolar dissociation constants for the top derivatives (for example, DS17) but presented experimental challenges with a limited assay window (Extended Data Fig. 6b and Methods). While the correlation between binding and degradation is apparent, effective quantification of these subnanomolar affinities is expected to further improve the description of this relationship (Extended Data Fig. 6b,c).

### Cyclin K degrader diversity

To gauge the extent of chemical diversity among our compound set, we explored the distribution of various physicochemical properties and we found that cyclin K degraders that display in vitro activity span molecular weights from 317 Da to 722 Da and clog*P* values between 0.4 and 5.1 (Extended Data Fig. 6d). While the compounds are highly diverse, most are conventionally drug-like, as expected for kinase inhibitor-derived or almost fragment-like compounds (Fig. 3g). We then computed the Tanimoto coefficients describing the similarity to CR8 for each compound. Notably, the compound’s Tanimoto similarity to CR8 and its in vitro activity show no clear correlation, with the ten best DDB1–CDK12 molecular glues (DS18, 21195, DS72, DS17, DS19, DS25, DS73, SR-4835, CR8 and DS16) ranging from CR8-like (1.0) to distinct (0.33) (Fig. 3h), further supporting that diverse chemistries facilitate the formation of this complex.

We next investigated the differences in cellular activity among these diverse compounds by probing the ubiquitin ligase-dependent cytotoxicity of the degraders. For this, we measured the viability of HEK293T cells after 72 h of compound exposure with and without pretreatment with the neddylation inhibitor MLN4924 (Fig. 4d, Supplementary Table 2 and Supplementary Fig. 5). While for some compounds cyclin K degradation appears to be the dominant contribution to cytotoxicity (for example, 919278), others (such as 21195) show less pronounced rescue and hence work partly by conventional kinase inhibition or other, potentially off-target mechanisms (Fig. 4d). We combined the results from multiple assays, logDC_50_, logEC_50(TR-FRET)_, logEC_50(cell viability)_ and a descriptor of cytotoxicity rescue upon MLN4924 co-treatment (%_MLNrescue_), as inputs for principal component analysis (PCA). Distinct clusters of cyclin K molecular glue degraders emerged, with the top degraders found in multiple subgroups (Extended Data Fig. 6e), illustrating that our experimental set features compounds with considerable diversity in physicochemical properties (Fig. 3g and Extended Data Fig. 6d) but also a range of cellular activities.

**Fig. 5:**
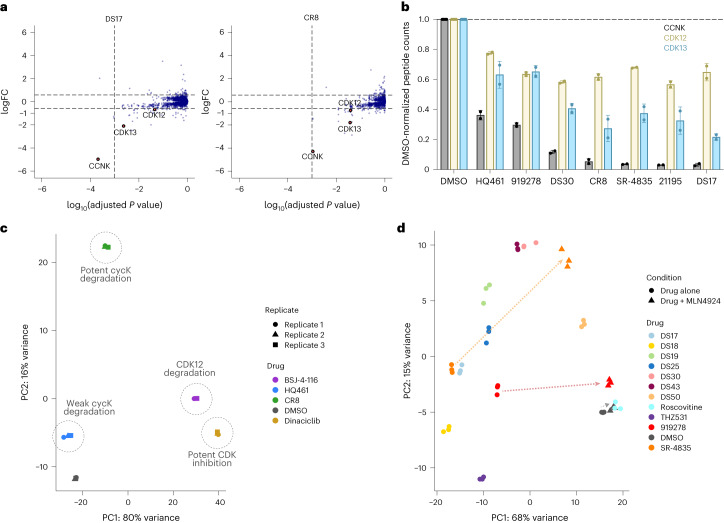
Diverse cyclin K molecular glue degraders give rise to unique cellular responses. **a**, MDA-MB-231 cells were exposed to 1 µM DS17, CR8 or DMSO for 5 h followed by whole proteome quantification using label-free mass spectrometry (mean log_2_ fold change, *P* value calculated by a moderated *t*-test, *n* = 4 (DMSO), *n* = 2 (DS17 and CR8)). **b**, Representation of the average peptide counts of cyclin K (dark gray), CDK12 (yellow) and CDK13 (blue). Data represent the mean ± standard deviation (*n* = 4 for DMSO and *n* = 2 for each compound treatment). The corresponding volcano plots can be found in Extended Data Fig. 7a. **c**, PCA of the RNA-seq data for CR8, BSJ-4-116, dinaciclib, HQ461 and DMSO (*n* = 3). **d**, PCA analysis of the RNA-seq data for a larger selection of compounds (*n* = 3). For 919278 and SR-4835, two conditions were assessed: compound alone (circle) or compound + MLN4924 (triangle). Co-treatment with the neddylation inhibitor resulted in large shifts of the resulting points (dashed arrows). In **c** and **d**, the corresponding volcano plots can be found in Extended Data Fig. 9. Source data

**Extended Data Fig. 7:**
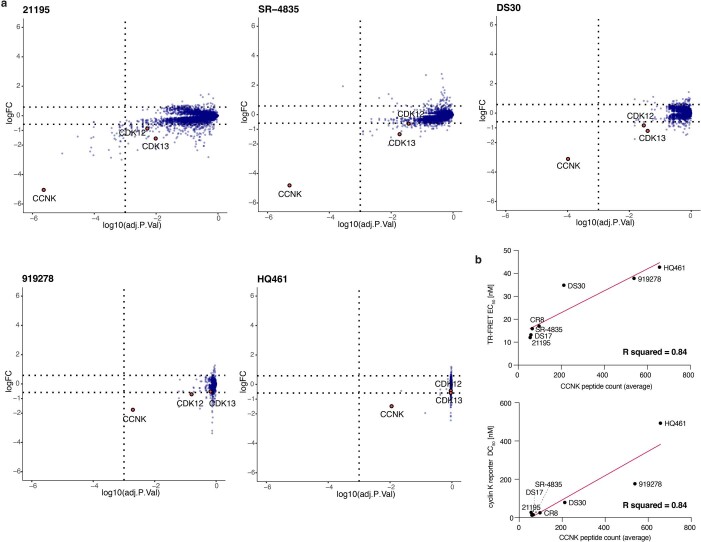
Proteomics reveal large differences in the extent of cyclin K depletion. **a**, Label-free proteomics. Mean log2 fold change, p value calculated by a moderated t-test; n = 4 (DMSO), n = 2 (21195, SR-4835, DS30, 919278, and HQ461). MDA-MB-231 cells were treated with 1 µM drug for 5 h. **b**, Correlation of the average cyclin K peptide count with the TR-FRET EC_50_ (left) or with the cyclin K_eGFP_ reporter DC_50_ (right). Source data

To better dissect the cellular responses, we examined the extent and depth of cellular CDK12, CDK13 and cyclin K degradation by mass spectrometry using quantitative label-free proteomics on MDA-MB-231 cells treated with representative compounds (Fig. 5a,b and Extended Data Fig. 7a). These experiments identified three of the six compounds tested (DS17, 21195 and SR-4835) to be more effective at depleting cyclin K than CR8 at 1 µM concentration (Fig. 5a,b). All compounds tested selectively degrade cyclin K and the extent of depletion correlates well with the cyclin K stability reporter and TR-FRET complex formation data (Extended Data Fig. 7b; *R*^2^ = 0.84). Interestingly, while CDK12 showed only a mild decrease of abundance with all compounds, CDK13 depletion was more pronounced and appeared to scale with that of cyclin K (Fig. 5b).

### Decoupling kinase inhibitory selectivity from glue activity

**Extended Data Fig. 8:**
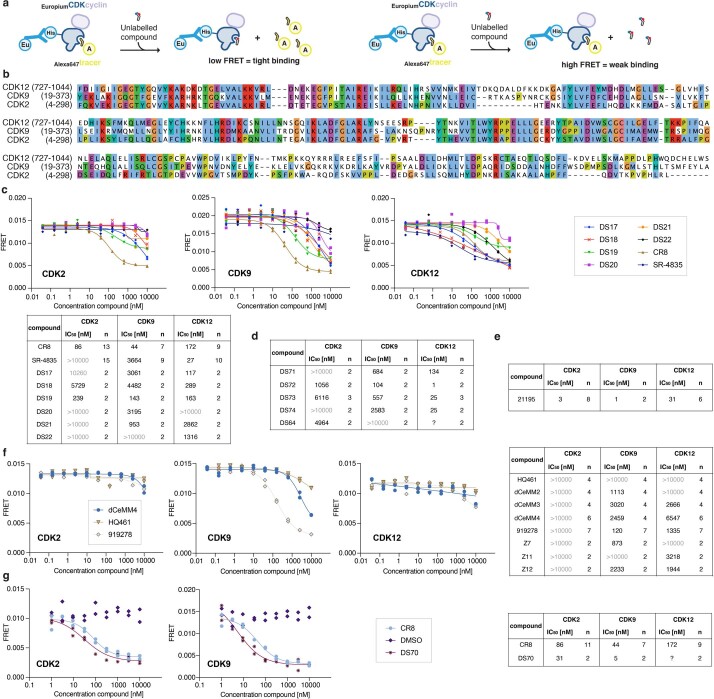
Cyclin K degraders do not require traditional kinase inhibitory properties for activity. **a**, Commercial in vitro Lanthascreen assay. The binding of an Alexa_647_-conjugated tracer to a kinase is detected by addition of an Eu-labelled anti-tag antibody. Binding of the tracer and antibody to a kinase yields a high TR-FRET signal, while tracer displacement with a kinase inhibitor results in signal loss. **b**, Multiple sequence alignment of CDK12, CDK9, and CDK2, omitting the C-terminal extension of CDK12. **c**, Selectivity profiling of CR8/SR-4835 hybrid compounds across CDK2/9/12 using Lanthascreen. Representative TR-FRET curves (n = 2) (top) and a table listing average IC_50_ values and total number (n) of measurements (bottom). **d**, Selectivity profiling results for compounds derived from a recent patent and related derivatives. **e**, Selectivity profiling of compound 21195. **f**, Representative TR-FRET curves (n = 2) (left) and a table listing average IC_50_ values and total number (n) of measurements (right) for low molecular weight cyclin K degraders. **g**, Representative TR-FRET curves (n = 2) (left) and a table listing average IC_50_ values and total number (n) of measurements (right) for CR8 and DS70, a hybrid of CR8 and dinaciclib bearing the hydroxyethyl piperidine functionality present in this potent CDK inhibitor. DS70 shows more pronounced pan-CDK engagement than CR8. (**c-g**) Additional replicates and further data can be found in Supplementary Fig. 6. Source data

As CR8 has dual CDK inhibitor/cyclin K degrader activity, we examined the CDK inhibitory activity of our compound set using Lanthascreen (Extended Data Fig. 8a–f), which monitors fluorescent tracer displacement from the active site of a Europium-labeled kinase (Extended Data Fig. 8a). The assays were performed with CDK12, as well as with CDK9 and CDK2, representative transcriptional and cell-cycle CDKs similar in sequence to the kinase domain of CDK12 (Extended Data Fig. 8b).

SR-4835 is CDK12/13 selective while CR8 has been reported to inhibit CDK1/2/5/7/9/12 (ref. ^34^). To investigate how specificity is tuned by compound modifications, we first evaluated the CDK inhibitory activity of the CR8/SR-4835 hybrid compounds (DS17–22) (Fig. 2b and Extended Data Fig. 8c). SR-4835, DS17 and DS18 were CDK12 selective, while CR8 and DS19 were not, linking potent pleiotropic CDK inhibition to the co-existence of phenylpyridine and isopropyl substituents on the purine scaffold (Extended Data Fig. 8c). Hence, the modification of the gluing moiety from phenylpyridine to dichlorobenzimidazole (CR8 versus DS17, DS19 versus DS18) is sufficient to increase the gluing potency and reduce off-target CDK inhibitory effects of CR8 (Extended Data Fig. 8c).

Small cyclin K degraders such as Z11 or HQ461 show less optimal engagement of the ATP pocket than CR8 derivatives (Fig. 3a). Accordingly, while CR8 displayed CDK IC_50_ values of 40–200 nM, the smaller compounds show weaker CDK association (Extended Data Fig. 8f). None of those compounds appreciably bound CDK2, and while compound 919278 showed some inhibition of CDK9/12 (IC_50(CDK9)_ 120 nM, IC_50(CDK12)_ 1,335 nM), dCeMM2-4 and Z11-12 displayed only micromolar IC_50_ values and HQ461 displayed no inhibition up to 10 µM (Extended Data Fig. 8f and Supplementary Fig. 6). Hence, cyclin K degraders do not require pronounced traditional kinase inhibitory properties for molecular glue activity, and kinase inhibitory selectivity can be partly decoupled and tuned separately from ternary complex affinity optimization.

### Cyclin K degraders show distinct transcriptional signatures

**Extended Data Fig. 9:**
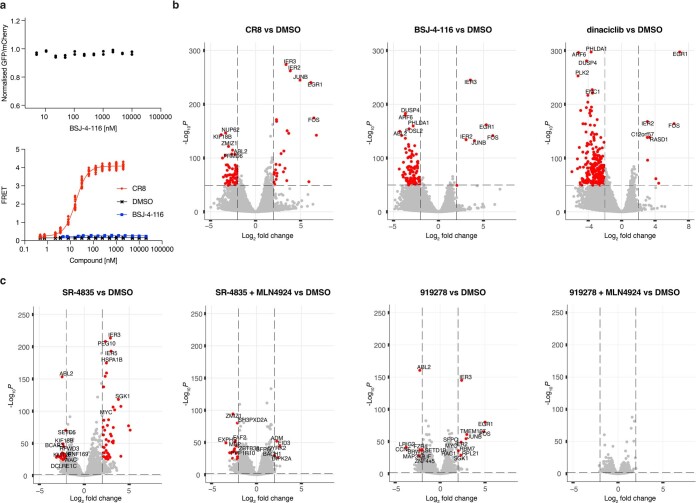
RNA sequencing reveals distinct transcriptional signatures for CDK12 inhibition, CDK12 degradation, and cyclin K degradation. **a**, Cyclin K reporter degradation assay (n = 2) (top). In vitro TR-FRET complex formation assay (bottom) showing no cyclin K molecular glue degrader activity for the CDK12 PROTAC BSJ-4-116 (n = 2 for BSJ-4-116, n = 17 for CR8). **b**, Volcano plots depicting the RNAseq results for CR8 (cyclin K degrader), BSJ-4-116 (CDK12 degrader), and dinaciclib (CDK inhibitor) (n = 3). **c**, Volcano plots showing the RNAseq results for 919278 and SR-4835, either alone or in combination with the neddylation inhibitor MLN4924 (n = 3). Source data

Finally, we asked whether cyclin K degraders effect a different transcriptional response than CDK12 degraders or CDK12 inhibitors. For this, we performed RNA sequencing (RNA-seq) in MDA-MB-231 cells, choosing triple-negative breast cancer cells as the relevant therapeutic context for CDK12/13–cyclin K inactivation^16,18^. While a CDK12 inhibitor (dinaciclib) and CDK12 degrader (PROTAC BSJ-4-116 (**91**) that does not degrade cyclin K (ref. ^35^; Extended Data Fig. 9a) clustered together following PCA analysis of RNA-seq data, both the potent (CR8) and the weak degrader (HQ461) clearly clustered separately (Fig. 5c and Extended Data Fig. 9b). Cyclin K degradation therefore results in a different transcriptional signature than degradation or inhibition of CDK12. Supporting this notion, messenger RNA sequencing of cells treated with a larger set of compounds followed by PCA revealed specific clusters relating to their mechanism of action, with the relative extent of cyclin K degradation (and the associated CDK12/13 depletion) versus sole CDK inhibition further differentiating the observed PCA clusters (Fig. 5d). Moreover, the analysis of related compounds (for example, CR8 versus DS17–19 and SR-4835) illustrates how subtle chemical modifications impacting both inhibitory selectivity and degrader potency further tune these signatures (Fig. 5d). We observed that co-treatment of cells with MLN4924 rescued the cyclin K degradation-related perturbation, resulting in a shift to a dimethyl sulfoxide (DMSO)-like transcriptional state for compounds with limited CDK12 inhibition (919278) or elsewhere for potent CDK12/13 inhibitors (SR-4835) (Fig. 5d and Extended Data Fig. 9c). Therefore, cyclin K degrader compounds range from multi-CDK inhibitors/cyclin K degraders (for example, CR8), to CDK12/13-selective inhibitors and cyclin K degraders (for example, SR-4835 and DS17), and degraders with little-to-no CDK inhibition (for example, HQ461), all of which differ in their cellular activity profiles, thereby offering distinct therapeutic opportunities.

## Discussion

In this study, we extensively surveyed the structure–activity relationship of cyclin K degraders and, in the process, identified multiple novel scaffolds that degrade cyclin K, including published kinase inhibitors. Over 90 chemically diverse compounds were evaluated, among which 40 were found to trigger CDK12–DDB1 complex formation with an affinity of <100 nM in vitro. Crystallographic dissection of 28 ternary complexes demonstrated that despite considerable diversity of the molecular glues (Fig. 3h and Extended Data Fig. 6d), the overall complex architecture is highly similar.

All the identified glues contact DDB1 Arg928, a residue we previously showed to be essential for complex formation^15^. Arginine residues are principally able to engage in polyvalent, low-selectivity ligand interactions and arene–arginine contacts have been shown to involve a mix of electrostatic and dispersion attractions^36–39^. Accordingly, we find that gluing moieties contact DDB1 preferentially through π–cation interactions (for example, DS08, Fig. 1d), which can support drug interactions over large distances (~6 Å) and variable geometries^39^. Some compounds instead contact Arg928 via hydrogen bonding (for example, HQ461 and Z11; Fig. 3a) and hydrophobic interactions (for example, DS06; Fig. 1c). The low-affinity DDB1–CDK12 interaction primes the complex, while the ligand, through filling the kinase pocket and bridging over to DDB1, drives robust association, offering a rationale for the unusually broad chemical diversity observed.

Other kinases beyond CDK12/13–cyclin K were not targeted in our studies (Fig. 5a and Extended Data Fig. 7a) and, notably, CDK13 degradation was more pronounced than that of CDK12 and appeared to scale with the loss of cyclin K (Fig. 5b). The specificity for the CDK12/13–DDB1 pairing therefore appears to be primed predominantly by the protein–protein interface (~2,100 Å^2^), featuring a CDK12/13-unique C-terminal tail^15^. This complementarity is underscored by a ~50 µM DDB1–CDK12–cyclin K binding affinity in the absence of compound^15^, yet no functional relevance of this interaction for endogenous kinase signaling is known^8,10,11,15^.

Nonetheless, additional compound-mediated interactions are required not for specificity but for sufficient stabilization of the ternary complex for cyclin K ubiquitination and degradation. Notably, the diversity of cyclin K degraders stems not only from the many distinct ways by which the CDK12–DDB1 interface can be bridged by gluing moieties, but also from the many possibilities of how the feature-rich ATP pocket of CDK12 can be engaged by small molecules. This in turn gives rise to chemically diverse compounds with a varying relative extent of inhibitory and gain-of-function characteristics, where the inhibitory versus degradative properties can be directly tuned and largely decoupled. This is most evident for smaller compounds (for example, HQ461), which provide starting points for developing de facto interface stabilizers with no inhibitory activity (Fig. 3a). The data presented herein reveal design principles for small molecules that robustly inactivate CDK12/13–cyclin K and have properties that differ from traditional kinase inhibitor scaffolds. We also present additional advantageous starting scaffolds for medicinal chemistry optimization of clinically relevant cyclin K degraders, including those with more potent cyclin K degradation and better kinase inhibitory selectivity profiles than CR8 (for example, DS17). As cyclin K degraders phenotypically differ from CDK12 degraders or inhibitors (Fig. 5c), the compounds discussed herein offer unique therapeutic opportunities for the therapeutic pursuit of CDK12/13–cyclin K as emerging targets in oncology^17,18^.

Our results yield principal learnings that can be applied to other molecular glue classes. We illustrate that small differences in ternary complex affinity translate to larger disparities in cellular degradation, which builds on an earlier concept of an affinity threshold (Fig. 4c and Extended Data Fig. 6a–c)^15,33^. The correlation between cellular degradation and in vitro complex formation observed among a large compound set suggests that biochemical ternary affinity is predictive of in-cell degradation. Our data also demonstrate that cellular degrader screening for cell viability ± MLN4924 (Fig. 4d) is feasible and informative for degraders and is akin to compound screening in wild-type versus hyponeddylated cellular models^8,15^.

More broadly, our results show that low-affinity interactions can be effectively strengthened by chemical matter. The more extensive and complementary the interface and the larger the interfacial cavity, the more solutions will exist to achieve gain-of-function glue activity with small molecules. Hence, low-affinity interfaces that encompass a defined ligand-binding pocket offer preferential starting points for molecular glue development. Furthermore, we propose that the absolute size of the protein–protein interface, as well as the compound’s relative contribution to the interface drives both the SAR and substrate specificity behavior. For thalidomide analogues and CRBN, the compound contributes *~*40% of the small interface area and therefore drives both specificity and affinity with tightly defined SAR^14,40,41^. In contrast, for DDB1–CDK12, the extensive protein–protein interface with *~*20% contributed by the compound allows for much greater variability in protein–ligand interactions, yielding a tolerant SAR but limiting the compound’s influence on neosubstrate specificity^14^.

## Methods

### Mammalian cell culture

The human HEK293T cell lines were provided by the Genetic Perturbation Platform, Broad Institute, MDA-MB-231 cells were purchased from ATCC and HEK293T_Cas9_ were previously published^33^. HEK293T cells were cultured in Dulbecco’s modified Eagle medium (Gibco) and MDA-MB-231 in RPMI (Gibco), with 10% fetal bovine serum (Invitrogen), glutamine (Invitrogen) and penicillin–streptomycin (Invitrogen) at 37 °C and 5% CO_2_.

### Compounds

The majority of compound bearing the ‘DS’ prefix were synthesized in-house (for synthetic chemistry methods, see Supplementary Note: Synthetic Procedures) on the basis of published synthetic procedures for related small molecules^42–44^. Compounds DS22, DS47, DS74, WX3 and WX4-INT were synthesized by WuXi AppTec. Other small molecules were obtained from the following commercial vendors: Tocris, Enzo Life Sciences, MedChemExpress, Cayman Chemical Company, ProbeChem, Sigma and LabNetwork, Enamine (Supplementary Table 3).

### Reporter vectors

The following reporters were used in this study: Cilantro 2 (PGK.BsmBICloneSite-10aaFlexibleLinker-eGFP.IRES.mCherry.cppt.EF1α.PuroR, Addgene #74450) for cyclin K degradation assay as previously reported^15^.

### Protein expression and purification

Human wild-type and mutant versions of DDB1 (Uniprot entry Q16531), CDK12 (Q9NYV4, K965R) and CCNK (O75909) were subcloned into pAC-derived vectors^45^ and recombinant proteins were expressed as N-terminal His_6_, His_6_–Spy, StrepII or StrepII–Avi fusions in *Trichoplusia ni* High Five insect cells using the baculovirus expression system (Invitrogen)^46^.

### Expression and purification of DDB1 constructs

Wild-type or mutant forms of full-length or beta-propeller B domain deletion (ΔBPB: a.a. 396–705 deleted) constructs of His_6_–DDB1_ΔBPB_, StrepII–Avi–DDB1, or His6–Spy–DDB1 were purified as previously described for DDB1–DCAF complexes^47^. Briefly, for His-tagged constructs, High Five insect cells expressing the above-mentioned proteins were lysed by sonication in 50 mM Tris–HCl (pH 8.0), 500 mM NaCl, 10% (v/v) glycerol, 10 mM imidazole, 0.25 mM tris(2-carboxyethyl)phosphine (TCEP), 0.1% (v/v) Triton X-100, 1 mM phenylmethylsulfonylfluoride (PMSF), and 1× protease inhibitor cocktail (Sigma). Following ultracentrifugation, the soluble fraction was passed over HIS-Select Ni^2+^ affinity resin (Sigma), washed first with 50 mM Tris–HCl pH 8.0, 500 mM NaCl, 10% glycerol, 0.5 mM TCEP and 10 mM imidazole, then with 50 mM Tris–HCl pH 8.0, 200 mM NaCl, 10% glycerol, 0.5 mM TCEP and 10 mM imidazole, and finally eluted in 50 mM Tris–HCl (pH 8.0), 200 mM NaCl, 10% (v/v) glycerol, 0.5 mM TCEP and 250 mM imidazole. For crystallography, affinity tags were removed by overnight Tobacco Etch Virus (TEV) protease treatment at a 1:50 (w/w) ratio. StrepII-tagged versions of DDB1 were affinity purified using Strep-Tactin Sepharose (IBA) omitting imidazole in lysis, wash and elution buffers, supplementing the elution buffer with 2.5 mM desthiobiotin (IBA), and using 50 mM Tris–HCl (pH 6.8) throughout. For ion exchange chromatography, affinity-purified proteins were diluted in a 1:1 ratio with buffer *A* (50 mM Tris–HCl (pH 8.0), 10 mM NaCl, 2.5% (v/v) glycerol and 0.5 mM TCEP) and passed over an 8 ml Poros 50HQ column. Bound DDB1 was eluted by a linear salt gradient mixing buffer A and buffer B (50 mM Tris–HCl (pH 8.0), 1 M NaCl, 2.5% (v/v) glycerol and 0.5 mM TCEP) over 15 column volumes to a final ratio of 60% buffer B. DDB1-containing fractions were concentrated and subjected to size exclusion chromatography in 50 mM HEPES (pH 7.4), 200 mM NaCl, 2.5% (v/v) glycerol and 0.5 mM TCEP. Peak fractions were concentrated (to a final concentration of approximately 200 µM for DDB1ΔB and 30 µM for full-length DDB1), flash frozen in liquid nitrogen and stored at −80 °C or directly used in crystallization trials.

### Expression and purification of CDK12-cyclin K for biophysical assays

High Five insect cells were infected with CDK12 and cyclin K viruses at a 2:1 ratio to avoid excess expression of cyclin K alone. Cells co-expressing truncated versions of wild-type or mutant His_6_–CDK12 (a.a. 713–1,052 or 713–1,032) and His_6_–Avi-tagged cycK (a.a. 1–267) were lysed by sonication in 50 mM Tris–HCl (pH 6.8), 500 mM NaCl, 10% (v/v) glycerol, 10 mM MgCl_2_, 10 mM imidazole, 0.25 mM TCEP, 0.1% (v/v) Triton X-100, 1 mM PMSF and 1× protease inhibitor cocktail (Sigma). Following ultracentrifugation, the soluble fraction was passed over HIS-Select Ni^2+^ affinity resin (Sigma), washed first with 50 mM Tris–HCl (pH 6.8), 1 M NaCl, 10% (v/v) glycerol, 0.5 mM TCEP and 10 mM imidazole, then with 50 mM Tris–HCl pH 6.8, 500 mM NaCl, 10 % (v/v) glycerol, 0.5 mM TCEP and 10 mM imidazole, and eluted in 50 mM Tris–HCl (pH 6.8), 200 mM NaCl, 10% (v/v) glycerol, 0.25 mM TCEP and 250 mM imidazole. For biophysical assays, the eluted fractions were directly concentrated and passed over a gel filtration column in 50 mM HEPES pH 7.4, 200 mM NaCl, 0.5 mM TCEP and 2.5% (v/v) glycerol. Peak fractions were concentrated to approximately 20 µM, flash frozen in liquid nitrogen and stored at −80 °C

### Expression and purification of CDK12–cyclin K for crystallography

High Five insect cells were infected with CDK12 and cyclin K viruses at a 2:1 ratio as described above. Cells co-expressing truncated versions of wild-type or mutant StrepII–CDK12 (a.a. 713–1,052) and StrepII–cycK (a.a. 1–267) were lysed by sonication in 50 mM Tris–HCl (pH 6.8), 500 mM NaCl, 10% (v/v) glycerol, 10 mM MgCl_2_, 10 mM imidazole, 0.25 mM TCEP, 0.1% (v/v) Triton X-100, 1 mM PMSF and 1× protease inhibitor cocktail (Sigma). Following ultracentrifugation, the soluble fraction was passed over Strep-Tactin Sepharose affinity resin (IBA), washed first with 50 mM Tris-HCl (pH 6.8), 1 M NaCl, 10% (v/v) glycerol and 0.5 mM TCEP, then with 50 mM Tris–HCl pH 6.8, 500 mM NaCl, 10 % (v/v) glycerol and 0.5 mM TCEP, and eluted in 50 mM Tris–HCl (pH 6.8), 200 mM NaCl, 10% (v/v) glycerol, 0.5 mM TCEP and 2.5 mM desthiobiotin (IBA). Affinity tags were subsequently removed by overnight incubation with TEV protease at 1:50 (w/w). Before ion-exchange chromatography, the protein solution was diluted in a 1:1 ratio with buffer A (50 mM Tris–HCl pH 6.8, 10 mM NaCl, 2.5 % (v/v) glycerol and 0.5 mM TCEP) and passed over an 8 ml Poros 50HQ column to remove contaminants. The flow-through was then again diluted in a 1:1 ratio with buffer A and loaded onto an 8 ml Poros 50HS column. Bound proteins were eluted by a linear salt gradient mixing buffer A and buffer B (50 mM Tris–HCl (pH 6.8), 1 M NaCl, 2.5% (v/v) glycerol and 0.25 mM TCEP) over 15 column volumes to a final ratio of 80% buffer B. Poros 50HS peak fractions containing the CDK12–cycK complex were concentrated and subjected to size exclusion chromatography in 50 mM HEPES (pH 7.4), 200 mM NaCl, 2.5% (v/v) glycerol and 0.25 mM TCEP. The concentrated proteins (at approximatel*y* 200 µM) were flash frozen in liquid nitrogen and stored at −80 °C or used directly in crystallization trials.

### Protein labeling

#### Labeling with fluorophore-conjugated maleimides

The SpyTag/SpyCatcher system was employed as a mean of conjugation of the TR-FRET acceptor (Alexa_488_ in a maleimide form) to the protein of interest as described before^15,33^. For this, mutant (S50C) SpyCatcher protein was purified according to published procedures^48^ and was reduced by incubation with dithiothreitol (DTT) (8 mM) at 4 °C for 1 h. DTT was subsequently removed during a gel filtration step in 50 mM HEPES pH 7.4, 200 mM NaCl. Alexa_488_–C5–maleimide was dissolved in 100% dimethyl sulfoxide (DMSO), mixed with the reduced SpyCatcher at a 4:1 ratio, and incubated for 3 h in a vacuum desiccator. The reaction was subsequently quenched with DTT (8 mM), and labeled SpyCatcher was purified through size exclusion chromatography in 50 mM HEPES pH 7.4, 200 mM NaCl and 0.5 mM TCEP. SpyCatcher_Alexa488_ was concentrated to ~50 μM, flash frozen in liquid nitrogen and stored at −80 °C. Purified Spy-tagged DDB1 was mixed with an equimolar concentration of labeled SpyCatcher, incubated for 1 h at room temperature and the labeling efficiency was monitored via sodium dodecyl sulfate–polyacrylamide gel electrophoresis. Labeled protein was flash frozen and stored at −80 °C (~20 μM).

#### Biotinylation

Conjugation of the TR-FRET donor (Strep-Tb) to CDK12–cyclin K was achieved through enzymatic biotinylation. For the biotinylation of His_6_–CDK12 His_6_/cyclin K–Avi, the complex at 20–50 μM was incubated with final concentrations of 2.5 μM BirA enzyme, 0.2 mM d-biotin and 20 mM ATP in 50 mM HEPES (pH 7.4), 200 mM NaCl, 10 mM MgCl_2_ and 0.5 mM TCEP. The reaction was incubated for 10–12 h at 4 °C and biotinylated complex was purified by gel filtration chromatography (in 50 mM HEPES pH 7.4, 200 mM NaCl, 0.5 mM TCEP), concentrated to ~10–20 μM, flash frozen and stored at −80 °C in small aliquots.

#### TR-FRET assay

For TR-FRET-based compound evaluation, a mixture of CDK12–cyclin K_biotin_ (50 nM), _Alexa488_DDB1 (100 nM), terbium-coupled streptavidin (Invitrogen) (4 nM) in a TR-FRET assay buffer (50 mM HEPES pH 7.4, 150 mM NaCl, 0.005% Tween 20, 0.5% DMSO, 0.05% bovine serum albumin, 1 mM TCEP and 2 mM ethylenediaminetetraacetic acid) was plated in a white 384-well microplate (Greiner, 784075) at a volume of 6 µl per well. Compounds were then dispensed digitally from DMSO stocks using the D300 dispenser (Tecan) to yield a 13-point dilution series of each small molecule, with the highest final concentration of 20 µM for poor molecular glues and 2 µM for potent compounds.

TR-FRET measurements were carried out using a PHERAstar FS microplate reader (BMG Labtech) equipped with a 337-520-490 filter set and 60 cycles were recorded over 1 h. A 70 μs delay was employed to reduce background fluorescence. The TR-FRET signal was obtained through calculating the ratio of 520 nm to 490 nm fluorescence, and Prism 9 (GraphPad) was used for further data analysis. The curves resulting from plotting the TR-FRET against compound concentration were fitted using equation (1) to determine the half-maximal effective concentrations (EC_50_).

1 $${y}={\rm{Bottom}}+\frac{\left({{x}}^{{{\mathrm{Hillslope}}}}\right)\times ({{\mathrm{Top}}}-{{\mathrm{Bottom}}})}{{{{\mathrm{EC}}}}_{50}^{{{\mathrm{Hillslope}}}}+\,{{x}}^{{{\mathrm{Hillslope}}}}\,}$$

Equation (1) shows [Agonist] versus the response from GraphPad Prism 9, where Top and Bottom refer to the curve plateaus.

Over 30 of the tested small molecules showed in vitro TR-FRET EC_50_ values below 50 nM. As the differences between such tight binders cannot be adequately judged using the current assay setup, we repeated the TR-FRET assay for selected compounds at a lower protein concentration (10 nM of CDK12–cyclin K) and determined their dissociation constants (*K*_d_) using a quadratic equation fit appropriate for cases where the expected dissociation constant value is below the protein concentration used (equation (2)):

2 $${y}={\rm{Bottom}}+({\rm{Top}}-{\rm{Bottom}})\,\left(\frac{{K}_{{\mathrm{d}}}+L+x-\sqrt{({\left({K}_{{\mathrm{d}}}+L+x\right)}^{2}-4{Lx})}}{2L}\right)$$

Equation (2) is a quadratic binding equation used for *K*_d_ determination,where Top and Bottom refer to the curve plateaus. Ligand concentration (*L*) was constrained to 50 nM.

Lowering of the CDK12–cyclin K concentration from 50 nM to 10 nM resulted in a much smaller assay window but yielded *K*_d_ values in the subnanomolar range for the top compounds (DS17 and DS73), while showing no difference for the weak recruiter roscovitine (Extended Data Fig. 6b). This indicates that the tightest glues, which recruit DDB1 with picomolar affinities, lie below the limit of detection of the TR-FRET assay, with DS17 showing binding so tight that it cannot be adequately quantified in either setup. Nonetheless, the reported affinity measurements still allow us to establish an approximate rank order of the compounds’ in vitro activities, and we note that the EC_50_ values are overestimated for the most potent molecular glues.

#### DDB1–CUL4–RBX1 reconstitution and in vitro CUL4 neddylation

In vitro CRL4 reconstitution and CUL4 neddylation were performed as described before^49^. Briefly, His_6_–CUL4A/His_6_–RBX1 at 3.5 µM was incubated with His_6_–DDB1 at 3 µM in a reaction mixture containing 3.8 μM NEDD8, 50 nM NAE1/UBA3 (E1), 30 nM UBC12 (E2), 1 mM ATP, 50 mM Tris (pH 7.5), 100 mM NaCl, 2.5 mM MgCl_2_, 0.5 mM DTT and 5% (v/v) glycerol for 1.5 h at room temperature. Neddylated and gel filtration-purified DDB1–CUL4–RBX1 (_N8_DDB1–CUL4–RBX1) was concentrated to 5–15 μM, flash frozen and stored at −80 °C.

#### Crystallization and data collection

A crystallization solution of purified and TEV-cleaved DDB1ΔB (70 µM), CDK12–cyclin K (80 µM) and compound (80–120 µM) in SEC buffer (50 mM HEPES 7.4, 200 mM NaCl and 0.25 mM TCEP) was set up in two-drop 96-well plates (Swissci) for sitting-drop crystallization. A liquid handling system Phoenix (Art Robbins Instruments, Dunn Labortechnik GmbH) was used to pipette crystallization drops containing 200 nl of the protein complex solution mixed with 200 nl of reservoir solution. Fine screens and additive screens were formulated around the two conditions in which the DDB1ΔB–CDK12–cyclin K-CR8 complex originally crystallized (0.9 M ammonium citrate tribasic pH 7.0 or 1.4 M ammonium sulfate and 70 mM HEPES pH 7.0) (ref. ^15^). The plates were incubated at 20 °C, and initial crystal formation was usually observed within 2–5 days.

Crystals designated for data collection were cryoprotected by supplementing the 400 nl drop with 1 µl of reservoir solution containing ethylene glycol (25 % (v/v)) as cryoprotectant. Crystals were flash frozen in liquid nitrogen. Diffraction data collection was performed at the Swiss Light Source (Paul-Scherrer-Institute, Villigen, Switzerland) beamline PXII (X10SA) equipped with an Eiger2 16M detector (Dectris). The wavelength was set to 1 Å and the crystal was cooled to 100 K in a liquid nitrogen cryo-jet.

#### Structure determination and model building

Pipedream (GlobalPhasing) was used to execute the following steps automatically. Data processing was performed using X-ray Detector Software^50^. Space group determination was done with POINTLESS^51^, and datasets were merged and scaled with AIMLESS^51^. Intensities were converted into structure factor amplitudes with STARANISO (Global Phasing)^52^ by applying a weighted CC_1/2_ of 0.3 resolution cutoff.

The structures of the different DDB1ΔB–CDK12–cyclin K-compound complexes were solved by means of molecular replacement with PHASER^53^ using a previously solved structure of the complex induced by CR8 (Protein Data Bank (PDB) 6TD3 (ref. ^15^); with ligand deleted) as the search model. Reciprocal space refinement was performed using phenix.refine^54^, followed by iterative real space refinement in COOT^55^. Ligand restrains were generated with jligand^56^. In case of ambiguities in the density in ligand-proximal regions (compounds dCeMM3, dCeMM4, HQ461, DS15, DS30, roscovitine and DS59), additional restrained refinement (Molecular Dynamics Flexible Fitting) based on available density maps was performed with ISOLDE^57^ to inform on the preferred ligand conformation, which was followed by a phenix refinement in each case. We acknowledge that in certain cases ambiguities persist, and we therefore suggest appropriate caution when interpreting the ternary complex structures, as well as refer the readers to Supplementary Figs. 1 and 2 where the density maps and omit maps are shown for each structure. When no density was present for certain ligand regions (DS50 and DS06), those regions were set to zero occupancy, which is clearly indicated in figure panels and legends throughout.

Omit maps were generated through three refinement macrocycles with one cycle of simulated annealing during the second macrocycle. The ligand was set to zero occupancy and atoms in 5 Å radius around the ligand were restrained to avoid refinement into the ligand density.

Structure validation was carried out with Molprobity^58^, and visualizations were generated with PyMol (Schrödinger). Interface analysis was performed using PISA^59^ and pocket dimensions were evaluated using CASTp (ref. ^60^).

#### Ligand docking

Ligand docking at the DDB1–CDK12 interface (structure 6TD3 (ref. ^15^) minimized using OPLS3e force field, processed for receptor grid generation using default settings, and with the CR8 ligand removed) was performed with Glide (Schrödinger)^61^. For docking of the cyclin K degrader set evaluated in the study, ligands were input as a SMILE strings, prepared using LigPrep (OPLS3e force field) and docked using XP precision^62^.

For the R_2_ group screen, CR8 derivatives with a library of 26,736 R_2_ groups possessing commercially available precursors was docked at the DDB1–CDK12 interface. Briefly, the ‘In-Stock Decorators’ library was downloaded from Chemspace and filtered for aliphatic primary amines with the ligfilter module using LigPrep. The r_group_enumeration tool was then used to exchange the R_2_ aminobutanol moiety in the CR8 ligand for the generated primary amine fragments from the previous LigPrep step. Docking was performed with SP precision.

For the docking of a kinase inhibitor library, a kinase inhibitor catalog containing commercially available compounds was downloaded from ZINC, prepared using LigPrep (OPLS3e force field), and a database was created using Phase (Schrödinger). The library was docked with HTVS precision.

#### Lanthascreen kinase binding assay

Lanthascreen experiments were performed using commercially available reagents (Thermo Fisher) and according to the manufacturer’s instructions. Briefly, a His-tagged kinase (CDK2–cyclin A, CDK9–cyclin T or CDK12–cyclin K at a final concentration of 5 nM) was mixed with a biotin anti-His antibody (PV6089; final concentration of 2 nM), Lanthascreen Europium-Streptavidin (PV5899; 2 nM) and Tracer 236 (PV5899; final concentration of 30 nM for CDK9, 100 nM for CDK2 and 200 nM for CDK12) in kinase buffer A (PV3189). A serial dilution of a test compound from a DMSO stock was prepared in kinase buffer A. The kinase solution and compound dilution series were mixed in a black 384-well microplate (Greiner, 784076) to a final volume of 12–15 µl. After excitation of europium (Eu) fluorescence at 337 nm, emissions at 665 nm (Eu) and 620 nm (Alexa647) were measured with a 100 μs delay to reduce background fluorescence and the reactions were followed over 60 min using a PHERAstar FS microplate reader (BMG Labtech). TR-FRET signal was calculated through the 665:620 nm ratio, and data were analyzed with Prism 9 (GraphPad) using equation (3):

3 $${y}={\rm{Bottom}}+\frac{({{\mathrm{Top}}}-{{\mathrm{Bottom}}})}{1+\,{\frac{{{\rm{IC}}}_{50}}{x}}^{{{\mathrm{Hillslope}}}}\,}$$

Equation (3) shows [Inhibitor] versus the response from GraphPad Prism 9, where Top and Bottom refer to the curve plateaus.

### Cyclin K reporter stability analysis

HEK293T–Cas9 cells expressing the cyclin K–eGFP degradation reporter were resuspended at 1 × 10^6^ ml^−1^ and 50 µl of cell suspension was seeded in non-cell-culture-treated 384-well plates. Shortly after, cells were treated with drug or DMSO for 5 h. Subsequently, cells were fixed with 50 µl of 4% paraformaldehyde solution (Chem Cruz, sc-281692) and stored at 4 °C. The fluorescent signal was quantified by flow cytometry (LSRFortessa flow cytometer, BD Biosciences). Using FlowJo (flow cytometry analysis software, BD Biosciences), the geometric mean of the eGFP and mCherry fluorescent signal for round and mCherry-positive cells was calculated. The ratio of eGFP to mCherry was normalized to the average of three DMSO-treated controls.

### Drug sensitivity assays

HEK293T–Cas9 cells were resuspended at 0.15 × 10^6^ ml^−1^ treated with DMSO or 100 nM MLN4924 and plated on 384-well plates. After 2 h, the indicated drugs were dispensed with a D300 digital dispenser (Tecan). After 3 days of drug exposure, cell viability was assessed using the CellTiter-Glo luminescent assay (Promega, G7572) on an CLARIOstar Plus, MARS 3.4 (BMG LabTech). Cell viabilities were calculated relative to DMSO controls. The half-maximum effective concentration (EC_50_) values were derived from standard four-parameter log-logistic curves fitted with the ‘dr4pl’ R package.

### Label-free quantitative mass spectrometry

#### Sample preparation

MDA-MB-231 cells were treated with DMSO or a cyclin K degrader compound at 1 µM for 5 h. Cells were collected by centrifugation and washed with phosphate-buffered saline before snap freezing in liquid nitrogen. Cells were lysed by addition of lysis buffer (8 M urea, 50 mM NaCl, 50 mM 4-(2-hydroxyethyl)-1-piperazineethanesulfonic acid (EPPS) pH 8.5, and protease and phosphatase inhibitors) and homogenization by bead beating (BioSpec) for three repeats of 30 s at 2,400 rpm. Bradford assay was used to determine the final protein concentration in the clarified cell lysate. Then 50 µg of protein for each sample was reduced, alkylated and precipitated using methanol/chloroform as previously described^63^, and the resulting washed precipitated protein was allowed to air dry. Precipitated protein was resuspended in 4 M urea, 50 mM HEPES pH 7.4, followed by dilution to 1 M urea with the addition of 200 mM EPPS, pH 8. Proteins were first digested with LysC (1:50; enzyme:protein) for 12 h at room temperature. The LysC digestion was diluted to 0.5 M urea with 200 mM EPPS pH 8 followed by digestion with trypsin (1:50; enzyme:protein) for 6 h at 37 °C. Sample digests were acidified with formic acid to a pH of 2–3 before desalting using C18 solid phase extraction plates (SOLA, Thermo Fisher Scientific). Desalted peptides were dried in a vacuum-centrifuged and reconstituted in 0.1% formic acid for liquid chromatography–mass spectrometry analysis.

Data were collected using a TimsTOF Pro2 (Bruker Daltonics) coupled to a nanoElute LC pump (Bruker Daltonics) via a CaptiveSpray nano-electrospray source. Peptides were separated on a reversed-phase C_18_ column (25 cm × 75 µm inner diameter, 1.6 µM, IonOpticks) containing an integrated captive spray emitter. Peptides were separated using a 50 min gradient of 2–30% buffer B (acetonitrile in 0.1% formic acid) with a flow rate of 250 nl min^−1^ and column temperature maintained at 50 °C.

Data-dependent acquisition (DDA) was performed in Parallel Accumulation-Serial Fragmentation (PASEF) mode to determine effective ion mobility windows for downstream diaPASEF data collection^64^. The ddaPASEF parameters included: 100% duty cycle using accumulation and ramp times of 50 ms each, one Trapped Ion Mobility Spectrometry(TIMS)-MS scan and ten PASEF ramps per acquisition cycle. The TIMS-MS survey scan was acquired between 100 *m*/*z* and 1,700 *m*/*z* and 1/*K*_0_ of 0.7–1.3 V s cm^−2^. Precursors with one to five charges were selected, and those that reached an intensity threshold of 20,000 arbitrary units were actively excluded for 0.4 min. The quadrupole isolation width was set to 2 *m*/*z* for *m*/*z* < 700 and 3 *m*/*z* for *m*/*z* > 800, with the *m*/*z* between 700 *m*/*z* and 800 *m/z* being interpolated linearly. The TIMS elution voltages were calibrated linearly with three points (Agilent ESI-L Tuning Mix Ions; 622, 922 and 1,222 *m*/*z*) to determine the reduced ion mobility coefficients (1/*K*_0_). To perform diaPASEF, the precursor distribution in the DDA *m*/*z*-ion mobility plane was used to design an acquisition scheme for data-independent acquisition (DIA) data collection that included two windows in each 50 ms diaPASEF scan. Data were acquired using 16 of these 25 Da precursor double window scans (creating 32 windows) that covered the diagonal scan line for doubly and triply charged precursors, with singly charged precursors able to be excluded by their position in the *m*/*z*-ion mobility plane. These precursor isolation windows were defined between 400 *m*/*z* and 1,200 *m*/*z* and 1/*K*_0_ of 0.7–1.3 V s cm^−2^.

#### LC-MS data analysis

The diaPASEF raw file processing and controlling peptide and protein level false discovery rates, assembling proteins from peptides, and protein quantification from peptides was performed using directDIA analysis in Spectronaut 14 (Version 15.5.211111.50606, Biognosys). DirectDIA mode includes first extracting the DIA data into a collection of MS2 spectra which are searched using Spectronaut’s Pulsar search engine. The search results are then used to generate a spectral library which is then employed for the targeted analysis of the DIA data. MS/MS spectra were searched against a Swissprot human database (January 2021). Database search criteria largely followed the default settings for directDIA including: tryptic with two missed cleavages, fixed carbomidomethylation of cysteine, and variable oxidation of methionine and acetylation of protein N-termini and precursor Q-value (FDR) cut-off of 0.01. Precursor quantification was performed using MS1 areas, cross run normalization was set to localised and imputation strategy was set to no imputation. Proteins with poor quality data were excluded from further analysis (summed abundance across channels of < 100 and mean number of precursors used for quantification < 2). Protein abundances were scaled using in-house scripts in the R framework^65^ and statistical analysis was carried out using the limma package within the R framework^66^.

### RNA-seq

#### Library preparation

A total of 300,000 MDA-MB-231 cells (ATCC) per technical replicate were treated with 1 μM of drug (or 10 μM in the case of less toxic compounds—DS20, DS21, DS25, 919278, DS50 and roscovitine) for 6 h. Pretreatment of relevant conditions with 0.1 μM MLN4924 (MedChemExpress) was performed for 2 h before compound treatment. RNA extraction was performed using TRIzol (Life Technologies Company) and quantified using Qubit (Thermo Fisher Scientific). RNA was then treated with DNase I, quantified using Qubit and evaluated using a High Sensitivity RNA kit on a TapeStation (Agilent). ERCC spike-in (Thermo Fisher Scientific) was then added. Total RNA prep, complementary DNA synthesis and library preparation was performed on the basis of Illumina Stranded Total RNA prep, Ligation with Ribo-Zero protocol (Illumina). RNA was rRNA depleted and bead purified using RNAClean XP beads (Beckman Coulter). RNA was then fragmented followed by cDNA synthesis and bead purification using AMPure XP beads (Beckman Coulter). cDNA was then dual indexed, amplified, bead purified, evaluated using a DNA 1000 kit (Agilent) on a TapeStation and quantified using a Qubit. Finally, libraries were pooled and sequenced on a NovaSeqS4 (Illumina), using 150 bp paired-end reads.

### RNA-seq analysis

Sequencing reads were aligned to the human genome (*H.sapiens*-ENSEMBL-GRCh38.r91) and quantified by BrowserGenome^67^. Differential gene expression analysis was performed in R (v4.1.1). In detail, raw counts were imported using the DESeqDataSetFromMatrix function implemented in the DESeq2 package (v.1.32.0) (ref. ^68^). After vst transformation and normalization using default parameters, normalized transcripts with an average expression lower than 10 across all replicates as well as a total overall expression lower than 200 across all samples were filtered out. PCA was performed using all transcripts as input and visualized using ggplot2 (v3.4.2). Differentially expressed genes were computed comparing each sample against the DMSO control separately using a significance cutoff of *P* = 0.05, a log_2_ fold change threshold of 0 and independent hypothesis weighting. The log_2_ fold change shrunken differentially expressed genes passing the filtering criteria corresponding to the figure descriptions were displayed using EnhancedVolcano (v.1.10.0). The following additional packages consitute an essential part of the code but do not infer with data processing directly: useful (v1.2.6), dplyr (v1.0.10), reshape2 (v1.4.4), ggpubr (v0.5.0), vsn (v3.66.0), pheatmap (v1.0.12), pals (v1.7), viridis (v0.6.2), stringr (v1.4.1), tidyr (v1.2.1), tidyverse (v1.3.2), ashr (v2.2), ggrepel (v0.9.2) and IHW (1.26.9).

### Reporting summary

Further information on research design is available in the Nature Portfolio Reporting Summary linked to this article.

## Online content

Any methods, additional references, Nature Portfolio reporting summaries, source data, extended data, supplementary information, acknowledgements, peer review information; details of author contributions and competing interests; and statements of data and code availability are available at 10.1038/s41589-023-01409-z.

### Supplementary information

Supplementary Information Supplementary Note, Tables 2 and 3 and Figs. 1–7.

Reporting Summary

Supplementary Table 1 X-ray data collection and refinement statistics.

Supplementary Code 1 Supplementary code sufficient to reproduce the results of RNA-seq analysis.

Supplementary Data 1 Source data for Supplementary Fig. 4.

Supplementary Data 2 Source data for Supplementary Fig. 5.

### Source data

Source Data Fig. 3 Statistical source data.

Source Data Fig. 4 Statistical source data.

Source Data Fig. 5 Statistical source data.

Source Data Extended Data Fig. 1 Statistical source data.

Source Data Extended Data Fig. 2 Statistical source data.

Source Data Extended Data Fig. 3 Statistical source data.

Source Data Extended Data Fig. 4 Statistical source data.

Source Data Extended Data Fig. 5 Statistical source data.

Source Data Extended Data Fig. 6 Statistical source data.

Source Data Extended Data Fig. 7 Statistical source data.

Source Data Extended Data Fig. 8 Statistical source data.

Source Data Extended Data Fig. 9 Statistical source data.

## Data Availability

Structural data have been deposited in the PDB under the accession codes 8BU1, 8BU2, 8BU3, 8BU4, 8BU5, 8BU6, 8BU7, 8BU9, 8BUA, 8BUB, 8BUC, 8BUD, 8BUE, 8BUF, 8BUG, 8BUH, 8BUI, 8BUJ, 8BUK, 8BUL, 8BUM, 8BUN, 8BUO, 8BUP, 8BUQ, 8BUR, 8BUS and 8BUT. Proteome quantification data are available in the PRIDE repository (PXD041836) or at https://github.com/fischerlab/. The protein-coding sequences employed can be identified through the following Uniprot entry IDs: human wild-type and mutant versions of DDB1 (Uniprot entry Q16531), CDK12 (Q9NYV4, K965R) and CCNK (O75909). Source data are provided with this paper.

## Code availability

Code necessary to reproduce statistical analysis is included as Supplementary Code 1.
