# Supplementary material for: Design principles for cyclin K molecular glue degraders
Source: Nat Chem Biol. 2023 Sep 7;20(1):93–102. doi: 10.1038/s41589-023-01409-z (PMC10746543; doi:10.1038/s41589-023-01409-z)
Supplement: Supplementary file 1 — Supplementary Note, Tables 2 and 3 and Figs. 1–7. [file 41589_2023_1409_MOESM1_ESM.pdf]

nature chemical biology

Article

https://doi.org/10.1038/s41589-023-01409-z

# Design principles for cyclin K molecular glue degraders

In the format provided by the authors and unedited

**Supplementary Note.** This note features first an additional discussion of the SAR around the CR8 scaffold, focused predominantly on derivatives not explicitly described in the main text. Then, synthetic procedures for all derivatives are described.

## Importance of pi-cation interactions – additional notes

To explore the importance of interactions between the DDB1 Arg928 and the solvent-exposed arene moiety of the ligand, we systematically surveyed compounds that feature aliphatic chains (DS01, DS36) or rings (DS12, DS32, DS33, DS37, DS38) as their gluing moieties and found these did not support robust complex formation (**Extended Data Fig. 1f, g**). We further assessed the importance of aromaticity by assessing the activity of otherwise identical derivatives bearing different rings. Comparing the two matched pairs, the cyclohexane-bearing DS32 showed ten-fold weaker binding than DS11, while the DS12-roscovitine pair with equivalent matched rings but on a shorter chain showed comparably poor binding (**Extended Data Fig. 1h**). This is likely due to the shorter chain in roscovitine only supporting complex formation through dispersion forces even when an aromatic ring is present due to its suboptimal position in relation to the DDB1 Arg928 side chain[2].

To investigate the optimal arene placement for π-cation contacts to Arg928, while also probing the dimensions and steric constraints of the DDB1-compound interface, we mapped the cavity with a derivative series bearing phenyl gluing moieties on alkyl chains of varying length (**Extended Data Fig. 2a**). DS11 was a robust glue (**Figure 1c, Extended Data Fig. 1e**) and while derivatives with longer chains (DS09, DS02, DS10; four-six carbon chain) were somewhat tolerated (5-10-fold lower affinity than DS11), shorter chains in roscovitine or DS31 (one-two carbon chain) showed more deleterious effects (12-22-fold lower affinity) (**Extended Data Fig. 2a**). We then probed the impact of larger π-systems by introducing naphthalene-containing gluing moieties. Compound DS08 served as an efficient complex inducer ($EC_{50}$ = 60 ± 2 nM), likely due to favourable interactions of the extended π system with Arg928[24,25], while a naphthalene on a longer chain (DS15) was only accommodated with a suboptimal geometry ($EC_{50}$ = 182 ± 19 nM) (**Figure 1d**, **Extended Data Fig. 2b**). The lower activity observed for DS15 can likely also be explained by the fact that the ternary complex freezing the compound in this suboptimal conformation drives up the entropic penalty. Rigidifying the linker by adding an additional phenyl ring (DS05) led to very poor DDB1 recruitment, while introducing three consecutive rings (DS04) entirely abolished binding (**Extended Data Fig. 2b**). These findings underscore the importance of the correct positioning of the arene for optimal Arg928 interactions and identify steric constraints for bulky compounds that lack conformational plasticity.

## Importance of hydrogen-bonding interactions – additional notes

As the nitrogen in the CR8 pyridine ring could serve as a potential HBA, we systematically explored the importance of its position in the gluing moiety. We tested whether changing the nitrogen position

in the ring impacts ternary complex formation and found that 4-pyridyl (DS44) and 3-pyridyl (DS45) were two- to three-fold poorer recruiters than CR8 (2-pyridyl) (**Extended Data Fig. 3a, b**).

Our structural evaluation highlighted the CDK12 residue Tyr815 in proximity of the binding pocket as a potential additional hydrogen-bonding contact. This residue is only present in CDK12/13 and hence its engagement by the ligand could confer specificity within the CDK family (**Extended Data Fig. 4f**). In an attempt to leverage this residue, we designed WX3 bearing a 2-pyridinone ring instead of the first phenyl ring of CR8 (**Extended Data Fig. 3c, d**). Crystallographic analysis showed the C=O functionality within hydrogen-bonding distance to Tyr815 and the N-H interacting with the Met816 carbonyl, yet the binding affinity did not improve ($EC_{50}$ = 21 ± 1 nM) (**Extended Data Fig. 3c, d**). This is not unexpected, as the Tyr815 side chain is likely to be bound by water, with an additional ligand-Tyr815 interaction more likely to modulate CDK selectivity (*e.g.* by reducing pan-CDK inhibitory activity as this residue is a phenylalanine in most other CDKs, **Extended Data Fig. 4f**) than gluing potency. Other modifications, such as installing a fluorine at this position (DS24), led to decreased affinity (**Extended Data Fig. 2c and 3c, d**).

**Supplementary Information: Synthetic Procedures**

*General Chemistry.* All solvents and reagents were used without any further purification/preparation and were purchased from the following suppliers: Sigma-Aldrich, Fisher Scientific, Alfa Aesar, VWR, J.T. Baker, Acros Organics, Fluka, Enamine, Ambeed, abcr, Toronto Research Chemicals, Key Organics, TCI Chemicals, AstaTech, and AK Scientific. Other starting materials were synthesized using methods found in the literature. The general reaction procedures found below are modified versions of previously published protocols.[1] Reaction progress was monitored using an Agilent 1290 Infinity II analytical ultra-high performance liquid chromatography (UHPLC) instrument coupled to a 6130 Quadrupole mass spectrometer using water/acetonitrile (ACN) gradients (containing 0.1% (v/v) formic acid) as the mobile phase. Gradient was 5-90% ACN in 3.50 min at a flow rate of 0.450 mL/min. The UHPLC was equipped with a 1.8 μm ZORBAX Eclipse Plus C18 column (2.1 x 50 mm). Purification of the compounds was carried out on a preparative Shimadzu ultra-fast liquid chromatography (UFLC) system equipped with a Phenomenex Gemini-NX 5 μm C18 column (250 x 21.2 mm) using water/ACN gradients (containing 0.1% (v/v) trifluoroacetic acid [TFA]) as the mobile phase. Unless mentioned otherwise, the gradient was 1-60% ACN in 29 min at a flow rate of 20 mL/min, and all purified derivatives were isolated as TFA salts. Yields are not reported for each individual step due to the utilization of crude product for subsequent reaction steps, unless otherwise noted. However, final purified yields (over 3 steps) were generally between 5-40% (calculated median was 17%) with obtained masses ranging from 0.5-50 mg.

*High-Resolution Mass Spectrometry (HRMS).* Mass spectrometry spectra were acquired using a Bruker maXis 4G high-resolution mass spectrometer equipped with an electrospray (ESI) ionization source. The samples were directly introduced into the instrument (injection volume = 1 μL) at a rate of 0.3 mL/min using a Thermo Fisher Ultimate 3000 ultra-performance liquid chromatography (UPLC) instrument. The heated capillary temperature was 200 °C and the spray voltage was 4.5 kV. The samples were analyzed in positive ion polarity mode, unless otherwise indicated.

*Nuclear Magnetic Resonance (NMR).* Solvents used for NMR were from Cambridge Isotope Laboratories, Sigma-Aldrich, or Apollo Scientific. All NMR experiments were performed on a Bruker Avance III HD four-channel NMR spectrometer operating at 600.13 MHz proton frequency. The instrument was equipped with a helium cryogenic 5mm four-channel QCI probe (H/C/N/F). The experiments were performed at 298 K and the temperature was calibrated using a methanol standard showing accuracy within +/- 0.2 K. Chemical shifts are reported relative to TMS using residual solvent signals as internal standard ($^{1}H$: acetonitrile 1.94 ppm, dmso 2.50 ppm; $^{13}C$: acetonitrile 1.39 ppm, dmso 39.51 ppm) and coupling constants are given for each derivative, along with the projected assignments

where possible. Carbon shifts were obtained from 1D carbon-13 experiments where possible. For some samples, $^{13}$C shifts had to be obtained from 2D heteronuclear single quantum coherence (HSQC) experiments due to too low concentration for 1D-$^{13}$C experiments and thus quaternary carbons are missing. Several compounds undergo an exchange between two conformations in the intermediate time regime - most likely due to a slow rotation around the C8-N bond and / or the C6-N bond. Atoms in the vicinity of these exchange sites, therefore, show two sets of resonances and for some of these, reduced intensities were observed, but the topology and identity of the compound could be confirmed in all cases. In the assignment only the conformer with the sharper and, hence, more intense lines is reported. Abbreviations: imp (impurity), n/a (not applicable).

**Experimental Procedures Chemistry**

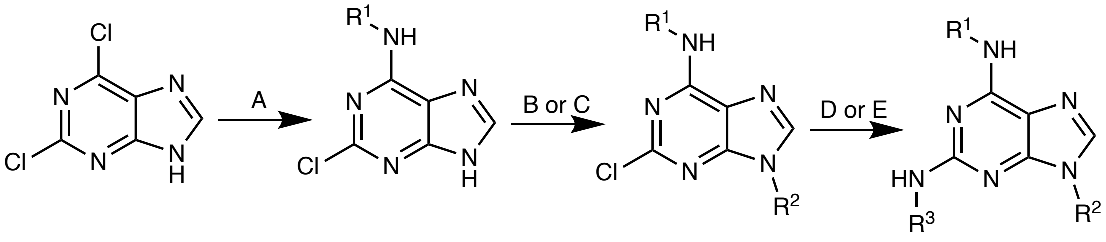


*General Procedure A:* A 400 mM solution of 2,6-dichloropurine (1.0 eq) was made using 1-butanol in a round-bottomed flask. The primary amine (1.2 eq) was added to the solution, followed by triethylamine (1.5 eq). If necessary, additional triethylamine was added to deprotonate the hydrochloric acid (HCl) salt of the primary amine. The reaction was allowed to stir in an oil bath at 110 °C for 3 h. The solution generally turned darker over time. Afterwards, the flask was removed from the heat and allowed to cool to room temperature where the product was then precipitated and washed multiple times with 1-heptane. The product was stored at 4 °C until the next reaction step.

*General Procedure B:* A 250 mM solution of product from Procedure A (1.0 eq) was made in dimethyl sulfoxide (DMSO). Potassium carbonate ($K_2CO_3$; 5.0 eq) was added to the flask, along with 2-bromopropane (2.5 eq). The mixture was allowed to react overnight (~16 h) at room temperature, unless otherwise noted. Water (4x volume excess to organic solvent) was then added to quench the reaction and multiple extractions (generally 3) with ethyl acetate (EtOAc) were performed (using a 1:1 volumetric ratio to the aqueous phase). The organic layers were combined, washed 1-2 times with equivolume brine, concentrated, and dried to give the desired product (stored at 4 °C until the next reaction step).

*General Procedure C (Copper-Mediated):* Product from procedure A (1.0 eq), copper (I) bromide (0.3 eq), and triethylamine (10.0 eq) were added to a round-bottomed flask containing dimethylformamide (DMF) [generally a 150 mM solution of product from procedure A is used]. For enhanced reaction

efficiency, a diaryliodonium salt was used, which was synthesized according to previously published work.[2] 4-(mesityl-$A^2$-iodanyl)-1-methyl-1H-pyrazole trifluoromethanesulfonate (1.3 eq) was added to the vessel and allowed to react overnight (~16 h) at 60 °C. Water (4x volume excess to organic solvent) was added to quench the reaction and multiple (generally 3) EtOAc extractions were performed (using a 1:1 volumetric ratio to the aqueous phase). The organic layers were combined, concentrated, and dried to give the desired crude product, which then underwent UHPLC purification (parameters noted in General Chemistry section). The purified product was stored at 4 °C until the next reaction step.

*General Procedure D (Liquid Amines):* Product from procedures B and C (1.0 eq; generally 10-50 mg) were added to a reaction tube containing only the liquid amine (30.0 eq; generally the total volume was 400-1000 μL). The reaction was allowed to proceed at 140 °C overnight (~16 h). Note that some amines may be solid at room temperature, but liquid under the reaction conditions. The resulting product was purified using UHPLC (parameters noted in General Chemistry section) and then stored at -20 °C until use.

*General Procedure E (Solid Amines):* Product from procedures B and C (1.0 eq; generally 10-50 mg) were added to a reaction tube containing DMF to create a ~250 mM solution. Then the amine (10.0 eq) and a base (triethylamine, tributylamine, or pyridine; 30.0 eq) were added to the flask and allowed to react at 140 °C overnight (~16 h). The resulting product was purified using UHPLC (parameters noted in General Chemistry section) and then stored at -20 °C until use.

**(*R*)-2-((6-((5-(ethyl(methyl)amino)pentyl)amino)-9-isopropyl-9*H*-purin-2-yl)amino)butan-1-ol (DS01)**

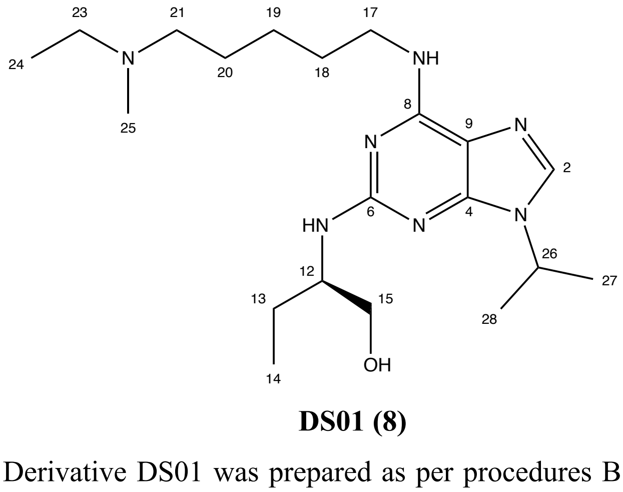


**DS01 (8)**

Derivative DS01 was prepared as per procedures B and D. UHPLC purification was performed at a detection wavelength of 254 nm. $^1$H NMR (600 MHz, $CD_3CN$): δ 7.82 (s, broad, 1H, $H_2$), 5.22 (s, broad, NH), 4.65 (m, broad, 1H, $H_{26}$), 3.99 (s, broad, 3H, $H_{12}$, $H_{17}$), 3.63 (dd, $J$ = 11.2, 4.5 Hz, 1H, $H_{15}$), 3.59

(dd, $J$ = 11.2, 5.4 Hz, 1H, $H_{15}$), 3.17 (m, 1H, $H_{23}$), 3.08 (m, 1H, $H_{21}$), 2.97 (m, 1H, $H_{21}$), 2.72 (d, $J$ = 5.0 Hz, 3H, $H_{25}$), 1.73 (m, 2H, $H_{20}$), 1.71 (m, 2H, $H_{18}$), 1.68 (m, 1H, $H_{13}$), 1.57 (m, 1H, $H_{13}$), 1.52 (d, $J$ = 7.6 Hz, 6H, $H_{27}$, $H_{28}$), 1.45 (m, broad, 2H, $H_{19}$), 1.25 (t, $J$ = 7.3 Hz, 3H, $H_{24}$), 0.96 (t, $J$ = 7.5 Hz, 3H, $H_{14}$). $^{13}C$ NMR (125 MHz, $CD_3CN$): δ 152.5 ($C_4$), 150.4 ($C_6$), 139.7 ($C_2$), 63.7 ($C_{15}$), 56.1 ($C_{21}$), 55.7 ($C_{12}$), 52.1 ($C_{23}$), 48.5 ($C_{26}$), 44.2 ($C_{17}$), 40.1 ($C_{25}$), 29.7 ($C_{18}$), 24.9 ($C_{13}$), 24.1 ($C_{20}$), 24.0 ($C_{19}$), 22.3 ($C_{27}$, $C_{28}$), 10.8 ($C_{14}$), 9.4 ($C_{24}$). HRMS (m/z): $[M+H]^+$ calcd. 392.3132, found 392.3131.

**(*R*)-2-((9-isopropyl-6-((5-phenylpentyl)amino)-9*H*-purin-2-yl)amino)butan-1-ol (DS02)**

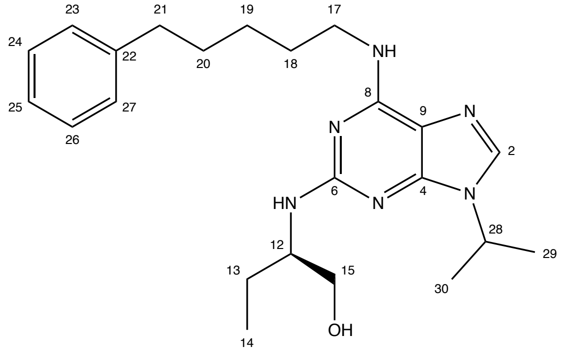

**DS02 (18)**

Derivative DS02 was prepared as per procedures B and D. UHPLC purification was performed at a detection wavelength of 210 nm. $^1H$ NMR (600 MHz, $CD_3CN$): δ 7.80 (s, 1H, $H_2$), 7.24 (m, 2H, $H_{24}$, $H_{25}$), 7.18 (m, 3H, $H_{23}$, $H_{26}$, $H_{27}$), 4.63 (m, 1H, $H_{28}$), 3.97 (s, broad, 3H, $H_{12}$, $H_{17}$), 3.61 (m, 2H, $H_{15}$), 2.60 (t, $J$ = 7.7 Hz, 2H, $H_{21}$), 1.66 (m, 6H, $H_{18}$, $H_{19}$, $H_{20}$), 1.51 (d, $J$ = 6.8 Hz, 6H, $H_{29}$, $H_{30}$), 1.41 (m, broad, 2H, $H_{13}$), 0.95 (t, $J$ = 7.6 Hz, 3H, $H_{14}$). $^{13}C$ NMR (151 MHz, $CD_3CN$): δ 152.5 ($C_4$), 150.3 ($C_6$), 143.6 ($C_{22}$), 139.5 ($C_2$), 129.3 ($C_{23}$, $C_{24}$, $C_{26}$, $C_{27}$), 126.6 ($C_{25}$), 116.5 ($C_9$), 64.1 ($C_{15}$), 55.7 ($C_{12}$), 49.2 ($C_{28}$), 44.5 ($C_{17}$), 36.2 ($C_{21}$), 31.8 ($C_{18}$), 30.2 ($C_{20}$), 26.8 ($C_{19}$), 24.6 ($C_{13}$), 22.2 ($C_{29}$, $C_{30}$), 10.8 ($C_{14}$). HRMS (m/z): $[M+H]^+$ calcd. 411.2867, found 411.2874.

**(*R*)-2-((6-((4-butylbenzyl)amino)-9-isopropyl-9*H*-purin-2-yl)amino)butan-1-ol (DS03)**

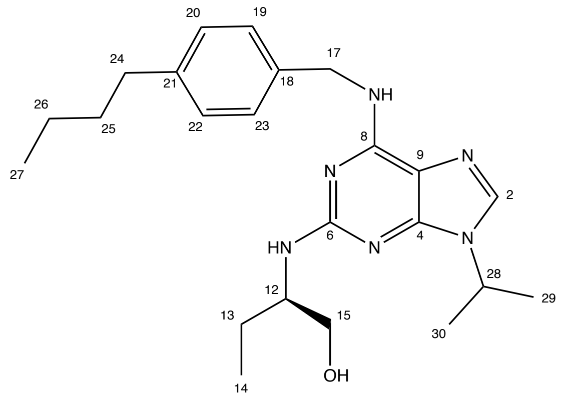

**DS03 (7)**

Derivative DS03 was prepared as per procedures B and D. UHPLC purification was performed at a detection wavelength of 210 nm. $^{1}$H NMR (600 MHz, $CD_3CN$): δ 7.82 (s, 1H, $H_2$), 7.29 (m, 2H, $H_{19}$, $H_{23}$), 7.16 (m, 2H, $H_{20}$, $H_{22}$), 6.80 (s, broad, NH), 5.22 (s, 2H, $H_{17}$), 4.68 (m, 1H, $H_{28}$), 3.97 (s, 2H, $H_{12}$), 3.58 (s, 2H, $H_{15}$), 2.58 (m, 2H, $H_{24}$), 1.65 (m, 2H, $H_{13}$), 1.52 (m, 8H, $H_{25}$, $H_{29}$, $H_{30}$), 1.32 (sext, $J$ = 6.9, 2H, $H_{26}$), 0.94 (m, 3H, $H_{14}$), 0.90 (m, 3H, $H_{27}$). $^{13}$C NMR (151 MHz, $CD_3CN$): δ 152.6 ($C_4$), 150.4 ($C_6$), 143.5 ($C_{21}$), 143.1 ($C_{21}$), 139.6 ($C_2$), 136.1 ($C_{18}$), 129.5 ($C_{19}$, $C_{23}$), 128.4 ($C_{20}$, $C_{22}$), 116.6 ($C_9$), 64.2 ($C_{15}$), 55.7 ($C_{12}$), 49.4 ($C_{28}$), 47.6 ($C_{17}$), 35.7 ($C_{24}$), 34.4 ($C_{25}$), 24.7 ($C_{13}$), 23.0 ($C_{26}$), 22.1 ($C_{29}$, $C_{30}$), 14.1 ($C_{27}$), 10.8 ($C_{14}$). HRMS (m/z): $[M+H]^+$ calcd. 411.2867, found 411.2874.

**(*R*)-2-((6-(([1,1':4',1''-terphenyl]-4-ylmethyl)amino)-9-isopropyl-9*H*-purin-2-yl)amino)butan-1-ol (DS04)**

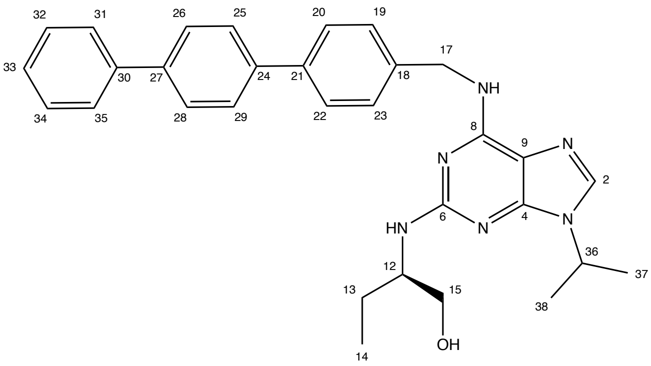

**DS04 (22)**

Derivative DS04 was prepared as per procedures B and D. UHPLC purification was performed at a detection wavelength of 290 nm. The gradient was 50-80% ACN in 15 min at a flow rate of 20 mL/min. $^{1}$H NMR (600 MHz, $CD_3CN$): δ 9.18 (s, NH), 7.83 (s, 1H, $H_2$), 7.73 (s, 4H, $H_{25}$, $H_{26}$, $H_{28}$, $H_{29}$), 7.69 (m, 4H, $H_{19}$, $H_{23}$, $H_{31}$, $H_{35}$), 7.48 (m, 4H, $H_{20}$, $H_{22}$, $H_{32}$, $H_{34}$), 7.38 (m, 1H, $H_{33}$), 6.81 (s, broad, NH), 5.33 (s, 1H, imp), 4.82 (s, 2H, $H_{17}$), 4.67 (s, 1H, $H_{36}$), 3.99 (s, 1H, $H_{12}$), 3.59 (m, 2H, $H_{15}$), 1.65 (m, 2H, $H_{13}$), 1.52 (d, $J$ = 6.8 Hz, 6H, $H_{37}$, $H_{38}$), 0.93 (m, 3H, $H_{14}$). $^{13}$C NMR (151 MHz, $CD_3CN$): δ 152.7 ($C_4$, $C_6$), 141.2 ($C_{30}$), 140.9 (n/a), 140.4 ($C_{24}$), 139.7 ($C_2$), 138.3 ($C_{18}$, $C_{21}$), 129.9 ($C_{32}$, $C_{34}$), 129.1 ($C_{20}$, $C_{22}$), 128.5 ($C_{33}$), 128.3 ($C_{25}$, $C_{26}$, $C_{28}$, $C_{29}$), 128.0 ($C_{19}$, $C_{23}$), 127.9 ($C_{31}$, $C_{35}$), 116.7 ($C_9$), 64.2 ($C_{15}$), 55.7 ($C_{12}$), 49.4 ($C_{36}$), 44.5 ($C_{17}$), 30.6 (imp), 24.8 ($C_{13}$), 22.2 ($C_{37}$, $C_{38}$), 10.8 ($C_{14}$). HRMS (m/z): $[M+H]^+$ calcd. 507.2867, found 507.2876.

**(*R*)-2-((9-isopropyl-6-((4-(naphthalen-2-yl)benzyl)amino)-9*H*-purin-2-yl)amino)butan-1-ol (DS05)**

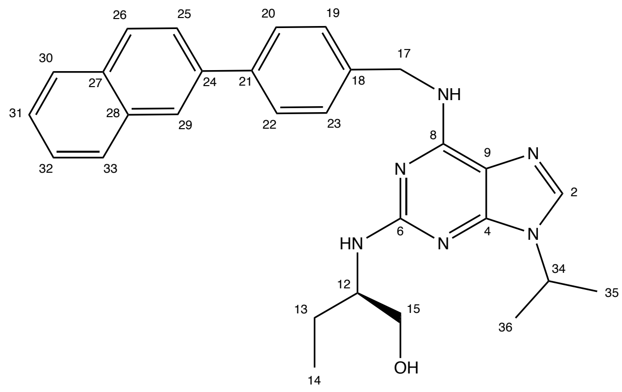

**DS05 (21)**

Derivative DS05 was prepared as per procedures B and D. UHPLC purification was performed at a detection wavelength of 260 nm. The gradient was 50-80% ACN in 15 min at a flow rate of 20 mL/min. $^{1}$H NMR (600 MHz, $CD_3CN$): δ 8.14 (s, 1H, $H_{29}$), 7.96 (d, $J$ = 7.6 Hz, 1H, $H_{26}$), 7.94 (d, $J$ = 7.6 Hz, 1H, $H_{33}$), 7.91 (d, $J$ = 7.6 Hz, 1H, $H_{30}$), 7.80 (dd, $J$ = 8.5, 1.9 Hz, 1H, $H_{25}$), 7.76 (m, broad, 2H, $H_{19}$, $H_{23}$), 7.53 (t, 1H, $H_{32}$), 7.52 (t, 1H, $H_{31}$), 7.52 (d, 2H, $H_{20}$, $H_{22}$), 6.81 (s, broad, NH), 5.34 (s, OH/NH), 4.83 (s, 2H, $H_{17}$), 4.67 (m, 1H, $H_{34}$), 3.99 (s, 1H, $H_{12}$), 3.58 (m, 2H, $H_{15}$), 1.66 (m, 2H, $H_{13}$), 1.52 (d, $J$ = 6.8 Hz, 6H, $H_{35}$, $H_{36}$), 0.95 (m, 3H, $H_{14}$). $^{13}$C NMR (151 MHz, $CD_3CN$): δ 152.7/150.7/141.1/140.7/138.7/138.3/136.4/ 134.7/133.6 ($C_4$ /$C_6$ /$C_8$ /$C_9$ /$C_{18}$/$C_{21}$/$C_{24}$/$C_{27}$/$C_{28}$), 139.7 ($C_2$), 129.4 ($C_{26}$), 129.1 ($C_{20}$/$C_{22}$/$C_{33}$), 128.5 ($C_{30}$), 128.3 ($C_{19}$/$C_{23}$), 127.4 ($C_{32}$), 127.1 ($C_{31}$), 126.5 ($C_{29}$), 126.2 ($C_{25}$), 116.5 ($C_9$), 64.2 ($C_{15}$), 55.8 ($C_{12}$), 49.4 ($C_{34}$), 44.6 ($C_{17}$), 30.6 (imp), 24.8 ($C_{13}$), 22.2 ($C_{35}$), 22.3 ( $C_{36}$), 10.8 ($C_{14}$). HRMS (m/z): $[M+H]^+$ calcd. 481.2710, found 481.2717.

**(*R*)-2-((9-isopropyl-6-(octylamino)-9*H*-purin-2-yl)amino)butan-1-ol (DS06)**

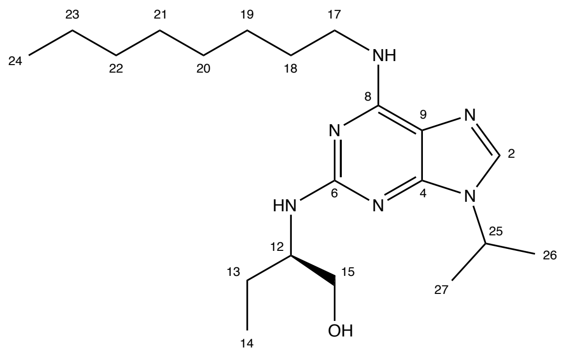

**DS06 (5)**

Derivative DS06 was prepared as per procedures B and D. UHPLC purification was performed at a detection wavelength of 220 nm. The gradient was 50-80% ACN in 15 min at a flow rate of 20 mL/min. $^{1}$H NMR (600 MHz, $CD_3CN$): δ 7.80 (s, 1H, $H_2$), 6.70 (s, broad, NH), 4.64 (m, 1H, $H_{25}$), 3.98 (m, 3H, $H_{12}$, $H_{17}$), 3.58 (m, 2H, $H_{15}$), 1.66 (m, 4H, $H_{13}$, $H_{18}$), 1.51 (d, $J$ = 6.8 Hz, 6H, $H_{26}$, $H_{27}$), 1.38 (s, broad,

2H, $H_{19}$), 1.30 (m, 8H, $H_{20}$, $H_{21}$, $H_{22}$, $H_{23}$), 0.95 (t, $J$ = 7.5 Hz, 3H, $H_{14}$), 0.87 (t, $J$ = 7.3 Hz, 3H, $H_{24}$). $^{13}$C NMR (151 MHz, $CD_3CN$): δ 139.4 ($C_2$), 64.1 ($C_{15}$), 55.7 ($C_{12}$), 48.4 ($C_{25}$), 44.7 ($C_{17}$), 32.5 ($C_{18}$, $C_{22}$), 30.5 ($C_{20}$/$C_{21}$), 29.9 ($C_{20}$/$C_{21}$), 27.1 ($C_{19}$), 24.8 ($C_{13}$), 23.3 ($C_{23}$), 22.2 ($C_{26}$, $C_{27}$), 14.4 ($C_{24}$), 10.8 ($C_{14}$). HRMS (m/z): $[M+H]^+$ calcd. 377.3023, found 377.3026.

**(*R*)-2-((6-((4-((dimethylamino)methyl)benzyl)amino)-9-isopropyl-9*H*-purin-2-yl)amino)butan-1-ol (DS07)**

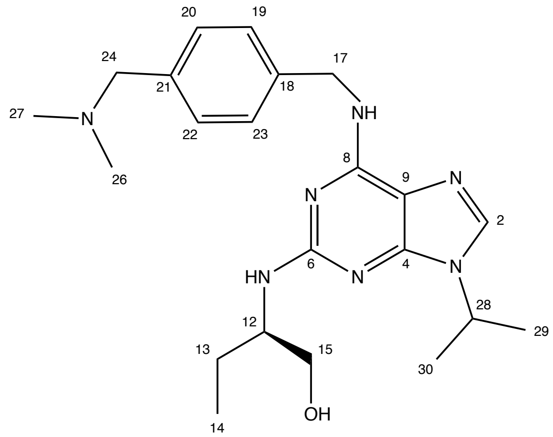


**DS07 (10)**

Derivative DS07 was prepared as per procedures B and D. UHPLC purification was performed at a detection wavelength of 210 nm. $^1$H NMR (600 MHz, $CD_3CN$): δ 7.81 (s, 1H, $H_2$), 7.45 (m, 4H, $H_{19}$, $H_{20}$, $H_{22}$, $H_{23}$), 6.77 (s, broad, NH), 4.78 (m, 2H, $H_{17}$), 4.68 (m, 1H, $H_{28}$), 4.16 (s, 2H, imp), 3.95 (s, 1H, $H_{12}$), 3.61 (m, 2H, $H_{15}$), 3.48 (m, 2H, $H_{24}$), 1.71 (m, 2H, $H_{13}$), 1.52 (d, $J$ = 7.0 Hz, 6H, $H_{29}$, $H_{30}$), 1.19 (d, $J$ = 6.3 Hz, 1H, imp), 0.95 (m, 3H, $H_{14}$). $^{13}$C NMR (151 MHz, $CD_3CN$): δ 132.1 ($C_{18}$), 131.1 ($C_{21}$), 129.0 ($C_{20}$, $C_{22}$), 128.5 ($C_{19}$, $C_{23}$), 116.7 ($C_9$), 64.2 ($C_{15}$), 61.2 ($C_{24}$), 55.7 ($C_{12}$), 49.3 ($C_{28}$), 44.4 ($C_{17}$), 42.8 ($C_{26}$, $C_{27}$), 24.7 ($C_{13}$), 22.2 ($C_{29}$, $C_{30}$), 10.8 ($C_{14}$). HRMS (m/z): $[M+H]^+$ calcd. 412.2819, found 412.2816.

**(*R*)-2-((9-isopropyl-6-((naphthalen-2-ylmethyl)amino)-9*H*-purin-2-yl)amino)butan-1-ol (DS08)**

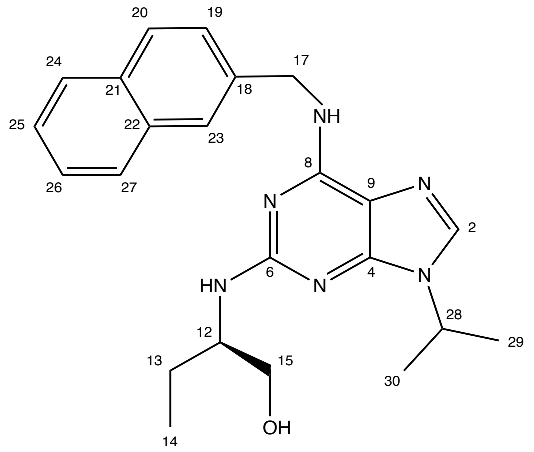


**DS08 (23)**

Derivative DS08 was prepared as per procedures B and D. UHPLC purification was performed at a detection wavelength of 220 nm. $^{1}$H NMR (600 MHz, $CD_3CN$): δ 9.22 (s, broad, NH), 7.87 (m, 1H, $H_{23}$), 7.86 (m, 2H, $H_{19}$, $H_{27}$), 7.84 (m, 1H, $H_{24}$), 7.81 (s, 1H, $H_2$), 7.53 (m, 1H, $H_{20}$), 7.52 (m, 1H, $H_{26}$), 7.49 (m, 1H, $H_{25}$), 6.82 (s, broad, NH), 4.93 (m, 2H, $H_{17}$), 4.67 (m, 1H, $H_{28}$) 3.98 (s, 1H, $H_{12}$), 3.55 (m, 2H, $H_{15}$), 1.63 (m, 2H, $H_{13}$), 1.51 (d, $J$ = 6.8 Hz, 6H, $H_{29}$, $H_{30}$), 0.90 (m, 3H, $H_{14}$). $^{13}$C NMR (151 MHz, $CD_3CN$): δ 139.7 ($C_2$), 136.5 ($C_{18}$), 134.3 ($C_{22}$), 133.7 ($C_{21}$), 129.3 ($C_{19}$), 128.6 ($C_{24}$, $C_{27}$), 127.3 ($C_{26}$), 127.0 ($C_{25}$), 126.9 ($C_{23}$), 126.8 ($C_{20}$) 116.7 ($C_9$), 64.2 ($C_{15}$), 55.8 ($C_{12}$), 49.4 ($C_{28}$) 45.0 ($C_{17}$), 24.7 ($C_{13}$), 22.2 ($C_{29}$, $C_{30}$), 10.8 ($C_{14}$). HRMS (m/z): $[M+H]^+$ calcd. 405.2397, found 405.2403.

**(*R*)-2-((9-isopropyl-6-((4-phenylbutyl)amino)-9*H*-purin-2-yl)amino)butan-1-ol (DS09)**

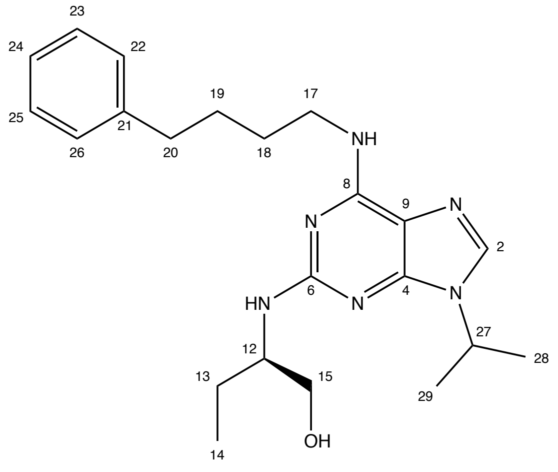


**DS09 (17)**

Derivative DS09 was prepared as per procedures B and D. UHPLC purification was performed at a detection wavelength of 210 nm. $^{1}$H NMR (600 MHz, $CD_3CN$): δ 7.80 (s, 1H, $H_2$), 7.25 (t, $J$ = 7.5 Hz, 2H, $H_{22}$, $H_{26}$), 7.19 (m, 2H, $H_{23}$, $H_{25}$), 7.15 (t, $J$ = 7.4 Hz, 1H, $H_{24}$), 6.70 (s, broad, NH), 4.62 (m, 1H, $H_{27}$), 3.98 (m, 3H, $H_{12}$, $H_{17}$), 3.60 (m, 2H, $H_{15}$), 2.64 (m, 2H, $H_{20}$), 1.68 (m, 6H, $H_{13}$, $H_{18}$, $H_{19}$), 1.51 (m,

6H, $H_{28}$, $H_{29}$), 0.95 (t, $J$ = 7.5 Hz, 3H, $H_{14}$). $^{13}$C NMR (151 MHz, $CD_3CN$): δ 152.5 ($C_4$), 150.4 ($C_6$), 143.8 ($C_{21}$), 139.5 ($C_2$), 129.3 ($C_{23}$, $C_{25}$), 129.2 ($C_{22}$, $C_{26}$), 126.7 ($C_{24}$), 116.6 ($C_9$), 64.1 ($C_{15}$), 55.7 ($C_{12}$), 49.2 ($C_{27}$), 44.4 ($C_{17}$), 35.9 ($C_{20}$), 30.1 ($C_{18}$), 29.1 ($C_{19}$), 24.7 ($C_{13}$), 22.2 ($C_{28}$, $C_{29}$), 10.8 ($C_{14}$). HRMS (m/z): $[M+H]^+$ calcd. 397.2710, found 397.2713.

**(*R*)-2-((9-isopropyl-6-((6-phenylhexyl)amino)-9*H*-purin-2-yl)amino)butan-1-ol (DS10)**

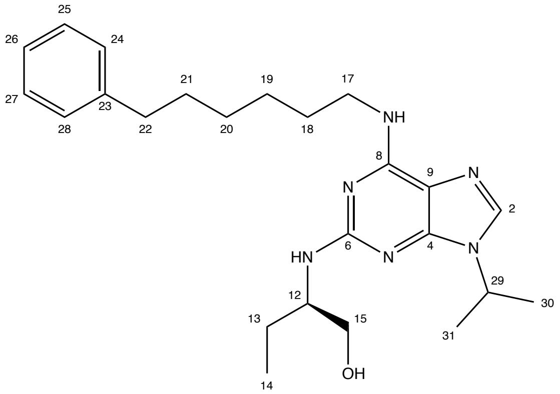


**DS10 (19)**

Derivative DS10 was prepared as per procedures B and D. UHPLC purification was performed at a detection wavelength of 210 nm. $^1$H NMR (600 MHz, $CD_3CN$): δ 7.79 (s, 1H, $H_2$), 7.25 (t, $J$ = 7.5 Hz, 2H, $H_{24}$, $H_{28}$), 7.17 (m, 3H, $H_{25}$, $H_{26}$, $H_{27}$), 6.70 (s, broad, NH), 4.63 (m, 1H, $H_{29}$), 3.96 (s, 3H, $H_{12}$, $H_{17}$), 3.60 (m, 2H, $H_{15}$), 2.59 (t, $J$ = 7.7 Hz, 2H, $H_{22}$), 1.63 (m, 6H, $H_{13}$, $H_{18}$, $H_{21}$), 1.51 (d, $J$ = 6.5 Hz, 6H, $H_{30}$, $H_{31}$), 1.37 (m, 4H, $H_{19}$, $H_{20}$), 0.95 (t, $J$ = 7.5 Hz, 3H, $H_{14}$). $^{13}$C NMR (151 MHz, $CD_3CN$): δ 152.5 ($C_4$), 150.4 ($C_6$), 143.8 ($C_{23}$), 139.5 ($C_2$), 129.3 ($C_{25}$, $C_{27}$), 129.2 ($C_{24}$, $C_{28}$), 126.5 ($C_{26}$), 116.4 ($C_9$), 64.1 ($C_{15}$), 55.7 ($C_{12}$), 49.3 ($C_{29}$), 44.7 ($C_{17}$), 36.3 ($C_{22}$), 32.1 ($C_{21}$), 30.3 ($C_{18}$), 29.5 ($C_{20}$), 26.7 ($C_{19}$), 24.7 ($C_{13}$), 22.1 ($C_{30}$, $C_{31}$), 10.8 ($C_{14}$). HRMS (m/z): $[M+H]^+$ calcd. 425.3023, found 425.3029.

**(*R*)-2-((9-isopropyl-6-((3-phenylpropyl)amino)-9*H*-purin-2-yl)amino)butan-1-ol (DS11)**

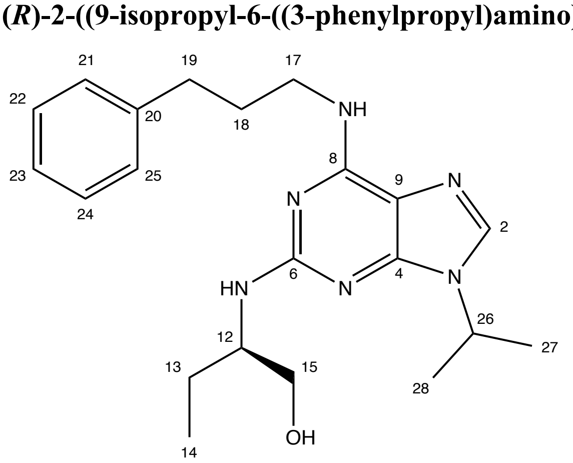


**DS11 (3)**

Derivative DS11 was prepared as per procedures B and D. UHPLC purification was performed at a detection wavelength of 210 nm. $^{1}$H NMR (600 MHz, $CD_3CN$): δ 7.79 (s, 1H, $H_2$), 7.24 (m, 4H, $H_{21}$, $H_{22}$, $H_{24}$, $H_{25}$), 7.17 (m, 1H, $H_{23}$), 6.68 (s, broad, NH), 4.62 (m, 1H, $H_{26}$), 3.96 (s, 3H, $H_{12}$, $H_{17}$), 3.60 (m, 2H, $H_{15}$), 2.71 (d, $J$ = 8.1 Hz, 2H, $H_{19}$), 1.97 (m, 2H, $H_{18}$), 1.61 (m, 2H, $H_{13}$), 1.51 (d, $J$ = 6.8 Hz, 6H, $H_{27}$, $H_{28}$), 0.95 (t, $J$ = 7.5 Hz, 3H, $H_{14}$). $^{13}$C NMR (151 MHz, $CD_3CN$): δ 152.5 ($C_4$), 150.4 ($C_6$), 142.7 ($C_{20}$), 139.5 ($C_2$), 129.3 ($C_{22}$, $C_{24}$), 126.8 ($C_{21}$, $C_{25}$), 120.6 ($C_{23}$), 116.7 ($C_9$), 64.2 ($C_{15}$), 55.7 ($C_{12}$), 49.1 ($C_{26}$), 44.3 ($C_{17}$), 33.3 ($C_{19}$), 32.3 ($C_{18}$), 31.7 (imp), 24.7 ($C_{13}$), 22.1 ($C_{27}$, $C_{28}$), 10.8 ($C_{14}$). HRMS (m/z): $[M+H]^+$ calcd. 383.2554, found 383.2553.

**(*R*)-2-((6-((cyclohexylmethyl)amino)-9-isopropyl-9*H*-purin-2-yl)amino)butan-1-ol (DS12)**

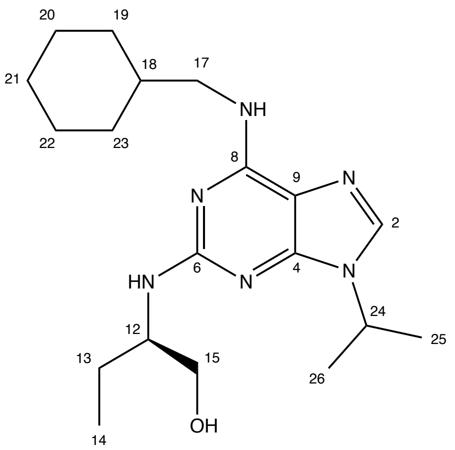


**DS12 (15)**

Derivative DS12 was prepared as per procedures B and D. UHPLC purification was performed at a detection wavelength of 210 nm. $^{1}$H NMR (600 MHz, $CD_3CN$): δ 7.80 (s, 1H, $H_2$), 6.79 (s, broad, NH), 4.63 (m, 1H, $H_{24}$), 3.97 (s, 1H, $H_{12}$), 3.85 (m, 2H, $H_{17}$), 1.79 (m, 2H, $H_{19}/H_{23}$), 1.71 (dt, $J$ = 12.8, 3.3 Hz, 2H, $H_{13}$), 1.65 (m, 2H, $H_{21}$), 1.51 (d, $J$ = 5.2 Hz, 6H, $H_{25}$, $H_{26}$), 1.21 (t, $J$ = 7.0 Hz, 4H, $H_{20}$, $H_{22}$), 1.01 (t, $J$ = 6.9 Hz, 3H, $H_{18}$, $H_{19}/H_{23}$), 0.95, (t, $J$ = 7.5 Hz, 3H, $H_{14}$). $^{13}$C NMR (151 MHz, $CD_3CN$): δ 152.6 ($C_4$), 150.5 ($C_6$), 139.4 ($C_2$), 116.6 ($C_9$), 64.0 ($C_{15}$), 55.8 ($C_{12}$), 50.6 (imp), 49.2 ($C_{24}$), 44.1 ($C_{17}$), 31.4 ($C_{18}$), 31.1 ($C_{19}$, $C_{23}$), 27.1 ($C_{21}$), 26.6 ($C_{20}$, $C_{22}$), 24.7 ($C_{13}$), 22.2 ($C_{25}$, $C_{26}$), 10.8 ($C_{14}$). HRMS (m/z): $[M+H]^+$ calcd. 361.2710, found 361.2716.

**(*R*)-2-((6-((2-(2-ethoxyethoxy)ethyl)amino)-9-isopropyl-9*H*-purin-2-yl)amino)butan-1-ol (DS13)**

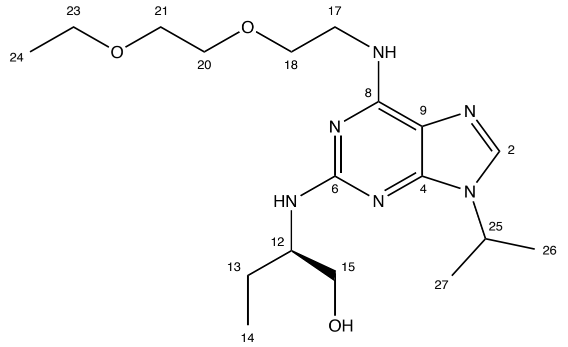


**DS13 (76)**

Derivative DS13 was prepared as per procedures B and D. UHPLC purification was performed at a detection wavelength of 220 nm. $^{1}$H NMR (600 MHz, $CD_3CN$): δ 7.85 (s, 1H, $H_2$), 7.13 (s, broad, NH), 4.65 (m, 1H, $H_{25}$), 4.08 (m, 2H, $H_{18}/H_{20}/H_{21}$), 3.98 (s, 1H, $H_{12}$), 3.68 (t, $J$ = 5.2 Hz, 2H, $H_{18}/H_{20}/H_{21}$), 3.59 (m, 4H, $H_{15}$, $H_{18}/H_{20}/H_{21}$), 3.43 (m, 2H, $H_{23}$), 1.63 (m, 2H, $H_{13}$) 1.51 (d, $J$ = 6.8 Hz, 6H, $H_{26}$, $H_{27}$), 1.08 (m, 3H, $H_{24}$), 0.95 (t, $J$ = 7.5 Hz, 3H, $H_{14}$). $^{13}$C NMR (151 MHz, $CD_3CN$): δ 152.6 ($C_4$), 150.6 ($C_6$), 139.7 ($C_2$), 116.4 ($C_9$), 71.1 ($C_{18}/C_{20}/C_{21}$), 70.4 ($C_{18}/C_{20}/C_{21}$), 70.1 ($C_{18}/C_{20}/C_{21}$), 67.0 ($C_{23}$), 65.9 ($C_{15}$), 55.9 ($C_{12}$), 49.3 ($C_{25}$), 44.6 ($C_{17}$), 24.7 ($C_{13}$), 22.2 ($C_{26}$, $C_{27}$), 15.5 ($C_{24}$), 10.8 ($C_{14}$). HRMS (m/z): $[M+H]^+$ calcd. 381.2609, found 381.2616; $[M+Na]^+$ calcd. 403.2428, found 403.2432.

**(*R*)-6-(((2-((1-hydroxybutan-2-yl)amino)-9-isopropyl-9*H*-purin-6-yl)amino)methyl)naphthalen-2-ol (DS14)**

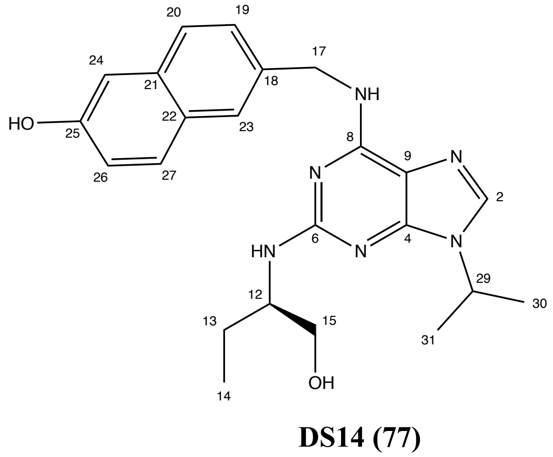


**DS14 (77)**

Derivative DS14 was prepared as per procedures B and D. UHPLC purification was performed at a detection wavelength of 230 nm. $^{1}$H NMR (600 MHz, $CD_3CN$): δ 9.20 (s, broad, NH), 7.86 (s, 1H, $H_2$), 7.78 (s, 1H, $H_{23}$), 7.72 (m, 2H, $H_{19}$, $H_{27}$), 7.48 (m, 1H, $H_{20}$), 7.22 (m, 1H, $H_{24}$), 7.09 (m, 1H, $H_{26}$), 4.88 (m, 2H, $H_{17}$), 4.67 (m, 1H, $H_{29}$), 4.41 (q, $J$ = 7.1 Hz, 2H, imp), 3.99 (s, broad, 1H, $H_{12}$), 1.52 (m, 2H, $H_{13}$), 1.34 (m, 6H, $H_{30}$, $H_{31}$), 0.96 (t, $J$ = 7.5 Hz, 3H, $H_{14}$). $^{13}$C NMR (151 MHz, $CD_3CN$): δ 161.1 ($C_6$), 156.8 ($C_{25}$), 141.7 ($C_2$), 135.0 ($C_{18}/C_{21}$), 134.4 ($C_{18}/C_{21}$), 130.1 ($C_{27}$), 129.5 ($C_{22}$), 128.0 ($C_{19}$), 127.4

($C_{20}$), 127.0 ($C_{23}$), 120.8 ($C_{26}$), 116.3 ($C_9$), 109.1 ($C_{24}$), 70.7 (imp), 61.0 ($C_{15}$), 56.5 ($C_{12}$), 49.5 ($C_{29}$), 45.3 ($C_{17}$), 24.6 ($C_{13}$), 22.2 ($C_{30}$, $C_{31}$), 10.8 ($C_{14}$). HRMS (m/z): $[M+H]^+$ calcd. 421.2347, found 421.2344.

**(*R*)-2-((9-isopropyl-6-((5-(naphthalen-1-yl)pentyl)amino)-9*H*-purin-2-yl)amino)butan-1-ol (DS15)**

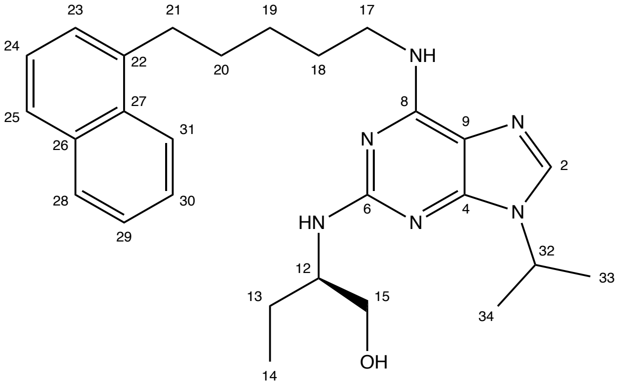


**DS15 (24)**

Derivative DS15 was prepared as per procedures B and D. UHPLC purification was performed at a detection wavelength of 225 nm. $^1$H NMR (600 MHz, $CD_3CN$): δ 8.04 (m, 1H, $H_{31}$), 7.84 (m, 1H, $H_{28}$), 7.74 (s, 1H, $H_2$), 7.69 (m, 1H, $H_{25}$), 7.45 (m, 2H, $H_{29}$, $H_{30}$), 7.36 (m, 1H, $H_{24}$), 7.31 (m, 1H, $H_{23}$), 6.67 (s, broad, NH), 4.61 (m, 1H, $H_{32}$), 3.95 (m, 3H, $H_{12}$, $H_{17}$), 3.56 (m, 2H, $H_{15}$), 3.05 (m, 2H, $H_{21}$), 1.71 (m, 6H, $H_{13}$, $H_{18}$, $H_{20}$), 1.49 (m, 8H, $H_{19}$, $H_{33}$, $H_{34}$), 0.93 (m, 3H, $H_{14}$). $^{13}$C NMR (151 MHz, $CD_3CN$): δ 152.5 ($C_4$), 150.4 ($C_6$), 139.6 ($C_2$), 134.9 ($C_{22}$), 132.7 ($C_{27}$), 129.5 ($C_{28}$), 127.3 ($C_{25}$), 127.0 ($C_{30}$), 126.7 ($C_{23}$), 126.6 ($C_{24}$), 126.5 ($C_{29}$), 124.8 ($C_{31}$), 116.4 ($C_9$), 64.1 ($C_{15}$), 55.8 ($C_{12}$), 48.3 ($C_{32}$), 44.5 ($C_{17}$), 33.4 ($C_{21}$), 31.1 ($C_{20}$), 30.2 ($C_{18}$), 29.6 (imp), 27.4 (imp), 27.1 ($C_{19}$), 24.7 ($C_{13}$), 22.1 ($C_{33}$, $C_{34}$), 10.8 ($C_{14}$). HRMS (m/z): $[M+H]^+$ calcd. 461.3023, found 461.3029.

**(*R*)-2-((6-(([1,1'-biphenyl]-4-ylmethyl)amino)-9-isopropyl-9*H*-purin-2-yl)amino)butan-1-ol (DS16)**

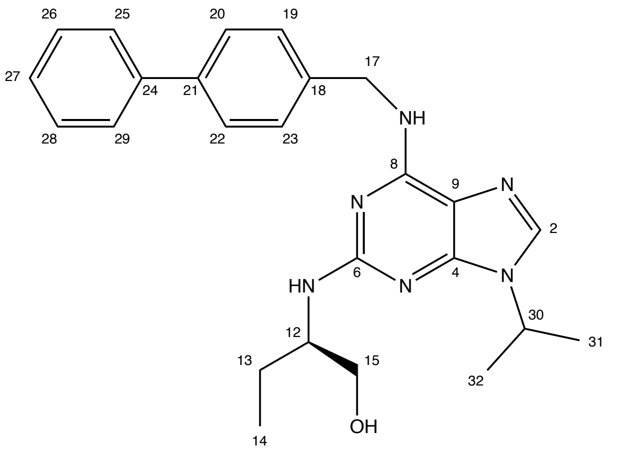

**DS16 (2)**

Derivative DS16 was prepared as per procedures B and D. UHPLC purification was performed at a detection wavelength of 210 nm. $^{1}$H NMR (600 MHz, $CD_3CN$): δ 7.83 (s, 1H, $H_2$), 7.62 (m, 4H, $H_{25}$, $H_{26}$, $H_{28}$, $H_{29}$), 7.46 (m, 4H, $H_{19}$, $H_{20}$, $H_{22}$, $H_{23}$), 7.36 (m, 1H, $H_{27}$), 6.80 (s, broad, NH), 5.32 (s, OH/NH), 4.81 (s, 2H, $H_{17}$), 4.67 (s, 1H, $H_{30}$), 3.98 (s, 1H, $H_{12}$), 3.57 (m, 2H, $H_{15}$), 1.66 (m, 2H, $H_{13}$), 1.52 (d, $J$ = 6.8 Hz, 6H, $H_{31}$, $H_{32}$), 0.91 (m, 3H, $H_{14}$). $^{13}$C NMR (151 MHz, $CD_3CN$): δ 152.6 ($C_4$), 150.5 ($C_6$), 141.2 ($C_{24}$), 139.8 ($C_{18}$), 138.2 ($C_2$), 129.9 ($C_{21}$), 129.1 ($C_{26}$/$C_{29}$), 128.9 ($C_{26}$/$C_{29}$), 128.5 ($C_{20}$, $C_{22}$), 128.2 ($C_{19}$, $C_{23}$), 128.0 ($C_{25}$), 127.9 ($C_{27}$), 120.0 ($C_{28}$), 116.2 ($C_9$), 64.2 ($C_{15}$), 55.8 ($C_{12}$), 49.6 ($C_{30}$), 44.7 ($C_{17}$), 24.7 ($C_{13}$), 22.2 ($C_{31}$, $C_{32}$), 10.8 ($C_{14}$). HRMS (m/z): $[M+H]^+$ calcd. 431.2554, found 431.2561.

**(*R*)-2-((6-(((5,6-dichloro-1*H*-benzo[*d*]imidazol-2-yl)methyl)amino)-9-isopropyl-9*H*-purin-2-yl)amino)butan-1-ol (DS17)**

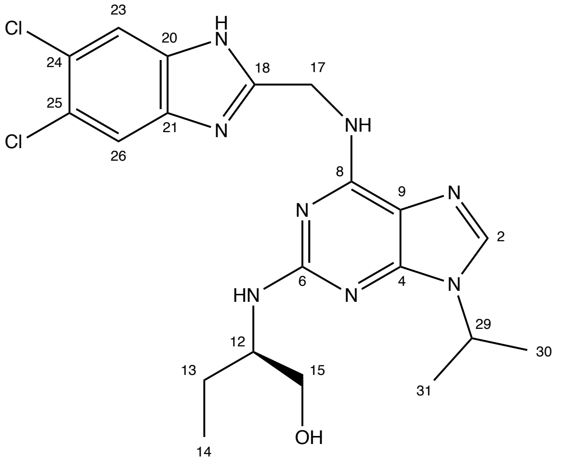

**DS17 (66)**

Derivative DS17 was prepared as per procedures B and D. UHPLC purification was performed at a detection wavelength of 210 nm. $^{1}$H NMR (600 MHz, $CD_3CN$): δ 7.81 (s, 2H, $H_{23}$, $H_{26}$), 5.10 (m, 2H,

$H_{17}$), 4.69 (m, 1H, $H_{29}$), 3.99 (s, 1H, $H_{12}$), 3.49 (m, 2H, $H_{15}$), 1.53 (d, $J$ = 6.8 Hz, 6H, $H_{30}$, $H_{31}$), 0.85 (s, 3H, $H_{14}$). $^{13}$C NMR (151 MHz, $CD_3CN$): δ 117.1 ($C_{23}$, $C_{26}$), 58.2 ($C_{15}$), 56.3 ($C_{12}$), 49.7 ($C_{29}$), 24.9 ($C_{13}$), 22.5 ($C_{30}$, $C_{31}$), 10.7 ($C_{14}$). HRMS (m/z): $[M+H]^+$ calcd. 463.1523, found 463.1523.

**_N_-((5,6-dichloro-1_H_-benzo[_d_]imidazol-2-yl)methyl)-9-isopropyl-2-morpholino-9_H_-purin-6-amine (DS18)**

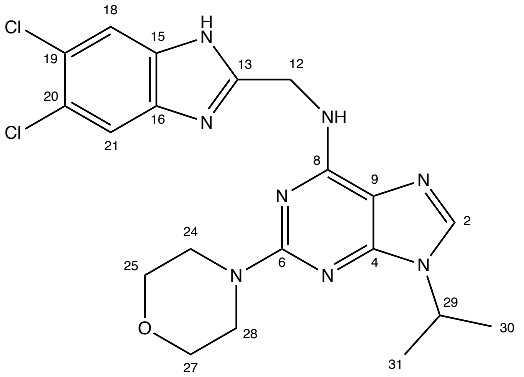


**DS18 (65)**

Derivative DS18 was prepared as per procedures B and D. UHPLC purification was performed at a detection wavelength of 210 nm. $^1$H NMR (600 MHz, $CD_3CN$): δ 9.48 (s, 1H, $H_2$), 8.46 (s, NH), 7.76 (s, 2H, $H_{18}$, $H_{21}$), 5.05 (d, $J$ = 5.5 Hz, 2H, $H_{12}$), 4.75 (m, 1H, $H_{29}$), 3.59 (m, 4H, $H_{24}$, $H_{28}$), 3.52 (m, 4H, $H_{25}$, $H_{27}$), 1.55 (d, $J$ = 6.8 Hz, 6H, $H_{30}$, $H_{31}$). $^{13}$C NMR (151 MHz, $CD_3CN$): δ 156.5 ($C_8$), 152.4 ($C_4$), 151.3 ($C_6$), 142.1 ($C_{13}$), 135.8 ($C_{15}/C_{16}$), 135.3 ($C_{15}/C_{16}$), 128.3 ($C_{19}$, $C_{20}$), 116.9 ($C_{18}$, $C_{21}$), 116.4 ($C_9$), 67.1 ($C_{25}$, $C_{27}$), 50.2 ($C_{24}$, $C_{28}$, $C_{29}$), 45.5 ($C_{12}$), 38.9 (n/a), 21.7 ($C_{30}$, $C_{31}$). HRMS (m/z): $[M+H]^+$ calcd. 461.1366, found 461.1372.

**9-isopropyl-2-morpholino-_N_-(4-(pyridin-2-yl)benzyl)-9_H_-purin-6-amine (DS19)**

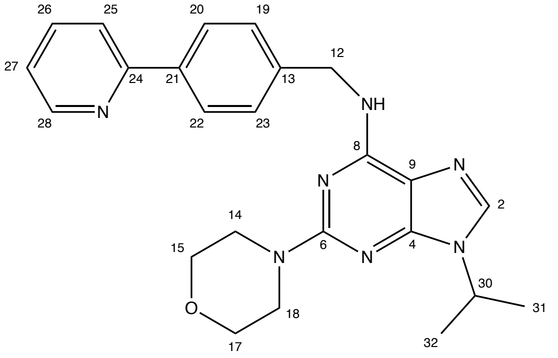


**DS19 (44)**

Derivative DS-19 was prepared as per procedures B and D. UHPLC purification was performed at a detection wavelength of 240 nm. $^1$H NMR (600 MHz, $CD_3CN$): δ 9.35 (s, NH), 8.75 (m, 1H, $H_{28}$), 8.48 (s, 1H, $H_2$), 8.10 (m, 1H, $H_{20}$), 7.96 (m, 3H, $H_{19}$, $H_{22}$, $H_{23}$), 7.55 (m, 3H, $H_{25}$, $H_{26}$, $H_{27}$), 4.80 (d, $J$ = 5.9

Hz, 2H, $H_{12}$), 4.77 (m, 1H, $H_{30}$), 3.72 (t, $J$ = 4.6 Hz, 4H, $H_{14}$, $H_{18}$), 3.63 (t, $J$ = 4.8 Hz, 4H, $H_{15}$, $H_{17}$), 1.56 (d, $J$ = 6.8 Hz, 6H, $H_{31}$, $H_{32}$). $^{13}$C NMR (151 MHz, $CD_3CN$): δ 161.0 ($C_6$), 155.8 ($C_{24}$), 152.6 ($C_4$), 151.1 (n/a), 147.4 ($C_{28}$), 143.2 (n/a), 141.9 ($C_{13}$), 135.6 ($C_{26}$), 135.0 ($C_{21}$), 129.1 ($C_{19}$), 128.4 ($C_{23}$), 124.5 ($C_{20}$, $C_{22}$), 123.6 ($C_{27}$), 120.5 ($C_{25}$), 116.7 ($C_9$), 105.9 (imp), 67.2 ($C_{15}$, $C_{17}$), 50.0 ($C_{14}$, $C_{18}$), 49.9 ($C_{30}$), 45.6 (imp), 44.4 ($C_{12}$), 21.8 ($C_{31}$, $C_{32}$). HRMS (m/z): $[M+H]^+$ calcd. 430.2350, found 430.2351.

**(*R*)-2-((9-(1-methyl-1*H*-pyrazol-4-yl)-6-((4-(pyridin-2-yl)benzyl)amino)-9*H*-purin-2-yl)amino)butan-1-ol (DS20)**

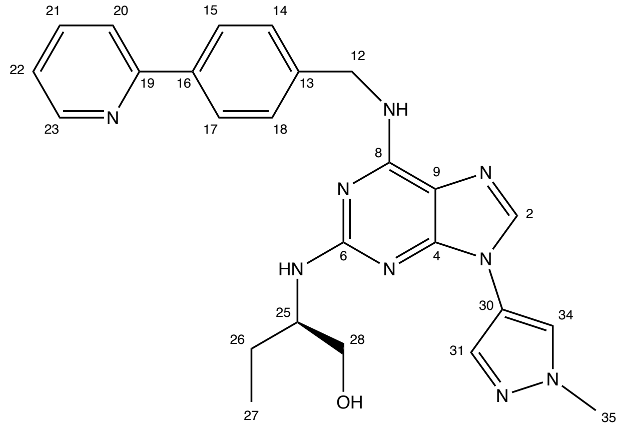


**DS20 (69)**

Derivative DS20 was prepared as per procedures C and D. A modified version of procedure C was used. Product from procedure A (1.0 eq), copper (I) bromide (0.3 eq), sodium ascorbate (0.6 eq), and potassium hydroxide (3.0 eq) were added to a round-bottomed flask. 2,2'-((1,10-phenanthroline-4,7-diyl)bis(azanediyl))bis(ethan-1-ol) was synthesized using a previously published protocol and was added (0.1 eq) as a catalyst to increase reaction efficiency.[3] Afterwards, 4-bromo-1-methyl-1H-pyrazole (1.8 eq) was mixed with DMF and added to the flask (generally a 150 mM solution of product from procedure A is used). The reaction proceeded at 140 °C overnight (~16 h). Water was added to quench the reaction and multiple EtOAc extractions were performed. The organic layers were combined, concentrated, and dried to give the desired crude product, which then underwent UHPLC purification (detection wavelength = 275 nm; gradient was 1-60% ACN in 29 min at a flow rate of 20 mL/min). UHPLC purification of the final product post procedure D was performed at a detection wavelength of 230 nm. $^1$H NMR (600 MHz, $CD_3CN$): δ 8.78 (d, $J$ = 5.0 Hz, 1H, $H_{23}$), 8.28 (t, $J$ = 7.8 Hz, 1H, $H_{21}$), 8.09 (d, $J$ = 8.2, 1H, $H_{20}$), 8.05 (s, 1H, $H_{31}$), 7.97 (d, $J$ = 8.0 Hz, 3H, $H_{15}$, $H_{17}$, $H_{34}$), 7.85 (s, 1H, $H_2$), 7.70 (t, $J$ = 6.6 Hz, 1H, $H_{22}$), 7.63 (d, $J$ = 8.1 Hz, 2H, $H_{14}$, $H_{18}$), 6.58 (m, 2H, $H_{20}$, $H_{22}$), 5.40 (s, 2H, $H_{12}$) 3.99 (m, 1H, $H_{25}$), 3.92 (s, 3H, $H_{35}$), 3.63 (m, 2H, $H_{28}$), 0.96 (m, 3H, $H_{27}$). $^{13}$C NMR (151MHz, $CD_3CN$): δ 128.4 ($C_{14}$, $C_{18}$), 128.1 ($C_{15}$, $C_{17}$, $C_{34}$), 124.4 ($C_{22}$), 124.0 ($C_{20}$), 63.0 ($C_{28}$), 54.9 ($C_{25}$), 46.5 ($C_{12}$), 39.1 ($C_{35}$) 23.0 ($C_{26}$), 10.8 ($C_{27}$). HRMS (m/z): $[M+H]^+$ calcd. 470.2411, found 470.2402.

**9-(1-methyl-1*H*-pyrazol-4-yl)-2-morpholino-*N*-(4-(pyridin-2-yl)benzyl)-9*H*-purin-6-amine (DS21)**

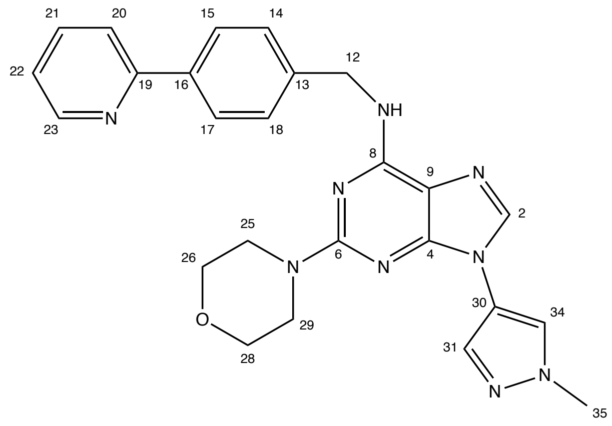

**DS21 (68)**

Derivative DS21 was prepared as per procedures C and D. A modified version of procedure C was used. Product from procedure A (1.0 eq), copper (I) bromide (0.3 eq), sodium ascorbate (0.6 eq), and potassium hydroxide (3.0 eq) were added to a round-bottomed flask. 2,2'-((1,10-phenanthroline-4,7-diyl)bis(azanediyl))bis(ethan-1-ol) was synthesized using a previously published protocol and was added (0.1 eq) as a catalyst to increase reaction efficiency.[3] Afterwards, 4-bromo-1-methyl-1H-pyrazole (1.8 eq) was mixed with DMF and added to the flask (generally a 150 mM solution of product from procedure A is used). The reaction proceeded at 140 °C overnight (~16 h). Water was added to quench the reaction and multiple EtOAc extractions were performed. The organic layers were combined, concentrated, and dried to give the desired crude product, which then underwent UHPLC purification (detection wavelength = 275 nm; gradient was 1-60% ACN in 29 min at a flow rate of 20 mL/min). UHPLC purification of the final product post procedure D was performed at a detection wavelength of 230 nm. $^1$H NMR (600 MHz, $CD_3CN$): 8.72 (d, *J* = 5.2 Hz, 1H, $H_{23}$), 8.17 (s, 1H, $H_{31}$), 8.11 (s, 1H, $H_{15}$), 8.07 (t, *J* = 7.8 Hz, 1H, $H_{17}$), 7.96 (m, 3H, $H_{14}$, $H_{18}$, $H_{34}$), 7.86 (s, 1H, $H_2$), 7.57 (d, 2H, $H_{20}$, $H_{21}$), 7.51 (t, *J* = 6.1 Hz, 1H, $H_{22}$), 4.81 (s, 2H, $H_{12}$), 3.92 (s, 3H, $H_{35}$), 3.72 (m, 4H, $H_{25}$, $H_{29}$), 3.65 (m, 4H, $H_{26}$, $H_{28}$). $^{13}$C NMR (151 MHz, $CD_3CN$): δ 132.4 ($C_2$), 129.1 ($C_{13}$), 128.2 ($C_{20}$, $C_{21}$), 127.4 ($C_{34}$), 123.8 ($C_{15}$, $C_{17}$), 123.4 ($C_{22}$), 122.3 ($C_{14}$, $C_{18}$), 66.4 ($C_{26}$, $C_{28}$), 44.9 ($C_{25}$, $C_{29}$), 43.4 ($C_{12}$), 40.1 ($C_{35}$). HRMS (m/z): $[M+H]^+$ calcd. 468.2255, found 468.2248.

**(*R*)-2-((6-(((5,6-dichloro-1*H*-benzo[*d*]imidazol-2-yl)methyl)amino)-9-(1-methyl-1*H*-pyrazol-4-yl)-9*H*-purin-2-yl)amino)butan-1-ol (DS22)**

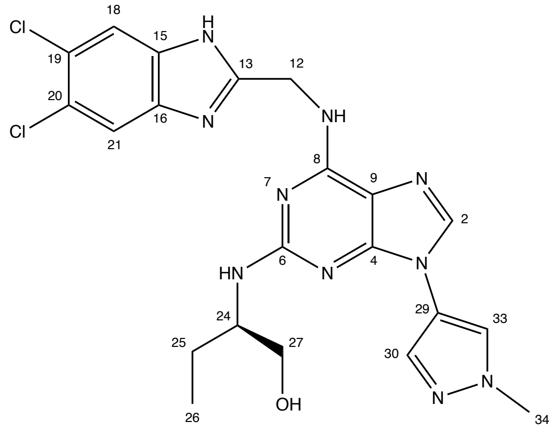

**DS22 (67)**

Derivative DS22 was commercially synthesized by Wuxi AppTec (Shanghai, China). The final compound was isolated as a free base. $^{1}$H NMR (600 MHz, $(CD_3)_2SO$): δ 8.28 (s, 1H, $H_2$), 8.09 (s, 1H, imp), 7.98 (s, 2H, $H_{18}$, $H_{21}$), 7.71 (s, 2H, $H_{30}$, $H_{33}$), 6.15 (s, broad, NH), 4.83 (s, 2H, $H_{12}$), 3.90 (s, 3H, $H_{34}$), 3.78 (m, 1H, $H_{24}$), 1.52 (m, 2H, $H_{25}$), 0.80 (m, 3H, $H_{26}$). $^{13}$C NMR (151 MHz, $(CD_3)_2SO$): δ 159.7 ($C_6$), 156.6 ($C_8$), 154.4 ($C_4$), 135.5 (imp), 130.7 ($C_{18}$, $C_{21}$), 123.5 ($C_2$), 118.8 ($C_{29}$), 113.4 ($C_9$), 63.1 ($C_{27}$), 54.2 ($C_{24}$), 40.0 ($C_{12}$, $C_{34}$), 23.8 ($C_{25}$), 10.7 ($C_{26}$). HRMS (m/z): $[M+Na]^+$ calcd. 523.1247, found 523.1246.

**(*R*)-2-((9-isopropyl-6-(methylamino)-9*H*-purin-2-yl)amino)butan-1-ol (DS23)**

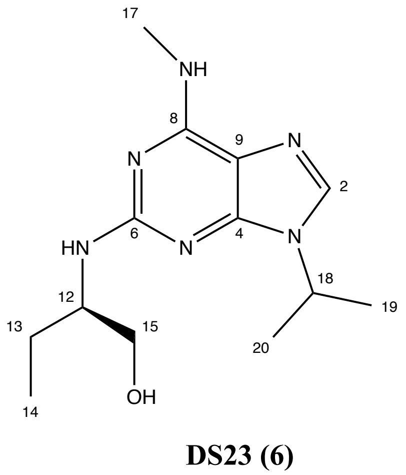

**DS23 (6)**

Derivative DS23 was prepared as per procedures B and D. UHPLC purification was performed at a detection wavelength of 225 nm. $^{1}$H NMR (600 MHz, $CD_3CN$): δ 7.86 (m, 1H, $H_2$), 6.90 (s, NH), 4.65 (m, 1H, $H_{18}$), 3.99 (m, 1H, $H_{12}$), 3.60 (m, 2H, $H_{15}$), 3.46 (s, 2H, imp), 3.10 (s, 3H, $H_{17}$), 1.65 (m, 2H, $H_{13}$), 1.52 (d, $J = 6.8$ Hz, 6H, $H_{19}$, $H_{20}$), 0.96 (t, $J = 7.5$ Hz, 3H, $H_{14}$). $^{13}$C NMR (151 MHz, $CD_3CN$): δ

152.5 ($C_4$), 139.6 ($C_2$), 116.2 ($C_9$), 64.1 ($C_{15}$), 56.1 ($C_{12}$), 49.3 ($C_{18}$), 31.6 ($C_{17}$), 24.7 ($C_{13}$), 22.2 ($C_{19}$, $C_{20}$), 10.8 ($C_{14}$). HRMS (m/z): $[M+H]^+$ calcd. 279.1928, found 279.1932.

**(*R*)-2-((6-((3-fluoro-4-(pyridin-2-yl)benzyl)amino)-9-isopropyl-9*H*-purin-2-yl)amino)butan-1-ol (DS24)**

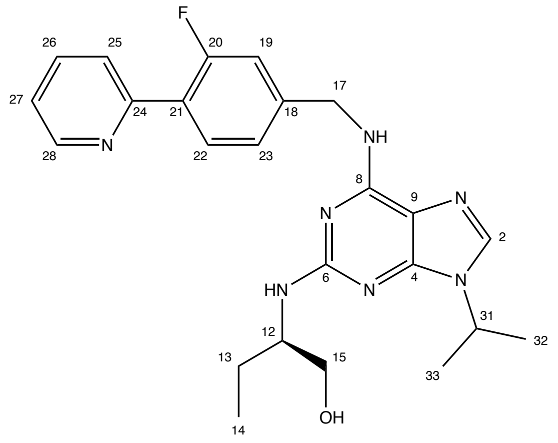


**DS24 (42)**

Derivative DS24 was prepared as per procedures B and D. UHPLC purification was performed at a detection wavelength of 305 nm. $^1$H NMR (600 MHz, $CD_3CN$): δ 8.83 (m, 1H, $H_{28}$), 8.36 (m, 1H, $H_{22}$), 7.79 (m, 3H, $H_{19}$, $H_{23}$, $H_{26}$), 7.42 (d, 1H, $H_{25}$), 7.38 (s, 1H, $H_2$), 7.29 (m, 1H, $H_{27}$), 6.81 (s, NH), 4.85 (m, 2H, $H_{17}$), 4.68 (s, 1H, $H_{31}$), 3.97 (m, 1H, $H_{12}$), 3.61 (m, 2H, $H_{15}$), 1.62 (m, 2H, $H_{13}$), 1.53 (d, $J$ = 6.7 Hz, 6H, $H_{32}$, $H_{33}$), 0.96 (t, $J$ = 7.5 Hz, 3H, $H_{14}$). $^{13}$C NMR (151 MHz, $CD_3CN$): δ 157.5 ($C_8$), 150.2 ($C_6$), 147.9 ($C_{28}$), 145.7 ($C_{20}$), 145.3 ($C_{24}$), 144.8 ($C_{18}$), 136.3 ($C_{26}$), 132.1 ($C_{22}$), 128.1 ($C_{21}$/$C_{23}$), 127.9 ($C_{21}$/$C_{23}$), 126.0 ($C_{19}$), 124.9 ($C_{25}$), 124.6 ($C_{27}$), 116.1 ($C_9$), 68.9 (imp), 64.1 ($C_{15}$), 55.7 ($C_{12}$), 49.7 ($C_{31}$), 44.3 ($C_{17}$), 40.8 (imp), 24.7 ($C_{13}$), 22.2 ($C_{32}$, $C_{33}$), 10.8 ($C_{14}$). HRMS (m/z): $[M+H]^+$ calcd. 450.2412, found 450.2410.

**(*R*)-2-((9-isopropyl-6-((4-(5-methylthiophen-2-yl)benzyl)amino)-9*H*-purin-2-yl)amino)butan-1-ol (DS25)**

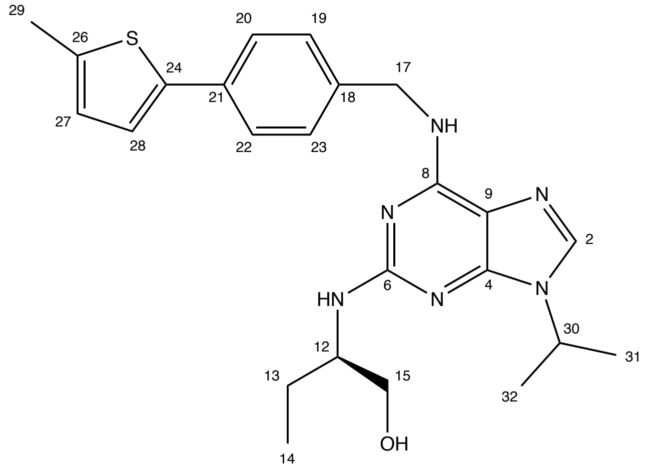

**DS25 (25)**

Derivative DS25 was prepared as per procedures B and D. UHPLC purification was performed at a detection wavelength of 210 nm. The gradient was 1-70% ACN in 16 min at a flow rate of 20 mL/min. $^{1}$H NMR (600 MHz, $CD_3CN$): δ 7.83 (s, 1H, $H_2$), 7.53 (m, 2H, $H_{20}$, $H_{22}$), 7.39 (m, 2H, $H_{19}$, $H_{23}$), 7.17 (m, 1H, $H_{28}$), 6.76 (m, 1H, $H_{27}$), 4.76 (s, 2H, $H_{17}$), 4.66 (s, 1H, $H_{30}$), 3.98 (s, 1H, $H_{12}$), 2.47 (s, 3H, $H_{29}$), 1.65 (m, 2H, $H_{13}$), 1.51 (d, $J$ = 6.8 Hz, 6H, $H_{31}$, $H_{32}$), 0.95 (m, 3H, $H_{14}$). $^{13}$C NMR (151 MHz, $CD_3CN$): δ 158.0 ($C_6$), 142.1 ($C_{18}$), 140.8 ($C_{24}$), 139.8 ($C_{26}$), 139.4 ($C_{21}$), 137.8 ($C_2$), 129.2 ($C_{20}$, $C_{22}$), 128.7 ($C_{19}/C_{23}$), 127.6 ($C_{19}/C_{23}$), 126.3 ($C_{27}$), 124.3 ($C_{28}$), 116.6 ($C_9$), 64.1 ($C_{15}$), 55.9 ($C_{12}$), 49.5 ($C_{30}$), 44.7 ($C_{17}$), 24.7 ($C_{13}$), 22.2 ($C_{31}$, $C_{32}$), 15.4 ($C_{29}$), 10.8 ($C_{14}$). HRMS (m/z): $[M+H]^+$ calcd. 451.2275, found 451.2281.

**(*R*)-2-((9-isopropyl-6-((3-(pyridin-4-yl)propyl)amino)-9*H*-purin-2-yl)amino)butan-1-ol (DS27)**

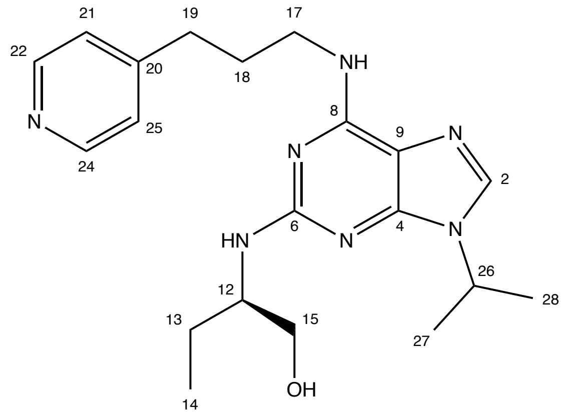

**DS27 (30)**

Derivative DS-27 was prepared as per procedures B and D. UHPLC purification was performed at a detection wavelength of 250 nm. $^{1}$H NMR (600 MHz, $CD_3CN$): δ 8.58 (d, $J$ = 6.7 Hz, 2H, $H_{22}$, $H_{24}$), 7.84 (d, $J$ = 6.0 Hz, 2H, $H_{21}$, $H_{25}$), 7.80 (s, 1H, $H_2$), 6.70 (m, 1H, imp), 4.65 (m, 1H, $H_{26}$), 4.01 (m, 3H,

$H_{12}$, $H_{17}$), 3.62 (m, 4H, $H_{15}$, $H_{19}$), 2.10 (t, $J$ = 7.3 Hz, 2H, $H_{18}$), 1.67 (m, 2H, $H_{13}$), 1.52 (m, 6H, $H_{27}$, $H_{28}$), 0.96 (m, 3H, $H_{14}$). $^{13}$C NMR (151 MHz, $CD_3CN$): δ 155.7 ($C_8$), 152.5 ($C_4$), 150.4 ($C_6$), 142.0 ($C_{20}$), 139.8 ($C_2$), 128.1 ($C_{22}$, $C_{24}$), 120.1 ($C_{21}$, $C_{25}$), 116.2 ($C_9$), 64.2 ($C_{15}$), 61.1 (imp), 56.4 (imp), 55.7 ($C_{12}$), 49.5 ($C_{26}$), 43.8 ($C_{17}$), 40.9 (imp), 33.4 (imp), 30.5 ($C_{19}$), 29.8 ($C_{18}$), 24.8 ($C_{13}$), 22.2 ($C_{27}$, $C_{28}$), 10.8 ($C_{14}$). HRMS (m/z): $[M+H]^+$ calcd. 384.2506, found 384.2504.

**(*S*)-2-((9-isopropyl-6-((4-(pyridin-2-yl)benzyl)amino)-9*H*-purin-2-yl)amino)butan-1-ol (DS28)**

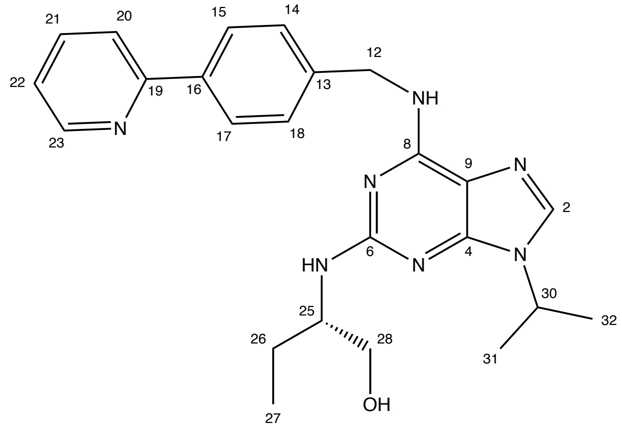


**DS28 (43)**

Derivative DS28 was prepared as per procedures B and D. UHPLC purification was performed at a detection wavelength of 290 nm. $^1$H NMR (600 MHz, $CD_3CN$): δ 8.82 (m, 1H, $H_{23}$), 8.34 (m, 1H, $H_{15}/H_{17}$), 8.10 (m, 1H, $H_{15}/H_{17}$), 7.92 (d, $J$ = 7.8 Hz, 2H, $H_{14}$, $H_{18}$), 7.74 (t, $J$ = 6.6 Hz, 1H, $H_{21}$), 7.62 (d, $J$ = 8.0 Hz, 1H, $H_{20}$, $H_{22}$), 4.86 (s, 2H, $H_{12}$), 4.66 (m, 1H, $H_{30}$), 3.98 (m, 1H, $H_{25}$), 1.61 (m, 2H, $H_{26}$), 1.52 (d, $J$ = 6.8 Hz, 6H, $H_{31}$, $H_{32}$), 0.96 (t, $J$ = 7.5 Hz, 3H, $H_{27}$). $^{13}$C NMR (151 MHz, $CD_3CN$): δ 154.5 ($C_4$), 145.0 ($C_{19}$), 136.7 ($C_{23}$), 136.2 ($C_{21}$), 134.8 ($C_{16}$), 129.2 ($C_{13}$), 128.8 ($C_{14}$), 127.0 ($C_{18}$), 125.5 ($C_{15}$, $C_{17}$), 125.4 ($C_{20}$), 120.0 ($C_{22}$), 116.2 ($C_9$), 68.8 (imp), 64.6 ($C_{28}$), 61.7 (imp), 56.5 ($C_{25}$), 49.5 ($C_{30}$), 44.5 ($C_{12}$), 41.0 (imp), 24.7 ($C_{26}$), 22.2 ($C_{31}$, $C_{32}$), 10.8 ($C_{27}$). HRMS (m/z): $[M+H]^+$ calcd. 432.2506, found 432.2503.

**(*R*)-2-((6-((3-(3-fluorophenyl)propyl)amino)-9-isopropyl-9*H*-purin-2-yl)amino)butan-1-ol (DS29)**

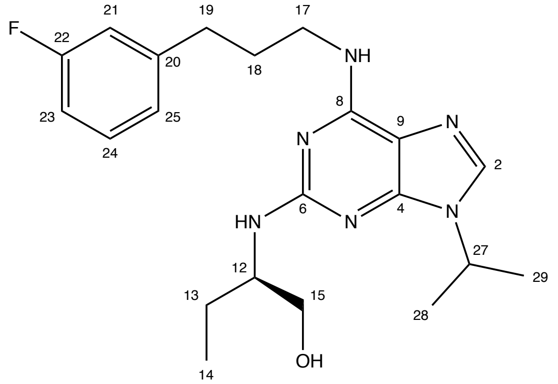


**DS29 (36)**

Derivative DS29 was prepared as per procedures B and D. UHPLC purification was performed at a detection wavelength of 210 nm. $^{1}$H NMR (600 MHz, $CD_3CN$): δ 7.80 (s, 1H, $H_2$), 7.26 (m, 1H, $H_{24}$), 7.04 (s, 1H, $H_{23}$), 6.98 (s, 1H, $H_{25}$), 6.91 (td, $J$ = 6.8, 2.6 Hz, 1H, $H_{21}$), 6.74 (s, broad, NH), 4.64 (m, 1H, $H_{27}$), 3.98 (m, 3H, $H_{12}$, $H_{17}$), 3.58 (m, 2H, $H_{15}$), 1.98 (m, 2H, $H_{18}$), 1.60 (m, 2H, $H_{13}$), 1.51 (d, $J$ = 6.8 Hz, 6H, $H_{28}$, $H_{29}$), 0.95 (m, 3H, $H_{14}$). $^{13}$C NMR (151 MHz, $CD_3CN$): δ 164.6 (n/a), 163.0 ($C_{22}$), 152.5 ($C_4$), 150.4 ($C_6$), 145.6 ($C_{20}$), 139.6 ($C_2$), 131.0 ($C_{24}$), 125.4 ($C_{25}$), 116.1 ($C_9$), 113.5 ($C_{23}$), 64.2 ($C_{15}$), 55.8 ($C_{12}$), 49.2 ($C_{27}$), 44.2 ($C_{17}$), 33.3 (imp), 31.9 ($C_{19}$), 31.2 ($C_{18}$), 24.8 ($C_{13}$), 22.2 ($C_{28}$, $C_{29}$), 10.8 ($C_{14}$). HRMS (m/z): $[M+H]^+$ calcd. 401.2460, found 401.2464.

**(*R*)-2-((9-isopropyl-6-((3-(*m*-tolyl)propyl)amino)-9*H*-purin-2-yl)amino)butan-1-ol (DS30)**

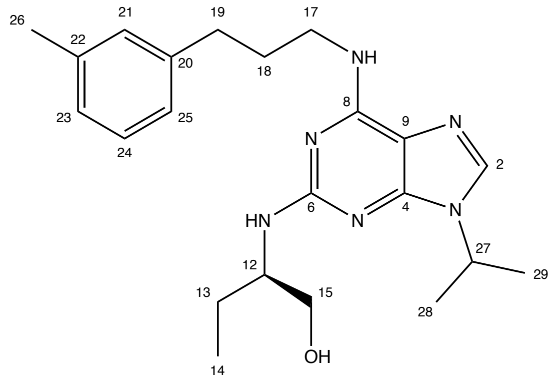


**DS30 (34)**

Derivative DS30 was prepared as per procedures B and D. UHPLC purification was performed at a detection wavelength of 210 nm. The gradient was 1-70% ACN in 16 min at a flow rate of 20 mL/min. $^{1}$H NMR (600 MHz, $CD_3CN$): δ 7.80 (s, 1H, $H_2$), 7.13 (s, 1H, $H_{24}$), 7.00 (m, 3H, $H_{21}$, $H_{23}$, $H_{25}$), 6.60 (s, NH), 4.64 (m, 1H, $H_{27}$), 3.97 (m, 3H, $H_{12}$, $H_{17}$), 3.59 (m, 2H, $H_{15}$), 2.67 (m, 2H, $H_{19}$), 2.26 (m, 3H, $H_{26}$), 1.97 (m, 2H, $H_{18}$), 1.62 (m, 2H, $H_{13}$), 1.52 (d, $J$ = 6.7 Hz, 6H, $H_{28}$, $H_{29}$), 0.96 (m, 3H, $H_{14}$). $^{13}$C NMR (151 MHz, $CD_3CN$): δ 152.5 ($C_4$), 142.5 ($C_{20}$), 139.6 ($C_2$), 138.9 ($C_{22}$), 130.1 ($C_{21}$), 129.2 ($C_{24}$), 127.5

($C_{23}$), 126.3 ($C_{25}$), 116.1 ($C_9$), 64.1 ($C_{15}$), 55.7 ($C_{12}$), 49.5 ($C_{27}$), 44.3 ($C_{17}$), 33.3 (imp), 32.1 ($C_{19}$), 31.4 ($C_{18}$), 24.8 ($C_{13}$), 22.2 ($C_{28}$, $C_{29}$), 21.4 ($C_{26}$), 10.8 ($C_{14}$). HRMS (m/z): $[M+H]^+$ calcd. 397.2710, found 397.2716.

**(*R*)-2-((9-isopropyl-6-(phenethylamino)-9*H*-purin-2-yl)amino)butan-1-ol (DS31)**

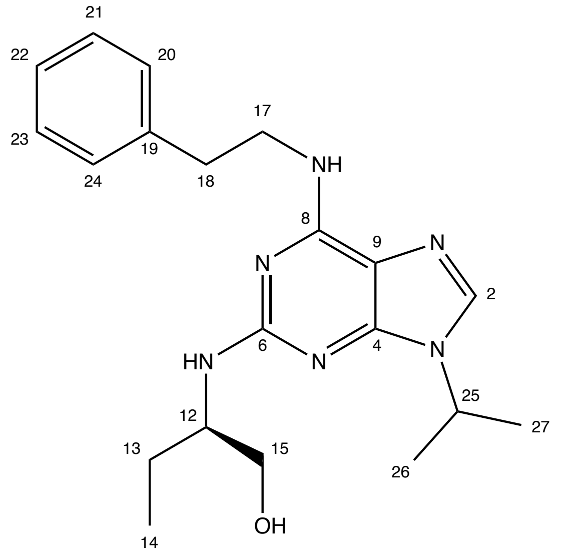


**DS31 (16)**

Derivative DS31 was prepared as per procedures B and D. UHPLC purification was performed at a detection wavelength of 220 nm. $^1$H NMR (600 MHz, $CD_3CN$): δ 7.84 (s, 1H, $H_2$), 7.30 (m, 4H, $H_{20}$, $H_{21}$, $H_{23}$, $H_{24}$), 7.22 (m, 1H, $H_{22}$), 6.81 (s, NH), 4.64 (s, 1H, $H_{25}$), 4.21 (s, 1H, imp), 3.98 (m, 1H, $H_{12}$), 3.82 (s, 2H, $H_{17}$), 3.61 (m, 2H, $H_{15}$), 2.98 (t, $J$ = 7.2 Hz, 2H, $H_{18}$), 1.62 (m, 2H, $H_{13}$), 1.52 (d, $J$ = 6.8 Hz, 6H, $H_{26}$, $H_{27}$), 0.96 (m, 3H, $H_{14}$). $^{13}$C NMR (151 MHz, $CD_3CN$): δ 152.6 ($C_4$), 139.8 ($C_{19}$), 139.4 ($C_2$), 129.9 ($C_{21}$, $C_{23}$), 128.5 ($C_{20}$, $C_{24}$), 127.5 ($C_{22}$), 116.0 ($C_9$), 64.1 ($C_{15}$), 55.8 ($C_{12}$), 49.5 ($C_{25}$), 46.1 ($C_{17}$), 36.6 (n/a), 35.8 ($C_{18}$), 24.7 ($C_{13}$), 22.2 ($C_{26}$, $C_{27}$), 10.9 ($C_{14}$). HRMS (m/z): $[M+H]^+$ calcd. 369.2397, found 369.2404.

**(*R*)-2-((6-((3-cyclohexylpropyl)amino)-9-isopropyl-9*H*-purin-2-yl)amino)butan-1-ol (DS32)**

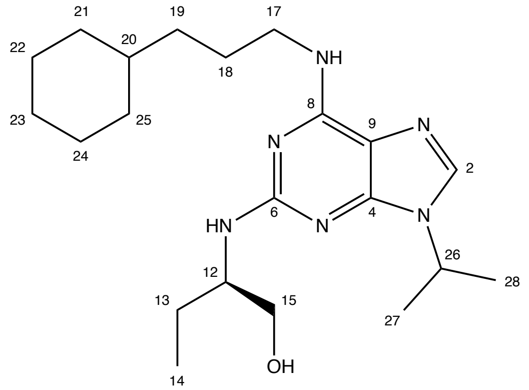

**DS32 (13)**

Derivative DS32 was prepared as per procedures B and D. UHPLC purification was performed at a detection wavelength of 220 nm. The gradient was 1-70% ACN in 16 min at a flow rate of 20 mL/min. $^1$H NMR (600 MHz, $CD_3CN$): δ 7.80 (s, 1H, $H_2$), 6.70 (s, NH), 4.63 (m, 1H, $H_{26}$), 3.96 (m, 3H, $H_{12}$, $H_{17}$), 3.61 (m, 2H, $H_{15}$), 1.68 (m, 9H, $H_{18}$, $H_{20}$, $H_{22}$, $H_{23}$, $H_{24}$), 1.60 (m, 2H, $H_{13}$), 1.51, (d, $J$ = 7.1 Hz, 6H, $H_{27}$, $H_{28}$), 1.25 (m, 4H, $H_{21}$, $H_{25}$), 1.18 (m, 2H, $H_{19}$), 0.96 (t, $J$ = 7.5 Hz, 3H, $H_{14}$), 0.88 (m, 3H, imp). $^{13}$C NMR (151 MHz, $CD_3CN$): δ 152.5 ($C_4$), 139.5 ($C_2$), 116.4 ($C_9$), 64.0 ($C_{15}$), 55.7 ($C_{12}$), 49.2 ($C_{26}$), 45.0 ($C_{17}$), 38.1 (n/a), 34.8 ($C_{19}$), 34.0 ($C_{21}$, $C_{25}$), 27.8 ($C_{18}$), 27.4 ($C_{23}$), 27.1 ($C_{22}$, $C_{24}$), 24.7 ($C_{13}$), 22.2 ($C_{27}$, $C_{28}$), 10.8 ($C_{14}$). HRMS (m/z): $[M+H]^+$ calcd. 389.3023, found 389.3023.

**(*R*)-2-((6-((3-cyclopentylpropyl)amino)-9-isopropyl-9*H*-purin-2-yl)amino)butan-1-ol (DS33)**

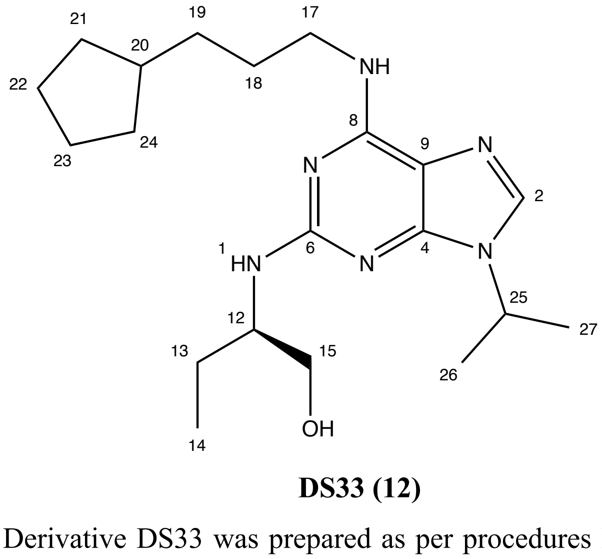

**DS33 (12)**

Derivative DS33 was prepared as per procedures B and D. UHPLC purification was performed at a detection wavelength of 220 nm. The gradient was 1-70% ACN in 16 min at a flow rate of 20 mL/min. $^1$H NMR (600 MHz, $CD_3CN$): δ 7.81 (s, 1H, $H_2$), 4.64 (m, 1H, $H_{25}$), 3.97 (m, 3H, $H_{12}$, $H_{17}$), 1.76 (m, 2H, $H_{21}$/$H_{24}$), 1.68 (m, 2H, $H_{21}$/$H_{24}$), 1.59 (m, 2H, $H_{22}$/$H_{23}$), 1.51 (d, $J$ = 7.3 Hz, 6H, $H_{26}$, $H_{27}$), 1.39 (m, 2H, $H_{18}$), 0.95 (t, $J$ = 7.5 Hz, 3H, $H_{14}$). $^{13}$C NMR (151 MHz, $CD_3CN$): δ 152.5 ($C_4$), 150.3 ($C_6$), 139.6

($C_2$), 116.0 ($C_9$), 64.0 ($C_{15}$), 55.8 ($C_{12}$), 49.2 ($C_{25}$), 40.6 ($C_{17}$), 33.8 ($C_{20}$), 33.6 ($C_{19}$), 33.3 ($C_{21}$, $C_{24}$), 29.8 ($C_{18}$), 25.8 ($C_{22}$, $C_{23}$), 24.7 ($C_{13}$), 22.2 ($C_{26}$, $C_{27}$), 10.8 ($C_{14}$). HRMS (m/z): $[M+H]^+$ calcd. 375.2867, found 375.2874.

**(*R*)-2-((6-((3-(furan-2-yl)propyl)amino)-9-isopropyl-9*H*-purin-2-yl)amino)butan-1-ol (DS34)**

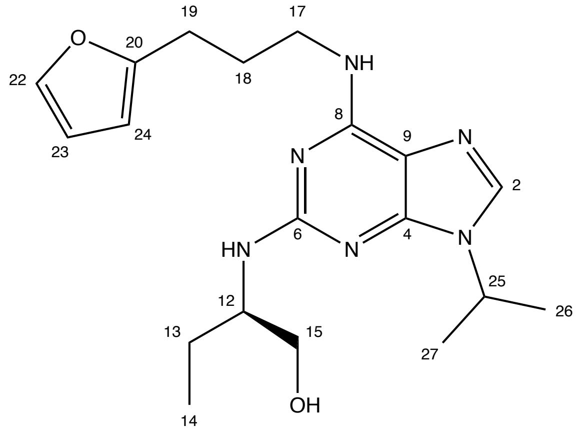


**DS34 (29)**

Derivative DS34 was prepared as per procedures B and D. UHPLC purification was performed at a detection wavelength of 250 nm. $^{1}H$ NMR (600 MHz, $CD_3CN$): δ 7.81 (s, 1H, $H_2$), 7.35 (s, 1H, $H_{22}$), 6.83 (s, NH), 6.29 (s, 1H, $H_{23}$), 6.07 (d, $J$ = 2.7 Hz, 1H, $H_{24}$), 4.64 (m 1H, $H_{25}$), 3.99 (m, 3H, $H_{12}$, $H_{17}$), 3.61 (m, 2H, $H_{15}$), 2.73 (t, $J$ = 7.7 Hz, 2H, $H_{19}$), 1.99 (m, 2H, $H_{18}$), 1.64 (m, 2H, $H_{13}$), 1.51 (d, $J$ = 6.9 Hz, 6H, $H_{26}$, $H_{27}$), 0.95 (t, $J$ = 7.5 Hz, 3H, $H_{14}$). $^{13}C$ NMR (151 MHz, $CD_3CN$): δ 156.1 ($C_{20}$), 152.5 ($C_4$), 150.3 ($C_6$), 142.2 ($C_{22}$), 139.7 ($C_2$), 116.1 ($C_9$), 111.2 ($C_{23}$), 106.2 ($C_{24}$), 64.1 ($C_{15}$), 55.9 ($C_{12}$), 49.4 ($C_{25}$), 44.1 ($C_{17}$), 29.0 (n/a), 28.3 ($C_{18}$), 25.7 ($C_{19}$), 24.8 ($C_{13}$), 22.2 ($C_{26}$, $C_{27}$), 10.9 ($C_{14}$). HRMS (m/z): $[M+H]^+$ calcd. 373.2347, found 373.2352.

**(*R*)-2-((9-isopropyl-6-((3-(1-methyl-1*H*-pyrazol-5-yl)propyl)amino)-9*H*-purin-2-yl)amino)butan-1-ol (DS35)**

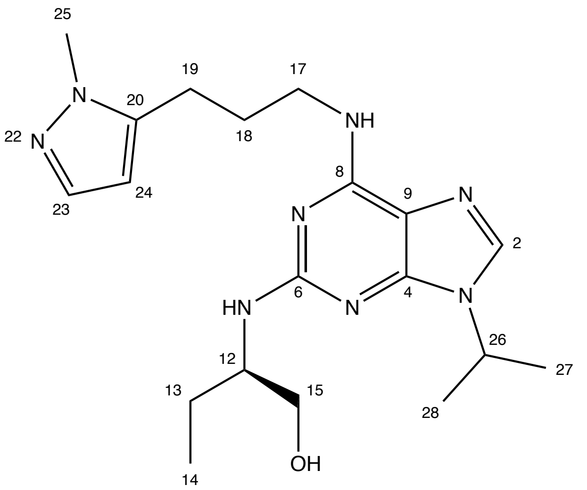

**DS35 (28)**

Derivative DS35 was prepared as per procedures B and D. UHPLC purification was performed at a detection wavelength of 230 nm. $^{1}$H NMR (600 MHz, $CD_3CN$): δ 7.82 (s, 1H, $H_2$), 7.41 (s, 1H, $H_{24}$), 7.39 (s, 1H, $H_{23}$), 6.69 (s, NH), 4.65 (m, 1H, $H_{26}$), 3.97 (m, 3H, $H_{12}$, $H_{17}$), 3.82 (s, 3H, $H_{25}$), 2.56 (m, 2H, $H_{19}$), 1.92 (m, 2H, $H_{18}$), 1.63 (m, 2H, $H_{13}$), 1.52 (d, $J$ = 6.6 Hz, 6H, $H_{27}$, $H_{28}$), 0.95 (t, $J$ = 7.5 Hz, 3H, $H_{14}$). $^{13}$C NMR (151 MHz, $CD_3CN$): δ 152.4 ($C_4$), 150.3 ($C_6$), 139.6 ($C_{20}$), 138.0 ($C_{23}$), 137.7 ($C_2$) 131.0 ($C_{24}$), 121.9 (imp), 115.8 ($C_9$), 64.1 ($C_{15}$), 55.8 ($C_{12}$), 49.5 ($C_{26}$), 44.0 ($C_{17}$), 38.9 ($C_{25}$), 31.5 ($C_{18}$), 30.8 ($C_{19}$), 24.7 ($C_{13}$), 22.2 ($C_{27}$, $C_{28}$), 10.8 ($C_{14}$). HRMS (m/z): $[M+H]^+$ calcd. 387.2615, found 387.2620.

**(*R*)-2-((6-((3-(dibutylamino)propyl)amino)-9-isopropyl-9*H*-purin-2-yl)amino)butan-1-ol (DS36)**

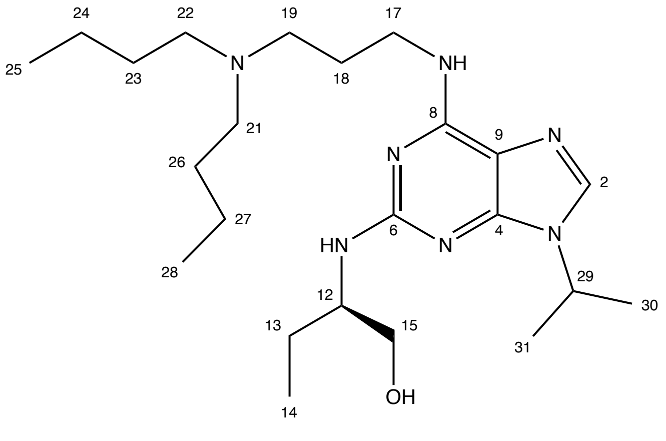

**DS36 (9)**

Derivative DS36 was prepared as per procedures B and D. UHPLC purification was performed at a detection wavelength of 225 nm. $^{1}$H NMR (600 MHz, $CD_3CN$): δ 7.90 (s, 1H, $H_2$), 6.76 (s, NH), 4.69 (m, 1H, $H_{29}$), 4.00 (m, 3H, $H_{12}$, $H_{17}$), 3.20 (m, 2H, $H_{19}$), 3.06 (m, 4H, $H_{21}$, $H_{22}$), 2.09 (p, $J$ = 7.4 Hz, 2H,

H$_{18}$), 1.62 (m, 6H, H$_{13}$, H$_{23}$, H$_{26}$), 1.53 (d, $J$ = 6.9 Hz, 6H, H$_{30}$, H$_{31}$), 1.34 (m, 4H, H$_{24}$, H$_{27}$), 0.96 (t, $J$ = 7.5 Hz, 3H, H$_{14}$), 0.92 (t, $J$ = 7.4 Hz, 6H, H$_{25}$, H$_{28}$). $^{13}$C NMR (151 MHz, CD$_3$CN): δ 116.2 (C$_9$), 63.7 (C$_{15}$), 56.0 (C$_{12}$), 49.3 (C$_{29}$), 53.7 (C$_{21}$, C$_{22}$), 51.6 (C$_{19}$), 26.1 (C$_{23}$, C$_{26}$), 24.8 (C$_{13}$, C$_{18}$), 22.1 (C$_{30}$, C$_{31}$), 20.4 (C$_{24}$, C$_{27}$), 13.8 (C$_{25}$, C$_{28}$), 10.8 (C$_{14}$). HRMS (m/z): [M+H]$^+$ calcd. 434.3602, found 434.3595.

**(*R*)-2-((6-((2-cyclopropylethyl)amino)-9-isopropyl-9*H*-purin-2-yl)amino)butan-1-ol (DS37)**

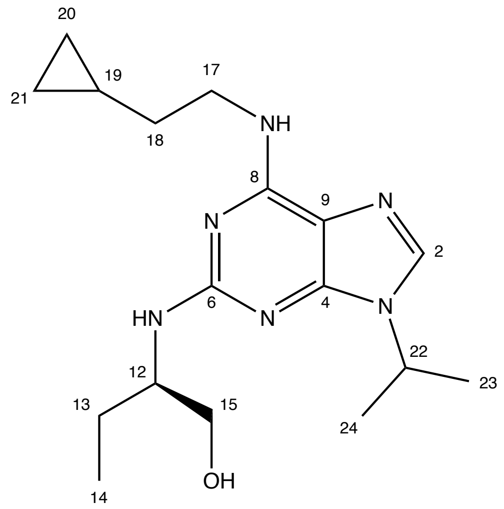


**DS37 (11)**

Derivative DS37 was prepared as per procedures B and D. UHPLC purification was performed at a detection wavelength of 225 nm. $^1$H NMR (600 MHz, CD$_3$CN): δ 7.81 (s, 1H, H$_2$), 6.77 (s, NH), 4.65 (m, 1H, H$_{22}$), 4.06 (m, 3H, H$_{12}$, H$_{17}$), 3.63 (m, 2H, H$_{15}$), 1.67 (m, 1H, imp), 1.56 (m, 2H, H$_{13}$), 1.52 (d, $J$ = 6.6 Hz, 6H, H$_{23}$, H$_{24}$), 1.11 (t, $J$ = 7.0 Hz, 2H, H$_{18}$), 0.96 (t, $J$ = 7.5 Hz, 3H, H$_{14}$), 0.76 (m, 1H, H$_{19}$), 0.44 (m, 2H, H$_{20}$), 0.11 (m, 2H, H$_{21}$). $^{13}$C NMR (151 MHz, CD$_3$CN): δ 152.5 (C$_4$), 150.3 (C$_6$), 140.0 (C$_2$), 116.2 (C$_9$), 64.1 (C$_{15}$), 55.7 (C$_{12}$), 49.5 (C$_{22}$), 45.0 (C$_{17}$), 35.3 (C$_{18}$), 34.6 (imp), 24.7 (C$_{13}$), 22.1 (C$_{23}$, C$_{24}$), 10.8 (C$_{14}$), 8.91 (C$_{19}$), 4.6 (C$_{20}$, C$_{21}$). HRMS (m/z): [M+H]$^+$ calcd. 333.2397, found 333.2403.

**(*R*)-2-((6-((2-cyclooctylethyl)amino)-9-isopropyl-9*H*-purin-2-yl)amino)butan-1-ol (DS38)**

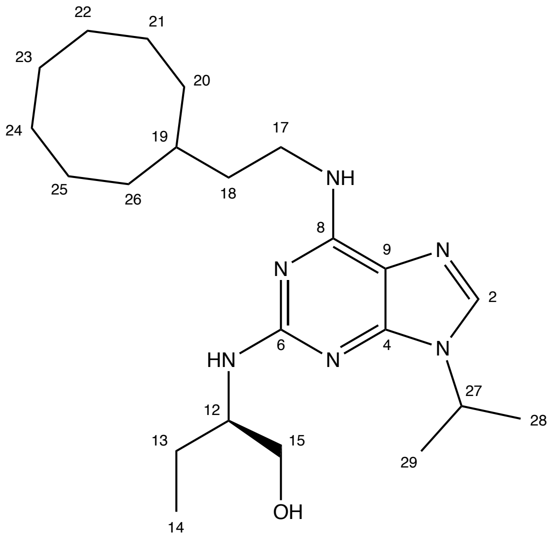

**DS38 (14)**

Derivative DS38 was prepared as per procedures B and D. UHPLC purification was performed at a detection wavelength of 220 nm. The gradient was 1-70% ACN in 16 min at a flow rate of 20 mL/min. $^1$H NMR (600 MHz, $CD_3CN$): δ 7.81 (s, 1H, $H_2$), 6.61 (s, NH), 4.68 (m, 1H, $H_{27}$), 3.99 (m, 3H, $H_{12}$, $H_{17}$), 3.62 (m, 2H, $H_{15}$), 1.67 (m, 6H, $H_{18}$, $H_{23}$, $H_{26}$), 1.59 (m, 6H, $H_{13}$, $H_{21}$, $H_{22}$), 1.61 (m, 11H, $H_{19}$, $H_{20}$, $H_{25}$, $H_{28}$, $H_{29}$), 1.34 (m, 2H, $H_{24}$), 0.96 (t, $J$ = 7.5 Hz, 3H, $H_{14}$). $^{13}$C NMR (151 MHz, $CD_3CN$): δ 152.5 ($C_4$), 150.1 ($C_6$), 139.6 ($C_2$), 115.8 ($C_9$), 64.0 ($C_{15}$), 55.7 ($C_{12}$), 49.8 ($C_{27}$), 43.1 ($C_{17}$), 38.6 ($C_{20}$, $C_{26}$), 37.5 ($C_{18}$), 32.8 ($C_{19}$), 27.8 ($C_{22}$, $C_{24}$), 27.0 ($C_{23}$), 26.1 ($C_{21}$, $C_{25}$), 24.7 ($C_{13}$), 22.1 ($C_{28}$, $C_{29}$), 10.8 ($C_{14}$). HRMS (m/z): $[M+H]^+$ calcd. 403.3180, found 403.3185.

**(*R*)-2-((6-((2-(benzyloxy)ethyl)amino)-9-isopropyl-9*H*-purin-2-yl)amino)butan-1-ol (DS40)**

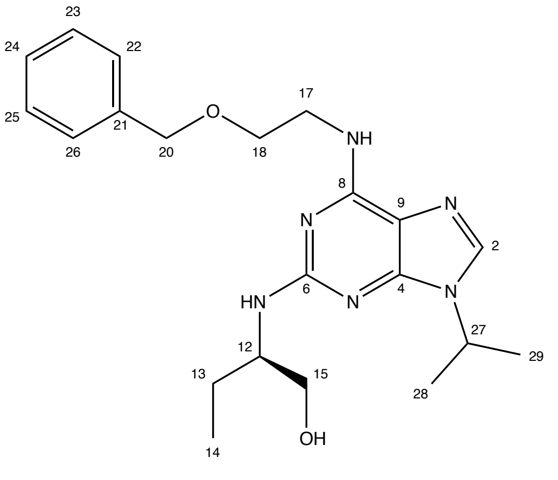

**DS40 (78)**

Derivative DS40 was prepared as per procedures B and D. UHPLC purification was performed at a detection wavelength of 200 nm. $^1$H NMR (600 MHz, $CD_3CN$): δ 7.80 (s, 1H, $H_2$), 7.28 (m, 5H, $H_{22}$,

$H_{23}$, $H_{24}$, $H_{25}$, $H_{26}$), 4.64 (m, 1H, $H_{27}$), 4.53 (s, 2H, $H_{20}$), 4.20 (m, 2H, $H_{18}$), 3.96 (s, 1H, $H_{12}$), 3.71 (m, 2H, $H_{17}$), 3.61 (m, 2H, $H_{15}$), 1.60 (m, 2H, $H_{13}$), 1.52 (d, $J$ = 7.4 Hz, 6H, $H_{28}$, $H_{29}$), 0.95 (m, 3H, $H_{14}$). $^{13}$C NMR (151 MHz, $CD_3CN$): δ 152.6 ($C_4$), 150.5 ($C_6$), 139.7 ($C_2$), 139.4 ($C_{21}$), 129.2 ($C_{23}$, $C_{25}$), 128.7 ($C_{22}$, $C_{26}$), 128.5 ($C_{24}$), 116.1 ($C_9$), 73.4 ($C_{20}$), 69.0 ($C_{18}$), 64.0 ($C_{15}$), 55.8 ($C_{12}$), 49.4 ($C_{27}$), 44.7 ($C_{17}$), 24.7 ($C_{13}$), 22.2 ($C_{28}$, $C_{29}$), 10.8 ($C_{14}$). HRMS (m/z): $[M+H]^+$ calcd. 399.2503, found 399.2506; $[M+Na]^+$ calcd. 421.2322, found 421.2317.

**(*R*)-2-((6-(((1-(3-chlorophenyl)-1*H*-pyrazol-3-yl)methyl)amino)-9-isopropyl-9*H*-purin-2-yl)amino)butan-1-ol (DS43)**

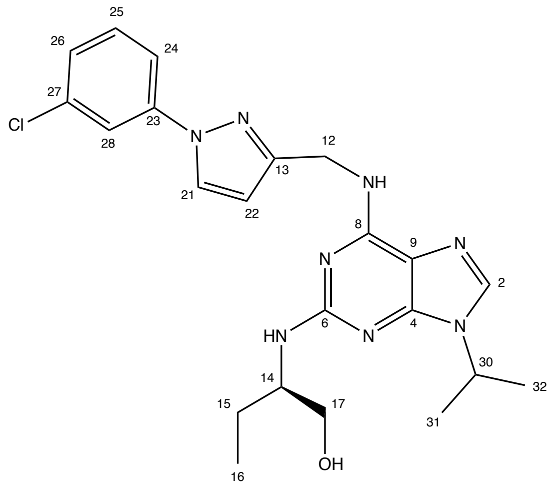


**DS43 (26)**

Derivative DS43 was prepared as per procedures B and D. UHPLC purification was performed at a detection wavelength of 210 nm. $^1$H NMR (600 MHz, $CD_3CN$): δ 8.10 (s, 1H, $H_{28}$), 7.80 (t, $J$ = 2.1 Hz, 1H, $H_{21}$), 7.69 (m, 1H, $H_{24}$), 7.45 (t, $J$ = 8.1 Hz, 1H, $H_{26}$), 7.31 (dd, $J$ = 8.0, 0.9 Hz, 1H, $H_{25}$), 6.52 (s, 1H, $H_{22}$), 4.83 (s, 2H, $H_{12}$), 4.67 (s, 1H, $H_{30}$), 4.00 (m, 1H, $H_{14}$), 3.58 (m, 2H, $H_{17}$), 1.62 (m, 2H, $H_{15}$), 1.63 (d, $J$ = 6.8 Hz, 6H, $H_{31}$, $H_{32}$), 0.95 (m, 3H, $H_{16}$). $^{13}$C NMR (151 MHz, $CD_3CN$): δ 144.5 ($C_{13}$), 141.8 ($C_{23}$), 135.5 ($C_{27}$), 132.0 ($C_{26}$), 129.8 ($C_{28}$), 127.1 ($C_{25}$), 119.5 ($C_{21}$), 117.7 ($C_{24}$), 115.8 ($C_9$), 108.1 ($C_{22}$), 64.3 ($C_{17}$), 55.8 ($C_{14}$), 49.6 ($C_{30}$), 40.8 ($C_{12}$), 24.8 ($C_{15}$), 22.2 ($C_{31}$, $C_{32}$), 10.8 ($C_{16}$). HRMS (m/z): $[M+H]^+$ calcd. 455.2069, found 455.2069.

**(*R*)-2-((9-isopropyl-6-((4-(pyridin-4-yl)benzyl)amino)-9*H*-purin-2-yl)amino)butan-1-ol (DS44)**

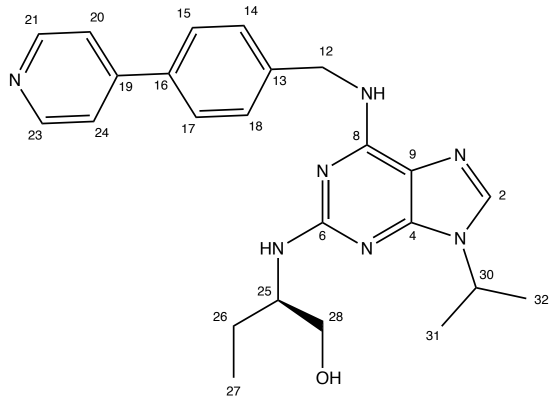

**DS44 (39)**

Derivative DS44 was prepared as per procedures B and D. UHPLC purification was performed at a detection wavelength of 300 nm. $^{1}$H NMR (600 MHz, $(CD_3)_2SO$): δ 8.83 (m, 2H, $H_{21}$, $H_{23}$), 8.12 (m, 2H, $H_{20}$, $H_{24}$), 7.93 (m, 2H, $H_{14}$, $H_{18}$), 7.57 (m, 2H, $H_{15}$, $H_{17}$), 5.32 (s, broad, OH/NH), 4.77 (m, 2H, $H_{12}$), 4.60 (m, 1H, $H_{30}$), 1.60 (m, 2H, $H_{26}$), 1.49 (m, 6H, $H_{31}$, $H_{32}$), 0.88 (m, 3H, $H_{27}$). $^{13}$C NMR (151 MHz, $(CD_3)_2SO$): δ 145.3 ($C_{21}$, $C_{23}$), 128.4 ($C_{14}/C_{15}/C_{17}/C_{18}$), 128.0 ($C_{14}/C_{15}/C_{17}/C_{18}$), 127.7 ($C_{14}/C_{15}/C_{17}/C_{18}$), 122.7 ($C_{20}$, $C_{24}$), 117.3 (n/a), 115.4 ($C_9$), 62.2 ($C_{25}$, $C_{28}$), 54.5 (imp), 47.5 ($C_{30}$), 43.4 ($C_{12}$), 23.7 ($C_{26}$), 21.8 ($C_{31}$, $C_{32}$), 10.5 ($C_{27}$). HRMS (m/z): $[M+H]^+$ calcd. 432.2506, found 432.2507.

**(*R*)-2-((9-isopropyl-6-((4-(pyridin-3-yl)benzyl)amino)-9*H*-purin-2-yl)amino)butan-1-ol (DS45)**

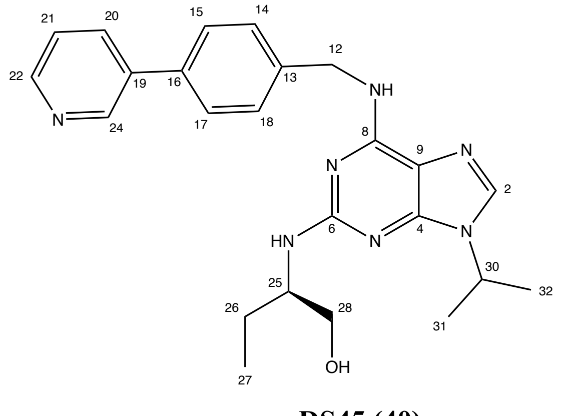

**DS45 (40)**

Derivative DS45 was prepared as per procedures B and D. UHPLC purification was performed at a detection wavelength of 230 nm. $^{1}$H NMR (600 MHz, $CD_3CN$): δ 8.97 (m, 1H, $H_{24}$), 8.71 (m, 2H, $H_{20}$, $H_{22}$), 8.04 (m, 1H, $H_{21}$), 7.72 (m, 2H, $H_{14}$, $H_{18}$), 7.60 (m, 2H, $H_{15}$, $H_{17}$), 5.37 (s, broad, OH/NH), 4.86 (s, 2H, $H_{12}$), 4.67 (m, 1H, $H_{30}$), 3.98 (m, 1H, $H_{25}$), 3.60 (m, 2H, $H_{28}$), 1.63 (m, 2H, $H_{26}$), 1.53 (m, 6H, $H_{31}$, $H_{32}$), 0.96 (t, $J$ = 7.6 Hz, 3H, $H_{27}$). $^{13}$C NMR (151 MHz, $CD_3CN$): δ 145.0 ($C_{22}$), 141.2 ($C_{20}/C_{24}$) 140.9 ($C_{20}/C_{24}$) , 129.5 ($C_{15}$, $C_{17}$), 128.6 ($C_{14}$, $C_{18}$), 128.3 ($C_{21}$), 117.8 (n/a), 115.9 ($C_9$), 64.1 ($C_{28}$), 55.2

($C_{25}$), 49.4 ($C_{30}$), 43.5 ($C_{12}$), 24.8 ($C_{26}$), 22.1 ($C_{31}$, $C_{32}$), 10.8 ($C_{27}$). HRMS (m/z): $[M+H]^+$ calcd. 432.2506, found 432.2506.

**(*R*)-3-(4-((2-((1-hydroxybutan-2-yl)amino)-9-isopropyl-9*H*-purin-6-yl)amino)butyl)benzo[*d*]oxazol-2(3*H*)-one (DS46)**

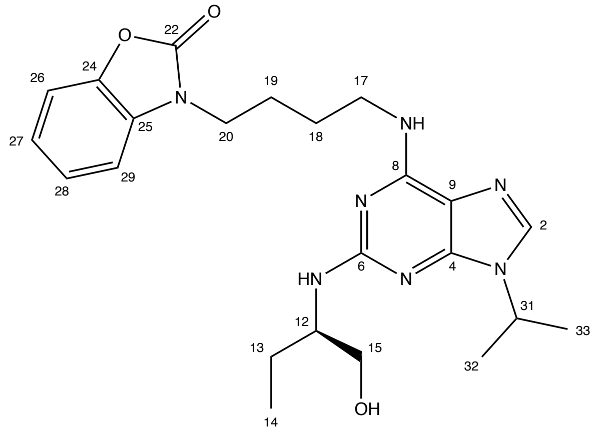


**DS46 (20)**

Derivative DS46 was prepared as per procedures B and D. UHPLC purification was performed at a detection wavelength of 200 nm. $^1$H NMR (600 MHz, $CD_3CN$): δ 7.75 (s, 1H, $H_2$), 7.18 (m, 2H, $H_{26}$, $H_{27}$), 7.11 (m, 2H, $H_{28}$, $H_{29}$), 6.65 (s, broad, NH), 4.62 (m, 1H, $H_{31}$), 4.00 (m, 3H, $H_{12}$, $H_{17}$), 3.85 (m, 2H, $H_{20}$), 3.60 (m, 2H, $H_{15}$), 1.86 (m, 2H, $H_{19}$), 1.74 (m, 2H, $H_{18}$), 1.61 (m, 2H, $H_{13}$), 1.51 (d, $J$ = 6.6 Hz, 6H, $H_{32}$, $H_{33}$), 0.95 (t, $J$ = 7.6 Hz, 3H, $H_{14}$). $^{13}$C NMR (151 MHz, $CD_3CN$): δ 143.6 ($C_{22}$), 139.6 ($C_{24}$), 132.4 ($C_{25}$), 124.8 ($C_{28}$), 123.1 ($C_{27}$), 116.2 ($C_9$), 110.5 ($C_{26}$), 109.8 ($C_{29}$), 63.0 ($C_{15}$), 55.7 ($C_{12}$), 47.8 ($C_{31}$), 43.2 ($C_{17}$), 42.6 ($C_{20}$), 27.5 ($C_{18}$), 25.3 ($C_{19}$), 24.7 ($C_{13}$), 22.2 ($C_{32}$, $C_{33}$), 10.8 ($C_{14}$). HRMS (m/z): $[M+H]^+$ calcd. 454.2561, found 454.2569.

**(*S*)-2-((*S*)-*N*-(4-(hydroxymethyl)phenyl)-2-((9-isopropyl-6-((4-(pyridin-2-yl)benzyl)amino)-9*H*-purin-2-yl)amino)-3-methylbutanamido)-5-ureidopentanamide (DS47)**

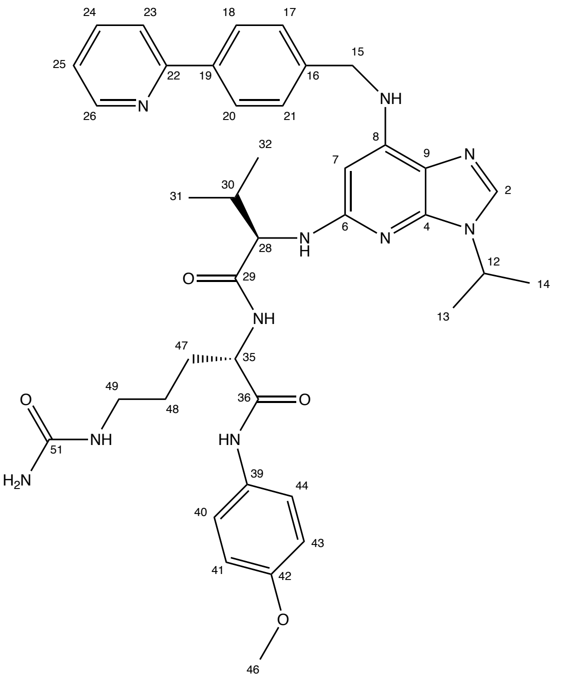

**DS47 (52)**

Derivative DS47 was commercially synthesized by Wuxi AppTec (Shanghai, China). The final compound was isolated as a free base. $^{1}$H NMR (600 MHz, $CD_3CN$): δ 8.76 (s, NH), 8.62 (m, 1H, $H_{26}$), 7.97 (m, 2H, $H_{18}$, $H_{20}$), 7.79 (m, 2H, $H_2$, $H_{23}$), 7.58 (m, 1H, $H_{24}$), 7.48 (m, 2H, $H_{17}$, $H_{21}$), 7.26 (m, 2H, $H_{40}$, $H_{44}$), 6.74 (m, 3H, $H_{25}$, $H_{41}$, $H_{43}$), 5.45 (s, OH/NH), 4.71 (m, 2H, $H_{15}$), 4.57 (m, 1H, $H_{12}$), 4.46 (m, 1H, $H_{35}$), 3.71 (m, 3H, $H_{46}$), 3.20 (m, 1H, n/a), 2.93 (m, 1H, $H_{28}$), 2.13 (m, 1H, $H_{30}$) 1.76/1.54 (m, 2H, $H_{47}$), 1.45 (m, 6H, $H_{13}$, $H_{14}$), 1.34 (m, 2H, $H_{48}$), 0.98 (m, 6H, $H_{31}$, $H_{32}$). $^{13}$C NMR (151 MHz, $CD_3CN$): δ 174.2 ($C_{36}$), 171.2 ($C_{29}$), 160.3 ($C_{51}$), 160.0 ($C_6$), 157.6 ($C_{42}$), 156.9 ($C_{22}$), 150.5 ($C_{26}$), 142.4 ($C_{16}$), 138.8 ($C_{19}$), 137.9 ($C_2$), 136.6 ($C_{24}$), 132.4 ($C_{39}$), 128.8 ($C_{17}$, $C_{21}$), 127.6 ($C_{18}$, $C_{20}$), 123.2 ($C_{25}$), 122.1 ($C_{40}$, $C_{44}$), 121.1 ($C_{23}$), 114.7 ($C_9$, $C_{41}$, $C_{43}$), 62.7 ($C_{28}$), 55.9 ($C_{46}$), 53.8 ($C_{12}$), 53.2 ($C_{35}$), 47.2 ($C_{15}$), 39.1 ($C_{49}$), 31.3 (n/a), 30.5 ($C_{30}$), 29.8 ($C_{47}$), 27.7 ($C_{48}$), 22.6 ($C_{13}$, $C_{14}$), 20.0, 19.0 ($C_{31}$, $C_{32}$). HRMS (m/z): $[M+H]^+$ calcd. 722.3885, found 722.3886.

**$N^2$-((3,5-dichloropyridin-4-yl)methyl)-9-isopropyl-$N^6$-(4-(pyridin-2-yl)benzyl)-9*H*-purine-2,6-diamine (DS48)**

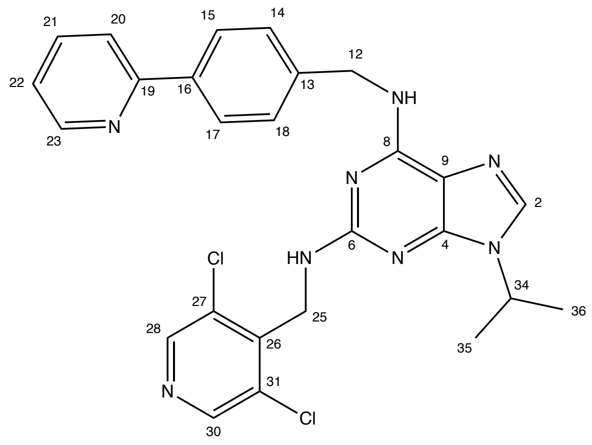

**DS48 (46)**

Derivative DS48 was prepared as per procedures B and E. If necessary, an excess of morpholine can be added once product forms in order to quench the reaction. UHPLC purification was performed at a detection wavelength of 200 nm. $^1$H NMR (600 MHz, $CD_3CN$): δ 8.78 (m, 1H, $H_{28}$/$H_{30}$), 8.50 (m, 1H, $H_{28}$/$H_{30}$), 8.44 (m, 1H, $H_{23}$), 8.30 (t, $J$ = 7.5 Hz, 1H, $H_{21}$), 8.08 (d, $J$ = 8.7 Hz, 1H, $H_{20}$), 7.92 (m, 2H, $H_{15}$, $H_{17}$), 7.71 (m, 1H, $H_{22}$), 7.60 (m, 2H, $H_{14}$, $H_{18}$), 4.85 (m, 4H, $H_{12}$, $H_{25}$), 4.41 (m, 1H, $H_{34}$), 3.83 (t, $J$ = 5.2 Hz, 1H, imp), 3.72 (t, $J$ = 4.0 Hz, 1H, imp), 3.63 (t, $J$ = 4.9 Hz, 1H, imp), 1.52 (m, 6H, $H_{35}$, $H_{36}$). $^{13}$C NMR (151 MHz, $CD_3CN$): δ 161.1 ($C_6$), 149.2 ($C_{19}$), 148.8 ($C_{28}$, $C_{30}$), 133.9 ($C_{27}$, $C_{31}$), 129.4 ($C_{14}$, $C_{18}$), 128.9 ($C_{15}$, $C_{17}$), 125.3 ($C_{20}$, $C_{22}$), 116.2 ($C_9$), 53.7 ($C_{34}$), 44.7 ($C_{12}$, $C_{25}$), 26.1 (imp), 22.2 ($C_{35}$, $C_{36}$), 20.4 (imp). HRMS (m/z): $[M+H]^+$ calcd. 519.1574, found 519.1564.

**2-((9-isopropyl-6-((4-(pyridin-2-yl)benzyl)amino)-9*H*-purin-2-yl)amino)-*N*-(2-methoxyphenethyl)acetamide (DS50)**

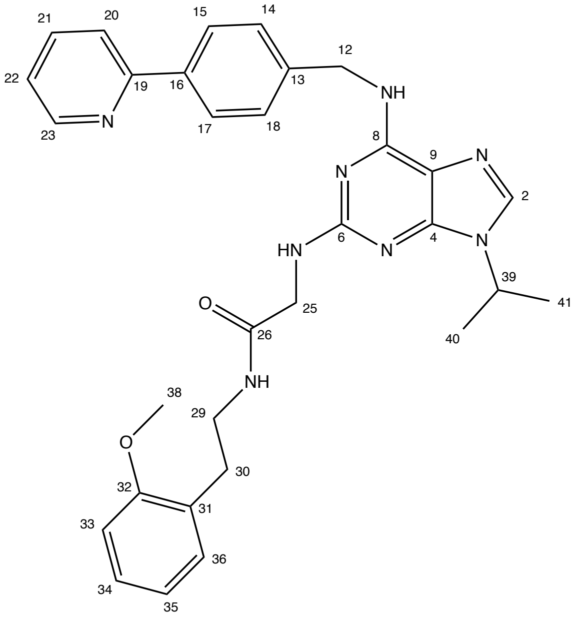


**DS50 (49)**

Derivative DS50 was prepared as per procedures B and E. UHPLC purification was performed at a detection wavelength of 300 nm. $^1$H NMR (600 MHz, $CD_3CN$): δ 8.79 (m, 1H, $H_{23}$), 8.29 (m, 1H, $H_{21}$), 8.06 (m, 1H, $H_{20}$), 7.90 (m, 3H, $H_2$, $H_{15}$, $H_{17}$), 7.71 (m, 1H, $H_{22}$), 7.60 (m, 2H, $H_{14}$, $H_{18}$), 7.04 (m, 1H, $H_{36}$), 6.90 (m, 2H, $H_{34}$, $H_{35}$), 6.75 (d, $J$ = 7.7 Hz, 1H, $H_{33}$), 5.41 (s, OH/NH), 4.71 (m, 2H, $H_{12}$), 3.95 (m, 1H, $H_{39}$), 3.82 (m, 2H, $H_{38}$), 3.71 (m, 2H, $H_{25}$), 3.35 (q, $J$ = 6.7 Hz, 2H, $H_{29}$), 1.53 (m, 6H, $H_{40}$, $H_{41}$). $^{13}$C NMR (151 MHz, $CD_3CN$): δ 160.5 ($C_6$), 158.6 ($C_{32}$), 131.5 ($C_{31}$), 129.5 ($C_{14}$, $C_{18}$), 129.2 ($C_{34}$), 128.9 ($C_{15}$, $C_{17}$), 127.3 ($C_{36}$), 125.4 ($C_{22}$), 121.6 ($C_{20}$), 121.2 ($C_{35}$), 116.2 ($C_9$), 111.6 ($C_{33}$), 73.5 (n/a), 56.1 ($C_{38}$), 53.5 ($C_{39}$), 45.4 ($C_{12}$/$C_{25}$), 44.3 ($C_{12}$/$C_{25}$), 41.0 ($C_{29}$), 29.2 ($C_{30}$), 26.2 (imp), 22.1 ($C_{40}$, $C_{41}$), 20.4 (imp). HRMS (m/z): $[M+H]^+$ calcd. 551.2877, found 551.2870; $[M+Na]^+$ calcd. 573.2697, found 573.2691; $[M+K]^+$ calcd. 589.2436, found 589.2425.

**6-((9-isopropyl-6-((4-(pyridin-2-yl)benzyl)amino)-9H-purin-2-yl)amino)-N-((2S,3R,4S,5S,6R)-3,4,5-trihydroxy-6-(hydroxymethyl)tetrahydro-2H-pyran-2-yl)hexanamide (DS51)**

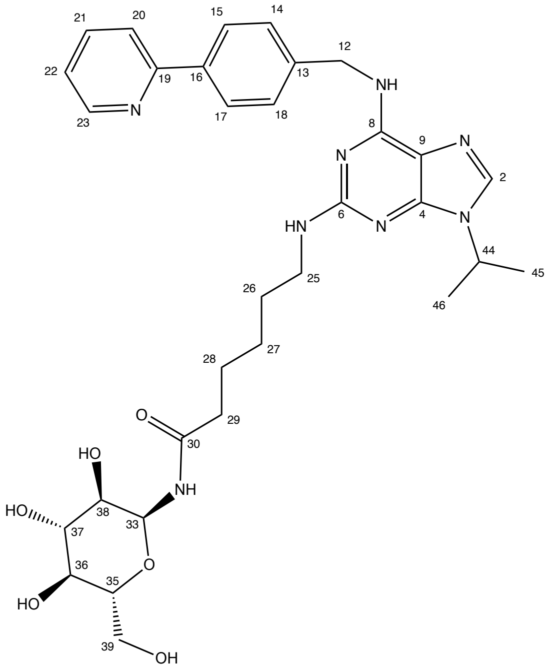

**DS51 (50)**

Derivative DS51 was prepared as per procedures B and E. To assist with dissolving the primary amine, DMF was added at a 1:1 volumetric ratio to the base. UHPLC purification was performed at a detection wavelength of 300 nm. $^{1}$H NMR (600 MHz, $(CD_3)_2SO$): δ 8.65 (m, 1H, $H_{23}$), 8.26 (m, 2H, n/a), 8.04 (d, $J$ = 7.9 Hz, 2H, $H_{15}$, $H_{17}$), 7.94 (d, $J$ = 8.0 Hz, 1H, $H_{21}$), 7.88 (td, $J$ = 7.7, 1.8 Hz, 1H, $H_{20}$), 7.47 (d, $J$ = 8.0 Hz, 2H, $H_{14}$, $H_{18}$), 7.34 (m, 1H, $H_{22}$), 4.89 (s, broad, 2H, $H_{33}$, $H_{44}$), 4.70 (m, 2H, $H_{12}$), 4.58 (m, 1H, $H_{38}$), 3.16 (m, 2H, $H_{25}$), 3.04 (m, 5H, $H_{35,}$ $H_{36}$, $H_{37}$, $H_{39}$), 2.08 (m, 2H, $H_{29}$), 1.48 (d, $J$ = 6.7 Hz, 6H, $H_{45}$, $H_{46}$), 1.26 (m, 6H, $H_{26,}$ $H_{27}$, $H_{28}$). $^{13}$C NMR (151 MHz, $(CD_3)_2SO$): δ 137.9 ($C_{20}$), 128.2 ($C_{14}$, $C_{18}$), 127.0 ($C_{15}$, $C_{17}$), 123.2 ($C_{22}$), 120.7 ($C_{21}$), 79.9 ($C_{12}$), 79.0 ($C_{37}$), 78.0 ($C_{25}$), 73.0 ($C_{35}$, $C_{36}$), 70.5 ($C_{39}$), 35.9 ($C_{29}$), 29.6 ($C_{26}$), 26.7 ($C_{27}$, $C_{28}$), 22.4 ($C_{45}$, $C_{46}$). HRMS (m/z): $[M+H]^+$ calcd. 635.3300, found 635.3292.

**$N^2$-(2-(1$H$-pyrazol-4-yl)ethyl)-9-isopropyl-$N^6$-(4-(pyridin-2-yl)benzyl)-9$H$-purine-2,6-diamine (DS52)**

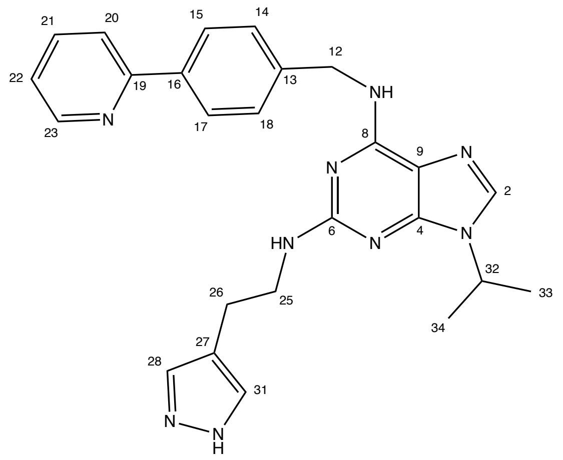

**DS52 (47)**

Derivative DS52 was prepared as per procedures B and E. To assist with dissolving the primary amine, DMF was added at a 2:1 volumetric ratio to the base. UHPLC purification was performed at a detection wavelength of 245 nm. $^1$H NMR (600 MHz, $(CD_3)_2SO$): δ 8.66 (m, 1H, $H_{23}$), 8.04 (m, 2H, $H_{15}$, $H_{17}$), 7.95 (d, $J$ = 8.1 Hz, 1H, $H_{21}$), 7.90 (td, $J$ = 7.7, 1.8 Hz, 1H, $H_{20}$), 7.47 (m, 4H, $H_{14}$, $H_{18}$, $H_{28}$, $H_{31}$), 7.37 (m, 1H, $H_{22}$), 4.72 (m, 2H, $H_{12}$), 4.62 (m, 1H, $H_{32}$), 3.48 (m, 2H, $H_{25}$), 3.33 (m, 2H, imp), 3.22 (m, 2H, $H_{26}$), 1.50 (m, 6H, $H_{33}$, $H_{34}$), 1.23 (s, 2H, imp). $^{13}$C NMR (151 MHz, $(CD_3)_2SO$): δ 158.1 ($C_6$), 155.5 ($C_{19}$), 149.8 ($C_{23}$), 138.4 ($C_{20}$), 128.3 ($C_{14}$, $C_{18}$, $C_{28}$, $C_{31}$), 127.3 ($C_{15}$, $C_{17}$), 123.3 ($C_{22}$), 121.1 ($C_{21}$), 47.1 ($C_{32}$), 43.7 ($C_{12}$), 42.9 ($C_{25}$), 39.1 ($C_{26}$), 29.4 (imp), 22.4 ($C_{33}$, $C_{34}$). HRMS (m/z): $[M+H]^+$ calcd. 454.2462, found 454.2454.

**($R$)-2-((6-(([2,4'-bipyridin]-5-ylmethyl)amino)-9-isopropyl-9$H$-purin-2-yl)amino)butan-1-ol (DS53)**

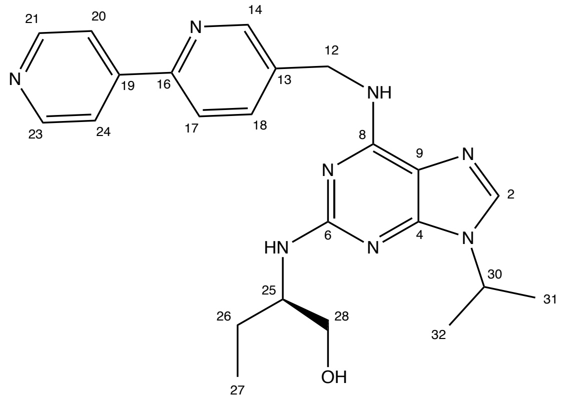

**DS53 (38)**

Derivative DS53 was prepared as per procedures B and D. UHPLC purification was performed at a detection wavelength of 290 nm. $^1$H NMR (600 MHz, $CD_3CN$): δ 8.80 (m, 3H, $H_{14}$, $H_{21}$, $H_{23}$), 8.32 (m,

2H, $H_{20}$, $H_{24}$), 8.04 (m, 1H, $H_{17}$), 7.97 (m, 1H, $H_{18}$), 4.84 (m, 2H, $H_{12}$), 4.70 (s, 1H, $H_{30}$), 3.96 (m, 1H, $H_{25}$), 3.52 (m, 2H, $H_{28}$), 1.53 (m, 2H, $H_{26}$), 1.52 (d, $J$ = 6.7 Hz, 6H, $H_{31}$, $H_{32}$), 0.96 (t, $J$ = 7.5 Hz, 3H, $H_{27}$). $^{13}$C NMR (151 MHz, $CD_3CN$): δ 156.3 ($C_{16}$), 153.9 ($C_4$), 150.7 ($C_{21}$, $C_{23}$) , 137.7 ($C_{18}$), 123.7 ($C_{20}$, $C_{24}$), 122.8 ($C_{17}$), 64.2 ($C_{28}$), 61.1 (imp), 55.7 ($C_{25}$), 48.6 ($C_{30}$), 41.3 ($C_{12}$), 24.7 ($C_{26}$), 23.1 (imp), 22.2 ($C_{31}$, $C_{32}$), 10.8 ($C_{27}$). HRMS (m/z): $[M+H]^+$ calcd. 433.2459, found 433.2454.

**2-(((9-(1-methyl-1*H*-pyrazol-4-yl)-2-morpholino-9*H*-purin-6-yl)amino)methyl)-1*H*-benzo[*d*]imidazol-5-ol (DS54)**

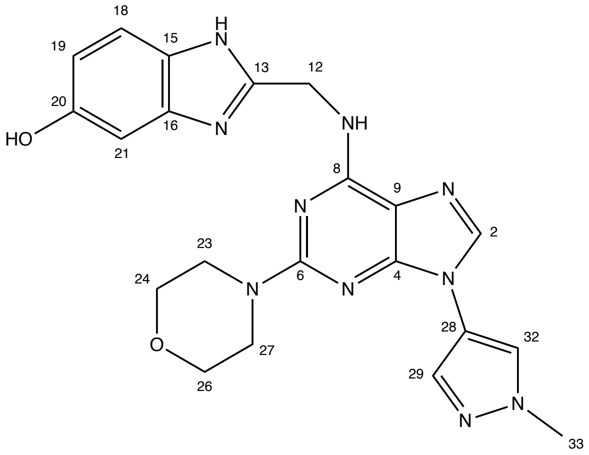


**DS54 (64)**

Derivative DS54 was prepared as per procedures C and D. The crude reaction mixture after procedure C was used directly in the next step without performing an extraction. UHPLC purification was performed at a detection wavelength of 230 nm. $^1$H NMR (600 MHz, $CD_3CN$): δ 8.23 (s, 1H, $H_2$), 8.11 (s, 1H, $H_{29}$), 7.86 (s, 1H, $H_{32}$), 7.54 (d, $J$ = 8.9 Hz, 1H, $H_{18}$), 7.09 (m, 1H, $H_{21}$), 7.02 (dd, $J$ = 8.9, 2.3 Hz, 1H, $H_{19}$), 5.09 (s, 2H, $H_{12}$), 3.92 (s, 3H, $H_{33}$), 3.83 (m, 3H, imp), 3.54 (m, 4H, $H_{23}$, $H_{27}$), 3.52 (m, 4H, $H_{24}$, $H_{26}$). $^{13}$C NMR (151 MHz, $CD_3CN$): δ 136.7 ($C_2$), 131.7 ($C_{32}$), 123.9 ($C_{29}$), 115.9 ($C_{19}$), 114.7 ($C_{18}$), 98.7 ($C_{21}$), 66.2 ($C_{24}$, $C_{26}$), 63.5 (imp), 44.9 ($C_{23}$, $C_{27}$), 39.3 ($C_{33}$), 37.0 ($C_{12}$). HRMS (m/z): $[M+H]^+$ calcd. 447.2000, found 447.1996; $[M+Na]^+$ calcd. 469.1819, found 469.1810.

**_N_-((1_H_-benzo[_d_]imidazol-2-yl)methyl)-9-(1-methyl-1_H_-pyrazol-4-yl)-2-morpholino-9_H_-purin-6-amine (DS55)**

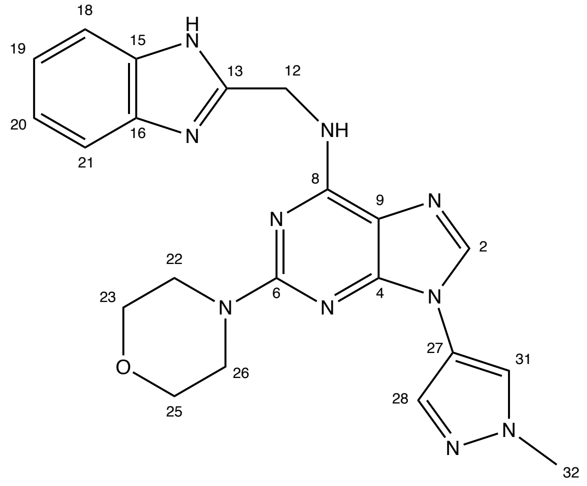

**DS55 (61)**

Derivative DS55 was prepared as per procedures C and D. The crude reaction mixture after procedure C was used directly in the next step without performing an extraction. UHPLC purification was performed at a detection wavelength of 200 nm. $^{1}H$ NMR (600 MHz, $CD_3CN$): δ 8.05 (s, 1H, $H_{28}$), 8.02 (s, 1H, $H_{31}$), 7.82 (s, 1H, n/a), 7.73 (m 2H, $H_{18}$, $H_{21}$), 7.51 (m, 2H, $H_{19}$, $H_{20}$), 5.18 (s, 2H, $H_{12}$), 3.90 (s, 3H, $H_{32}$), 3.52 (m, 4H, $H_{22}$, $H_{26}$), 3.50 (m, 4H, $H_{23}$, $H_{25}$). $^{13}C$ NMR (151 MHz, $CD_3CN$): δ 137.5 ($C_{31}$), 132.2 ($C_{15}$, $C_{16}$), 126.9 ($C_{19}$, $C_{20}$), 124.3 ($C_{28}$), 115.9 ($C_9$), 115.0 ($C_{18}$, $C_{21}$), 67.1 ($C_{23}$, $C_{25}$), 45.6 ($C_{22}$, $C_{26}$), 39.9 ($C_{32}$), 38.0 ($C_{12}$). HRMS (m/z): $[M+H]^+$ calcd. 431.2051, found 431.2055.

**_N_-((1_H_-indol-2-yl)methyl)-9-(1-methyl-1_H_-pyrazol-4-yl)-2-morpholino-9_H_-purin-6-amine (DS56)**

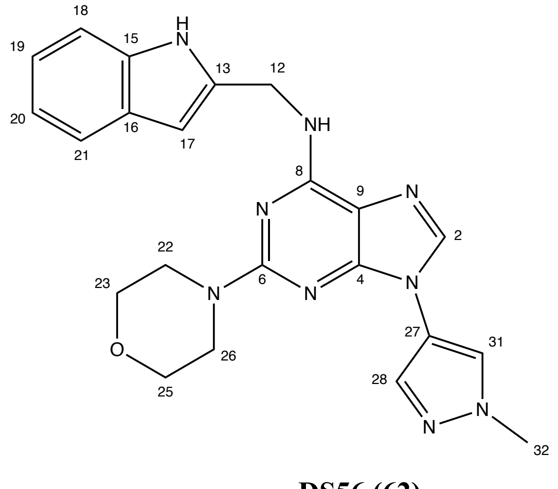

**DS56 (62)**

Derivative DS56 was prepared as per procedures C and D. The crude reaction mixture after procedure C was used directly in the next step without performing an extraction. UHPLC purification was

performed at a detection wavelength of 225 nm. $^1$H NMR (600 MHz, $CD_3CN$): δ 8.12 (s, 1H, $H_{28}$), 7.86 (s, 1H, $H_{31}$), 7.68 (m, 1H, n/a), 7.49 (m, 1H, $H_{21}$), 7.33 (m, 1H, $H_{18}$), 7.07 (m, 1H, $H_{19}$), 7.00 (m, 1H, $H_{20}$), 6.38 (s, 1H, $H_{17}$), 4.88 (s, 2H, $H_{12}$), 3.93 (s, 3H, $H_{32}$), 3.84 (m, 2H, imp), 3.79 (m, 4H, $H_{22}$, $H_{26}$), 3.69 (m, 4H, $H_{23}$, $H_{25}$). $^{13}$C NMR (151 MHz, $CD_3CN$): δ 131.6 ($C_{31}$), 123.8 ($C_{28}$), 121.3 ($C_{19}$), 120.1 ($C_{21}$), 119.6 ($C_{20}$), 111.0 ($C_{18}$), 100.0 ($C_{17}$), 66.4 ($C_{23}$, $C_{25}$), 63.5 (imp), 45.1 ($C_{22}$, $C_{26}$), 39.1 ($C_{32}$), 37.4 ($C_{12}$). HRMS (m/z): $[M+H]^+$ calcd. 430.2098, found 430.2096.

**$N^2$-(2-(1*H*-pyrazol-4-yl)ethyl)-$N^6$-((5,6-dichloro-1*H*-benzo[*d*]imidazol-2-yl)methyl)-9-(1-methyl-1*H*-pyrazol-4-yl)-9*H*-purine-2,6-diamine (DS57)**

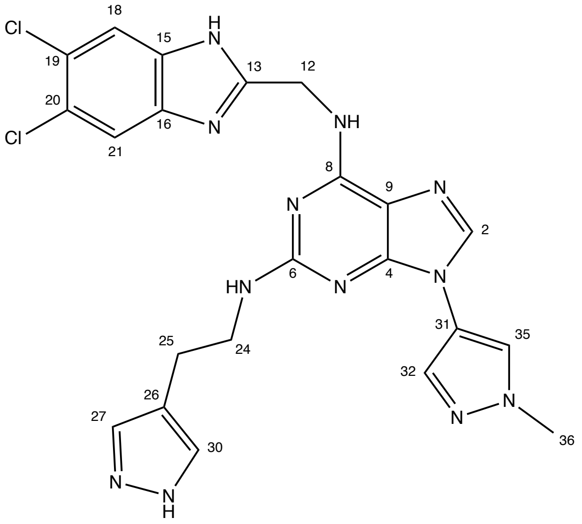


**DS57 (63)**

Derivative DS57 was prepared as per procedures C and E. To assist with dissolving the primary amine, DMF was added at a 1:1 volumetric ratio to the base. UHPLC purification was performed at a detection wavelength of 220 nm. $^1$H NMR (600 MHz, $(CD_3)_2SO$): δ 8.99 (s broad, NH), 8.55 (s, 2H, $H_{18}$, $H_{21}$), 8.42 (s, 1H, $H_2$), 8.07 (s, 1H, $H_{32}$), 7.83 (s, 1H, $H_{35}$), 7.77 (s, broad, 4H, $H_{27}$, $H_{30}$, NH), 4.94 (m, 2H, $H_{12}$), 3.96 (s, 3H, $H_{36}$), 3.06 (m, 4H, imp), 2.79 (m, 2H, $H_{25}$), 1.57 (m, 2H, imp), 1.23 (m, 2H, $H_{24}$). $^{13}$C NMR (151 MHz, $(CD_3)_2SO$): δ 143.0 ($C_{27}$, $C_{30}$, $C_{35}$), 140.5 ($C_{18}$, $C_{21}$), 132.5 ($C_{32}$), 128.6 (n/a), 124.7 ($C_2$), 52.3 (imp), 39.8 ($C_{25}$), 39.7 ($C_{36}$), 39.5 ($C_{12}$), 29.4 ($C_{24}$), 25.7 (imp). HRMS (m/z): $[M+H]^+$ calcd. 523.1384, found 523.1392; $[M+Na]^+$ calcd. 545.1203, found 545.1206.

**(*S*)-*N*$^{2}$-(2-(4-(benzyloxy)phenyl)-1-(4*H*-1,2,4-triazol-3-yl)ethyl)-9-isopropyl-*N*$^{6}$-(4-(pyridin-2-yl)benzyl)-9*H*-purine-2,6-diamine (DS58)**

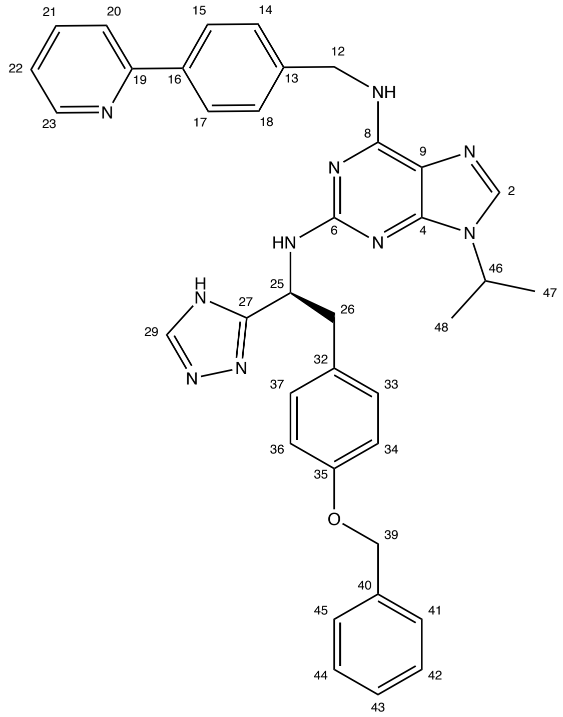

**DS58 (51)**

Derivative DS58 was prepared as per procedures B and E. To assist with dissolving the primary amine, DMF was added at a 2:1 volumetric ratio to the base. UHPLC purification was performed at a detection wavelength of 230 nm. $^{1}$H NMR (600 MHz, $CD_3CN$): δ 9.21 (s, 1H, $H_{29}$), 8.73 (d, *J* = 5.4 Hz, 1H, $H_{23}$), 8.17 (m, 2H, $H_{15}$, $H_{17}$), 8.00 (m, 1H, $H_{20}$), 7.94 (d, *J* = 7.9 Hz, 2H, $H_{41}$, $H_{45}$), 7.68 (d, *J* = 7.9 Hz, 2H, $H_{42}$, $H_{44}$), 7.61 (t, *J* = 6.5 Hz, 1H, $H_{22}$), 7.37 (m, 4H, $H_{14}$, $H_{18}$, $H_{21}$, $H_{43}$), 7.30 (m, 1H, n/a), 7.14 (d, *J* = 8.2 Hz, 2H, $H_{33}$, $H_{37}$), 6.87 (m, 2H, $H_{34}$, $H_{36}$), 5.00 (s, 2H, $H_{39}$), 4.89 (m, 2H, $H_{12}$), 4.84 (m, 1H, $H_{25}$), 4.76 (m, 1H, $H_{46}$), 3.38 (m, 2H, $H_{26}$), 1.56 (d, *J* = 6.8 Hz, 6H, $H_{47}$, $H_{48}$). $^{13}$C NMR (151 MHz, $CD_3CN$): δ 161.5 ($C_{27}$), 159.1 ($C_{19}$), 155.4 ($C_{35}$), 150.1 ($C_6$), 146.3 ($C_{23}$, $C_{29}$), 143.3 ($C_{15}$, $C_{17}$), 140.3 ($C_{13}$, $C_{16}$), 138.2 ($C_{40}$), 131.8 ($C_{33,}$ $C_{37}$), 129.7 ($C_{42}$/$C_{44}$), 129.5 ($C_{42}$/$C_{44}$), 128.9 ($C_{14}$, $C_{18}$), 128.7 ($C_{43}$), 128.6 ($C_{41}$, $C_{45}$), 127.8 ($C_{21}$), 125.0 ($C_{22}$), 124.4 ($C_{20}$), 116.0 ($C_9$, $C_{34}$, $C_{36}$), 70.5 ($C_{39}$), 52.4 ($C_{25}$), 48.8 ($C_{46}$), 44.6 ($C_{12}$), 38.1 ($C_{26}$), 22.6 ($C_{47}$, $C_{48}$). HRMS (m/z): $[M+H]^+$ calcd. 637.3146, found 637.3150; $[M+Na]^+$ calcd. 659.2966, found 659.2964; $[M+K]^+$ calcd. 675.2705, found 675.2703.

**5-(((9-isopropyl-6-((4-(pyridin-2-yl)benzyl)amino)-9*H*-purin-2-yl)amino)methyl)-1,3-dimethyl-1*H*-pyrazole-4-sulfonamide (DS59)**

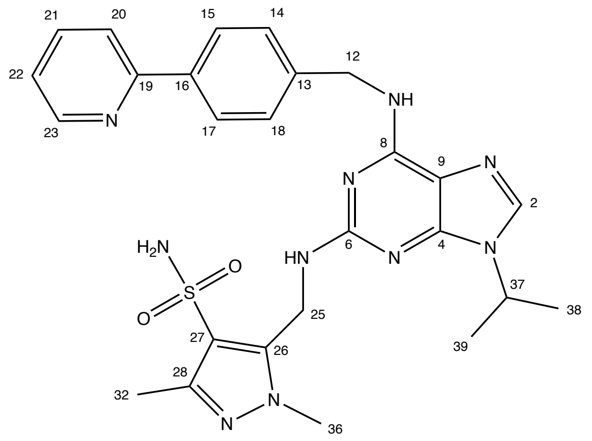

**DS59 (48)**

Derivative DS59 was prepared as per procedures B and E. To assist with dissolving the primary amine, DMF was added at a 1:1 volumetric ratio to the base. UHPLC purification was performed at a detection wavelength of 230 nm. $^1$H NMR (600 MHz, $CD_3CN$): δ 8.74 (m, 1H, $H_2$), 8.08 (m, 1H, $H_{23}$), 7.97 (m, 4H, $H_{15}$, $H_{17}$, $H_{20}$, $H_{21}$), 7.55 (m, 3H, $H_{14}$, $H_{18}$, $H_{22}$), 6.17 (s, broad, NH), 4.92 (s, 2H, $H_{12}$), 4.72 (m, broad, 1H, $H_{37}$), 3.91 (s, 2H, $H_{25}$), 3.75 (d, 1H, imp), 2.30 (m, 6H, $H_{32}$, $H_{36}$), 1.52 (d, $J$ = 6.8 Hz, 6H, $H_{38}$, $H_{39}$). $^{13}$C NMR (151 MHz, $CD_3CN$): δ 147.2 ($C_2$), 140.3 ($C_{23}$), 127.9 ($C_{14}$, $C_{18}$), 127.6 ($C_{15}$, $C_{17}$), 123.5 ($C_{22}$), 122.3 ($C_{20}$, $C_{21}$), 48.9 ($C_{37}$), 43.5 ($C_{12}$), 36.8 ($C_{25}$), 21.4 ($C_{38}$, $C_{39}$), 12.0 ($C_{32}$, $C_{36}$). HRMS (m/z): $[M+H]^+$ calcd. 547.2347, found 547.2347; $[M+Na]^+$ calcd. 569.2166, found 569.2159.

**(*R*)-6'-(((2-((1-hydroxybutan-2-yl)amino)-9-isopropyl-9*H*-purin-6-yl)amino)methyl)-[2,3'-bipyridin]-2'(1'*H*)-one (WX3)**

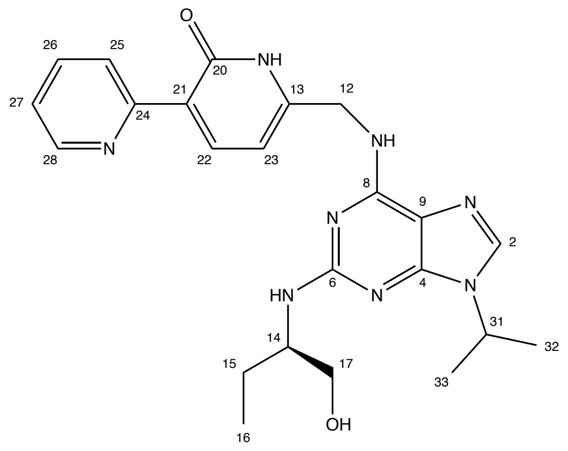

**WX3 (41)**

Derivative WX3 was commercially synthesized by Wuxi AppTec (Shanghai, China). The final compound was isolated as a free base. $^1$H NMR (600 MHz, $CD_3CN$): δ 8.59 (m, 1H, $H_{28}$), 8.41 (s, 1H, $H_2$), 7.78 (s, 1H, $H_{22}$), 7.63 (s, 1H, $H_{25}$), 7.28 (s, 1H, $H_{26}$), 6.67 (s, 1H, $H_{27}$), 6.44 (s, 1H, $H_{23}$), 5.25 (m,

1H, $H_{31}$), 4.57 (m, 2H, $H_{12}$), 3.98 (m, 1H, $H_{14}$), 3.58 (m, 2H, $H_{17}$), 1.63 (m, 2H, $H_{15}$), 1.51 (d, $J$ = 6.9 Hz, 6H, $H_{32}$, $H_{33}$), 0.95 (m, 3H, $H_{16}$). $^{13}$C NMR (151 MHz, $CD_3CN$): δ 155.8 ($C_{20}$), 152.2 ($C_{28}$), 143.4 ($C_{22}$), 137.0 ($C_{25}$), 123.3 ($C_{26}$), 114.8 ($C_9$), 65.8 ($C_{17}$), 56.4 ($C_{14}$), 47.5 ($C_{12}$), 25.4 ($C_{15}$), 22.5 ($C_{32}$, $C_{33}$), 11.1 ($C_{16}$). HRMS (m/z): $[M+H]^+$ calcd. 449.2408, found 449.2409; $[M+Na]^+$ calcd. 471.2227, found 471.2224.

**(*R*)-2-((6-(((4-(1,3-dioxolan-2-yl)-[2,2'-bipyridin]-5-yl)methyl)amino)-9-isopropyl-9*H*-purin-2-yl)amino)butan-1-ol (WX4)**

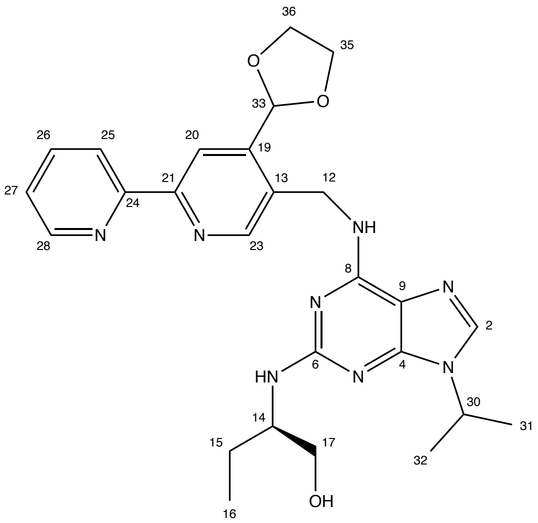


**WX4 (27)**

Derivative WX4 was commercially synthesized by Wuxi AppTec (Shanghai, China). The final compound was isolated as a free base. $^1$H NMR (600 MHz, $CD_3CN$): δ 8.74 (s, 1H, $H_{20}$), 8.66 (m, 1H, $H_{28}$), 8.52 (s, 1H, $H_{23}$), 8.37 (m, 1H, $H_{25}$), 7.86 (m, 1H, $H_{26}$), 7.57 (s, 1H, $H_2$), 7.37 (m, 1H, $H_{27}$), 6.56 (s, broad, NH), 6.20 (s, 1H, $H_{33}$), 5.15 (s, 1H, imp), 4.87 (s, 2H, $H_{12}$), 4.54 (m, 1H, $H_{30}$), 4.13 (m, 4H, $H_{35}$, $H_{36}$), 3.85 (m, 1H, $H_{14}$), 3.52 (m, 2H, $H_{17}$), 1.62 (m, 2H, $H_{15}$), 1.48 (d, $J$ = 6.8 Hz, 6H, $H_{31}$, $H_{32}$), 0.91 (t, $J$ = 7.5 Hz, 3H, $H_{16}$). $^{13}$C NMR (151 MHz, $CD_3CN$): δ 160.4 ($C_{24}$), 156.5 ($C_8$), 156.2 ($C_{21}$), 155.5 ($C_4$), 151.2 ($C_{23}$), 150.2 ($C_{28}$), 146.0 ($C_{19}$), 138.0 ($C_{26}$), 136.4 ($C_2$), 134.6 ($C_{13}$), 124.9 ($C_{25}$, $C_{27}$), 121.5 ($C_{20}$), 115.4 ($C_9$), 101.3 ($C_{33}$), 66.3 ($C_{35}$, $C_{36}$), 65.7 ($C_{17}$), 55.9 ($C_{14}$), 47.3 ($C_{30}$), 25.2 ($C_{15}$), 22.4 ($C_{31}$, $C_{32}$), 11.1 ($C_{16}$). HRMS (m/z): $[M+H]^+$ calcd. 505.2670, found 505.2673; $[M+Na]^+$ calcd. 527.2490, found 527.2490; $[M+2H]^{2+}$ calcd. 253.1371, found 253.1381.

**(*R*)-2-((6-((4-(2-hydroxyethoxy)benzyl)amino)-9-isopropyl-9*H*-purin-2-yl)amino)butan-1-ol (DS61)**

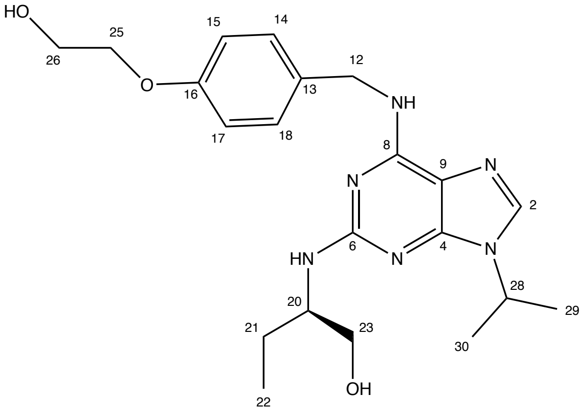


**DS61 (79)**

Derivative DS61 was prepared as per procedures B and D. UHPLC purification was performed at a detection wavelength of 250 nm. $^{1}$H NMR (600 MHz, $CD_3CN$): δ 7.83 (s, 1H, $H_2$), 7.32 (m, 2H, $H_{14}$, $H_{18}$), 6.89 (m, 2H, $H_{15}$, $H_{17}$), 5.20 (s, broad, OH/NH), 4.87 (m, 2H, $H_{12}$), 4.00 (m, 3H, $H_{25}$, $H_{28}$), 3.78 (m, 2H, $H_{26}$), 3.59 (m, 2H, $H_{23}$), 1.66 (m, 2H, $H_{21}$), 1.52 (d, $J$ = 6.8 Hz, 6H, $H_{29}$, $H_{30}$), 0.95 (m, 3H, $H_{22}$). $^{13}$C NMR (151 MHz, $CD_3CN$): δ 152.5 ($C_4$), 139.7 ($C_2$), 130.0 ($C_{14}$, $C_{18}$), 116.2 ($C_9$), 115.5 ($C_{15}$, $C_{17}$), 70.5 ($C_{25}$), 67.6 (imp), 66.2 (imp), 64.2 ($C_{23}$), 61.3 ($C_{26}$), 55.7 ($C_{20}$), 24.7 ($C_{21}$), 22.1 ($C_{29}$, $C_{30}$), 10.8 ($C_{22}$). HRMS (m/z): $[M+H]^+$ calcd. 415.2452, found 415.2459; $[M+Na]^+$ calcd. 437.2272, found 437.2273; $[M+K]^+$ calcd. 453.2011, found 453.2005.

**2-(4-(((9-(1-methyl-1*H*-pyrazol-4-yl)-2-morpholino-9*H*-purin-6yl)amino)methyl)phenoxy)ethan-1-ol (DS62)**

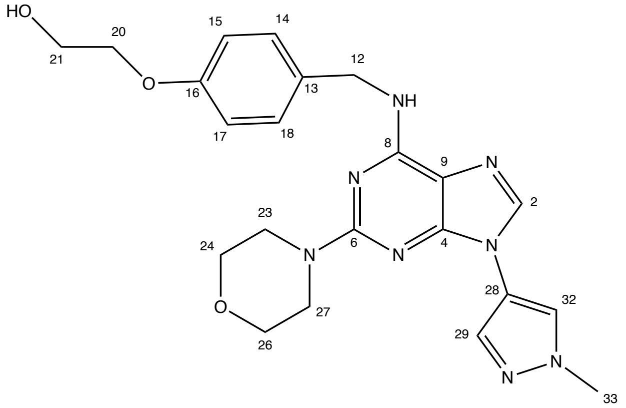


**DS62 (80)**

Derivative DS62 was prepared as per procedures C and D. UHPLC purification was performed at a detection wavelength of 230 nm. $^{1}$H NMR (600 MHz, $CD_3CN$): δ 8.34 (s, 1H, $H_{29}$), 8.13 (s, 1H, $H_{32}$), 7.86 (s, 1H, $H_2$), 7.33 (m, 2H, $H_{14}$, $H_{18}$), 6.87 (m, 2H, $H_{15}$, $H_{17}$), 5.22 (s, broad, OH/NH), 4.67 (m, 2H, $H_{12}$), 3.99 (m, 2H, $H_{20}$), 3.93 (s, 3H, $H_{33}$), 3.75 (m, 6H, $H_{21}$, $H_{24}$, $H_{26}$), 3.68 (m, 4H, $H_{23}$, $H_{27}$). $^{13}$C NMR

(151 MHz, $CD_3CN$): δ 132.7 ($C_2$), 130.1 ($C_{14}$, $C_{18}$), 125.3 ($C_{32}$), 117.1 ($C_9$), 115.4 ($C_{15}$, $C_{17}$), 70.4 ($C_{20}$), 67.4 ($C_{24}$, $C_{26}$), 61.3 ($C_{21}$), 45.7 ($C_{23}$, $C_{27}$), 44.0 ($C_{12}$), 40.1 ($C_{33}$). HRMS (m/z): $[M+H]^+$ calcd. 451.2201, found 451.2206; $[M+Na]^+$ calcd. 473.2020, found 473.2013; $[M+K]^+$ calcd. 489.1759, found 489.1755.

**(*R*)-2-((6-(((4,5-difluoro-1*H*-benzo[*d*]imidazol-2-yl)methyl)amino)-9-isopropyl-9*H*-purin-2-yl)amino)butan-1-ol (DS64)**

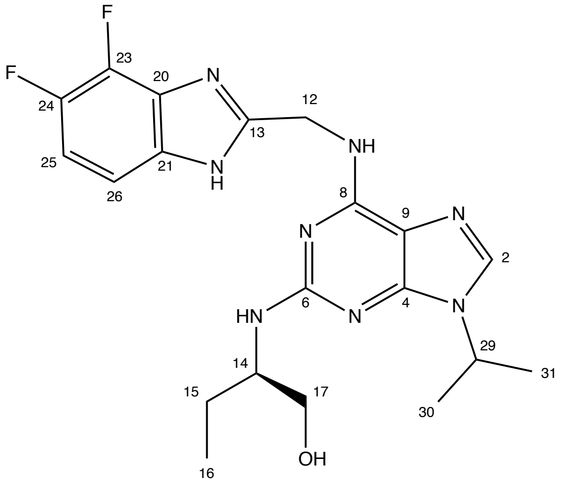

**DS64 (74)**

Derivative DS64 was prepared as per procedures B and D. UHPLC purification was performed at a detection wavelength of 235 nm. $^1H$ NMR (600 MHz, $CD_3CN$): δ 8.03 (s, 1H, $H_2$), 7.33 (m, 1H, $H_{26}$), 7.22 (m, 1H, $H_{25}$), 5.16 (s, broad, OH/NH), 5.02 (m, 2H, $H_{12}$), 4.68 (m, 1H, $H_{29}$), 3.98 (m, 1H, $H_{14}$), 1.61 (m, 2H, $H_{15}$), 1.52 (d, $J$ = 6.8 Hz, 6H, $H_{30}$, $H_{31}$), 0.96 (m, 3H, $H_{16}$). $^{13}C$ NMR (151 MHz, $CD_3CN$): δ 140.3 ($C_{24}$), 135.3 ($C_2$), 133.7 ($C_{23}$), 116.2 ($C_9$), 113.2 ($C_{26}$), 109.9 ($C_{25}$), 65.9 ($C_{17}$), 61.0 (imp), 56.6 ($C_{14}$), 49.2 ($C_{29}$), 24.6 ($C_{15}$), 22.1 ($C_{30}$, $C_{31}$), 10.8 ($C_{16}$). HRMS (m/z): $[M+H]^+$ calcd. 431.2114, found 431.2118; $[M+Na]^+$ calcd. 453.1933, found 453.1933; $[M+K]^+$ calcd. 469.1673, found 469.1668.

**(*R*)-2-((6-((3-(3,4-dichlorophenyl)propyl)amino)-9-isopropyl-9*H*-purin-2-yl)amino)butan-1-ol (DS65)**

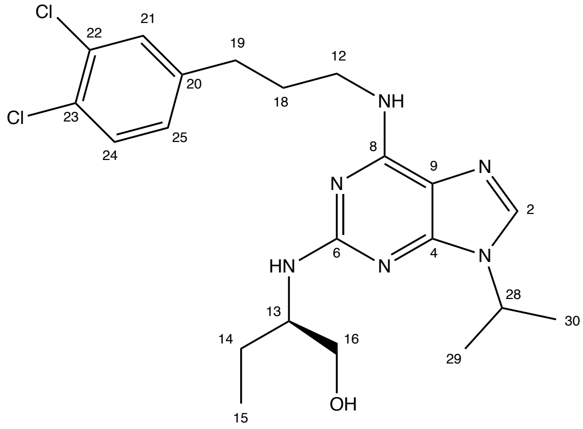


**DS65 (32)**

Derivative DS65 was prepared as per procedures B and D. UHPLC purification was performed at a detection wavelength of 230 nm. $^1$H NMR (600 MHz, $CD_3CN$): δ 7.79 (s, 1H, $H_2$), 7.39 (m, 2H, $H_{21}$, $H_{24}$), 7.15 (m, 1H, $H_{25}$), 4.64 (s, 1H, $H_{28}$), 3.97 (m, 3H, $H_{12}$, $H_{13}$), 3.61 (m, 2H, $H_{16}$), 2.71 (m, 2H, $H_{19}$), 1.99 (m, 2H, $H_{18}$), 1.62 (m, 2H, $H_{14}$), 1.52 (d, $J$ = 6.6 Hz, 6H, $H_{29}$, $H_{30}$), 0.96 (m, 3H, $H_{15}$). $^{13}$C NMR (151 MHz, $CD_3CN$): δ 139.6 ($C_2$), 132.4 ($C_{22}$), 131.4 ($C_{23}$), 131.2 ($C_{24}$), 130.0 ($C_{21}$), 129.6 ($C_{25}$), 116.0 ($C_9$), 65.9 ($C_{16}$), 61.0 (imp), 56.6 (imp), 55.7 ($C_{13}$), 49.5 ($C_{28}$), 44.0 ($C_{12}$), 32.3 (n/a), 31.5 ($C_{19}$), 30.8 ($C_{18}$), 24.7 ($C_{14}$), 22.2 ($C_{29}$, $C_{30}$), 10.8 ($C_{15}$). HRMS (m/z): $[M+H]^+$ calcd. 451.1774, found 451.1784; $[M+Na]^+$ calcd. 473.1594, found 473.1588; $[M+K]^+$ calcd. 489.1333, found 489.1329.

**(*R*)-2-((6-((3-(3,4-dimethylphenyl)propyl)amino)-9-isopropyl-9*H*-purin-2-yl)amino)butan-1-ol (DS66)**

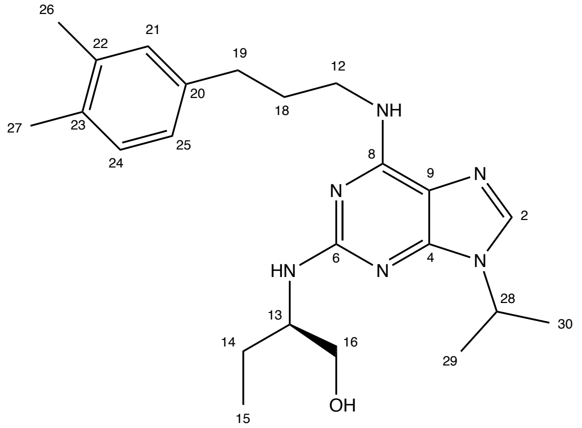


**DS66 (31)**

Derivative DS66 was prepared as per procedures B and D. UHPLC purification was performed at a detection wavelength of 255 nm. $^1$H NMR (600 MHz, $CD_3CN$): δ 7.80 (s, 1H, $H_2$), 6.99 (m, 2H, $H_{21}$, $H_{24}$), 6.92 (m, 1H, $H_{25}$), 4.66 (m, 1H, $H_{28}$), 3.96 (m, 3H, $H_{12}$, $H_{13}$), 3.58 (m, 2H, $H_{16}$), 2.63 (m, 2H, $H_{19}$),

2.18 (s, 6H, $H_{26}$, $H_{27}$), 1.61 (m, 2H, $H_{14}$), 1.52 (d, $J$ = 6.9 Hz, 6H, $H_{29}$, $H_{30}$), 0.95 (m, 3H, $H_{15}$). $^{13}$C NMR (151 MHz, $CD_3CN$): δ 139.8 ($C_2$), 137.4 ($C_{22}$), 134.9 ($C_{23}$), 130.7 ($C_{21}$), 130.4 ($C_{24}$), 126.7 ($C_{25}$), 116.1 ($C_9$), 65.8 ($C_{16}$), 55.7 ($C_{13}$), 49.4 ($C_{28}$), 44.3 ($C_{12}$), 32.7 ($C_{18}$/$C_{19}$), 32.2 ($C_{18}$/$C_{19}$), 24.7 ($C_{14}$), 22.2 ($C_{29}$, $C_{30}$), 19.8 ($C_{26}$), 19.3 ($C_{27}$), 10.9 ($C_{15}$). HRMS (m/z): $[M+H]^+$ calcd. 411.2867, found 411.2870.

**(*R*)-2-((6-((3-(3-ethylphenyl)propyl)amino)-9-isopropyl-9*H*-purin-2-yl)amino)butan-1-ol (DS67)**

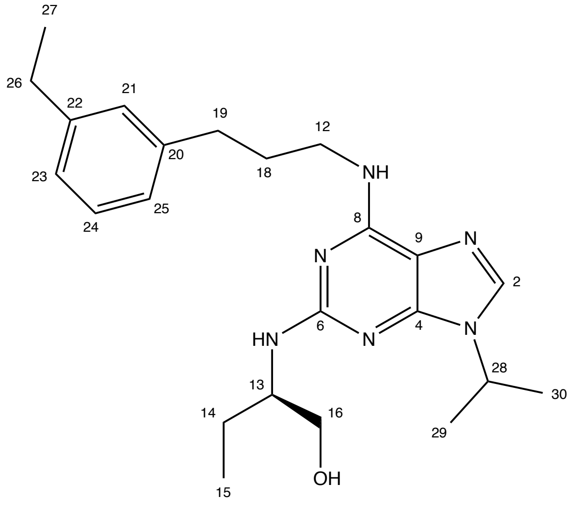


**DS67 (33)**

Derivative DS67 was prepared as per procedures B and D. UHPLC purification was performed at a detection wavelength of 200 nm. $^1$H NMR (600 MHz, $CD_3CN$): δ 7.80 (s, 1H, $H_2$), 7.16 (m, 1H, $H_{24}$), 7.03 (m, 3H, $H_{21}$, $H_{23}$, $H_{25}$), 6.60 (s, broad, NH), 4.64 (m, 1H, $H_{28}$), 3.97 (m, 3H, $H_{12}$, $H_{13}$), 3.60 (m, 2H, $H_{16}$), 2.69 (m, 2H, $H_{26}$), 2.57 (m, 2H, $H_{19}$), 1.98 (m, 2H, $H_{18}$), 1.64 (m, 2H, $H_{14}$), 1.52 (d, $J$ = 6.6 Hz, 6H, $H_{29}$, $H_{30}$), 1.16 (m, 3H, $H_{27}$), 0.95 (m, 3H, $H_{15}$). $^{13}$C NMR (151 MHz, $CD_3CN$): δ 145.4 ($C_{22}$), 142.6 ($C_{20}$), 139.6 ($C_2$), 129.3 ($C_{24}$), 129.0 ($C_{21}$), 126.6 ($C_{25}$), 126.3 ($C_{23}$), 116.1 ($C_9$), 65.9 ($C_{16}$), 55.7 ($C_{13}$), 49.4 ($C_{28}$), 44.4 ($C_{12}$), 33.3 ($C_{26}$), 32.2 ($C_{18}$), 29.4 ($C_{19}$), 24.7 ($C_{14}$), 22.2 ($C_{29}$, $C_{30}$), 16.1 ($C_{27}$), 10.8 ($C_{15}$). HRMS (m/z): $[M+H]^+$ calcd. 411.2867, found 411.2871.

**(*R*)-3-(3-((2-((1-hydroxybutan-2-yl)amino)-9-isopropyl-9*H*-purin-6-yl)amino)propyl)phenol (DS68)**

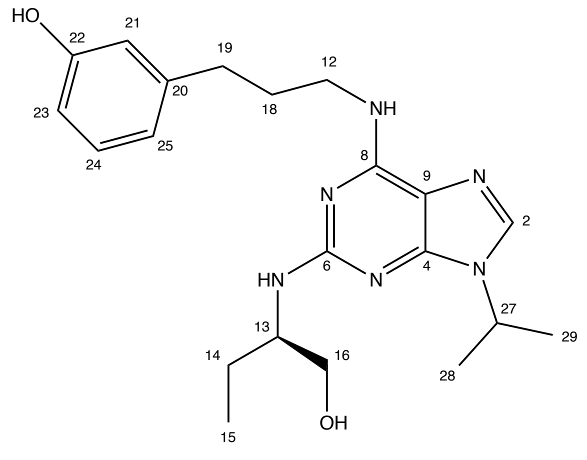


**DS68 (37)**

Derivative DS68 was prepared as per procedures B and D. UHPLC purification was performed at a detection wavelength of 205 nm. $^1$H NMR (600 MHz, $CD_3CN$): δ 7.84 (s, 1H, $H_2$), 7.15 (t, $J$ = 7.8 Hz, 1H, $H_{24}$), 6.77 (d, $J$ = 7.5 Hz, 1H, $H_{23}$), 6.74 (s, 1H, $H_{21}$), 6.69 (m, 1H, $H_{25}$), 4.54 (m, 1H, $H_{27}$), 3.93 (m, 3H, $H_{12}$, $H_{13}$), 3.59 (m, 2H, $H_{16}$), 1.59 (m, 2H, $H_{14}$), 1.26 (d, $J$ = 6.1 Hz, 6H, $H_{28}$, $H_{29}$), 0.93 (m, 3H, $H_{15}$). $^{13}$C NMR (151 MHz, $CD_3CN$): δ 159.1 ($C_{22}$), 154.0 ($C_4$), 144.6 ($C_{20}$), 141.6 ($C_2$), 130.4 ($C_{24}$), 121.5 ($C_{25}$), 116.9 ($C_9$, $C_{21}$), 114.0 ($C_{23}$), 70.3 ($C_{27}$), 64.1 ($C_{16}$), 56.4 ($C_{13}$), 47.4 ($C_{27}$), 43.3 ($C_{12}$), 41.6 (imp), 33.8 ($C_{19}$), 31.4 ($C_{18}$), 24.7 ($C_{14}$), 22.3 ($C_{28}$, $C_{29}$), 10.9 ($C_{15}$). HRMS (m/z): $[M+H]^+$ calcd. 399.2503, found 399.2502; $[M+Na]^+$ calcd. 421.2322, found 421.2317.

**(*R*)-2-((9-isopropyl-6-((3-(6-methylpyridin-2-yl)propyl)amino)-9*H*-purin-2-yl)amino)butan-1-ol (DS69)**

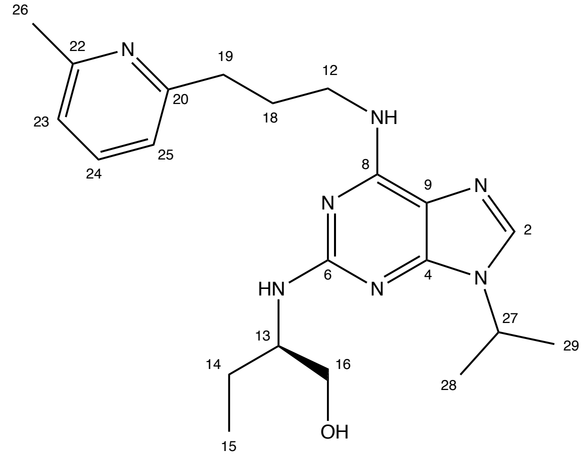


**DS69 (35)**

Derivative DS69 was prepared as per procedures B and D. UHPLC purification was performed at a detection wavelength of 320 nm. $^1$H NMR (600 MHz, $CD_3CN$): δ 8.20 (m, 1H, $H_{24}$), 7.58 (m, 2H, $H_{23}$,

$H_{25}$), 6.53 (s, broad, NH), 4.65 (m, 1H, $H_{27}$), 4.00 (m, 3H, $H_{12}$, $H_{13}$), 3.61 (m, 2H, $H_{16}$), 3.14 (m, 2H, $H_{19}$), 2.69 (s, 3H, $H_{26}$), 2.16 (m, 2H, $H_{18}$), 1.58 (m, 2H, $H_{14}$), 1.52 (d, $J$ = 7.1 Hz, 6H, $H_{28}$, $H_{29}$), 0.96 (m, 3H, $H_{15}$). $^{13}$C NMR (151 MHz, $CD_3CN$): δ 155.0 ($C_{22}$), 152.5 ($C_4$), 150.4 ($C_6$), 146.9 ($C_{24}$), 140.0 ($C_2$), 126.2 ($C_{25}$), 125.1 ($C_{23}$), 116.4 ($C_9$), 65.8 ($C_{16}$), 55.8 ($C_{13}$), 49.5 ($C_{27}$), 43.2 ($C_{12}$), 29.8 ($C_{19}$), 28.9 ($C_{18}$), 24.8 ($C_{14}$), 22.1 ($C_{28}$, $C_{29}$), 19.7 ($C_{26}$), 10.9 ($C_{15}$). HRMS (m/z): $[M+H]^+$ calcd. 398.2663, found 398.2669; $[M+Na]^+$ calcd. 420.2482, found 420.2477; $[M+K]^+$ calcd. 436.2222, found 436.2214.

**(*S*)-2-(1-(9-isopropyl-6-((4-(pyridin-2-yl)benzyl)amino)-9*H*-purin-2-yl)piperidin-2-yl)ethan-1-ol (DS70)**

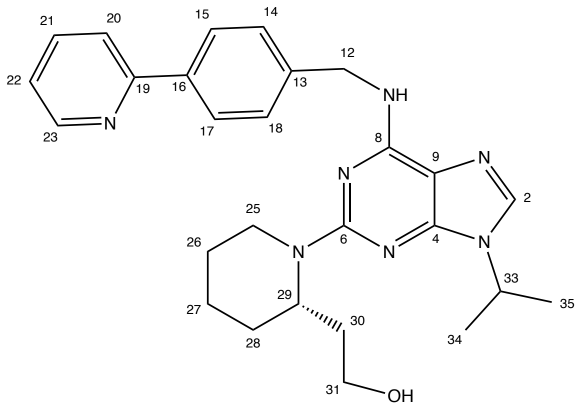


**DS70 (45)**

Derivative DS70 was prepared as per procedures B and E. To assist with dissolving the primary amine, DMF was added at a 1:1 volumetric ratio to the base. UHPLC purification was performed at a detection wavelength of 205 nm. $^1$H NMR (600 MHz, $CD_3CN$): δ 8.80 (m, 1H, $H_{23}$), 8.28 (m, 1H, $H_{21}$), 8.06 (m, 1H, $H_{20}$), 7.93 (m, 3H, $H_2$, $H_{15}$, $H_{17}$), 7.69 (m, 1H, $H_{22}$), 7.61 (m, 2H, $H_{14}$, $H_{18}$), 4.83 (m, 2H, $H_{12}$), 4.70 (s, 1H, $H_{33}$), 3.97 (s, 1H, imp), 3.60 (m, 2H, $H_{31}$), 3.12 (m, 3H, $H_{25}$, $H_{29}$), 1.66 (m, 4H, $H_{28}$, $H_{30}$), 1.55 (m, 4H, $H_{26}$, $H_{27}$), 1.52 (d, $J$ = 6.7 Hz, 6H, $H_{34}$, $H_{35}$). $^{13}$C NMR (151 MHz, $CD_3CN$): δ 144.9 ($C_{23}$), 143.2 ($C_{21}$), 133.9 (imp), 128.3 ($C_{14}$, $C_{18}$), 128.0 ($C_2$, $C_{15}$, $C_{17}$), 124.2 ($C_{20}$, $C_{22}$), 63.0 ($C_{31}$), 55.2 (imp), 48.7 ($C_{33}$), 46.7 ($C_{25}$, $C_{29}$), 43.6 ($C_{12}$), 32.1 ($C_{30}$), 25.4 ($C_{28}$), 21.5 ($C_{34}$, $C_{35}$), 20.9 ($C_{26}$, $C_{27}$). HRMS (m/z): $[M+H]^+$ calcd. 472.2819, found 472.2823; $[M+K]^+$ calcd. 510.2378, found 510.2369.

**8-bromo-*N*-((4,5-difluoro-1*H*-benzo[*d*]imidazol-2-yl)methyl)-2-(4-methylpiperazin-1-yl)pyrazolo[1,5-*a*][1,3,5]triazin-4-amine (DS71)**

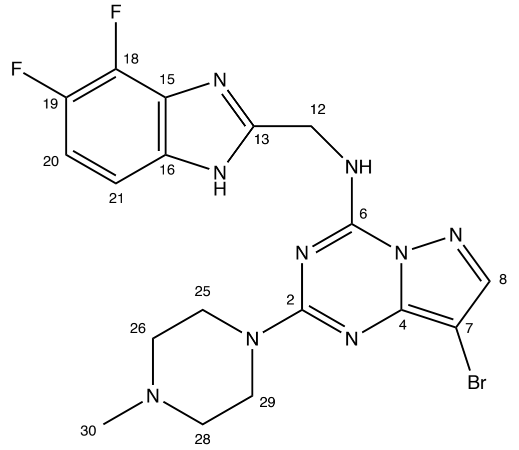


**DS71 (73)**

Derivative DS71 was prepared using a modified version of the procedures. First for procedure A, 8-bromo-4-chloro-2-(methylthio)pyrazolo[1,5-a][1,3,5]triazine was utilized as the starting material. All other parameters for procedure A were kept constant. Procedure B was removed and replaced with the following protocol. A 8.5 mM solution was made in dichloromethane of the product from procedure A. The solution was cooled to 0 °C and then a 3x molar excess of *meta*-chloroperoxybenzoic acid (mCPBA)was added to the flask and allowed to stir at room temperature for 2 h. Afterwards, the organic phase was washed with 15 mL of saturated $Na_2CO_3$ (2 times). The organic phase was rotoevaporated and the crude product was utilized in the next step. Procedure D was performed as reported above. UHPLC purification was performed at a detection wavelength of 200 nm. $^1$H NMR (600 MHz, $CD_3CN$): δ 7.86 (s, 1H, $H_8$), 7.26 (d, $J$ = 8.8 Hz, 1H, $H_{20}$), 7.15 (m, 1H, $H_{21}$), 5.00 (m, 2H, $H_{12}$), 4.73 (m, 2H, imp), 3.31 (m, 4H, $H_{25}$, $H_{29}$). $^{13}$C NMR (151 MHz, $CD_3CN$): δ 158.0 ($C_6$), 150.3 ($C_{19}$), 148.8 ($C_{13}$), 146.6 ($C_{18}$), 144.8 ($C_8$), 131.1 ($C_{15}$), 112.8 ($C_{21}$), 109.3 ($C_{20}$), 94.7 ($C_7$), 78.9 (imp), 53.5 ($C_{25}$, $C_{29}$), 43.6 ($C_{30}$), 42.0 (imp), 39.4 ($C_{12}$). HRMS (m/z): $[M+H]^+$ calcd. 478.0909, found 478.0909.

**8-bromo-*N*-((4,5-difluoro-1*H*-benzo[*d*]imidazol-2-yl)methyl)-2-morpholinopyrazolo[1,5-*a*][1,3,5]triazin-4-amine (DS72)**

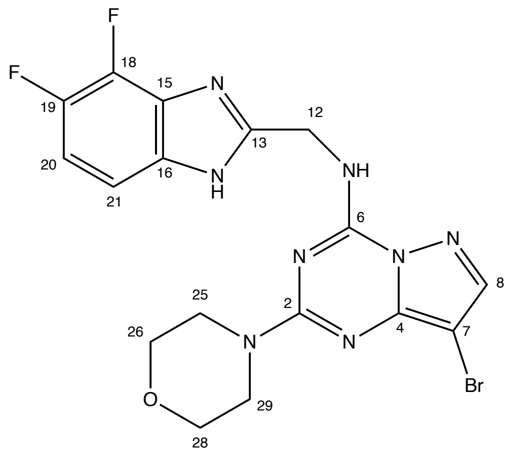


**DS72 (71)**

Derivative DS72 was prepared using a modified version of the procedures. First for procedure A, 8-bromo-4-chloro-2-(methylthio)pyrazolo[1,5-a][1,3,5]triazine was utilized as the starting material. All other parameters for procedure A were kept constant. Procedure B was removed and replaced with the following protocol. A 8.5 mM solution was made in dichloromethane of the product from procedure A. The solution was cooled to 0 °C and then a 3x molar excess of *meta*-chloroperoxybenzoic acid (mCPBA)was added to the flask and allowed to stir at room temperature for 2 h. Afterwards, the organic phase was washed with 15 mL of saturated $Na_2CO_3$ (2 times). The organic phase was rotoevaporated and the crude product was utilized in the next step. Procedure D was performed as reported above. UHPLC purification was performed at a detection wavelength of 200 nm. $^1$H NMR (600 MHz, $CD_3CN$): δ 7.81 (m, 1H, $H_8$), 7.72 (s, broad, NH), 7.24 (m, 1H, $H_{21}$), 7.14 (m, 1H, $H_{20}$), 4.99 (m, 2H, $H_{12}$), 3.69 (m, 4H, $H_{25}$, $H_{29}$), 3.57 (m, 4H, $H_{26}$, $H_{28}$). $^{13}$C NMR (151 MHz, $CD_3CN$): δ 158.4 ($C_6$), 150.1 ($C_{19}$), 149.0 ($C_{13}$), 146.3 ($C_{18}$), 144.6 ($C_8$), 112.9 ($C_{21}$), 109.2 ($C_{20}$), 78.1 (imp), 67.0 ($C_{26}$, $C_{28}$), 45.4 ($C_{25}$, $C_{29}$), 39.3 ($C_{12}$). HRMS (m/z): $[M+H]^+$ calcd. 465.0593, found 465.0596; $[M+Na]^+$ calcd. 487.0412, found 487.0403.

**8-bromo-*N*-((5,6-dimethyl-1*H*-benzo[*d*]imidazol-2-yl)methyl)-2-(4-methylpiperazin-1-yl)pyrazolo[1,5-*a*][1,3,5]triazin-4-amine (DS73)**

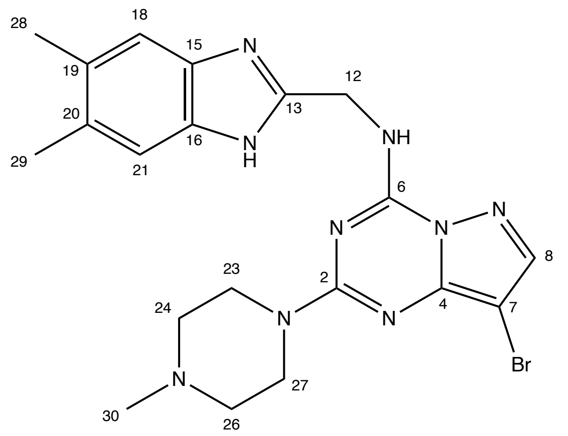


**DS73 (72)**

Derivative DS73 was prepared using a modified version of the procedures. First for procedure A, 8-bromo-4-chloro-2-(methylthio)pyrazolo[1,5-a][1,3,5]triazine was utilized as the starting material. All other parameters for procedure A were kept constant. Procedure B was removed and replaced with the following protocol. A 8.5 mM solution was made in dichloromethane of the product from procedure A. The solution was cooled to 0 °C and then a 3x molar excess of *meta*-chloroperoxybenzoic acid (mCPBA) was added to the flask and allowed to stir at room temperature for 2 h. Afterwards, the organic phase was washed with 15 mL of saturated $Na_2CO_3$ (2 times). The organic phase was rotoevaporated and the crude product was utilized in the next step. Procedure D was performed as reported above. UHPLC purification was performed at a detection wavelength of 235 nm. $^1$H NMR (600 MHz, $CD_3CN$): δ 8.45 (s, broad, NH), 7.84 (s, 1H, $H_8$), 7.49 (s, 2H, $H_{18}$, $H_{21}$), 5.19 (m, 2H, $H_{12}$), 4.69 (m, 2H, imp), 3.28 (m, 4H, $H_{23}$, $H_{27}$), 2.76 (m, 4H, $H_{24}$, $H_{26}$), 2.69 (s, 3H, $H_{30}$), 2.37 (s, 6H, $H_{28}$, $H_{29}$). $^{13}$C NMR (151 MHz, $CD_3CN$): δ 157.8 ($C_6$), 150.7 ($C_{13}$), 150.2 (n/a), 148.7 ($C_4$), 146.7 ($C_8$), 137.0 ($C_{15}$, $C_{16}$), 130.8 ($C_{19}$, $C_{20}$), 114.7 ($C_{18}$, $C_{21}$), 78.9 (imp), 53.5 ($C_{23}$, $C_{24}$, $C_{26}$, $C_{27}$), 43.7 ($C_{30}$), 42.2 (imp), 37.4 ($C_{12}$), 20.4 ($C_{28}$, $C_{29}$). HRMS (m/z): $[M+H]^+$ calcd. 470.1411, found 470.1407.

***N*-((5,6-dichloro-1*H*-benzo[*d*]imidazol-2-yl)methyl)-8-(1-methyl-1*H*-pyrazol-4-yl)-2-morpholinopyrazolo[1,5-*a*][1,3,5]triazin-4-amine (DS74)**

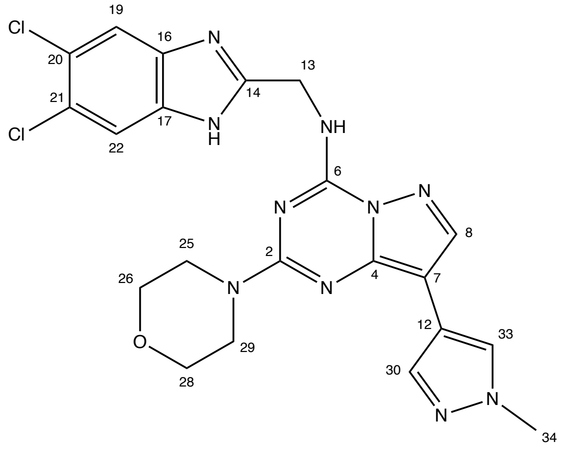

**DS74 (70)**

Derivative DS74 was commercially synthesized by Wuxi AppTec (Shanghai, China). The final compound was isolated as a free base. $^{1}$H NMR (600 MHz, $CD_3CN$): δ 12.62 (s, NH), 9.04 (s, NH), 8.21 (s, 1H, $H_{30}$), 7.99 (s, 1H, $H_{33}$), 7.82 (s, 2H, $H_8$, $H_{19}$), 7.67 (s, 1H, $H_{22}$), 4.87 (m, 2H, $H_{13}$), 3.86 (s, 3H, $H_{34}$), 3.64 (s, 4H, $H_{25}$, $H_{29}$), 3.52 (s, 4H, $H_{26}$, $H_{28}$). $^{13}$C NMR (151 MHz, $CD_3CN$): δ 156.4 ($C_6$), 155.0 ($C_{14}$), 148.8 ($C_{16}$, $C_{17}$), 145.9 ($C_4$), 142.7 ($C_{30}$), 135.3 ($C_8$), 126.1 ($C_{33}$), 123.8 ($C_{20}$, $C_{21}$), 119.6 ($C_{19}$), 112.9 ($C_{12}$, $C_{22}$), 97.9 ($C_7$), 65.9 ($C_{26}$, $C_{28}$), 44.1 ($C_{25}$, $C_{29}$), 38.7 ($C_{13}$), 38.5 ($C_{34}$). HRMS (m/z): $[M+Na]^+$ calcd. 521.1091, found 521.1083.

## References


[1] Oumata, N.; Ferandin, Y.; Meijer, L. ; Galons, H. *Organic Process Research & Development,* **2009**, *13,* 641.

[2] Monastyrskyi, A. Nilchan, N.; Quereda, V.; Noguchi, Y.; Ruiz, C.; Grant, W.; Cameron, M.; Duckett, D.; Roush. *Bioorganic & Medicinal Chemistry,* **2018**, *26,* 590.

[3] Larsen, A.F.; Ulven, T. *Chemical Communications* **2014**, *50,* 4997.

**Supplementary Table 2.** Summary of all assay data for the evaluated compounds.

| Name | Structure | TR-FRET | Cellular assays | | | | Lanthascreen $IC_{50}$ [nM] | | | Other experiments | | | # | Ref. |
|---|---|---|---|---|---|---|---|---|---|---|---|---|---|---|
| | | $EC_{50}$ TR-FRET [nM] | $DC_{50}$ [nM] | $EC_{50}$ [nM] | $EC_{50}$ (+MLN) [nM] | Toxicity rescue | CDK12 | CDK2 | CDK9 | Proteomics | RNA-seq | Crystal structure | | |
| **CR8** | 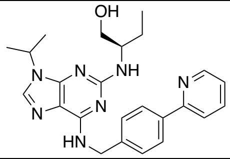 | 16.36 | 24 | 159 | 703 | 342 % | 172 | 86 | 44 | ✓ | ✓ | ✓ | 1 | [1] |
| **DS01** | 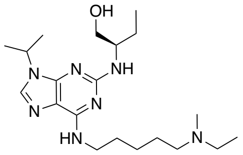 | 297.3 | n.d. | 424 | 1774 | 318 % | | | | | | | 8 | This work |
| **DS02** | 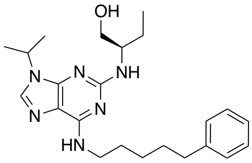 | 285.4 | 1775 | n.d. | n.d. | 0 % | | | | | | | 18 | This work |
| **DS03** | 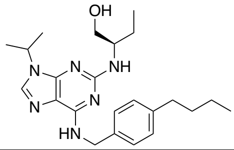 | 184.8 | n.d. | n.d. | n.d. | 0 % | | | | | | | 7 | This work |
| **DS04** | 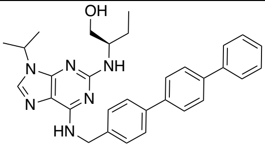 | n.d. | n.d. | n.d. | n.d. | 0 % | | | | | | | 22 | This work |
| **DS05** | 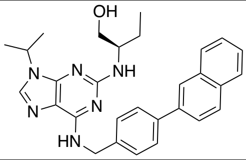 | 935.5 | n.d. | n.d. | n.d. | 0 % | | | | | | | 21 | This work |
| **DS06** | 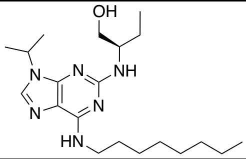 | 1162 | n.d. | n.d. | n.d. | 0 % | | | | | | ✓ | 5 | This work |
| **DS07** | 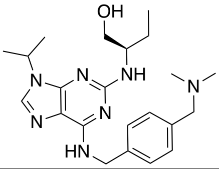 | 285.4 | n.d. | n.d. | n.d. | 0 % | | | | | | | 10 | This work |
| **DS08** | 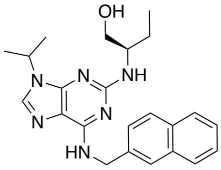 | 60.32 | 724 | 3497 | n.d. | 186 % | | | | | | ✓ | 23 | This work |
| **DS09** | 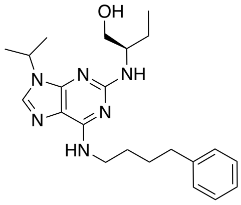 | 349.2 | n.d. | n.d. | n.d. | 0 % | | | | | | | 17 | This work |
| **DS10** | 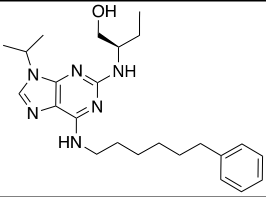 | (595.2) | n.d. | n.d. | n.d. | 0 % | | | | | | | 19 | This work |

| | | | | | | | | | | | | | | |
|---|---|---|---|---|---|---|---|---|---|---|---|---|---|---|
| **DS11** | 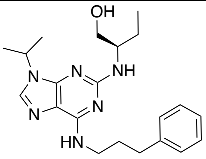 | 56.77 | n.d. | n.d. | n.d. | 0 % | | | | | | ✓ | 3 | This work |
| **DS12** | 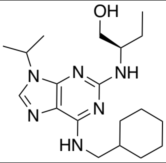 | (774) | n.d. | n.d. | n.d. | 0 % | | | | | | | 15 | This work |
| **DS13** | 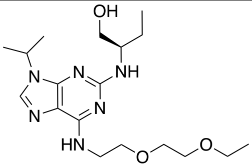 | n.d. | n.d. | n.d. | n.d. | 0 % | | | | | | | 76 | This work |
| **DS14** | 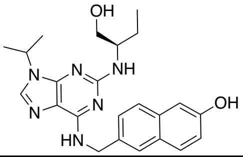 | n.d. | n.d. | n.d. | n.d. | 0 % | | | | | | | 77 | This work |
| **DS15** | 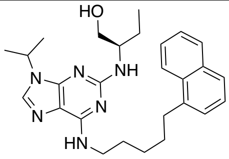 | 182 | n.d. | n.d. | n.d. | 0 % | | | | | | ✓ | 24 | This work |
| **DS16** | 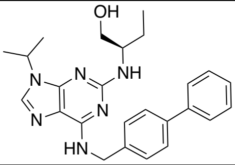 | 16.68 | 1029 | 600 | n.d. | 1567 % | | | | | | ✓ | 2 | This work |
| **DS17** | 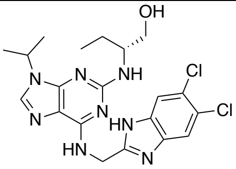 | 13.19 | 12 | 93 | 142 | 53 % | 117 | 10260 | 3061 | ✓ | ✓ | ✓ | 66 | This work |
| **DS18** | 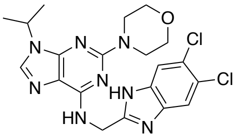 | 8.771 | 13 | 101 | 191 | 89 % | 289 | 5729 | 4482 | | ✓ | ✓ | 65 | This work |
| **DS19** | 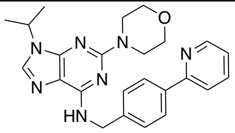 | 14 | 62 | 203 | 813 | 300 % | 163 | 239 | 143 | | ✓ | ✓ | 44 | This work |
| **DS20** | 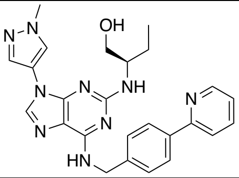 | 1837 | n.d. | n.d. | n.d. | 0 % | n.d. | n.d. | 3195 | | | | 69 | This work |
| **DS21** | 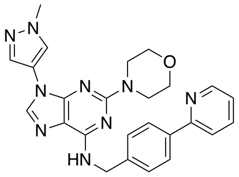 | 299 | 3124 | 4940 | n.d. | 102 % | 2862 | n.d. | 953 | | | | 68 | This work |
| **DS22** | 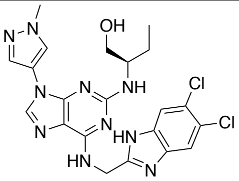 | 19.33 | 109 | 935 | 1549 | 66 % | 1316 | n.d. | n.d. | | | ✓ | 67 | This work |

| | | | | | | | | | | | | | | |
|---|---|---|---|---|---|---|---|---|---|---|---|---|---|---|---|
| **DS23** | 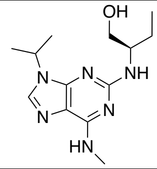 | n.d. | n.d. | n.d. | n.d. | 0 % | | | | | | | 6 | This work |
| **DS24** | 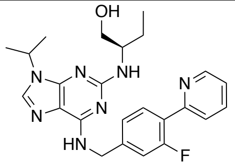 | 78.24 | 172 | 794 | 2941 | 270 % | | | | | | ✓ | 42 | This work |
| **DS25** | 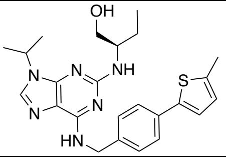 | 14.98 | 90 | 267 | 2390 | 795 % | | | | | ✓ | | 25 | This work |
| **DS27** | 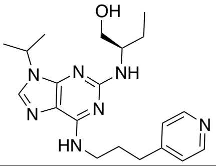 | n.d. | n.d. | n.d. | n.d. | 0 % | | | | | | | 30 | This work |
| **DS28** | 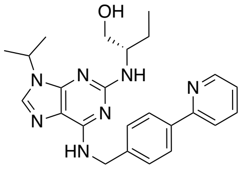 | 31.82 | 27 | 221 | 1569 | 610 % | | | | | | | 43 | (S-CR8) |
| **DS29** | 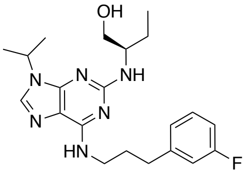 | 45.71 | 253 | 452 | 3676 | 713 % | | | | | | | 36 | This work |
| **DS30** | 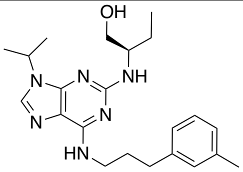 | 34.83 | 78 | 451 | 2688 | 496 % | | | | ✓ | ✓ | ✓ | 34 | This work |
| **DS31** | 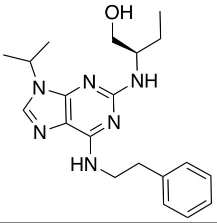 | n.d. | n.d. | n.d. | n.d. | 0 % | | | | | | | 16 | This work |
| **DS32** | 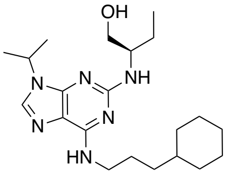 | 675.5 | n.d. | n.d. | n.d. | 0 % | | | | | | | 13 | This work |
| **DS33** | 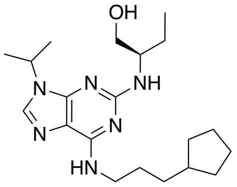 | 1270 | n.d. | n.d. | n.d. | 0 % | | | | | | | 12 | This work |
| **DS34** | 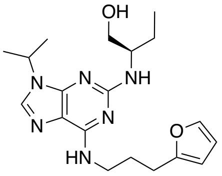 | 603.3 | n.d. | n.d. | n.d. | 0 % | | | | | | | 29 | This work |

| | | | | | | | | | | | | | | |
|---|---|---|---|---|---|---|---|---|---|---|---|---|---|---|
| **DS35** | 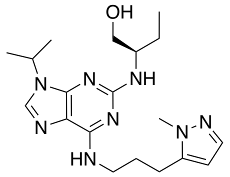 | 245.7 | n.d. | n.d. | n.d. | 0 % | | | | | | | 28 | This work |
| **DS36** | 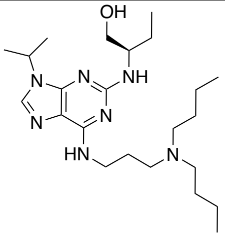 | n.d. | n.d. | n.d. | n.d. | 0 % | | | | | | | 9 | This work |
| **DS37** | 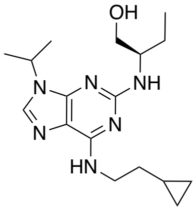 | n.d. | n.d. | n.d. | n.d. | 0 % | | | | | | | 11 | This work |
| **DS38** | 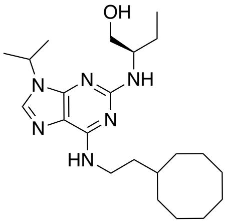 | 5737 | n.d. | n.d. | n.d. | 0 % | | | | | | | 14 | This work |
| **DS40** | 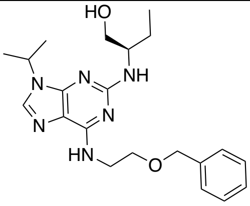 | 1302 | n.d. | n.d. | n.d. | 0 % | | | | | | | 78 | This work |
| **DS43** | 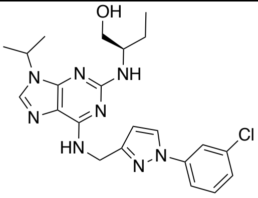 | 43.67 | 75 | 1255 | n.d. | 697 % | | | | | ✓ | ✓ | 26 | This work |
| **DS44** | 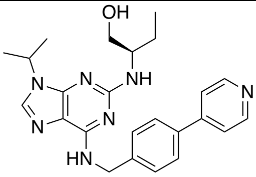 | 35.59 | 62 | 300 | 2870 | 857 % | | | | | | | 39 | This work |
| **DS45** | 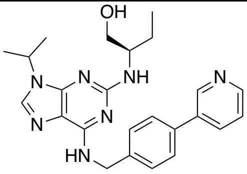 | 45.14 | 36 | 242 | 3394 | 1302 % | | | | | | | 40 | This work |
| **DS46** | 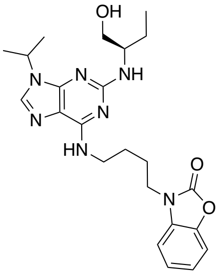 | (627) | * | n.d. | n.d. | 0 % | | | | | | | 20 | This work |

| | | | | | | | | | | | | | | |
|---|---|---|---|---|---|---|---|---|---|---|---|---|---|---|
| **DS47** | 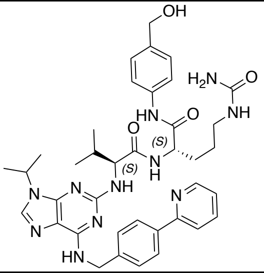 | 8809 | 4390 | n.d. | n.d. | 0 % | | | | | | | 52 | This work |
| **DS48** | 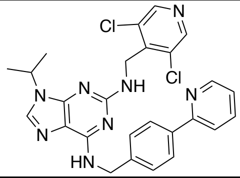 | 45.99 | 366 | 907 | 3742 | 313 % | | | | | | | 46 | This work |
| **DS50** | 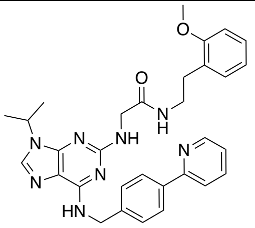 | 1916 | 3569 | n.d. | n.d. | 0 % | | | | | ✓ | ✓ | 49 | This work |
| **DS51** | 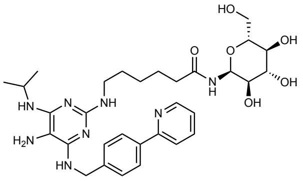 | 274.5 | n.d. | n.d. | n.d. | 0 % | | | | | | | 50 | This work |
| **DS52** | 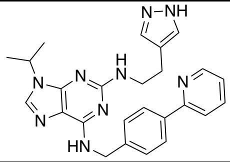 | 71.43 | 125 | 495 | 3261 | 559 % | | | | | | | 47 | This work |
| **DS53** | 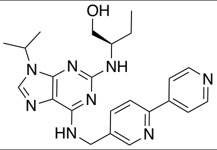 | 27.7 | 260 | 635 | 1522 | 140 % | | | | | | | 38 | This work |
| **DS54** | 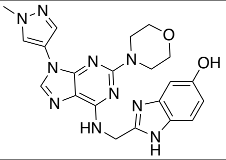 | (252) | n.d. | n.d. | n.d. | 0 % | | | | | | | 64 | This work |
| **DS55** | 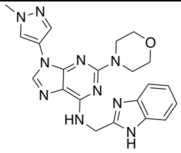 | 35.61 | * | 1026 | 4478 | 336 % | | | | | | ✓ | 61 | This work |
| **DS56** | 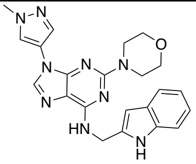 | (2114) | * | ? | ? | ? | | | | | | | 62 | This work |
| **DS57** | 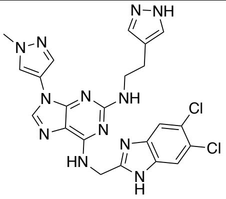 | 72.33 | 587 | n.d. | n.d. | 0 % | | | | | | | 63 | This work |

| | | | | | | | | | | | | | | |
|---|---|---|---|---|---|---|---|---|---|---|---|---|---|---|
| **DS58** | 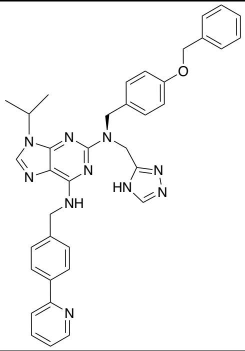 | 947.7 | 2020 | 1784 | n.d. | 461 % | | | | | | | 51 | This work |
| **DS59** | 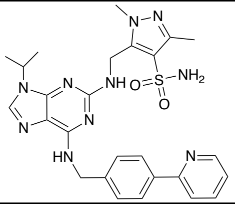 | 101.1 | 566 | 1416 | 4673 | 230 % | | | | | | ✓ | 48 | This work |
| **DS61** | 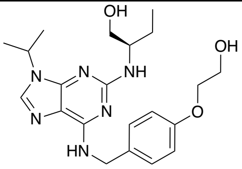 | 168.5 | 917 | 283 | 1070 | 278 % | | | | | | ✓ | 79 | This work |
| **DS62** | 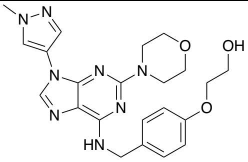 | (2198?) | * | n.d. | n.d. | 0 % | | | | | | | 80 | This work |
| **DS64** | 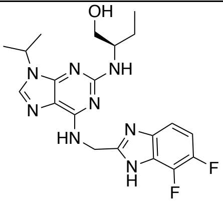 | 40.94 | 62 | 527 | 5643 | 971 % | ? | 4964 | n.d. | | | | 74 | This work |
| **DS65** | 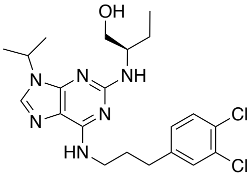 | 20.84 | 103 | 528 | 1657 | 214 % | | | | | | | 32 | This work |
| **DS66** | 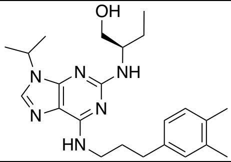 | 17.67 | 88 | n.d. | n.d. | 0 % | | | | | | | 31 | This work |
| **DS67** | 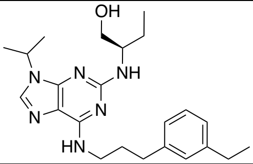 | 30.53 | 219 | 517 | 4752 | 819 % | | | | | | | 33 | This work |
| **DS68** | 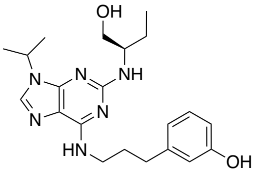 | 103.7 | 571 | 2523 | n.d. | 296 % | | | | | | | 37 | This work |
| **DS69** | 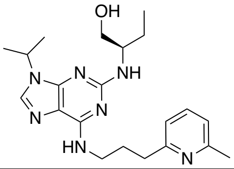 | 35.94 | 217 | 864 | 6037 | 599 % | | | | | | | 35 | This work |

| | | | | | | | | | | | | | | |
|---|---|---|---|---|---|---|---|---|---|---|---|---|---|---|
| **DS70** | 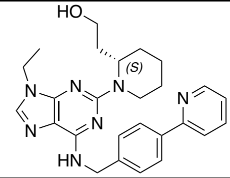 | 17.56 | * | 265 | 345 | 30 % | 1 | 31 | 5 | | | | 45 | This work |
| **DS71**<br>**(P133)** | 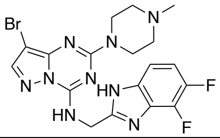 | 27.6 | 4 | 10 | 71 | 610 % | 134 | n.d. | 684 | | | | 73 | 2 |
| **DS72**<br>**(P25)** | 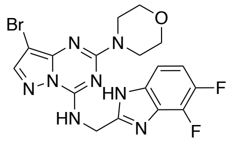 | 12.24 | 6 | 6 | 38 | 533 % | 1 | 1056 | 104 | | | | 71 | 2 |
| **DS73**<br>**(P342)** | 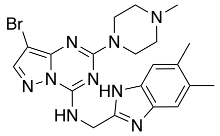 | 15.32 | 1 | 2 | 19 | 850 % | 25 | 6116 | 557 | | | | 72 | 2 |
| **DS74**<br>**(P419)** | 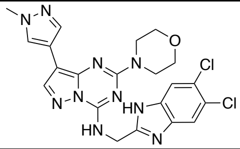 | 19.77 | 51 | 104 | 1057 | 916 % | 25 | n.d. | 2583 | | | | 70 | 2 |
| **WX3** | 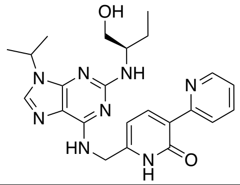 | 21.36 | 23 | 179 | 1887 | 954 % | | | | | | ✓ | 41 | This work |
| **WX4** | 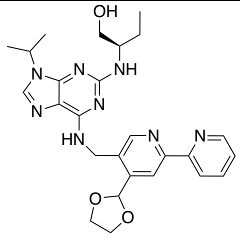 | 78.53 | 365 | 1175 | 6251 | 432 % | | | | | | | 27 | This work |
| **Roscovitine** | 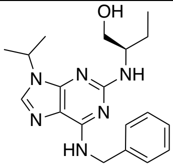 | 702.6 | n.d. | n.d. | n.d. | 0 % | | | | | ✓ | ✓ | 4 | 3 |
| **DRF053** | 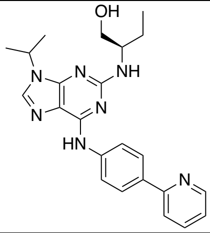 | 703.6 | n.d. | 1310 | 6383 | 387 % | | | | | | ✓ | 55 | 4 |
| **Flavopiridol** | 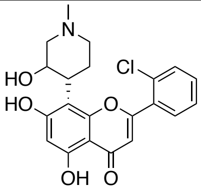 | 51.77 | n.d. | 395 | 400 | 1 % | | | | | | | 53 | 5 |
| **21195** | 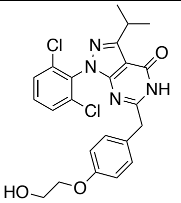 | 12.03 | 26 | 39 | 127 | 226 % | 31 | 3 | 1 | ✓ | ✓ | ✓ | 75 | 6 |

| | | | | | | | | | | | | | | |
|---|---|---|---|---|---|---|---|---|---|---|---|---|---|---|
| **SR-4835** | 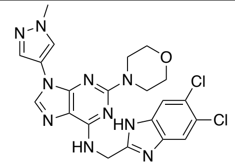 | 15.9 | 14 | 42 | 313 | 645 % | 27 | n.d. | 3664 | ✓ | ✓ | ✓ | 60 | 7 |
| **919278** | 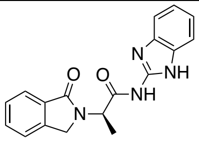 | 37.8 | 176 | 559 | 4748 | 749 % | 1335 | n.d. | 120 | ✓ | ✓ | ✓ | 81 | 8 |
| **dCeMM2** | 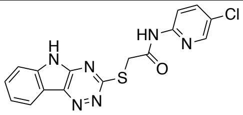 | 83.22 | 633 | 1423 | 7084 | 398 % | n.d. | n.d. | 1113 | | | | 88 | 9 |
| **dCeMM3** | 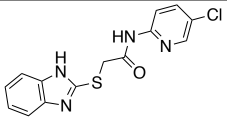 | 263.4 | n.d. | n.d. | n.d. | 0 % | 2666 | n.d. | 3020 | | | ✓ | 85 | 9 |
| **dCeMM4** | 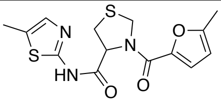 | 278.8 | 1210 | 2626 | n.d. | 281 % | 6547 | n.d. | 2459 | | | ✓ | 89 | 9 |
| **HQ461** | 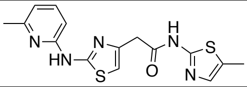 | 42.7 | 493 | 1296 | 3358 | 159 % | n.d. | n.d. | n.d. | ✓ | ✓ | ✓ | 82 | 10 |
| **Z7** | 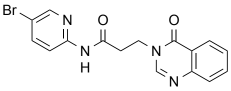 | 302.6 | n.d. | n.d. | n.d. | 0 % | n.d. | n.d. | 873 | | | ✓ | 84 | This work |
| **Z11** | 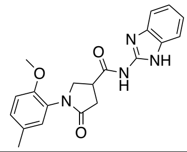 | 177.9 | 436 | 1066 | n.d. | 838 % | 3218 | n.d. | n.d. | | | ✓ | 83 | This work |
| **Z12** | 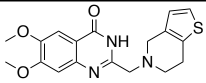 | 833 | 3492 | 1366 | 2036 | 49 % | 1944 | n.d. | 2233 | | | ✓ | 86 | This work |
| **NCT02** | 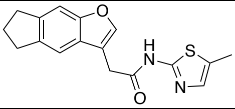 | n/t | n/t | n/t | n/t | n/t | n/t | n/t | n/t | | | | 87 | 11 |
| **SNS032** | 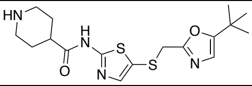 | n.d. | n.d. | 265 | 207 | -22 % | | | | | | | 90 | 12 |
| **Dinaciclib** | 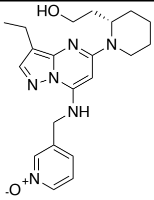 | n.d. | n.d. | 17 | 15 | -12 % | | | | | ✓ | | 56 | 13 |
| **Danusertib** | 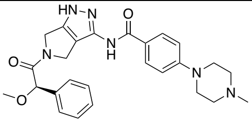 | n.d. | n.d. | n.d. | n.d. | 0 % | | | | | | | 58 | 14 |
| **NVP-2** | 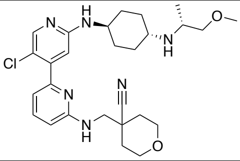 | n.d. | n.d. | 23 | 18 | -22 % | | | | | | | 59 | 15 |
| **THZ531** | 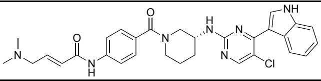 | n.d. | n.d. | 271 | 212 | -22 % | | | | | ✓ | | 54 | 16 |
| **GSK2250665 A** | 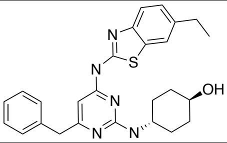 | n.d. | n.d. | ? | ? | ? | | | | | | | 57 | 17 |

| | | | | | | | | | | | | | | |
|---|---|---|---|---|---|---|---|---|---|---|---|---|---|---|
| **BSJ-4-116** | 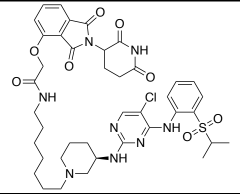 | n.d. | n.d. | 155 | 511 | 230 % | | | | | ✓ | | 91 | 18 |
| **THAL-SNS032** | 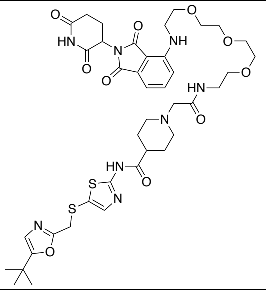 | n.d. | <50 | 192 | 705 | 267 % | | | | | ✓ | | 92 | 15 |

# column represents the order of mention in the manuscript and figures. n.d. refers to measurements were $EC_{50}/DC_{50}/IC_{50}$ value is higher than the highest compound concentration tested (10 µM). For MLN4924-mediated cytotoxicity rescue calculation, the value of 10 µM is used in such cases. Asterisk (*) denotes cases where the reporter degradation curve could not be fitted due to compound autofluorescence. Question mark (?) indicates cases where the fit was ambiguous for other reasons. n/t: not tested.

## References


1. Bettayeb, K. *et al.* CR8, a potent and selective, roscovitine-derived inhibitor of cyclin-dependent kinases. *Oncogene* **27**, 5797–5807 (2008).
2. Thede, K. *et al.* PYRAZOLOTRIAZINES. Patent WO2021116178. (2021).
3. Meijer, L. *et al.* Biochemical and Cellular Effects of Roscovitine, a Potent and Selective Inhibitor of the Cyclin-Dependent Kinases cdc2, cdk2 and cdk5. *Eur J Biochem* **243**, 527–536 (1997).
4. Oumata, N. *et al.* Roscovitine-derived, dual-specificity inhibitors of cyclin-dependent kinases and casein kinases 1. *J Med Chem* **51**, 5229–5242 (2008).
5. Sedlacek, H. H. *et al.* Flavopiridol (L86 8275; NSC 649890), a new kinase inhibitor for tumor therapy. *Int J Oncol* **9**, 1143–1168 (1996).
6. Caligiuri, M. *et al.* A proteome-wide CDK/CRK-specific kinase inhibitor promotes tumor cell death in the absence of cell cycle progression. *Chem Biol* **12**, 1103–1115 (2005).
7. Quereda, V. *et al.* Therapeutic Targeting of CDK12/CDK13 in Triple-Negative Breast Cancer. *Cancer Cell* **36**, 545-558.e7 (2019).
8. Henry, K. L. *et al. CDK12-mediated transcriptional regulation of noncanonical NF-kB components is essential for signaling.* http://stke.sciencemag.org/ (2018).
9. Mayor-Ruiz, C. *et al.* Rational discovery of molecular glue degraders via scalable chemical profiling. *Nat Chem Biol* (2020) doi:10.1038/s41589-020-0594-x.
10. Lv, L. *et al.* Discovery of a molecular glue promoting CDK12-DDB1 interaction to trigger Cyclin K degradation. *Elife* **9**, (2020).
11. Dieter, S. M. *et al.* Degradation of CCNK/CDK12 is a druggable vulnerability of colorectal cancer. *Cell Rep* **36**, 109394 (2021).
12. SNS-032 is a potent and selective inhibitor of CDK2, 7 and 9 and induces cell death by inhibiting cell cycle progression and the expression of antiapoptotic proteins | Cancer Research | American Association for Cancer Research. https://aacrjournals.org/cancerres/article/66/8_Supplement/491/528216/SNS-032-is-a-potent-and-selective-inhibitor-of.
13. Parry, D. *et al.* Dinaciclib (SCH 727965), a novel and potent cyclin-dependent kinase inhibitor. *Mol Cancer Ther* **9**, 2344–2353 (2010).
14. Carpinelli, P. *et al.* PHA-739358, a potent inhibitor of Aurora kinases with a selective target inhibition profile relevant to cancer. *Mol Cancer Ther* **6**, 3158–3168 (2007).
15. Olson, C. M. *et al.* Pharmacological perturbation of CDK9 using selective CDK9 inhibition or degradation. *Nat Chem Biol* **14**, 163–170 (2018).
16. Zhang, T. *et al.* Covalent targeting of remote cysteine residues to develop CDK12 and CDK13 inhibitors. *Nat Chem Biol* **12**, 876–884 (2016).
17. Alder, C. M. *et al.* Identification of a Novel and Selective Series of Itk Inhibitors via a Template-Hopping Strategy. *ACS Med Chem Lett* **4**, 948–952 (2013).
18. Jiang, B. *et al.* Discovery and resistance mechanism of a selective CDK12 degrader. *Nature Chemical Biology 2021 17:6* **17**, 675–683 (2021).

**Supplementary Table 3.** Compounds purchased from commercial vendors.

| Compound | Company | Catalogue number |
|---|---|---|
| *R*-CR8 | Tocris | 3605 |
| GSK2250665A | Tocris | 5401 |
| Flavopiridol | Enzo Life Sciences | ALX-430-161-M005 |
| Roscovitine | MedChemExpress | HY-30237 |
| THZ531 | MedChemExpress | HY-103618 |
| LDC00067 | MedChemExpress | HY-15878 |
| SR-4835 | MedChemExpress | HY-130250 |
| Danusertib | MedChemExpress | HY-10179 |
| Dinaciclib | MedChemExpress | HY-10492 |
| BSJ-4-116 | MedChemExpress | HY-139039 |
| 21195 | Cayman Chemical Company | 21195 |
| 919278 | ProbeChem | PC-35532 |
| DRF053 | Sigma | D6946 |
| dCeMM2 | LabNetwork | STK119616 |
| dCeMM3 | Enamine | Z54609541 |
| dCeMM4 | Enamine | Z1126858802 |
| Z7 | Enamine | Z200170434 |
| Z11 | Enamine | Z27665843 |
| Z12 | Enamine | Z225160068 |
| SNS032 | MedChemExpress | HY-10008 |
| NVP-2 | MedChemExpress | HY-12214A |
| *S*-CR8 | Enzo Life Sciences | ALX-270-509-M005 |

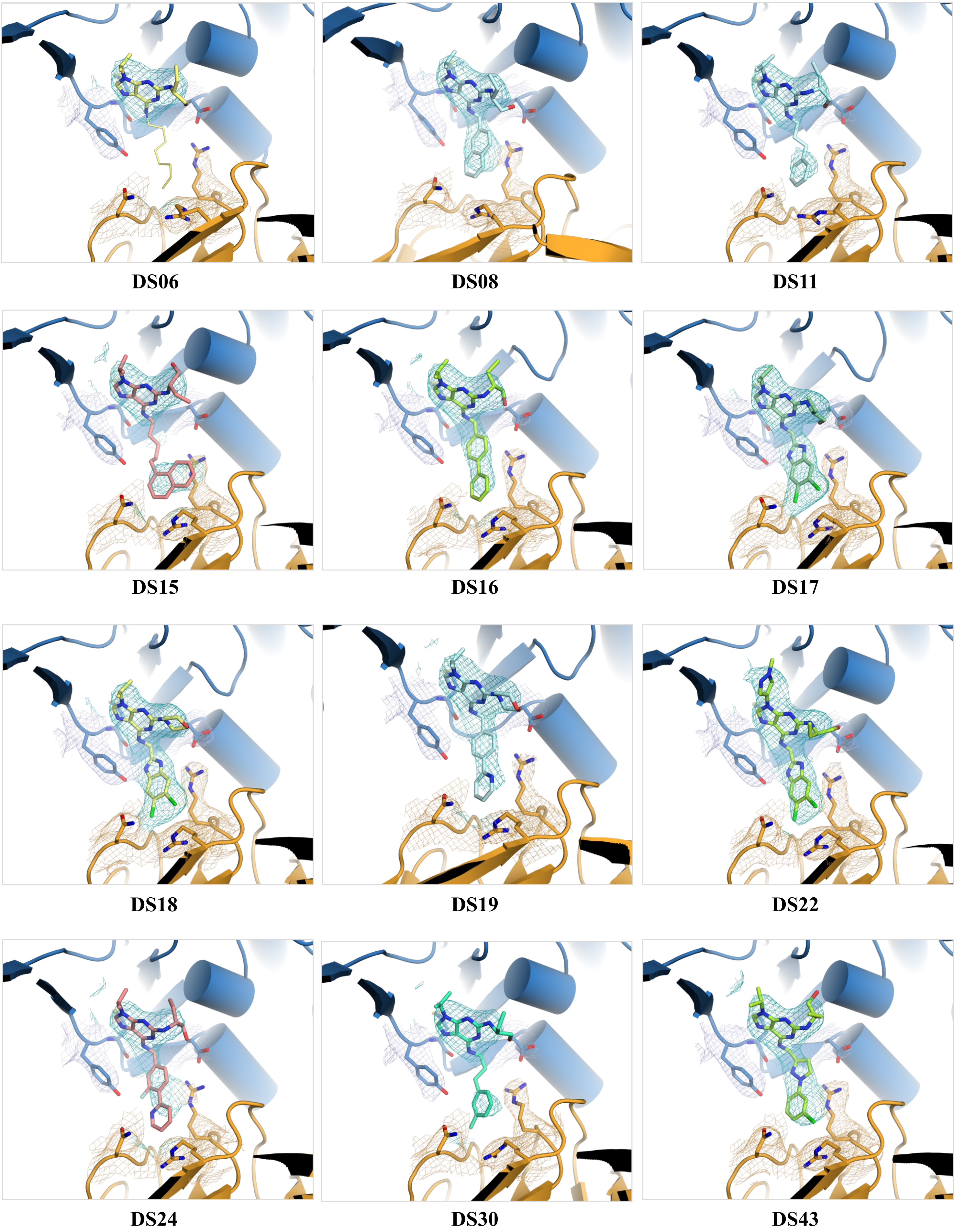


**Supplementary Figure 1.** 2Fo-Fc density maps (at the contour level of 1σ) for all ternary complex structures. Density for the small molecule, DDB1 residues R907, R928, R947, and CDK12 residues M816 and Y815 is shown.

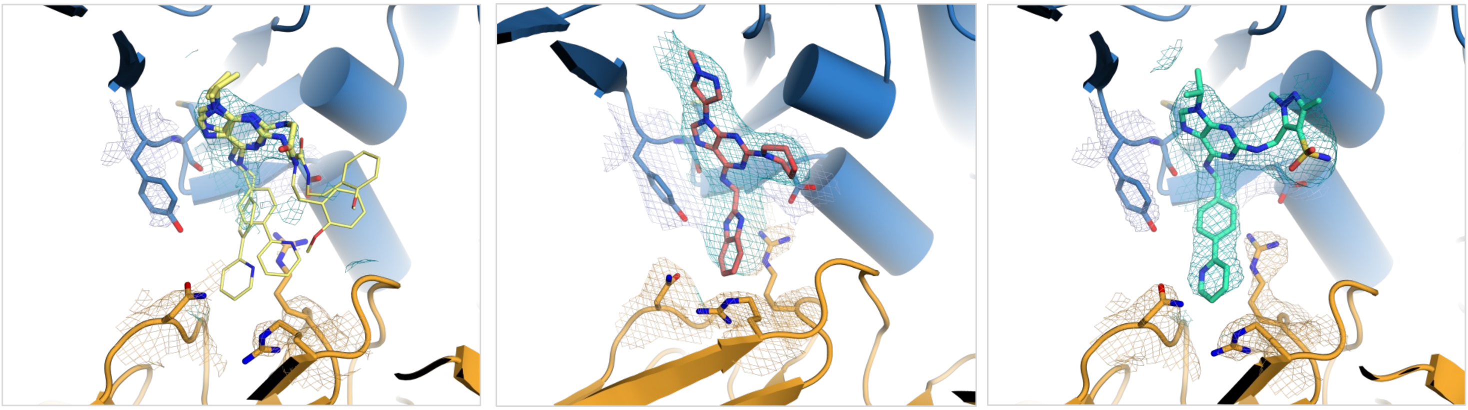

**DS50** **DS55** **DS59**

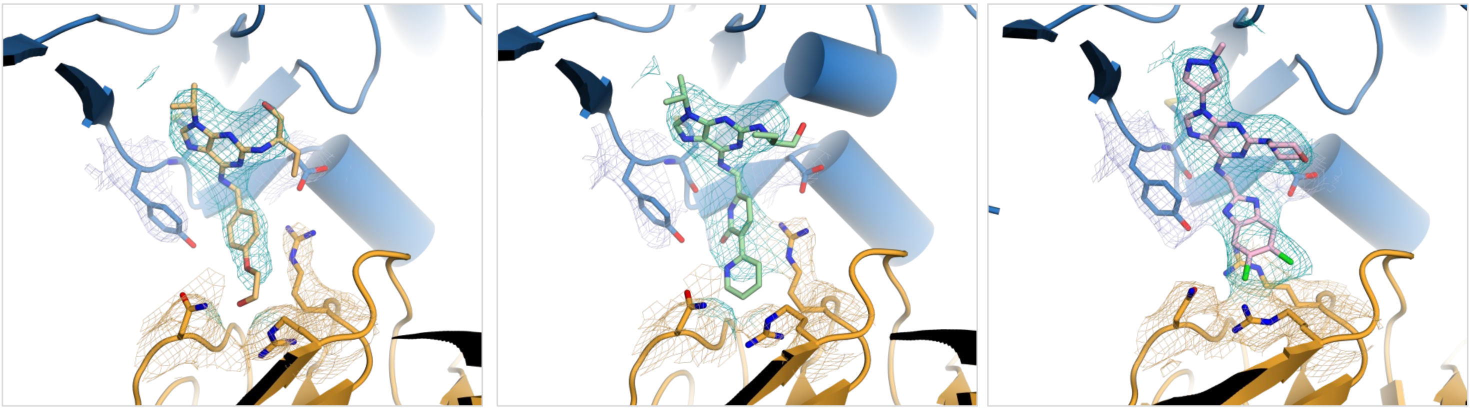

**DS61** **WX3** **SR-4835**

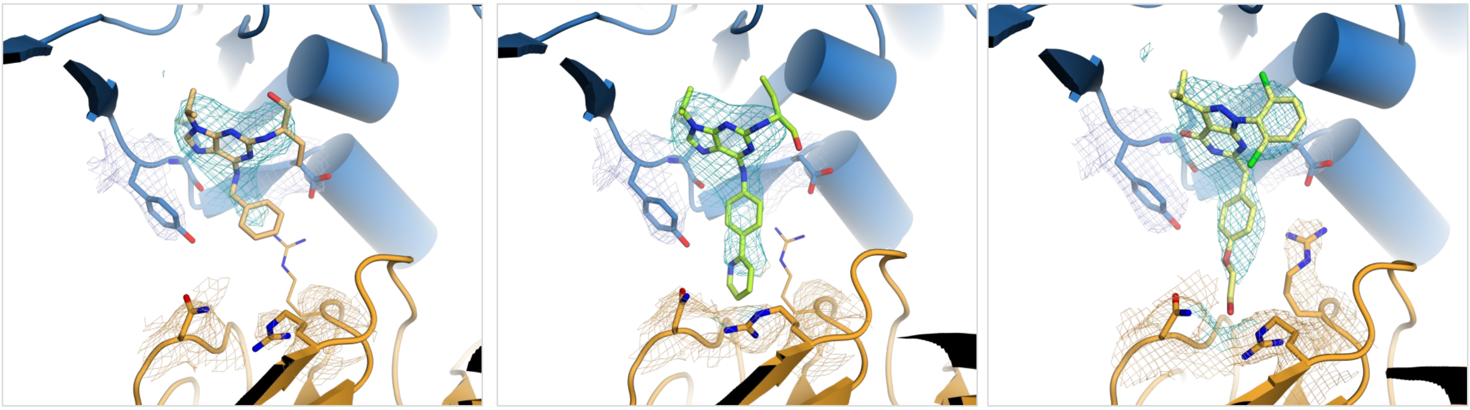

**Roscovitine** **DRF053** **21195**

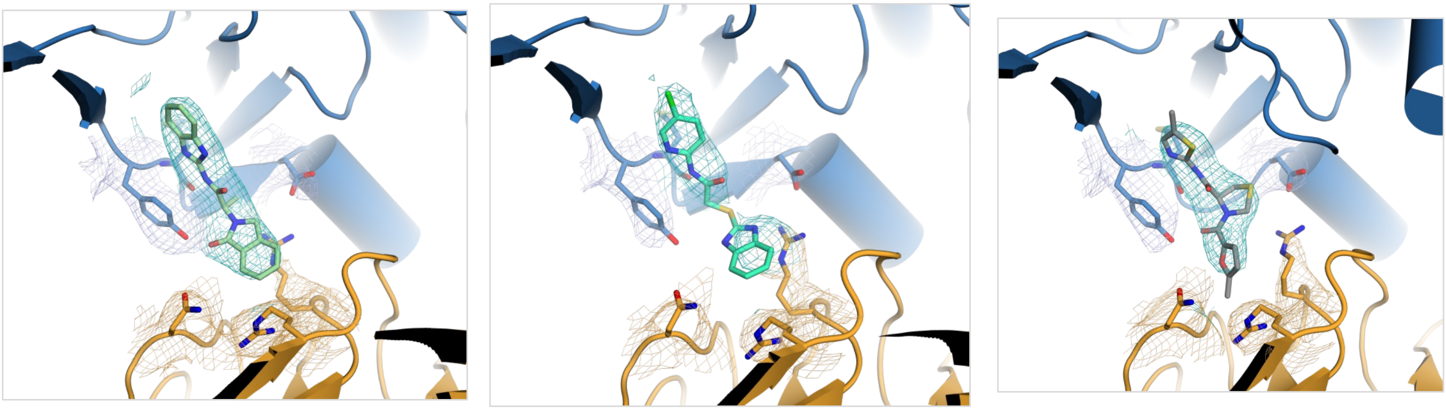

**919278** **dCeMM3** **dCeMM4**

**Supplementary Figure 1 (cont.).** Density maps (at the contour level of 1σ) for all ternary complex structures. Density for the small molecule, DDB1 residues R907, R928, R947, and CDK12 residues M816 and Y815 is shown.

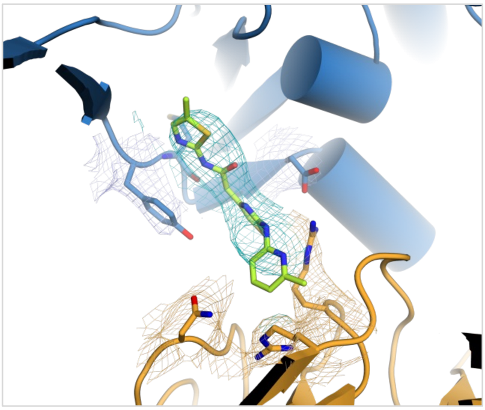

**HQ461**

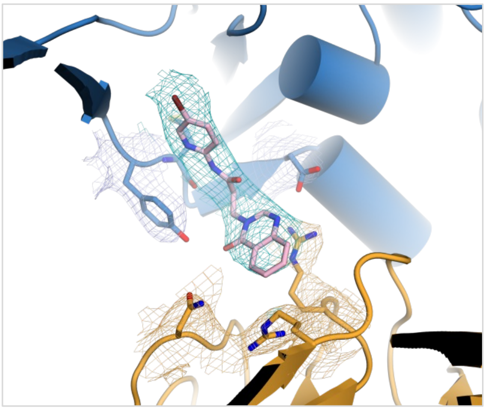

**Z7**

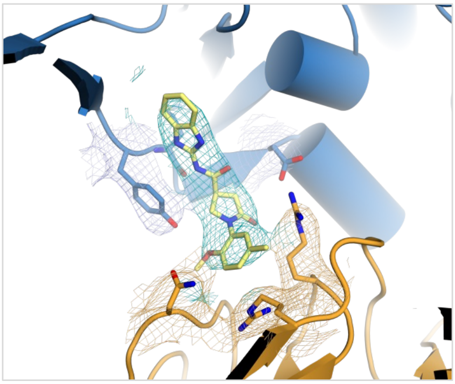

**Z11**

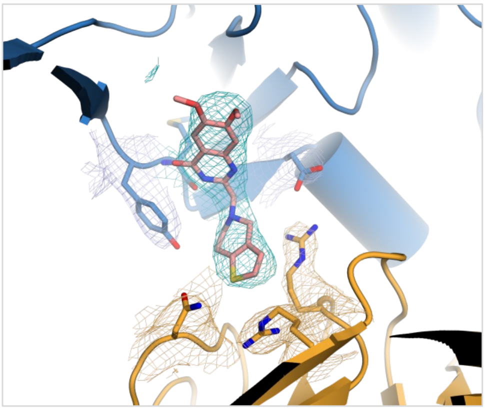

**Z12**

**Supplementary Figure 1 (cont.).** Density maps (at the contour level of 1σ) for all ternary complex structures. Density for the small molecule, DDB1 residues R907, R928, R947, and CDK12 residues M816 and Y815 is shown.

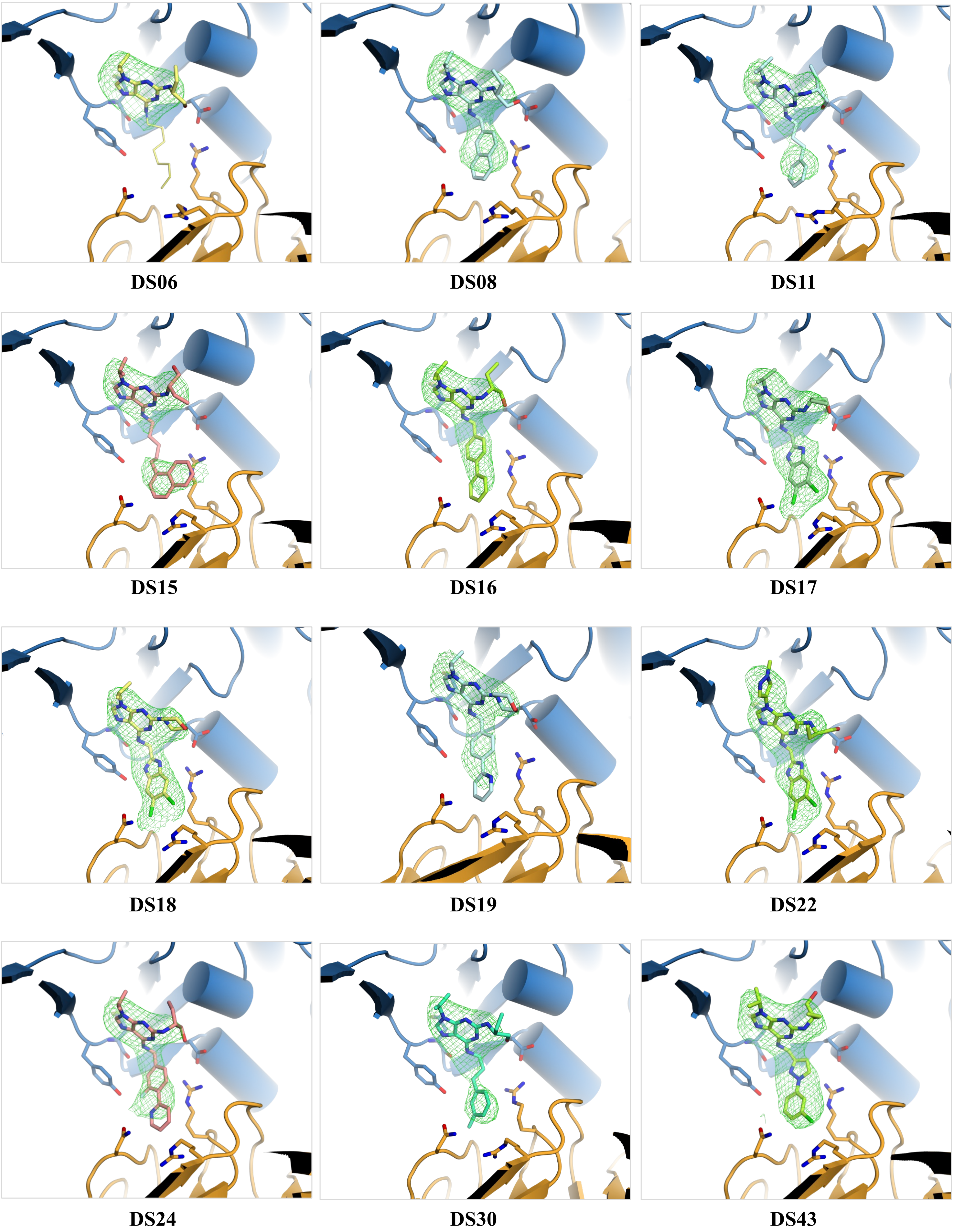


**Supplementary Figure 2.** Omit Fo-Fc difference density maps for the small molecule at the default contour level of 3σ. The omit maps were generated through simulated annealing to remove model bias.

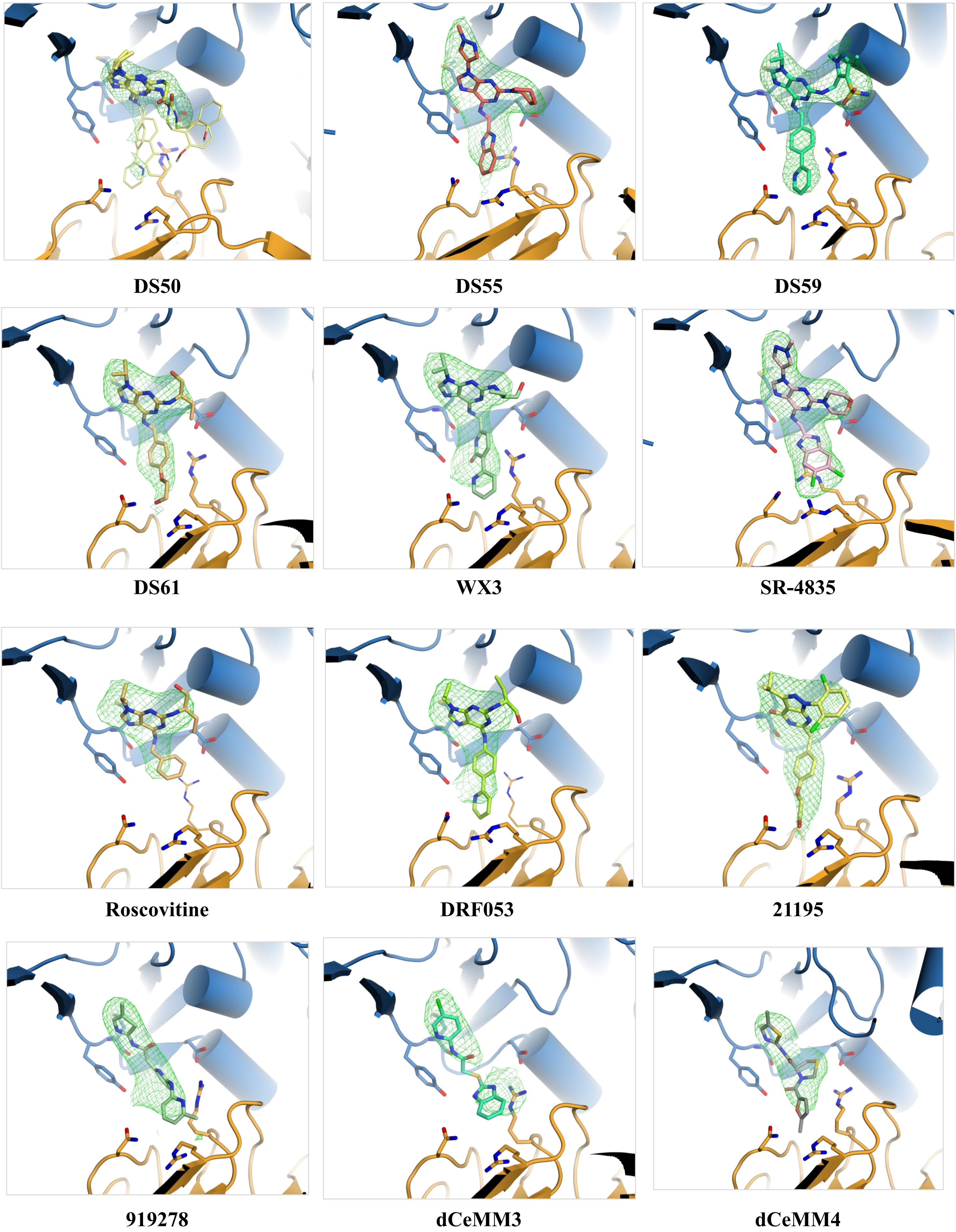


**Supplementary Figure 2 (cont.).** Omit Fo-Fc difference density maps for the small molecule at the default contour level of 3σ. The omit maps were generated using simulated annealing to remove model bias.

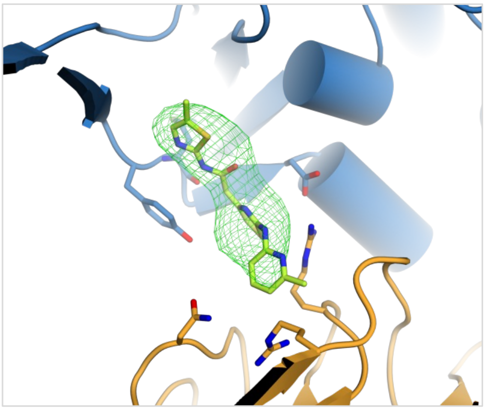

**HQ461**

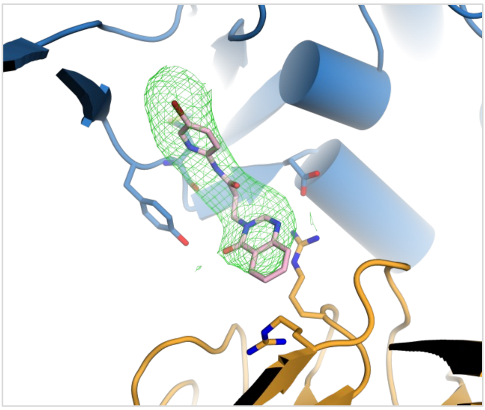

**Z7**

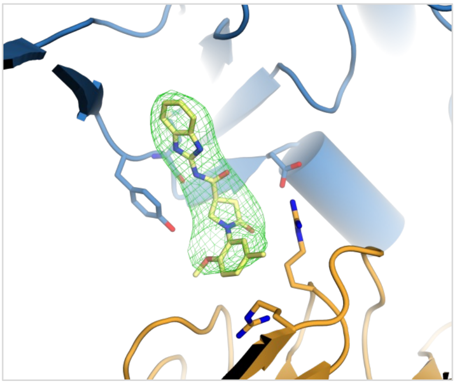

**Z11**

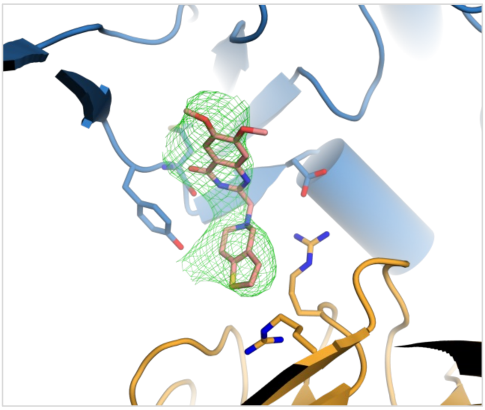

**Z12**

**Supplementary Figure 2 (cont.).** Omit Fo-Fc difference density maps for the small molecule at the default contour level of 3σ. The omit maps were generated through simulated annealing to remove model bias.

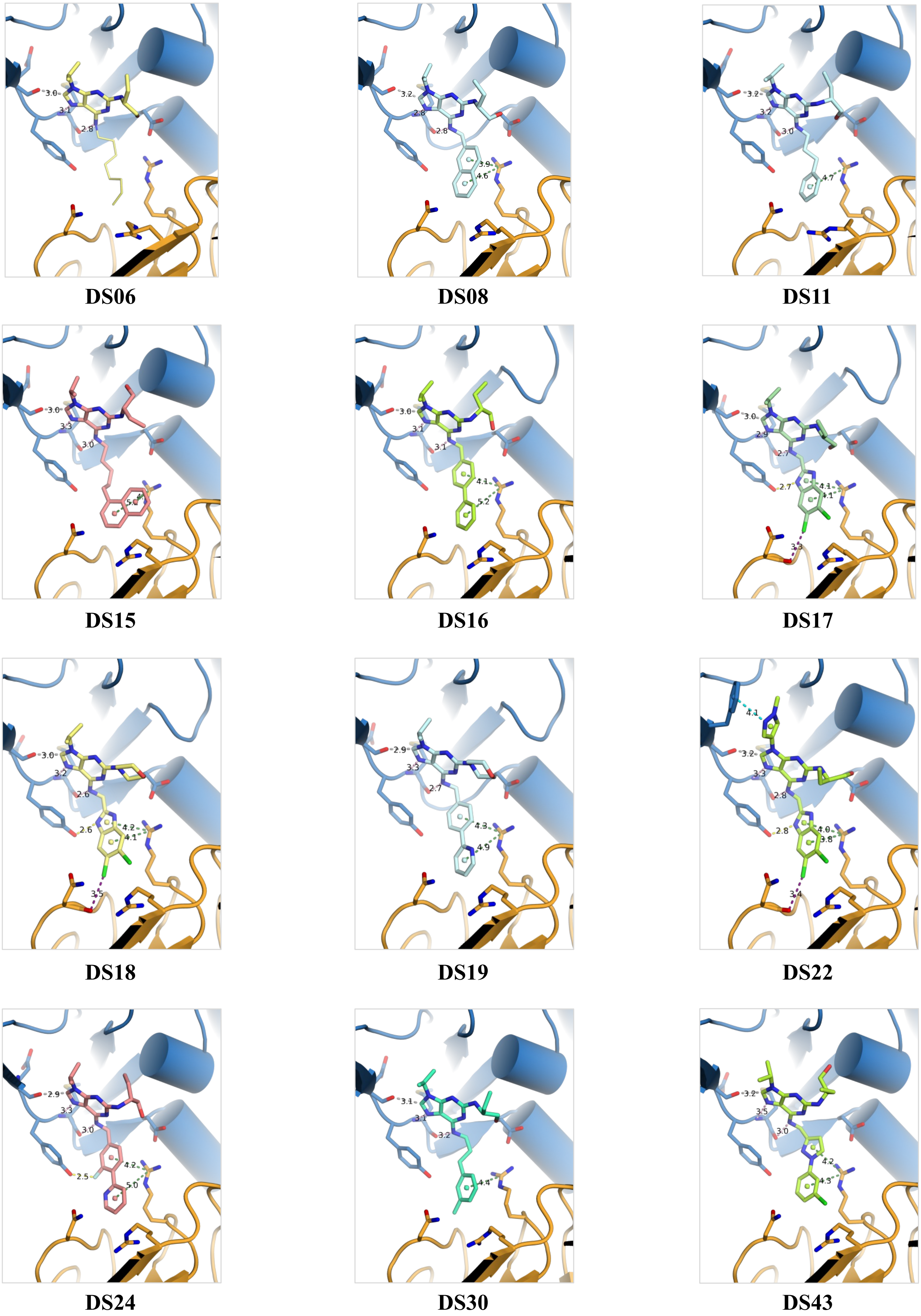


**Supplementary Figure 3.** Key protein-ligand interactions for all ternary complex structures. Distances to other proximal residues are also shown where appropriate. Spheres indicate the centre of mass of each aromatic ring in the gluing moiety. These centre points were used for the relevant measurements.

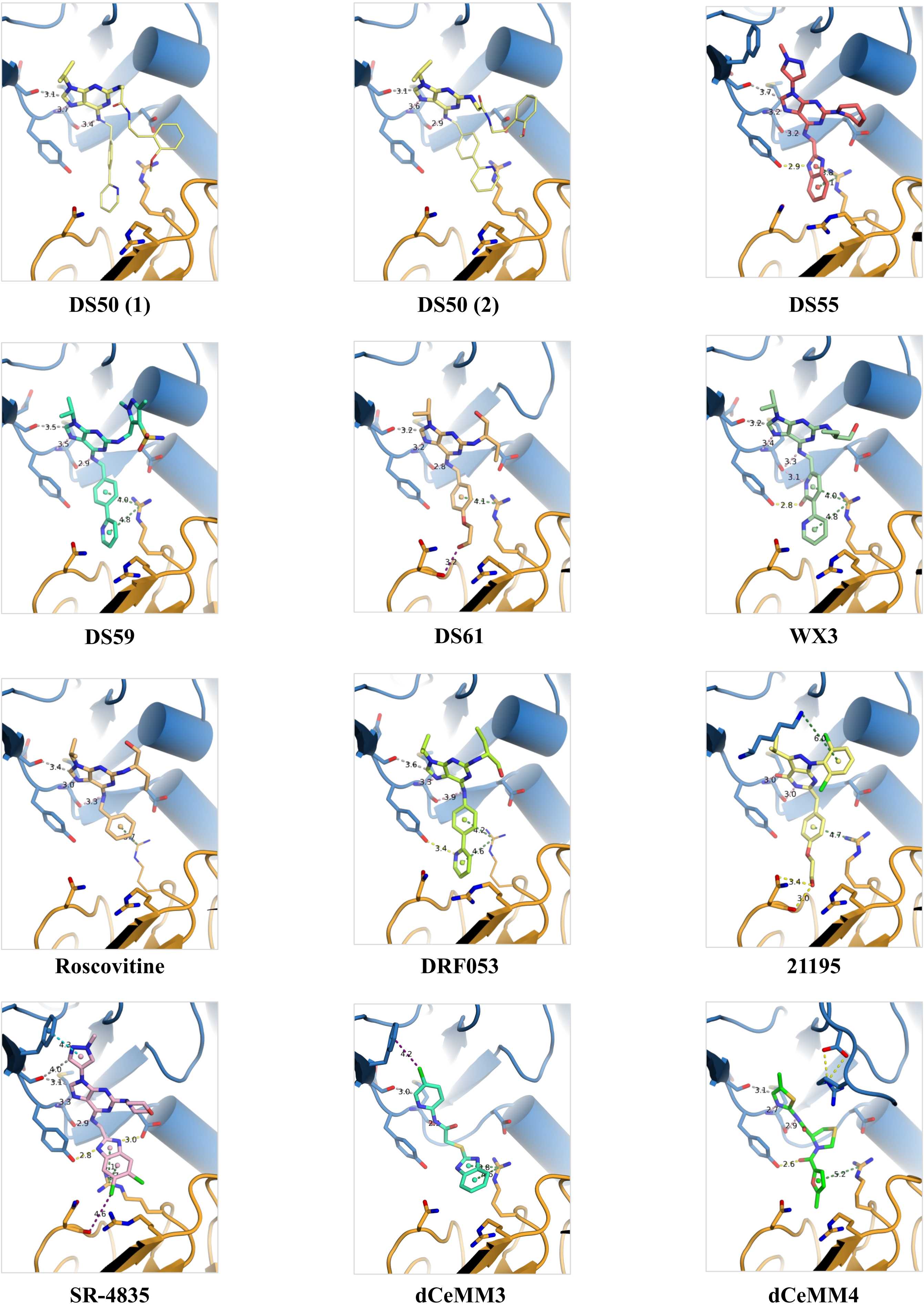


**Supplementary Figure 3 (cont.).** Key protein-ligand interactions for all ternary complex structures. Distances to other proximal residues are also shown where appropriate. Spheres indicate the centre of mass of each aromatic ring in the gluing moiety. These centre points were used for the relevant measurements.

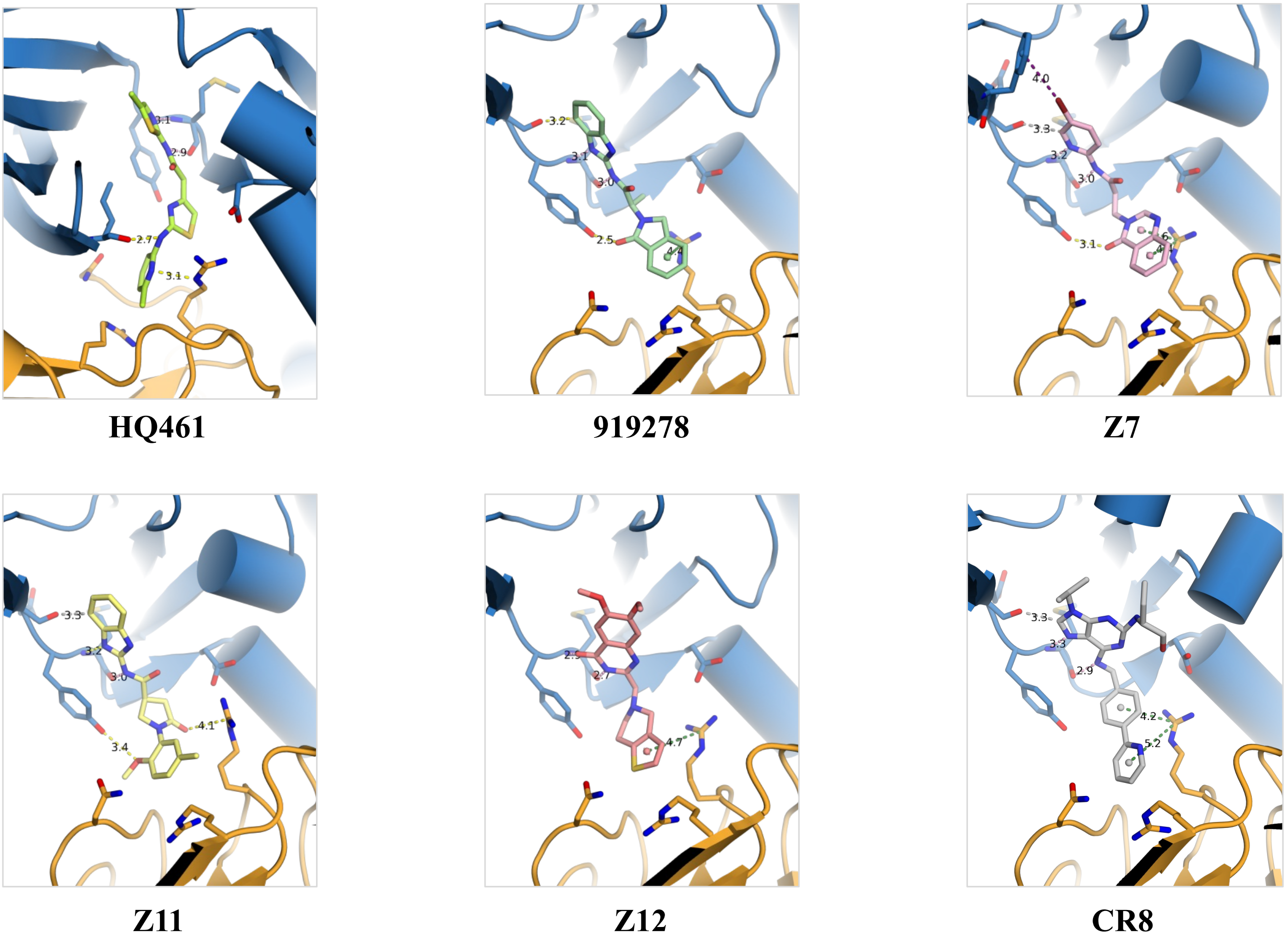


**Supplementary Figure 3 (cont.).** Key protein-ligand interactions for all ternary complex structures. Distances to other proximal residues are also shown where appropriate. Spheres indicate the centre of mass of each aromatic ring in the gluing moiety. These centre points were used for the relevant measurements.

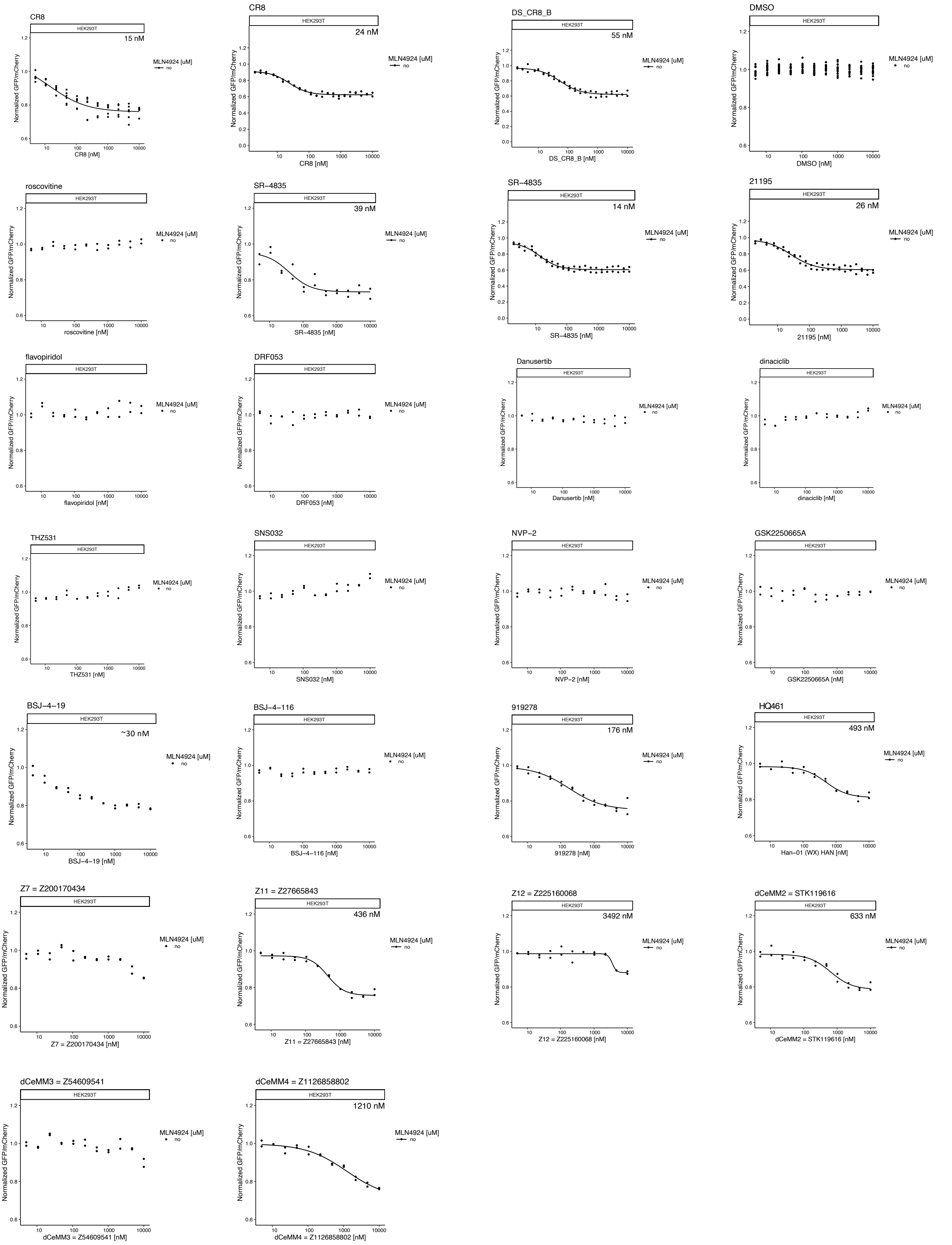


**Supplementary Figure 4.** HEK293 cycK$_{eGFP}$ reporter assay results. Cells were treated with the indicated compound for 5h. Individual replicates are shown (n=2).

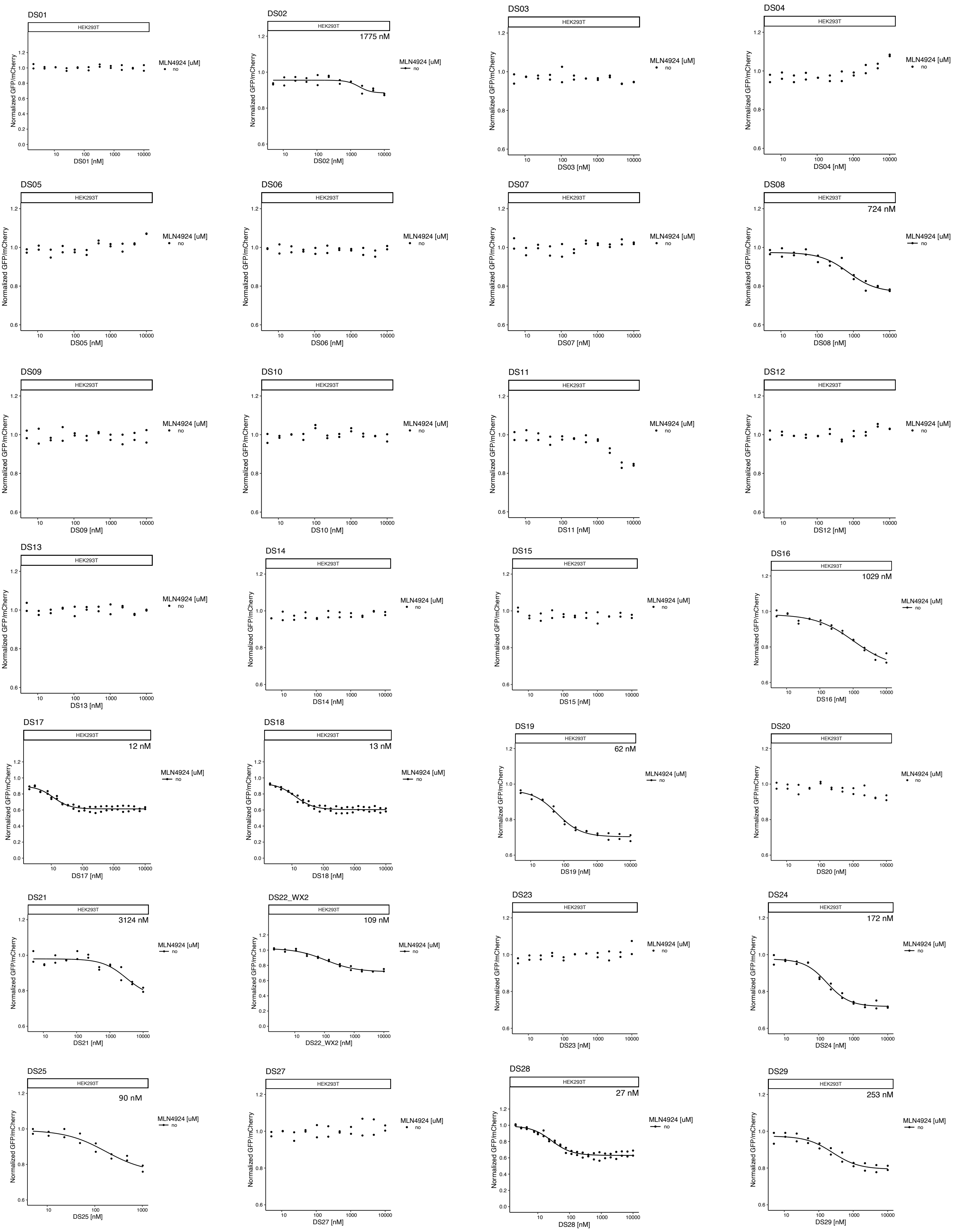


**Supplementary Figure 4 (cont.).** HEK293 cycK$_{eGFP}$ reporter assay results. Cells were treated with the indicated compound for 5h. Individual replicates are shown (n=2).

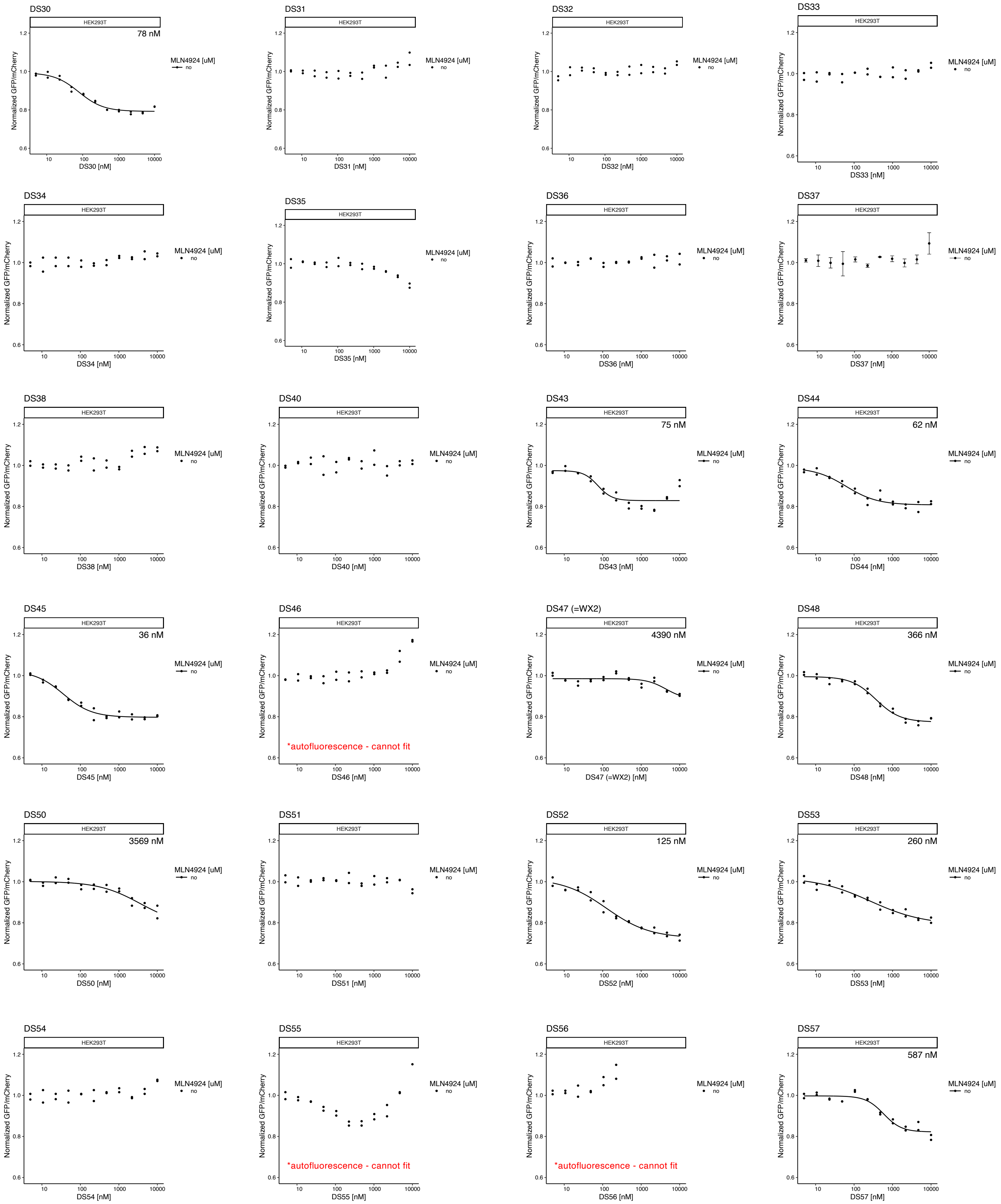


**Supplementary Figure 4 (cont.).** HEK293 cycK$_{eGFP}$ reporter assay results. Cells were treated with the indicated compound for 5h. Individual replicates are shown (n=2).

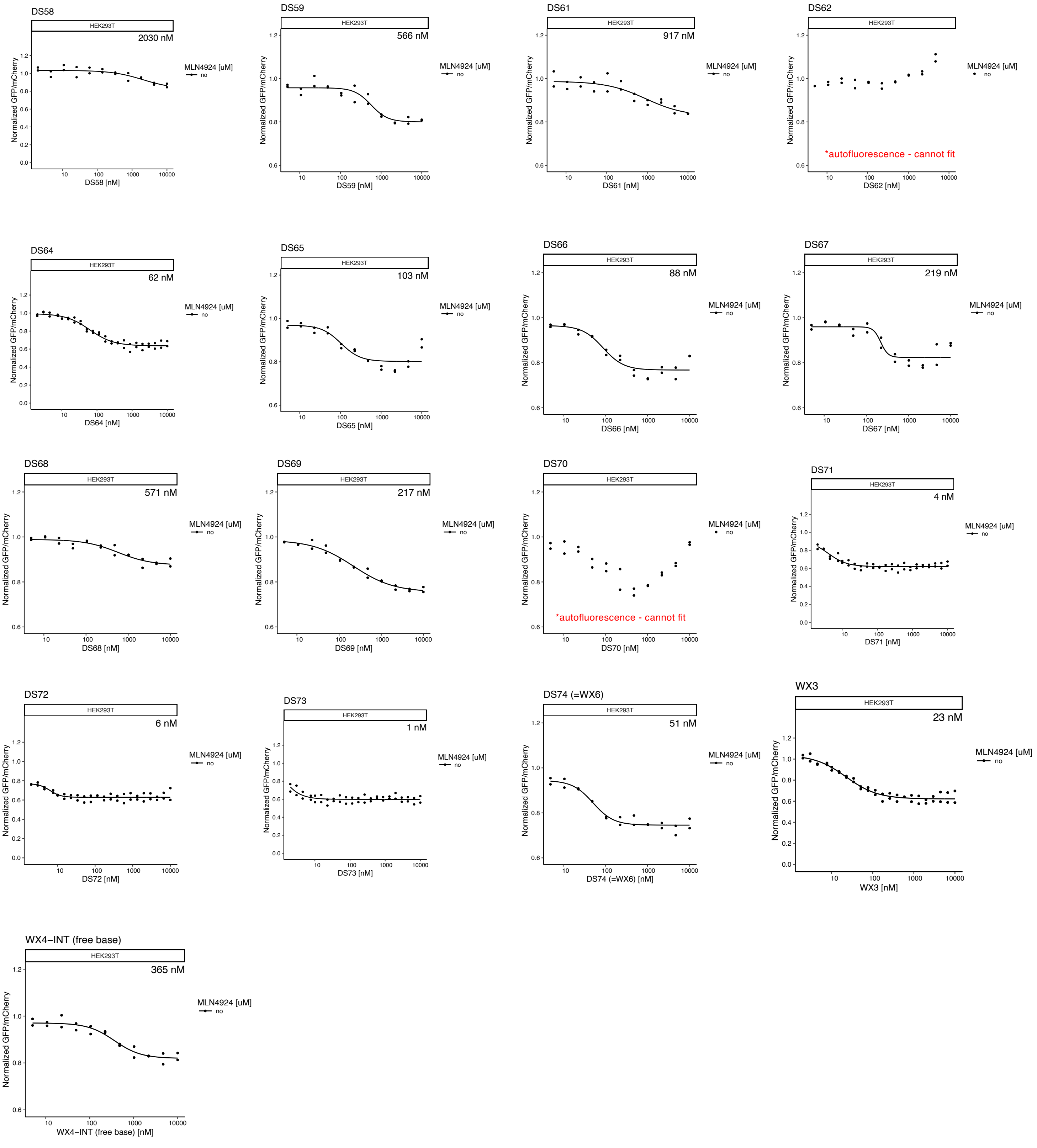


**Supplementary Figure 4 (cont.).** HEK293 cycK$_{eGFP}$ reporter assay results. Cells were treated with the indicated compound for 5h. Individual replicates are shown (n=2).

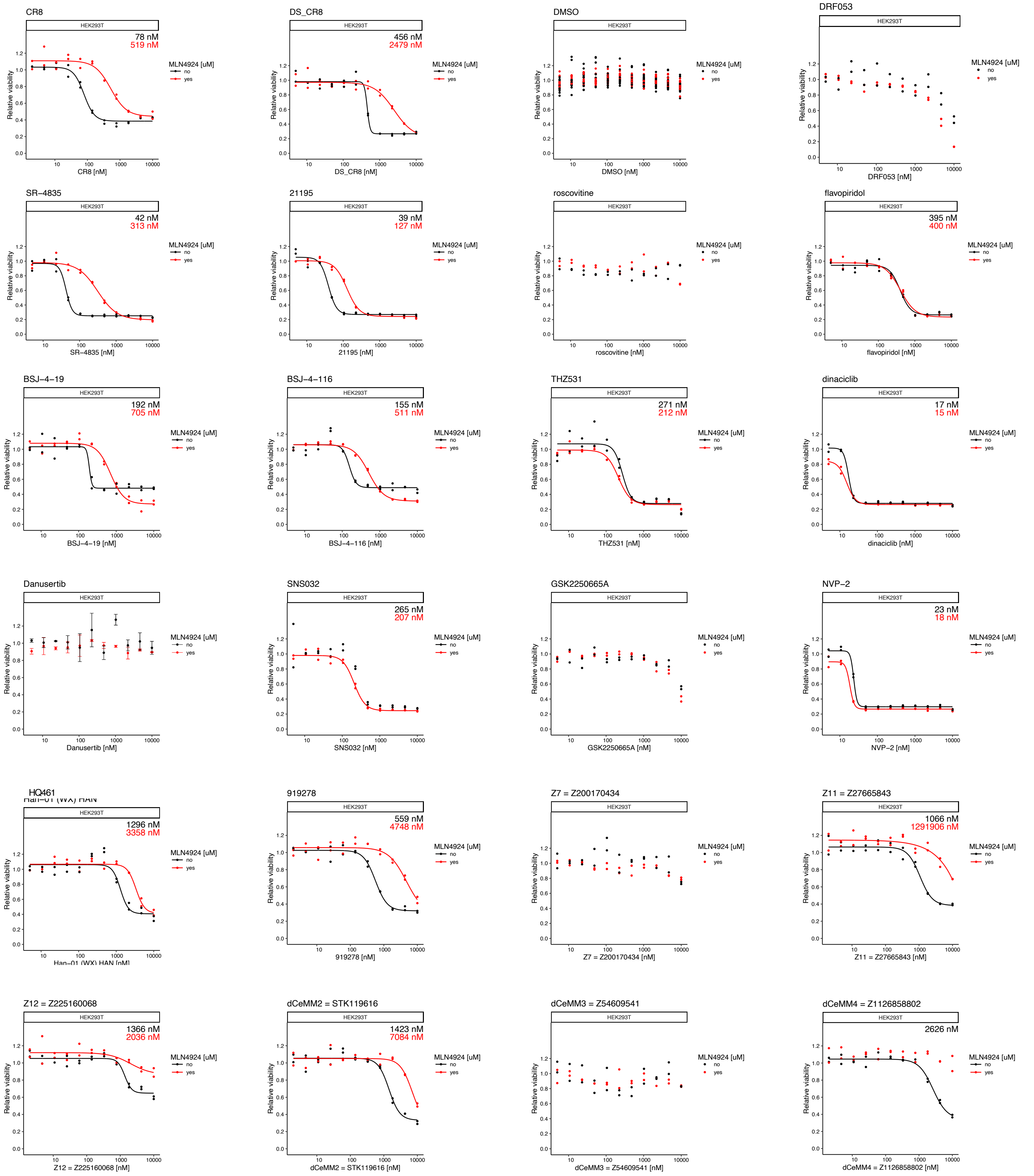


**Supplementary Figure 5.** CellTiter Glo assay evaluating HEK293T cell viability 72h after compound treatment. Red curve corresponds to additional pre-treatment of cells with 100 nM of the neddylation inhibitor MLN4924. Individual replicates are shown (n=2).

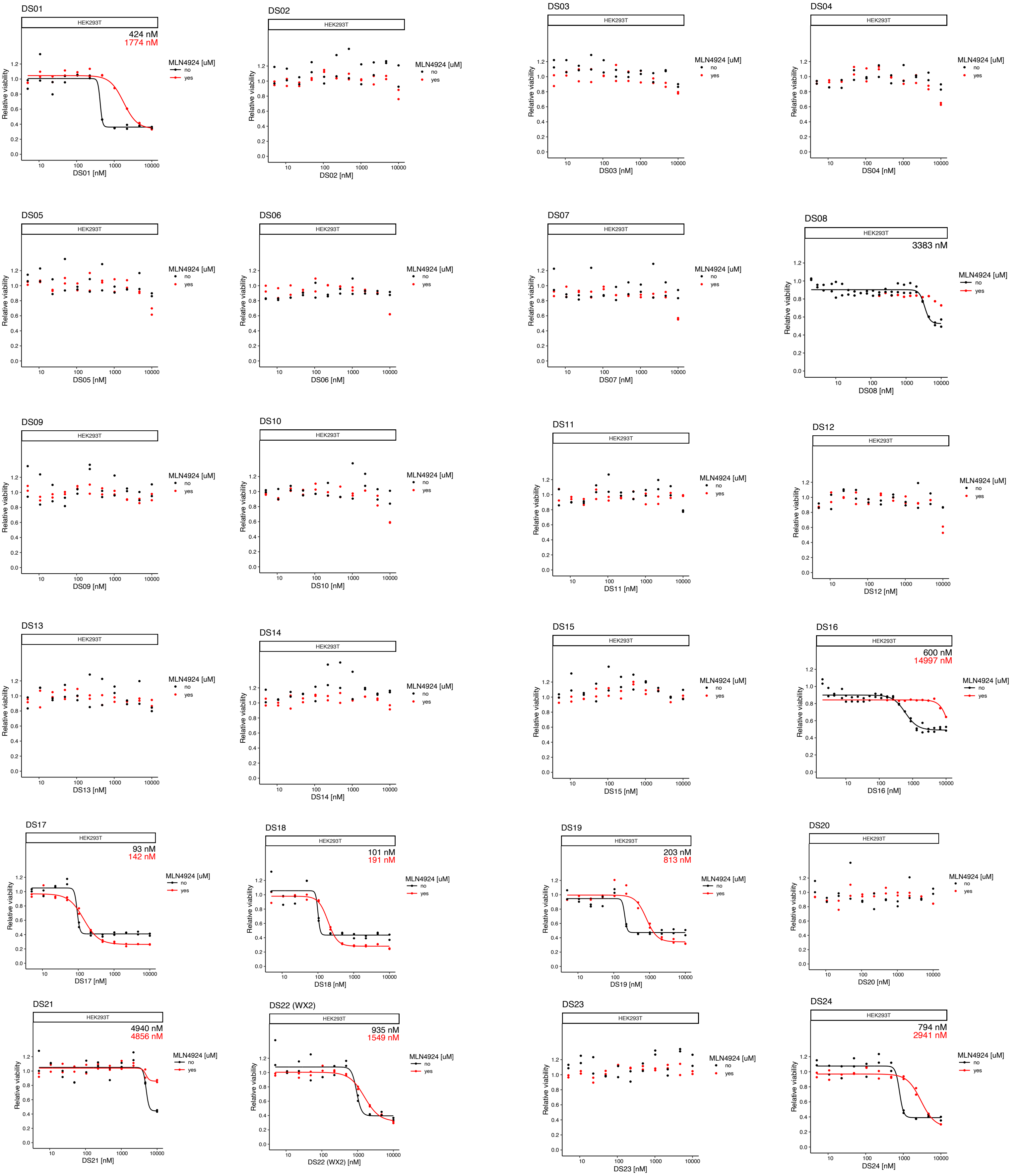


**Supplementary Figure 5 (cont.).** CellTiter Glo assay evaluating HEK293T cell viability 72h after compound treatment. Red curve corresponds to additional pre-treatment of cells with 100 nM of the neddylation inhibitor MLN4924. Individual replicates are shown (n=2).

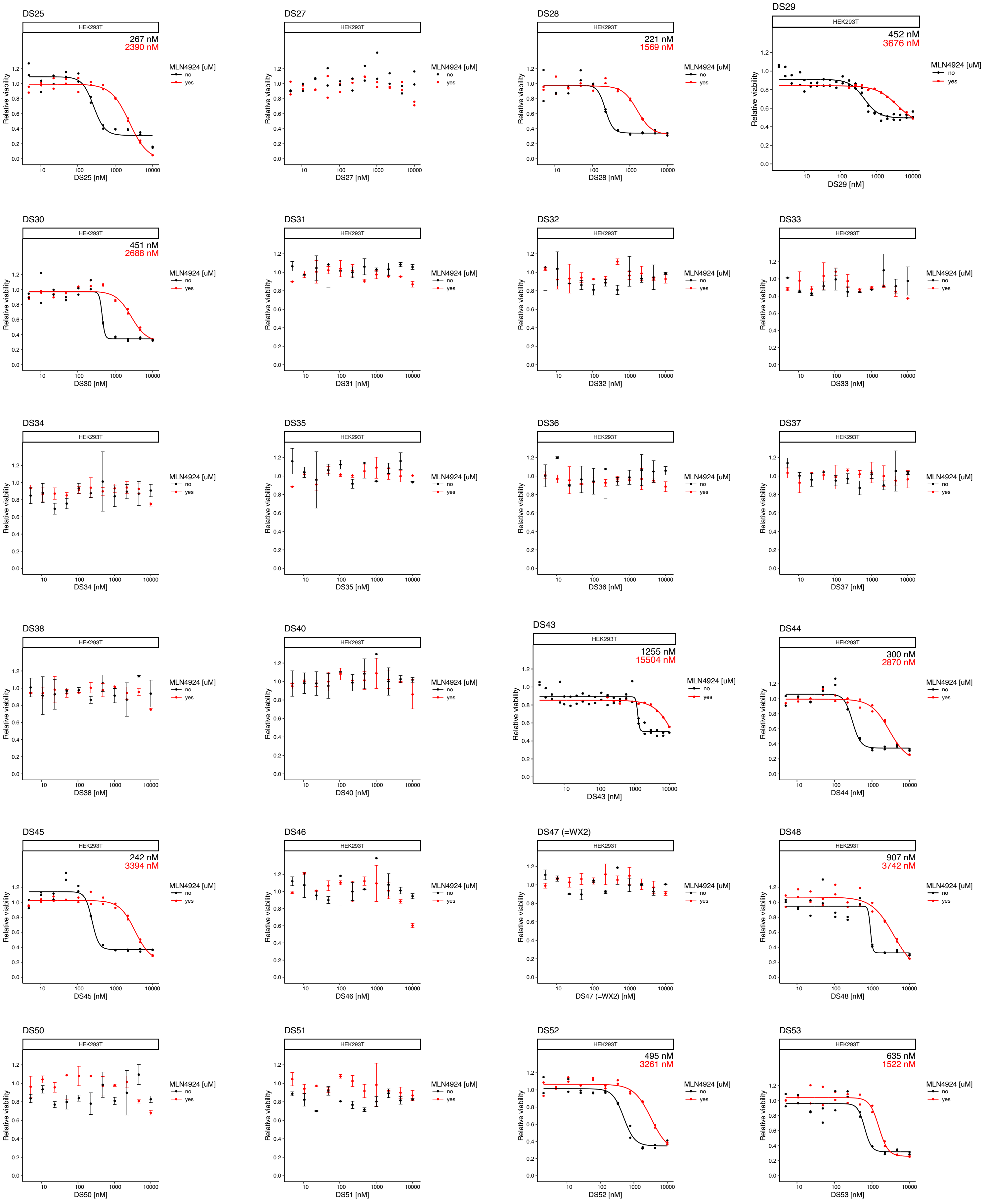


**Supplementary Figure 5 (cont.).** CellTiter Glo assay evaluating HEK293T cell viability 72h after compound treatment. Red curve corresponds to additional pre-treatment of cells with 100 nM of the neddylation inhibitor MLN4924. Individual replicates are shown (n=2).

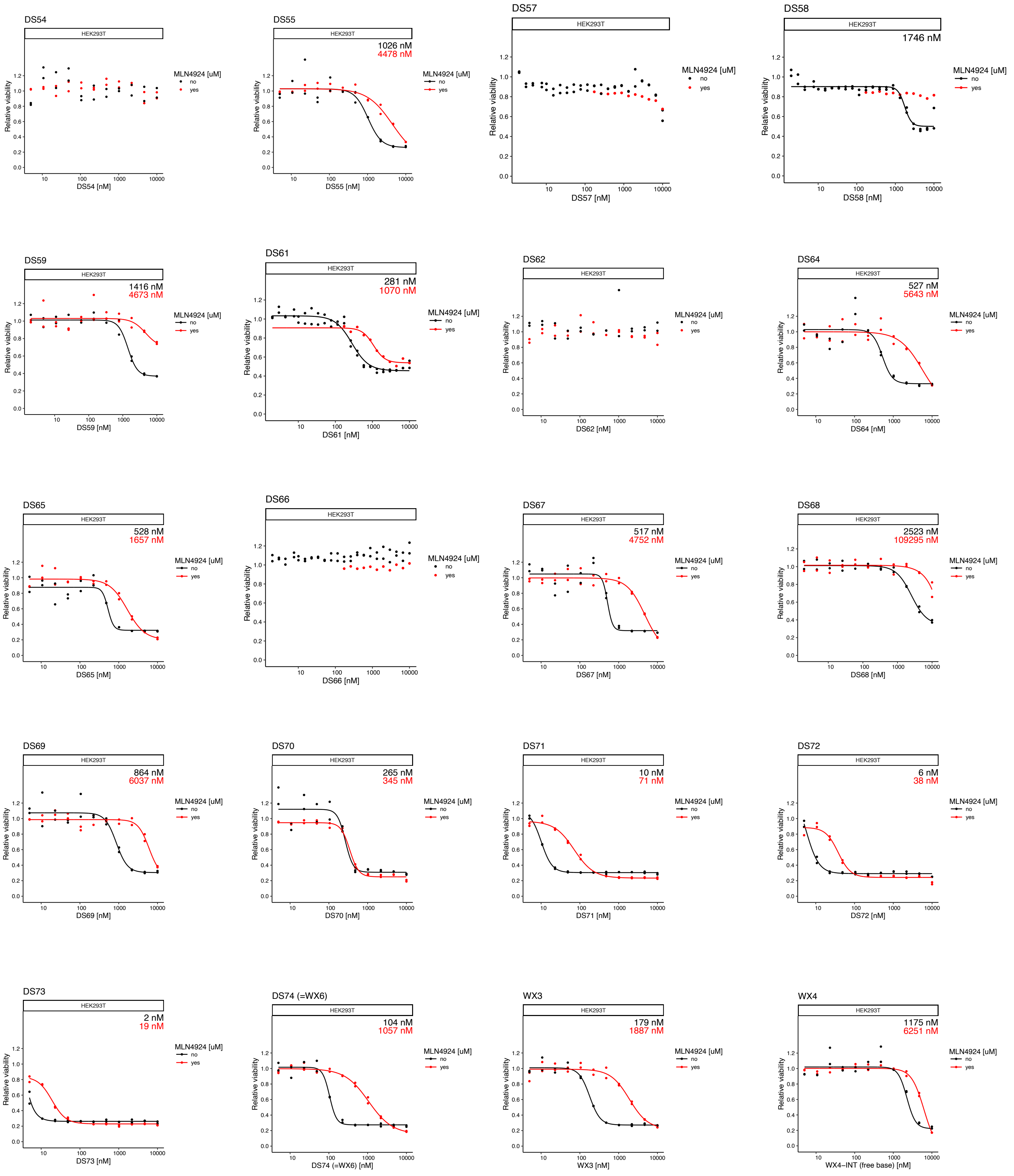


**Supplementary Figure 5 (cont.).** CellTiter Glo assay evaluating HEK293T cell viability 72h after compound treatment. Red curve corresponds to additional pre-treatment of cells with 100 nM of the neddylation inhibitor MLN4924. Individual replicates are shown (n=2).

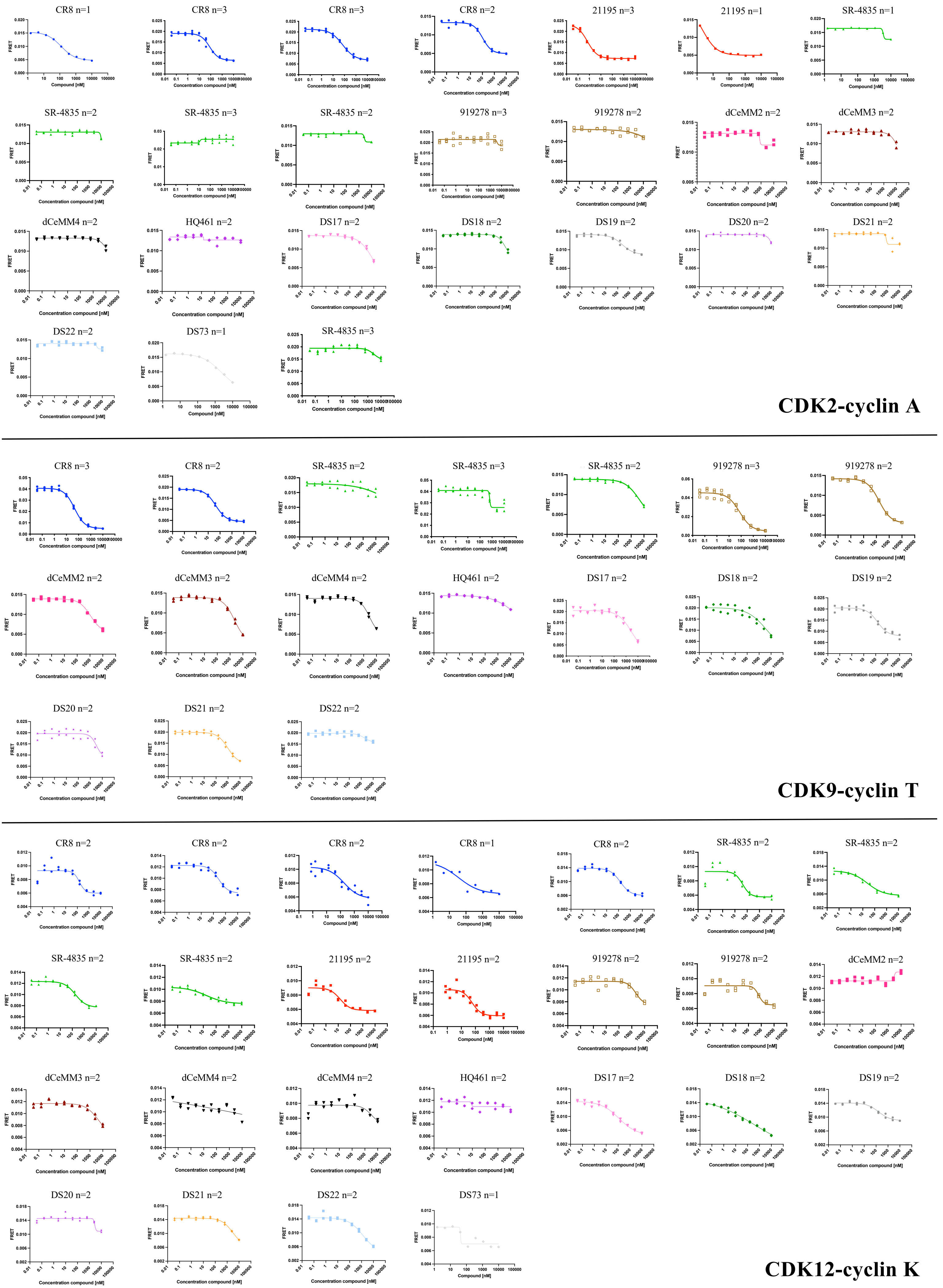


**Supplementary Figure 6.** Lanthascreen data for the indicated compound and kinase (n specified in each panel). Compound dilutions were performed manually.

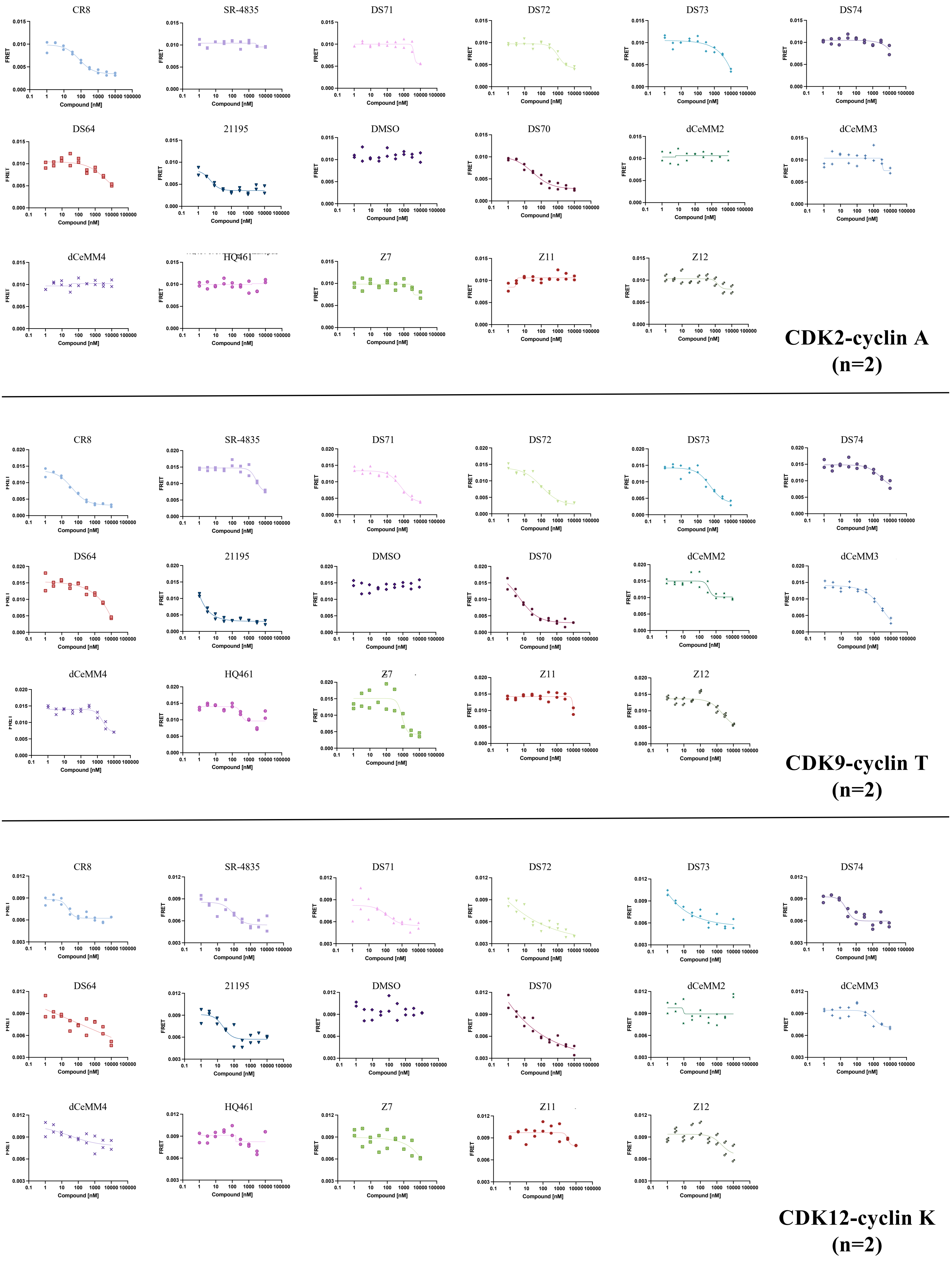


**Supplementary Figure 6 (cont.).** Lanthascreen data for the indicated compound and kinase (n=2). Compound dilutions were performed using a D300 digital dispenser (Tecan).

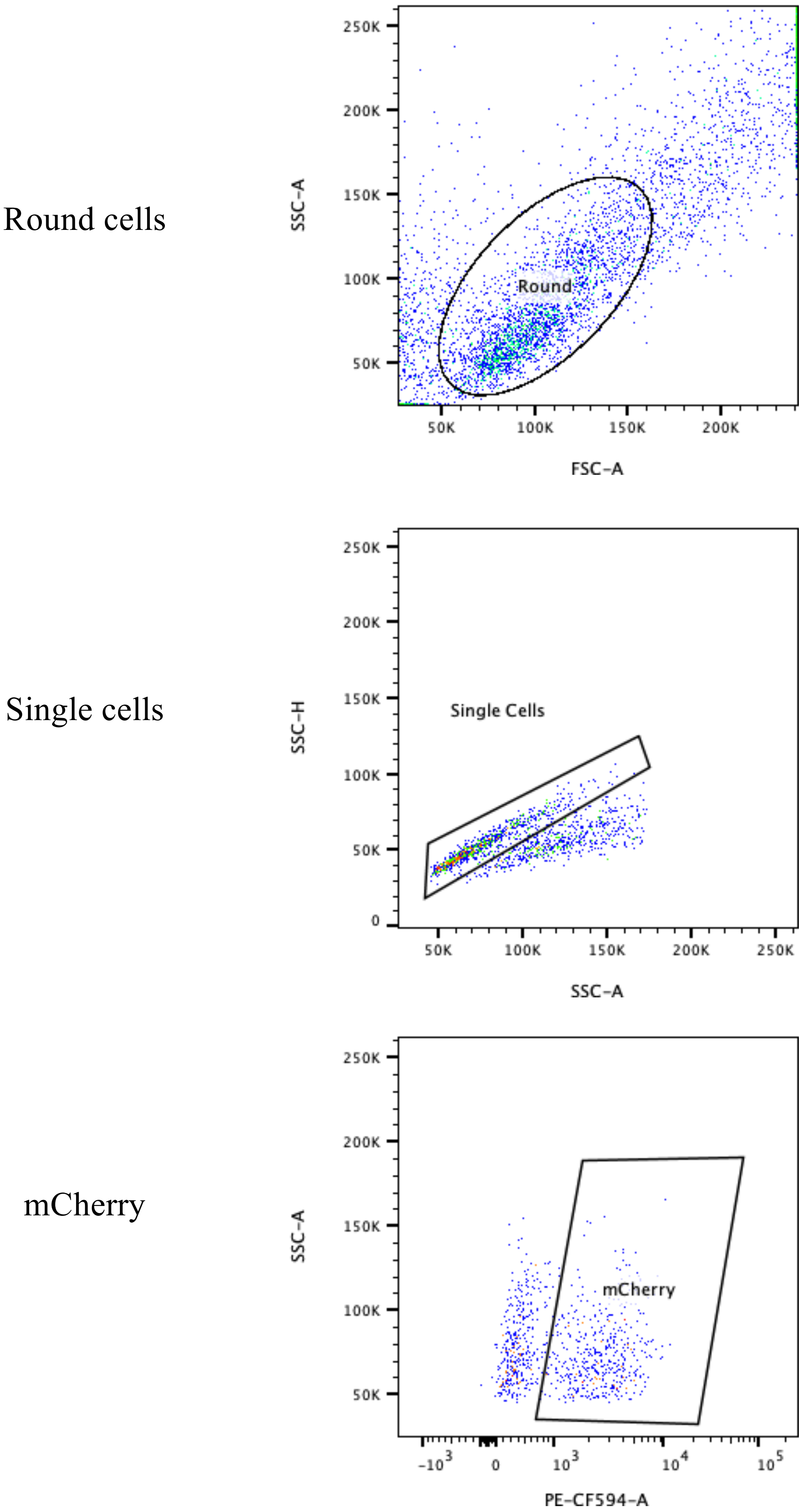


**Supplementary Figure 7.** Flow cytometry gating strategy.
