# Supplementary material for: Design principles for cyclin K molecular glue degraders
Source: Nat Chem Biol. 2023 Sep 7;20(1):93–102. doi: 10.1038/s41589-023-01409-z (PMC10746543; doi:10.1038/s41589-023-01409-z)
Supplement: Supplementary file 2 — Reporting Summary [file 41589_2023_1409_MOESM2_ESM.pdf]

nature research

Corresponding author(s): Nicolas H. Thoma

Last updated by author(s): Jun 14, 2023

Reporting Summary

Nature Research wishes to improve the reproducibility of the work that we publish. This form provides structure for consistency and transparency in reporting. For further information on Nature Research policies, see Authors & Referees and the Editorial Policy Checklist.

Statistics

For all statistical analyses, confirm that the following items are present in the figure legend, table legend, main text, or Methods section.

n/a	Confirmed	
☐	☒	The exact sample size (n) for each experimental group/condition, given as a discrete number and unit of measurement
☒	☐	A statement on whether measurements were taken from distinct samples or whether the same sample was measured repeatedly
☐	☒	The statistical test(s) used AND whether they are one- or two-sided<br>*Only common tests should be described solely by name; describe more complex techniques in the Methods section.*
☒	☐	A description of all covariates tested
☒	☐	A description of any assumptions or corrections, such as tests of normality and adjustment for multiple comparisons
☐	☒	A full description of the statistical parameters including central tendency (e.g. means) or other basic estimates (e.g. regression coefficient) AND variation (e.g. standard deviation) or associated estimates of uncertainty (e.g. confidence intervals)
☐	☒	For null hypothesis testing, the test statistic (e.g. F, t, r) with confidence intervals, effect sizes, degrees of freedom and P value noted<br>*Give P values as exact values whenever suitable.*
☒	☐	For Bayesian analysis, information on the choice of priors and Markov chain Monte Carlo settings
☒	☐	For hierarchical and complex designs, identification of the appropriate level for tests and full reporting of outcomes
☒	☐	Estimates of effect sizes (e.g. Cohen's d, Pearson's r), indicating how they were calculated

Our web collection on statistics for biologists contains articles on many of the points above.

Software and code

Policy information about availability of computer code

Data collection	TR-FRET and Lanthascreen measurements were carried out using a PHERAstar FS microplate reader (BMG Labtech). Flow data were collected with a BD FACSDiva 8.0 (BD Biosciences). For reporter assays, the fluorescent signal was quantified using a LSRFortessa flow cytometer (BD Biosciences). Mass spectrometry data were collected using a TimsTOF Pro2 (Bruker Daltonics, Bremen, Germany). The luminescent CTG signal was acquired with CLARIOstar Plus, MARS 3.4 (BMG LabTech).
Data analysis	Crystallographic data processing and refinement was done using pipedream (version 1.2.4) XDS (Jan 31, 2020 (BUILT 20200131), Feb 5, 2021 (BUILT 20210323), Jan 10, 2022 (BUILT 20220820)), AIMLESS (CCP4 suite, versions 0.7.4, 0.7.7), POINTLESS (1.12.1, 1.12.4, 1.12.8, 1.12.10), STARANISO (2.3.36 (20200511)), PHASER (2.8.3), STARANISO (2.3.36 (20200511)), COOT (0.9.6), phenix (1.19.1-4122, 1.20-4459, 1.20.1-4487), BUSTER (2.11.7), ISOLDE (1.3, 1.4), eLBOW (1.19.1-4122, 1.20-4459, 1.20.1-4487), jligand (2.6), PyMOL (2.5), MOLPROBITY/PDB-REDO (4.5.2), CCP4 (7.1.002, 7.1.010, 7.1.013, 7.1.016). Fluorescence polarization fits were done using Prism9 (Graphpad). FlowJo 10.8.1 was used for flow cytometry data analysis. Custom R script were used to analyze cellular assay data as well as RNA sequencing data as described in the Methods. RNAseq data analysis script is provided in the Supplementary Information. The following packages were employed: DESeq2 package (v.1.32.0), ggplot2 (v3.4.2), EnhancedVolcano (v.1.10.0), useful (v1.2.6), dplyr (v1.0.10), reshape2 (v1.4.4), ggpubr (v0.5.0), vsn (v3.66.0), pheatmap (v1.0.12), pals (v1.7), viridis (v0.6.2), stringr (v1.4.1), tidyr (v1.2.1), tidyverse (v1.3.2), ashr (v2.2), ggrepel (v0.9.2) and IHW (1.26.9). The package dr4pl was also used for curve fitting for viability and reporter screens.

For manuscripts utilizing custom algorithms or software that are central to the research but not yet described in published literature, software must be made available to editors/reviewers. We strongly encourage code deposition in a community repository (e.g. GitHub). See the Nature Research guidelines for submitting code & software for further information.

Data

Policy information about availability of data

All manuscripts must include a data availability statement. This statement should provide the following information, where applicable:

- Accession codes, unique identifiers, or web links for publicly available datasets
- A list of figures that have associated raw data
- A description of any restrictions on data availability

Structural data have been deposited in the PDB under the accession codes 8BU1, 8BU2, 8BU3, 8BU4, 8BU5, 8BU6, 8BU7, 8BU9, 8BUA, 8BUB, 8BUC, 8BUD, 8BUE, 8BUF, 8BUG, 8BUH, 8BUI, 8BUJ, 8BUK, 8BUL, 8BUM, 8BUN, 8BUO, 8BUP, 8BUQ, 8BUR, 8BUS, 8BUT. Proteome quantification data are available in the PRIDE repository (PXD041836) or at https://github.com/fischerlab/. The expression constructs used can be identified via the following Uniprot identifiers: DDB1 (Q16531), CDK12 (Q9NYV4, K965R) and CCNK (O75909).

Field-specific reporting

Please select the one below that is the best fit for your research. If you are not sure, read the appropriate sections before making your selection.

☒ Life sciences ☐ Behavioural & social sciences ☐ Ecological, evolutionary & environmental sciences

For a reference copy of the document with all sections, see nature.com/documents/nr-reporting-summary-flat.pdf

Life sciences study design

All studies must disclose on these points even when the disclosure is negative.

Sample size	No sample size calculation was performed. The sample size for the number of replicates (n) for each experiment is provided in the figure captions. All experiments have n equal to or greater than 2.
Data exclusions	No data was excluded from the analysis.
Replication	Number of replicates is indicated in the figure captions. All experiments could be successfully replicated across multiple days. Data represent technical replicates, however drug treatment or perturbation was always performed independently for each replicate.
Randomization	No randomisation was performed as no animal-based experiments were performed.
Blinding	Investigators were not blinded during data collection or analysis. However, controls and samples were analysed in exactly the same way experimentally and using the same computational pipeline.

Reporting for specific materials, systems and methods

We require information from authors about some types of materials, experimental systems and methods used in many studies. Here, indicate whether each material, system or method listed is relevant to your study. If you are not sure if a list item applies to your research, read the appropriate section before selecting a response.

Materials & experimental systems

n/a	Involved in the study	
☐	☒	Antibodies
☐	☒	Eukaryotic cell lines
☒	☐	Palaeontology
☒	☐	Animals and other organisms
☒	☐	Human research participants
☒	☐	Clinical data

Methods

n/a	Involved in the study	
☒	☐	ChIP-seq
☐	☒	Flow cytometry
☒	☐	MRI-based neuroimaging

Antibodies

Antibodies used	Biotin anti-His antibody (ThermoFischer Scientific, cat. no. PV6089, LOT no. 2048928E). This antibody was used in the Lanthascreen assay at a final concentration of 2 nM.
Validation	The antibodies is commercially available and was validated by the manufacturer through a functional assay: "Functional Testing: The performance of each lot of Biotin-anti-His Antibody is confirmed by testing in a LanthaScreen® Eu Kinase Binding Assay."

Eukaryotic cell lines

Policy information about cell lines

Cell line source(s)	The human HEK293T cell lines were provided by the Genetic Perturbation Platform, Broad Institute, MD-MBA-231 cell line was purchased from ATCC, and HEK293TCas9 cells were previously published. Sf9 (purchased from Thermo Fischer Scientific cat. no. 11496-015) and Hi5 (Tni cells, purchased from Expression Systems cat. no. 94-002F) cells were also used in this study.
Authentication	HEK293T, MD-MBA0231, and HEK293TCas9 cells were authenticated by STR profiling. Sf9 and Hi5 cells were authenticated by the vendor.
Mycoplasma contamination	Mycoplasma negative
Commonly misidentified lines (See ICLAC register)	None of commonly misidentified lines were used in this study.

Flow Cytometry

Plots

Confirm that:

☒ The axis labels state the marker and fluorochrome used (e.g. CD4-FITC).

☒ The axis scales are clearly visible. Include numbers along axes only for bottom left plot of group (a 'group' is an analysis of identical markers).

☒ All plots are contour plots with outliers or pseudocolor plots.

☒ A numerical value for number of cells or percentage (with statistics) is provided.

Methodology

Sample preparation	Cells were fixed with 50 µL of 4% paraformaldehyde solution (Chem Cruz, sc-281692) in non-cell culture-treated 384-well plates . The fluorescent signal was quantified by flow cytometry (LSRFortessa flow cytometer, BD Biosciences) using FlowJo (flow cytometry analysis software, BD Biosciences).
Instrument	LSRFortessa (BD)
Software	FlowJo 10.8.1
Cell population abundance	At least 500 mCherry-positive cells were used for each measurement point.
Gating strategy	Cells were first gated for live cells based on forward and side scatter. Single cells were discriminated based on the area vs the height of the side scatter. Finally, reporter-positive cells were considered based on their mCherry expression.

☒ Tick this box to confirm that a figure exemplifying the gating strategy is provided in the Supplementary Information.
